# A cautionary tale: an evaluation of the performance of treatment switching adjustment methods in a real world case study

**DOI:** 10.1186/s12874-024-02140-6

**Published:** 2024-01-22

**Authors:** Nicholas R Latimer, Alice Dewdney, Marco Campioni

**Affiliations:** 1 Sheffield Centre for Health and Related Research (SCHARR), University of Sheffield, Regent Court, 30 Regent Street, Sheffield, South Yorkshire S1 4DA UK; 2 Delta Hat Limited, Nottingham, UK; 3 Weston Park Cancer Centre, Sheffield Teaching Hospital, Sheffield, UK; 4 Amgen (Europe) GmbH, Rotkreuz, Switzerland

**Keywords:** Treatment switching, Treatment crossover, Survival analysis, Cancer, Inverse probability weighting, Rank preserving structural failure time model, Two-stage estimation, Health technology assessment, Adjustment methods

## Abstract

**Background:**

Treatment switching in randomised controlled trials (RCTs) is a problem for health technology assessment when substantial proportions of patients switch onto effective treatments that would not be available in standard clinical practice. Often statistical methods are used to adjust for switching: these can be applied in different ways, and performance has been assessed in simulation studies, but not in real-world case studies. We assessed the performance of adjustment methods described in National Institute for Health and Care Excellence Decision Support Unit Technical Support Document 16, applying them to an RCT comparing panitumumab to best supportive care (BSC) in colorectal cancer, in which 76% of patients randomised to BSC switched onto panitumumab. The RCT resulted in intention-to-treat hazard ratios (HR) for overall survival (OS) of 1.00 (95% confidence interval [CI] 0.82–1.22) for all patients, and 0.99 (95% CI 0.75–1.29) for patients with wild-type KRAS (Kirsten rat sarcoma virus).

**Methods:**

We tested several applications of inverse probability of censoring weights (IPCW), rank preserving structural failure time models (RPSFTM) and simple and complex two-stage estimation (TSE) to estimate treatment effects that would have been observed if BSC patients had not switched onto panitumumab. To assess the performance of these analyses we ascertained the true effectiveness of panitumumab based on: (i) subsequent RCTs of panitumumab that disallowed treatment switching; (ii) studies of cetuximab that disallowed treatment switching, (iii) analyses demonstrating that only patients with wild-type KRAS benefit from panitumumab. These sources suggest the true OS HR for panitumumab is 0.76–0.77 (95% CI 0.60–0.98) for all patients, and 0.55–0.73 (95% CI 0.41–0.93) for patients with wild-type KRAS.

**Results:**

Some applications of IPCW and TSE provided treatment effect estimates that closely matched the point-estimates and CIs of the expected truths. However, other applications produced estimates towards the boundaries of the expected truths, with some TSE applications producing estimates that lay outside the expected true confidence intervals. The RPSFTM performed relatively poorly, with all applications providing treatment effect estimates close to 1, often with extremely wide confidence intervals.

**Conclusions:**

Adjustment analyses may provide unreliable results. How each method is applied must be scrutinised to assess reliability.

**Supplementary Information:**

The online version contains supplementary material available at 10.1186/s12874-024-02140-6.

## Background

Treatment changes are common in randomised controlled trials (RCTs) of new cancer treatments. The randomised treatment may stop working, or may not be tolerable, resulting in a treatment switch. For health technology assessment (HTA), the decision problem usually involves estimating the clinical and cost-effectiveness of inserting a new technology into the treatment pathway, and treatment switches that are representative of realistic treatment pathways that would be observed in standard clinical practice are consistent with this. Difficulties arise when treatment switches are not compatible with treatment pathways that would be observed in standard clinical practice. Most often this occurs when participants randomised to the control group switch onto the experimental treatment. Then it becomes necessary to adjust for the switch – to estimate the treatment effect that would have been observed if switching had not occurred. To this end, statistical adjustment methods are often used to adjust for treatment switching to inform HTA decision making [1–6]. 

A technical support document published by the National Institute for Health and Care Excellence (NICE) Decision Support Unit describes the methods most often used to adjust for treatment switching - Inverse Probability of Censoring Weights (IPCW), Rank Preserving Structural Failure Time Models (RPSFTM) and Two Stage Estimation (TSE) [1, 6]. These methods have been used in several technology appraisals - for example, RPSFTM analyses reduced incremental cost-effectiveness ratios from £90,500 to £31,800 per quality adjusted life year gained in NICE’s appraisal of sunitinib for gastrointestinal stromal tumours [7], and from £75,489 to £52,327 in the appraisal of vemurafenib for malignant melanoma [8]. In both cases the adjustment analysis was accepted, and NICE recommended both treatments [7, 8]. 

Methods such as RPSFTM, IPCW and TSE improve upon naïve adjustment methods, such as simply censoring patients who switch treatments, but each method makes important assumptions and may result in bias if their assumptions do not hold. Several studies report on the performance of adjustment methods in a range of simulated scenarios [9–12]. These studies involve simulating survival data for two treatments in a fictional RCT, simulating treatment switching, and then applying the adjustment methods to estimate what the treatment effect would have been if treatment switching had not occurred. Because the data are simulated, the ‘true’ treatment effect is known, enabling an assessment of the accuracy of the adjustment methods. These studies offer valuable insight and have shown that the RPSFTM performs well across a range of scenarios provided that the treatment effect in switchers and patients initially randomised to the experimental group is similar, and that IPCW and TSE methods are prone to bias when sample sizes are small and switching proportions are high [9–12]. 

However, simulation studies may be criticised due to artificiality: the data are not ‘real’, and results may be influenced by the data generation mechanism. Also, adjustment methods can be applied in a multitude of ways, using different model specifications, but it is difficult to test the performance of these variants in a simulation study in which relatively small numbers of prognostic covariates are simulated. It would therefore be valuable to show how well different applications of adjustment methods work in ‘real’ data. This is usually impossible – we cannot tell how well the methods have performed because we do not know what would have actually happened in the absence of switching. In this study we present an assessment of the performance of adjustment methods in a unique real-world case where we have a very good idea of what the results of an RCT affected by treatment switching would have been if treatment switching had not occurred. First, we present a dual-purpose Methods section – we describe various clinical studies investigating panitumumab in patients with metastatic colorectal cancer, explaining how we have a good idea of the true effect of panitumumab despite the treatment switching observed in the pivotal RCT, then we describe the treatment switching methods and their application in this case study. Next we present results, and discuss their implications for future research and decision making.

## Panitumumab in patients with metastatic colorectal cancer

### Pivotal trial – study 20020408

Study 20020408 (NCT00113763) recruited patients with metastatic colorectal cancer refractory to chemotherapy between 2004 and 2005, and randomised to panitumumab plus best supportive care (BSC) (*n* = 231) and BSC alone (*n* = 232) [13]. The sample size was calculated based on the primary endpoint of progression-free survival (PFS). Overall survival (OS) and best objective response by blinded central review were co-secondary endpoints. Patients randomised to BSC were allowed to switch onto panitumumab after investigator-assessed disease progression, as part of a separate study (Study 20030194, NCT00113776), and 176 did so (221 BSC patients experienced disease progression). Treatment switching was included in the protocol for Study 20020408 based on prior evidence of the activity of panitumumab and cetuximab, a similar anti-EGFR (epidermal growth factor receptor) monoclonal antibody, in colorectal cancer [13]. Data on a range of potentially prognostic variables such as Eastern Cooperative Oncology Group (ECOG) performance status, health related quality of life, response status, lesion characteristics, adverse events, and laboratory tests were collected at baseline and over time. Data were collected at 8-week intervals in the first year, and then every 3 months until centrally reviewed disease progression. For some variables a small number of observations were available beyond disease progression, primarily due to differences in investigator-assessed and centrally reviewed progression times. Appendix A provides more information on data collection.

The intention-to-treat (ITT) hazard ratios (HR) were 0.54 for PFS (95% confidence interval [CI], 0.44–0.66), and 1.00 for OS (95% CI, 0.82 to 1.22) [13]. Post-hoc subgroup analyses were undertaken in an attempt to estimate the effect on OS adjusted for treatment switching. These did not use RPSFTM, IPCW, TSE or any other formal adjustment method, and instead were based on two key assertions. Firstly, a finding made during the study indicated that patients with mutant (MT) KRAS (Kirsten rat sarcoma virus) tumours could not benefit from panitumumab [14]. Secondly, it was assumed that KRAS status is not prognostic for survival in patients treated with BSC, supported by previous research [14–22] and a finding that survival was similar between KRAS groups in BSC patients who did not switch onto panitumumab in Study 20020408 [15]. Two of the post-hoc subgroup analyses were of particular interest for the current study:

i) ‘All-comer’ analysis (to estimate the treatment effect of panitumumab in the full trial population, including all randomised patients irrespective of KRAS type, had there been no switching from BSC to panitumumab): All KRAS assessable patients (MT and wild type [WT]) randomised to panitumumab (*n* = 208) were compared to patients with MT KRAS randomised to BSC (*n* = 100), resulting in an OS HR of 0.76 (95% CI, 0.60 to 0.98) [15].
ii) WT KRAS analysis (to estimate the treatment effect of panitumumab in patients likely to benefit from it – those with WT KRAS – had there been no switching from BSC to panitumumab): Patients with WT KRAS randomised to panitumumab (*n* = 124) were compared to patients with MT KRAS randomised to BSC (*n* = 100), resulting in an OS HR of 0.66 (95% CI, 0.49 to 0.87) [15].

### Evidence from related studies

To provide further evidence on the treatment effect of panitumumab on OS, we considered cetuximab trials. Panitumumab and cetuximab have similar mechanisms of action and the CO.17 study compared cetuximab to BSC in the same population as Study 20020408 and was conducted at a similar time, recruiting 572 patients between 2003 and 2005 [16]. Importantly, treatment switching from BSC onto cetuximab was not permitted. The OS HR for cetuximab was 0.77 (95% CI, 0.64 to 0.92) in the full population [16], and 0.55 (95% CI, 0.41 to 0.74) in the WT KRAS population (0.62, 95% CI 0.44 to 0.87 when adjusted for potentially prognostic variables) [23]. 

Given the similarities between panitumumab and cetuximab, and that both had shown effectiveness for PFS, but only cetuximab had shown effectiveness for OS (likely due to the treatment switching permitted in Study 20020408), it was of interest to compare these two treatments. To that end, the ASPECCT study used a non-inferiority design to assess whether panitumumab preserved the OS benefit previously observed for cetuximab in the CO.17 study [24]. The study recruited patients between 2010 and 2012, by which time it was known that both panitumumab and cetuximab were only effective in patients with WT KRAS, and therefore only patients with WT KRAS were included. The study recruited 999 patients and found an OS HR of 0.97 (95% CI, 0.84 to 1.11), leading to the conclusion that panitumumab is non-inferior to cetuximab [24]. Although concluding non-inferiority does not prove that the treatments have the same effect, the authors of the ASPECCT study further concluded that panitumumab and cetuximab provided similar OS benefits and highlighted that results were also similar for other outcome measures assessed – the PFS HR was 1.00 (95% CI, 0.88 to 1.14), results were consistent across all subgroups, and response rates were almost identical for the two treatments [24]. 

While the ASPECCT study was ongoing, a further study investigating the treatment effect of panitumumab on OS was carried out. The phase 3 RCT Study 20100007 (NCT01412957) was designed with the primary objective of evaluating the effect of panitumumab plus BSC compared to BSC alone on OS in patients with chemo-refractory WT KRAS metastatic colorectal cancer [25]. Study 20100007 therefore essentially replicated Study 20020408, but recruited only WT KRAS patients, included OS as the primary outcome measure, and disallowed treatment switching [25]. 377 patients were recruited between 2011 and 2013, and the HR associated with panitumumab for OS was 0.73 (95% CI, 0.57 to 0.93). The authors suggested that this treatment effect was slightly lower than expected, potentially reflecting an improvement over time in survival for patients receiving BSC [25]. 

### Summary – the effectiveness of panitumumab

Study 20020408 was confounded by treatment switching, but subsequent subgroup analyses based on KRAS type and evidence from other RCTs provide evidence on the likely treatment effect of panitumumab on OS. Table 1 summarises this evidence for two estimands – Estimand 1: All-comers, and; Estimand 2: WT KRAS patients. For Estimand 1, the target is an estimate of the treatment effect in the full trial population, including WT and MT KRAS patients. For Estimand 2, the target is an estimate of the treatment effect in WT KRAS patients.

**Table 1:** Evidence on the effectiveness of panitumumab

Source of evidence	Treatment effect compared to BSC		Comments
	Estimand 1. All comers – OS HR (95% CI)	Estimand 2. WT KRAS – OS HR (95% CI)	
Study 20020408 Panitumumab + BSC (*n* = 231) KRAS assessable (*n* = 208) WT KRAS (*n* = 124) MT KRAS (*n* = 84) BSC alone (*n* = 232) KRAS assessable (*n* = 219) WT KRAS (*n* = 119) MT KRAS (*n* = 100) Study affected by treatment switching from BSC onto panitumumab. Conducted analysis of KRAS groups to ‘adjust’ for switching [15]	Compared WT and MT KRAS patients randomised to panitumumab, to MT KRAS patients randomised to BSC HR = 0.76 (0.60–0.98)	Compared WT KRAS patients randomised to panitumumab, to MT KRAS patients randomised to BSC HR = 0.66 (0.49–0.87)	Assumption that KRAS status is not prognostic for survival, caution required due to breaking of randomisation
Study 20100007 [25] Panitumumab + BSC (*n* = 189) BSC alone (*n* = 188) Study only included patients with WT KRAS. Switching from BSC onto panitumumab not allowed	-	Intention-to-treat analysis HR = 0.73 (0.57–0.93)	Newer study, BSC survival may have improved
CO.17 study [16] Cetuximab + BSC (*n* = 287) BSC alone (*n* = 285) Switching from BSC onto cetuximab not allowed	Intention-to-treat analysis HR = 0.77 (0.64–0.92)	Compared WT KRAS patients randomised to cetuximab, to WT patients randomised to BSC HR = 0.55 (0.41–0.74) [0.62 (0.44–0.87) when adjusted for potentially prognostic covariates]	Assume similar effectiveness for cetuximab and panitumumab, based on ASPECCT study [24]

Notes: BSC - Best Supportive Care; CI - Confidence Interval; HR - Hazard Ratio; KRAS - Kirsten Rat Sarcoma Virus; OS - Overall Survival; WT - Wild Type. For estimand 1, the target is an estimate of the treatment effect in the complete trial population, including WT and MT KRAS patients. For estimand 2, the target is an estimate of the treatment effect in WT KRAS patients

Analyses that break the randomisation of a trial and comparisons between trials should be interpreted with caution. However, it seems reasonable to expect that if treatment switching had not been allowed in Study 20020408, the point-estimate of the OS HR for panitumumab compared to BSC would have been in the region of 0.55 to 0.73 in WT KRAS patients (with 95% confidence intervals ranging between 0.41 and 0.93), and around 0.76 to 0.77 in all-comers (with 95% confidence intervals ranging between 0.60 and 0.98). In the remainder of this paper we apply RPSFTM, IPCW and TSE methods to adjust for the treatment switching observed in Study 20020408, to determine whether they result in estimates of the OS treatment effect of panitumumab that are close to these ‘truths’.

## Treatment switching adjustment methods

Stata Version 17.0 was used to conduct analyses [26]. Detailed information on the application of methods is provided in supplementary materials. Key points are provided here.

### Inverse probability of censoring weights

IPCW censors switchers, and attempts to correct for selection bias induced by censoring by identifying and upweighting patients who have not switched but who have prognostic characteristics similar to those in switchers [27]. The method accounts for baseline and time-dependent confounding using time-dependent weights and is therefore reliant upon the ‘no unmeasured confounding’ assumption. There must also be no prognostic covariates that perfectly predict switching - otherwise no similar patients remain for the IPCW analysis to upweight.

In Study 20020408 information on several potentially prognostic covariates were collected at baseline and over time (see Appendix A), but data were only routinely collected up to the time of disease progression. Covariates to include in the IPCW analyses were determined through discussion with a clinical expert [AD]. Our aim was not to identify variables that improve the predictive ability of our switching models, but to identify confounding variables – those that are causes of switching and survival [28]. The clinical expert was taken through the data available, and the concept of directed acyclic graphs was introduced using simple examples. Variable selection was then based on an assessment of which variables were likely to be common causes of switching and survival. Two model specifications were tested – an ‘inclusive’ model, including all variables considered potentially important, and a ‘reduced’ model, including variables considered most important (see Appendix A). The inclusive models included variables for age, ECOG performance status, region, primary tumour diagnosis, EuroQol 5 dimensions (EQ-5D) score, time of disease progression, best tumour response category, lesion size, serious adverse events, and laboratory values for bilirubin, aspartate transaminase (AST), creatinine, albumin, lactate dehydrogenase, carcinoembryonic antigen, alanine amino transferase, and alkaline phosphatase. Reduced models included variables for ECOG performance status, region, primary tumour diagnosis, EQ-5D score, time of progression, best tumour response category, and lesion size. Very little data were missing at baseline, but data collection reduced over time, with minimal data available after disease progression. The most recently recorded values were used for time-varying covariates (using a last observation carried forward approach). Missing data was not imputed, because in order to be a confounder a variable must be observable to decision makers (the clinician and/or patient). In addition, we created variables to indicate whether data for each variable was missing at the most recent visit, in line with previously published research using IPCW analyses [29]. This resulted in 33 covariates being included in inclusive models, and 15 being included in reduced models.

Only patients with WT KRAS benefit from panitumumab, and therefore in our IPCW analyses we only censored switchers with WT KRAS: MT KRAS switchers were not censored as it was not necessary to adjust their survival times. Therefore, in our analyses we took advantage of our knowledge that patients with MT KRAS do not benefit from panitumumab.

In our primary IPCW analyses, models for the probability of treatment switching used to estimate weights were only applied to BSC patients with WT KRAS, as only WT KRAS switchers were censored. Theoretically this is the correct approach, because only patients who were ‘at risk’ of switching should be weighted: in our analyses, because we only adjust for switching in WT KRAS patients, we essentially define switching to only include patients with WT KRAS and thus only WT KRAS patients are ‘at risk’ of switching. However, this means that weighting models are applied to a small sample, which could result in error. Therefore, we conducted supplementary secondary analyses which included BSC WT KRAS *and* BSC MT KRAS patients in these weighting models, with BSC MT KRAS patients assigned as non-switchers irrespective of whether or not they switched onto panitumumab. This again uses our knowledge that patients with MT KRAS do not benefit from panitumumab, making it reasonable to analyse these patients as non-switchers, and allowing us to increase the sample size used in the weighting models. This approach requires the additional assumption that KRAS status is not predictive of survival other than through treatment with panitumumab, which is supported by previous research [14–22]. 

We used an IPCW-weighted Cox proportional hazards model to estimate the HR adjusted for treatment switching and conducted analyses with stabilised and unstabilised weights, and with inclusive and reduced models, for Estimands 1 and 2. This resulted in 4 primary analyses for each estimand, and 4 secondary analyses for each estimand (see Appendix A for further details).

### Two-stage estimation

TSE estimates the effect of treatment switching by comparing switchers to non-switchers after a disease related secondary baseline. The effect estimate is used to derive counterfactual survival times for switchers [10, 12]. The simple version of the TSE method (TSEsimp) uses simple regression to estimate the effect of switching, whereas a more complex version uses g-estimation (TSEgest) [12]. 

Treatment switching was permitted after disease progression in Study 20020408, so this was used as the secondary baseline. The ‘inclusive’ and ‘reduced’ model specifications described for IPCW were used, but, because simple regression cannot deal with time-dependent confounding, our TSEsimp analyses did not use values of variables measured after disease progression. TSEsimp assumes no unmeasured confounding at the secondary baseline time-point *and* that no confounding occurs between the secondary baseline and the time of switch [10]. TSEgest can include time-dependent confounding variables, so does not need to assume that no confounding occurs between the secondary baseline and the time of switch, but only offers advantages over TSEsimp if useful data are collected during this period [12]. 

In our primary TSE analyses we compared BSC WT KRAS switchers to BSC WT KRAS non-switchers to estimate the effect of switching. In common with our IPCW analyses, we conducted secondary analyses which compared BSC WT KRAS switchers to BSC WT KRAS non-switchers *and* BSC MT KRAS patients (irrespective of switch status) to estimate the effect of switching – again taking advantage of our knowledge that patients with MT KRAS do not benefit from panitumumab and allowing models for the effect of switching to be applied to a larger sample of patients.

Cox proportional hazards models were applied to TSE-adjusted datasets to estimate the switching-adjusted HR, for Estimands 1 and 2. We tested various different AFT models for TSEsimp, applied TSEgest using different g-estimation procedures, and tested both models with and without re-censoring, and with inclusive and reduced models. This resulted in 8 primary analyses for each estimand for each method, and 8 secondary analyses for each estimand for each method (see Appendix A). The Stata command stgest3 was used to implement TSEgest [12]. 

### Rank preserving structural failure time model

The RPSFTM differs importantly from IPCW and TSE because it does not require the no unmeasured confounding assumption. Instead, it uses g-estimation and assumes that there is a ‘common treatment effect’ – the treatment effect in switchers must be the same as in patients originally randomised to the experimental group [30]. 

In the context of Study 20020408, the RPSFTM method is problematic, because there was substantial treatment effect heterogeneity: WT KRAS patients benefited from panitumumab whereas MT KRAS patients did not. We addressed this by specifying the RPSFTM such that MT KRAS patients received zero benefit from panitumumab.

Cox proportional hazards models were applied to RPSFTM-adjusted datasets to estimate the switching-adjusted HR, for Estimands 1 and 2. We tested RPSFTM applied on an ‘ever treated’ and an ‘as treated’ basis [1], using alternative g-estimation processes (including iterative parameter estimation [IPE] [31]), with and without re-censoring [32–34]. This resulted in 12 analyses for each estimand (see Appendix A). The Stata command strbee was used to implement RPSFTM [35]. 

## Results

Table 2 presents the KRAS and switch status of patients recruited to Study 20020408.

**Table 2:** KRAS and switch status of patients in Study 20020408

	BSC (*n* = 232)	Panitumumab plus BSC (*n* = 231)
MT KRAS	100	84
WT KRAS	119	124
Unknown KRAS	13	23
MT KRAS switchers	77 (23 non-switchers)	-
WT KRAS switchers	91 (28 non-switchers)	-
Switchers with unknown KRAS	8	-

Notes: BSC - Best Supportive Care; KRAS - Kirsten Rat Sarcoma Virus; MT - Mutant Type; WT - Wild Type

**Fig. 1:**
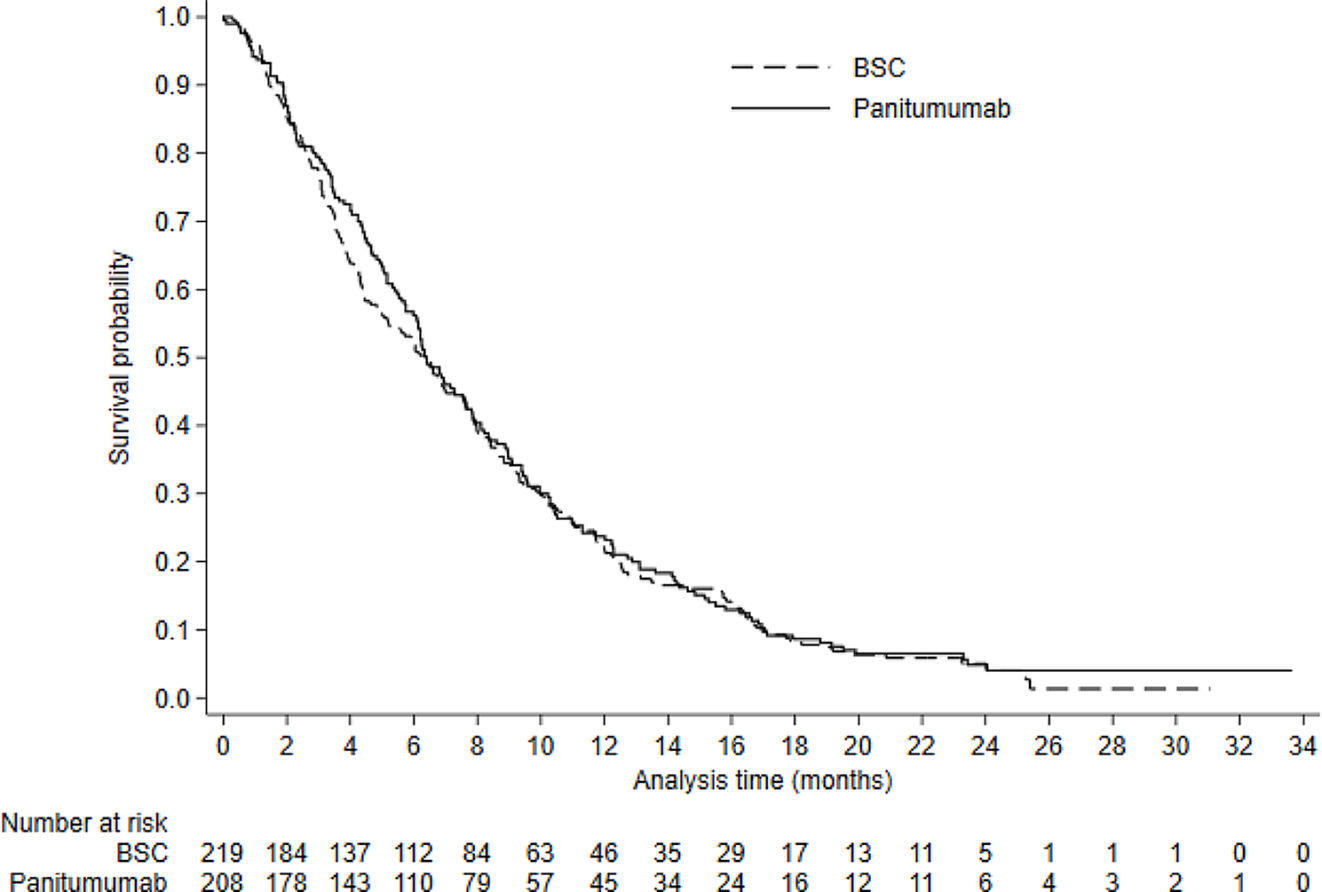
Kaplan-Meier overall survival in KRAS identifiable patients Notes: BSC - Best Supportive Care; KRAS - Kirsten Rat Sarcoma Virus

**Fig. 2:**
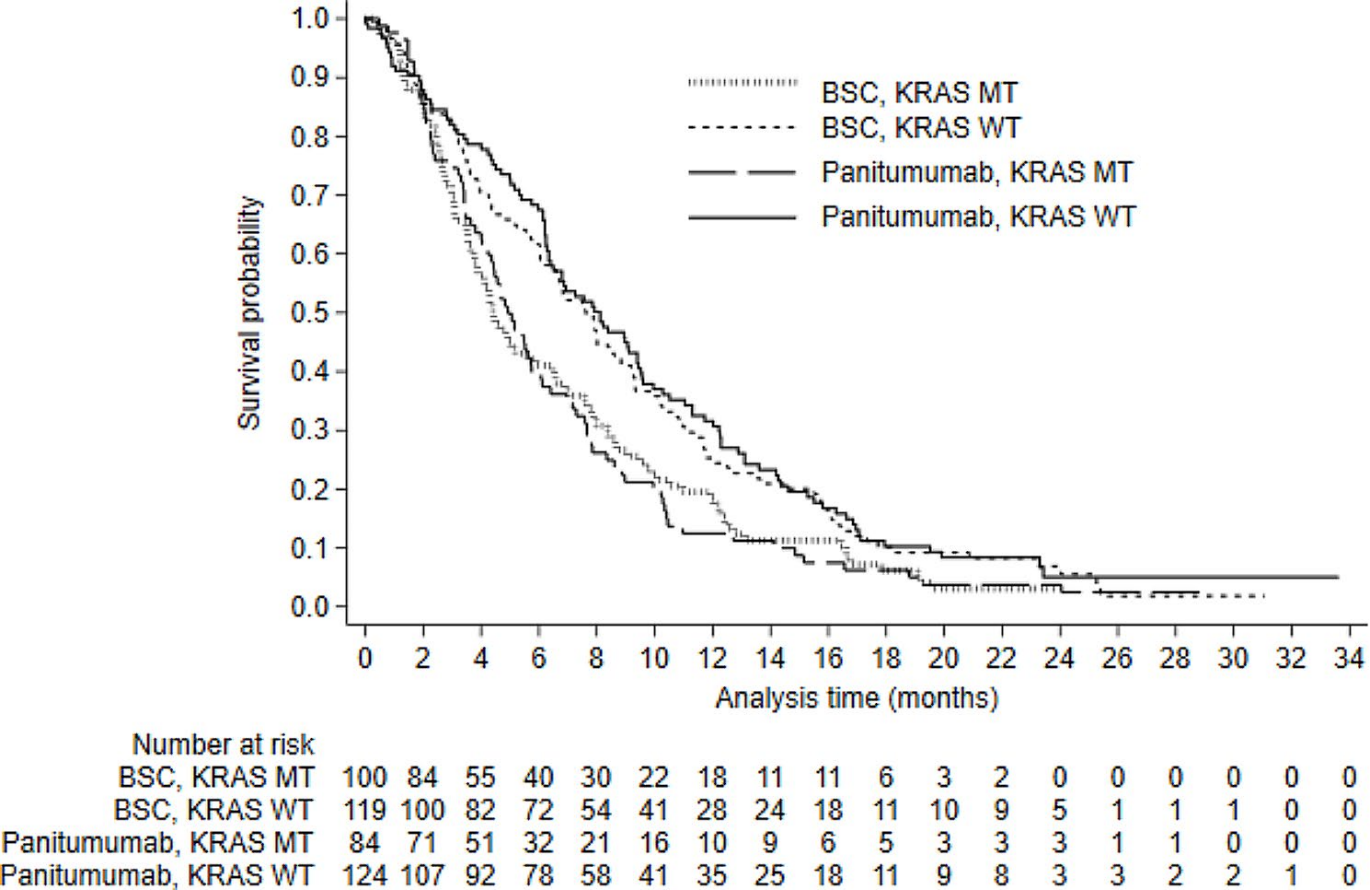
Kaplan-Meier overall survival in KRAS identifiable patients – by KRAS status Notes: BSC - Best Supportive Care; KRAS - Kirsten Rat Sarcoma Virus; MT - Mutant Type; WT - Wild Type

**Fig. 3:**
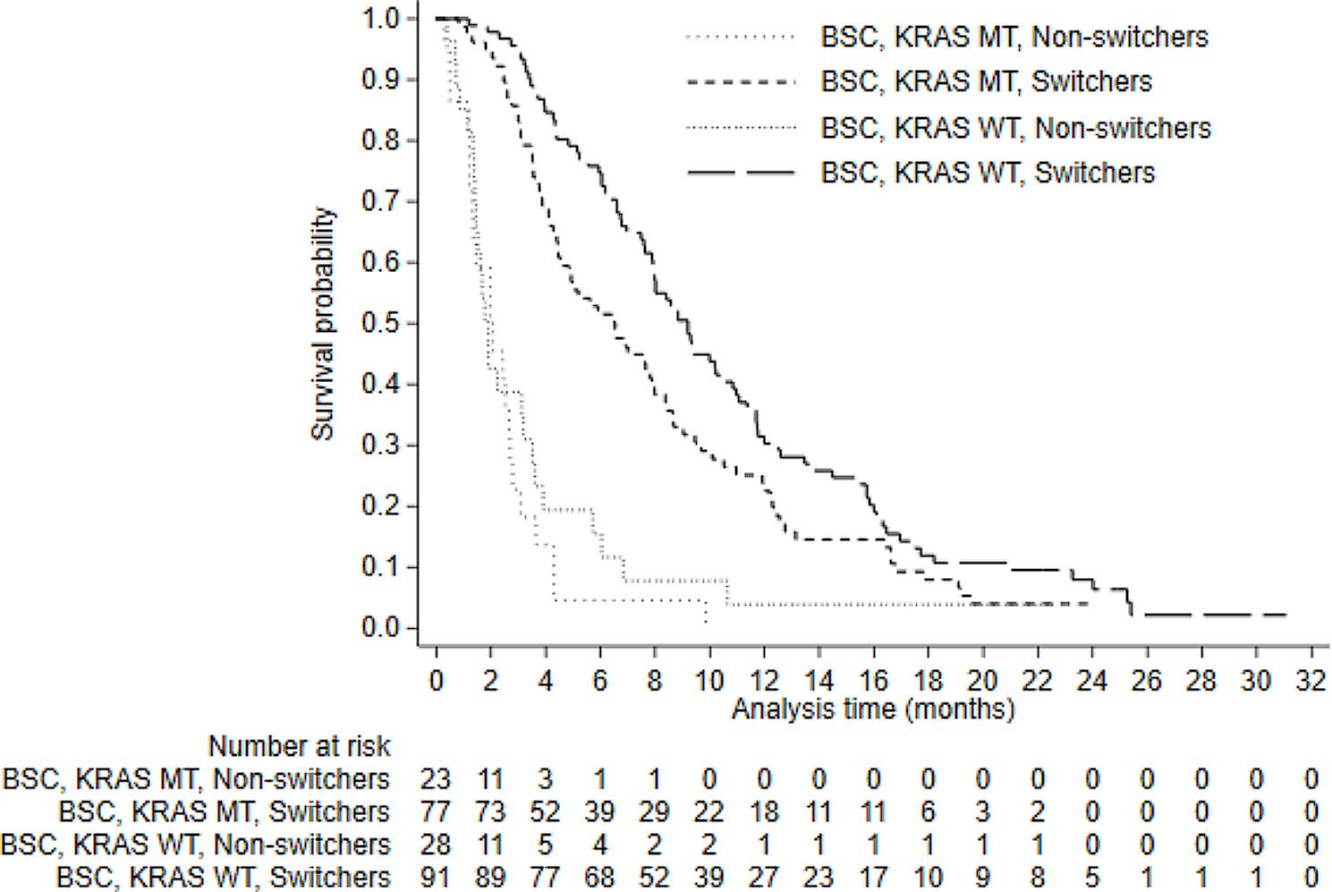
Kaplan-Meier overall survival in BSC KRAS identifiable patients – by KRAS and switch status Notes: BSC - Best Supportive Care; KRAS - Kirsten Rat Sarcoma Virus; MT - Mutant Type; WT - Wild Type. These curves cannot be used to compare survival because they are subject to immortal time bias. Curves are shown to illustrate poor prognosis in non-switchers: only 8 BSC non-switchers survived longer than 4 months

Patient experience and time-to-switch plots are presented in Appendix B. These demonstrate that approximately 95% of switching occurred within 1 month of disease progression. Figures 1, 2 and 3 present Kaplan-Meier survival curves for KRAS assessable patients. Survival differed substantially across KRAS groups, but there was minimal difference between patients randomised to panitumumab and BSC (Figs. 1 and 2). ITT analyses (unadjusted for treatment switching) for both estimands resulted in HRs close to 1 (0.97, 95% CI: 0.79–1.18 for Estimand 1; 0.99, 95% CI: 0.76–1.30 for Estimand 2). Figure 3 presents survival by switch and KRAS status in the BSC group. These curves should not be used to compare survival amongst these groups because they are conditioned on a post-randomisation event (switching) – we present them here only to illustrate that just 8 BSC non-switchers survived longer than 4 months, indicating that non-switchers had extremely poor prognosis.

Table 3 presents results for selected key adjustment analyses. One analysis is presented for RPSFTM, and two analyses are presented for IPCW, TSEsimp and TSEgest (because primary and secondary analyses were specified for these methods). For IPCW, TSEsimp and TSEgest we selected analyses that used ‘inclusive’ rather than ‘reduced’ models for presentation in Table 3, because inclusive models reduce the likelihood of unmeasured confounding. For IPCW, we present results for applications that used stabilised rather than unstabilised weights due to the potential for extreme weights given the small sample sizes included in our analyses. For TSEsimp and TSEgest, we present results for applications that included re-censoring to avoid the possibility of informative censoring in the counterfactual datasets. For TSEsimp we present results for applications that used Weibull AFT models to estimate the treatment effect in switchers, because these resulted in lower Akaike Information Criterion (AIC) values than other AFT models. For TSEgest we present results from applications that used interval bisection rather than a grid search within the g-estimation procedure. For RPSFTM, we present results from an application that included re-censoring (to be consistent with analyses presented for TSEsimp and TSEgest) and interval bisection (to be consistent with analyses presented for TSEgest). Results for all other applications of the adjustment methods are presented in Appendix C, and, where relevant, these are referred to below. Code and statistical output are provided in Appendix D.

**Table 3:** Results, Overall Survival, KRAS identifiable population

Analysis^*^	Estimand 1 (all KRAS assessable patients) hazard ratio (95% CI)	Estimand 2 (KRAS WT) hazard ratio (95% CI)
Expected ‘truth’^§^	0.76–0.77 (0.60–0.98)	0.55–0.73 (0.41–0.93)
ITT	0.97 (0.79–1.18)	0.99 (0.75–1.29)
IPCW 1 (primary analysis, inclusive model, stabilised weights)	0.75 (0.59–0.95)	0.55 (0.28–1.09)
IPCW 5 (secondary analysis, inclusive model, stabilised weights)	0.75 (0.59–0.96)	0.65 (0.50–0.85)
TSEsimp 1 (primary analysis, inclusive model, with re-censoring)	0.64 (0.46–0.98)	0.44 (0.24–0.95)
TSEsimp 9 (secondary analyses, inclusive model, with re-censoring)	0.70 (0.54–0.90)	0.59 (0.43–0.78)
TSEgest 1 (primary analysis, inclusive model, with re-censoring)	0.54 (0.30–1.51)	0.31 (0.13–2.12)
TSEgest 9 (secondary analyses, inclusive model, with re-censoring)	0.79 (0.57–0.99)	0.67 (0.46–0.86)
RPSFTM 1 (with re-censoring, ever treated, interval bisection)	0.91 (0.49–1.68)	0.87 (0.02–35.88)

Notes: CI - Confidence Interval; IPCW - Inverse Probability of Censoring Weights; ITT - Intention to treat; KRAS - Kirsten Rat Sarcoma Virus; RPSFTM - Rank preserving structural failure time model; TSEgest - Two-stage estimation with g-estimation; TSEsimp - Two-stage estimation with simple regression; WT - Wild Type. Confidence intervals for TSE analyses were calculated by bootstrapping the entire adjustment analyses, with 5,000 samples. For TSEgest 1 the g-estimation process did not converge in 17% of samples. For TSEgest 9 the g-estimation process did not converge in 4% of samples

^*^For IPCW, primary analyses only included patients with WT KRAS in models used to obtain weights. Secondary analyses added BSC MT KRAS patients to the weighting models, under the assumption that KRAS was not prognostic for survival in patients receiving BSC. For TSE, primary analyses compared BSC WT KRAS switchers to BSC WT KRAS non-switchers to estimate the effect of switching. Secondary analyses compared BSC WT KRAS switchers to BSC WT KRAS non-switchers and BSC MT KRAS patients (irrespective of switch status) to estimate the effect of switching. ‘Inclusive’ models included all variables considered potentially important, and ‘reduced’ models included only those variables considered most important. The analysis number refers to the way in which the method was applied - see supplementary materials for further details

^§^The expected truths are taken from Table 1, with the point-estimate range representing the range of point-estimates observed in the relevant analyses from Study 20020408, 20100007 and CO.17, and the confidence interval range representing the minimum and maximum 95% confidence intervals reported for these point-estimates

The primary and secondary IPCW analyses provided treatment effect estimates that closely matched the expected true point-estimates and CIs for Estimand 1, irrespective of whether inclusive or reduced models or stabilised or unstabilised weights were used (IPCW 1 [primary analysis] HR 0.75, 95% CI: 0.59–0.95; IPCW 5 [secondary analysis] HR 0.75, 95% CI: 0.59–0.96, Table 3 and Appendix C). For Estimand 2, the primary IPCW analyses provided treatment effect estimates that fell within the 95% confidence interval of the expected truth, but point estimates were towards the lower end (that is, further from the null) of the range expected (HR 0.55, 95% CI: 0.28–1.09 for IPCW 1, Table 3). Secondary IPCW analyses provided estimates for Estimand 2 that more closely matched the expected true point-estimates and CIs (HR 0.65, 95% CI: 0.50–0.85 for IPCW 5, Table 3 and similar results for alternative secondary analysis applications reported in Appendix C). For IPCW 1, the primary analysis included in Table 3, weights ranged from 0.05 to 14.4. Weight ranges in secondary analyses were much lower: for IPCW 5, the secondary analysis included in Table 3, weights ranged between 0.47 and 5.7. Using inclusive or reduced models or stabilised or unstabilised weights had little impact on treatment effect estimates, with the only factor that made an appreciable difference to the IPCW results being the use of secondary analyses rather than primary analyses for Estimand 2 (Appendix D).

The primary TSEsimp analyses resulted in estimates of the treatment effect that were close to the lower 95% confidence limit of the expected truth for both estimands (Estimand 1 HR 0.64, 95% CI: 0.46–0.98, Estimand 2 HR 0.44, 95% CI: 0.24–0.95 for TSEsimp 1, Table 3), with some analyses providing point estimates that fell below the lower expected true confidence interval limit, indicating that the treatment effect may have been over-estimated (see TSEsimp 5 and TSEsimp 6, Appendix C). In contrast, the secondary TSEsimp analyses provided treatment effect estimates that more closely matched the expected truths (Estimand 1 HR 0.70, 95% CI: 0.54–0.90; Estimand 2 HR 0.59, 95% CI: 0.43–0.78) (Table 3). The type of AFT model used to estimate the treatment effect in switchers had very little impact on the results, whereas excluding re-censoring resulted in marginally lower treatment effect estimates (Appendix C). Within the secondary analyses, using a reduced model specification also resulted in marginally lower treatment effect estimates (Appendix C).

The primary TSEgest analyses consistently over-estimated the treatment effect for both estimands, resulting in point estimates that fell below the lower confidence limit of the expected truth – although the TSEgest confidence intervals were very wide and overlapped with those of the expected truths (Estimand 1 HR 0.54, 95% CI: 0.30–1.51, Estimand 2 HR 0.31, 95% CI: 0.13–2.12 for TSEgest 1, Table 3). Primary TSEgest analyses that used reduced models and excluded re-censoring resulted in numerically higher HRs that fell within the range of the expected truths (see TSEgest 7 and TSEgest 8, Appendix C). The secondary TSEgest analyses provided estimates of the treatment effect that closely matched the expected true point-estimates and CIs for both estimands (Estimand 1 HR 0.79, 95% CI: 0.57–0.99; Estimand 2 HR 0.67, 95% CI: 0.46–0.86) (Table 3). Secondary TSEgest analyses that used a reduced model specification and excluded re-censoring resulted in marginally lower treatment effect estimates (Appendix C). Results were not impacted by whether an interval bisection of grid search process was used within the g-estimation procedure.

The RPSFTM analyses provided point estimates of the treatment effect that were close to 1 for both estimands (Estimand 1 HR 0.91, 95% CI: 0.49–1.68; Estimand 2 HR 0.87, 95% CI: 0.02–35.88 for RPSFTM 1, Table 3) across all analyses (Appendix C), indicating that the treatment effect may have been under-estimated. However, point-estimates did fall within the 95% confidence intervals of the expected truths in the majority of analyses, and the RPSFTM confidence intervals were extremely wide, overlapping those of the expected truths in all analyses. Results were unaffected by whether or not re-censoring was incorporated, or whether interval bisection or a grid search was used within the g-estimation process. However, confidence intervals were narrower when iterative parameter estimation was used in place of g-estimation (Appendix C).

## Discussion

In this study our intent was to demonstrate the performance of treatment switching adjustment methods in a real case study where the treatment effect in the absence of switching is approximately known, building on studies that have demonstrated the performance of these methods in simulated datasets [9–12]. Study 20020408, comparing panitumumab to BSC in patients with metastatic colorectal cancer, represented an ideal case study; switching occurred in the study, but estimates of the treatment effect that would have been observed in the absence of switching could be ascertained from similar studies that did not permit switching, and from post-hoc subgroup analyses of Study 20020408. We found that some adjustment analyses provided point estimates and CIs for the treatment effect that closely matched the expected truths, whereas others provided point estimates that fell outside the expected true confidence intervals, and some analyses provided confidence intervals that were extremely wide: it was clear that some adjustment analyses performed better than others. By considering methodological assumptions and the characteristics of the trial and the data, it is possible to understand why some analyses performed well and others did not, providing valuable learning for future applications of adjustment methods.

RPSFTM analyses performed relatively poorly. Although treatment effect estimates generally fell within the 95% confidence intervals of the expected truths, the point estimates were closer to 1 than expected and confidence intervals were extremely wide, indicating a lack of precision. This can be explained by considering how the method works and the ITT trial results. In Study 20020408 the ITT analyses provided HRs close to 1. The RPSFTM method compares exposure to the treatment and outcomes in each randomised group, and estimates a treatment effect that would result in the two randomised groups having equal average survival times in a counterfactual world where no patients received any treatment. If there is very little difference in observed outcomes between randomised groups, the RPSFTM will attribute only a very small treatment effect to any additional exposure to treatment that is present in either randomised group. Fundamentally, the RPSFTM will only successfully identify treatment effects if there are appreciable differences in exposure to the experimental treatment in the two randomised groups and if this results in an appreciable difference in outcomes in an ITT analysis. This was not the case in Study 20020408.

For the IPCW and TSE adjustment methods a key concern was that very little information on potentially confounding variables was collected after disease progression in Study 20020408. IPCW and TSEgest require information on variables that are time-dependent confounders, and TSEsimp requires that there is no time-dependent confounding between the time of disease progression and the time of switch. Therefore, IPCW and TSEgest are prone to bias if data collection on confounding variables stops a considerable amount of time before switching could occur, and TSEsimp is prone to bias if there is a considerable gap between the time of disease progression and the time of switch. In Study 20020408, however, the vast majority of switching happened very soon after disease progression. This means that the scope for bias due to unmeasured confounding should be low for the IPCW and TSE methods, providing that relevant confounders are captured in the ‘inclusive’ and ‘reduced’ models that we used to apply them.

Primary and secondary IPCW analyses performed well for both treatment effect estimands, although point estimates from the primary analyses for Estimand 2 were towards the lower end of the expected range – for this estimand the secondary analyses more closely matched the expected true point-estimates and CIs. The primary IPCW analyses used the same weights for both estimands, suggesting that the potential problem with Estimand 2 arose from the population to which the weights were applied. For Estimand 2, the treatment effect was estimated specifically for WT KRAS patients – MT KRAS patients were excluded in the primary IPCW analysis and because very few BSC WT KRAS non-switchers survived beyond 4 months, and BSC WT switchers were censored at the time of switch, this analysis included very little long-term information in the BSC group. As a result, the analysis may have over-estimated the treatment effect. This problem was avoided in the secondary IPCW analyses, which included BSC MT KRAS patients as non-switchers (irrespective of their actual switch status), under the assumption that patients with MT KRAS do not benefit from panitumumab. This increased the sample size for the analysis, and resulted in estimates of the treatment effect that more closely represented the expected truths.

Our primary TSE analyses appeared to perform poorly – TSEsimp resulted in estimates of the treatment effect that were close to the lower limit of the 95% CI of the expected truth for both estimands, and TSEgest resulted in very wide CIs indicating a lack of precision, and point-estimates that fell below the lower limit of the CI of the expected truth. These primary analyses compared post-progression survival in 91 BSC WT KRAS switchers and 28 BSC WT KRAS non-switchers to estimate the effect of switching. Only 5 BSC WT KRAS non-switchers survived beyond 4 months, which appears to have prevented the TSE models from accurately estimating the effect of switching. A key difference between TSE and IPCW is that TSE requires the effect of switching to be estimated, whereas IPCW does not. This explains the apparent better performance of the IPCW primary analyses compared to the TSE primary analyses in this case study.

Secondary TSE analyses performed substantially better than the primary analyses, with both TSEsimp and TSEgest producing point estimates and confidence intervals that were consistent with the expected truths. These secondary analyses estimated the effect of switching by comparing post-progression survival in the 91 BSC WT KRAS switchers to the 28 BSC WT KRAS non-switchers *and* 100 BSC MT KRAS patients (some of whom had relatively good survival). This increased sample size appears to have allowed the effect of switching to be estimated more accurately.

However, results of secondary analyses for TSEsimp and TSEgest did differ, with TSEgest consistently producing point estimates and confidence limits that were approximately 0.1 higher (closer to 1) than those provided by TSEsimp. Given that switching occurred soon after disease progression, and that very little prognostic data were collected after progression, there may be grounds to prefer TSEsimp in this case study. Model specification and re-censoring also had a potentially important impact. ‘Inclusive’ models included 33 covariates, with 15 included in ‘reduced’ models. Although the reduced models may increase the possibility of unmeasured confounding, these still included a reasonably large number of variables, and all those considered most likely to be important confounders by a clinical expert [AD]. 189 deaths occurred amongst BSC patients who experienced disease progression, so the 33 covariates included in the inclusive models may risk over-fitting, and therefore the reduced models may be preferred [36, 37]. Re-censoring is a concern when it results in a substantial loss of data [32], but in this case it reduced the number of observed events only by 5. Therefore, secondary TSEsimp analyses which used reduced models and included re-censoring may be preferable in this case. These provided results close to those provided by secondary IPCW analyses with reduced models (TSEsimp 13 Estimand 1 HR 0.76, 95% CI: 0.58–0.95; Estimand 2 HR 0.63, 95% CI: 0.46–0.83; IPCW 7 Estimand 1 HR 0.79, 95% CI: 0.63-1.00; Estimand 2 HR 0.69, 95% CI: 0.53–0.90, see Appendix C). Notably, these results closely match the expected true point-estimates and CIs, and fall closely on either side of the HRs estimated by Poulin-Costello et al. when attempting to adjust for the treatment switching observed in Study 20020408 purely by making comparisons between various MT and WT KRAS groups (Estimand 1 HR 0.76, 95% CI: 0.60–0.98; Estimand 2 HR 0.66, 95% CI: 0.49–0.87, see Table 1) [15]. 

It is valuable to compare our findings to those found in simulation studies. In the most recently published simulation study that compared each of the methods included in our study, Latimer et al. found that TSEgest, IPCW and RPSFTM produced low bias in scenarios with a similar sample size, switching proportion, and treatment effect to those observed in Study 20020408 [12]. TSEsimp resulted in similarly low levels of bias when there was no time-dependent confounding between the time of disease progression and the time of switch, but produced moderate bias (5–6%) when important time-dependent confounding was present – in Study 20020408 it appears that the scope for time-dependent confounding occurring between the time of disease progression and the time of switch was low, because switching happened soon after disease progression. Latimer et al. found that IPCW became prone to substantial bias when maximum weights exceeded 10% of the size of the treatment group in which switching occurred. In our IPCW analyses of Study 20020408, the maximum weights as a proportion of the group being weighted were approximately: 6% for the primary analysis for Estimand 1; 12% for the primary analysis for Estimand 2; 2% for the secondary analyses for both estimands.

Therefore, based on Latimer et al.’s simulation study, we might expect each of the adjustment methods to produce low levels of bias in our analyses of Study 20020408, with some concerns around the primary IPCW analyses for Estimand 2. In fact, whilst the IPCW performed as expected based on the size of the weights estimated – that is, performance was good across analyses but appeared prone to error in primary analyses for Estimand 2 – we found that the RPSFTM appeared to perform relatively poorly, as did the primary TSEsimp and TSEgest analyses. This is likely to be because the characteristics of Study 20020408 deviated from scenarios included in Latimer et al.’s simulation study in two important ways. Firstly, simulation studies have not considered scenarios where the ITT treatment effect is equivalent to a HR of approximately 1. This explains why the RPSFTM appears to have performed relatively poorly in our analyses of Study 20020408, as discussed previously. Secondly, the nature of the switching observed in Study 20020408 differed from that assessed in simulation studies – the switching proportion was not excessively high, but non-switchers appear to have had extremely poor prognosis, with very few surviving beyond 4 months. This appears to have severely restricted the ability of our primary TSE analyses to accurately estimate the effect of switching, leading to performance that is worse than might have been expected based purely on a consideration of switching proportion and sample size.

Consequently, whilst our results generally support those found in simulation studies [9–12], we provide further insight on the limitations associated with the RPSFTM, and on nuances around IPCW and TSE analyses. We show that it is not simply the number of switchers and non-switchers that is important – even if the switching proportion is not excessively high, adjustment methods will be prone to error if non-switchers are prognostically very different from switchers. In Study 20020408 this was indicated by very short survival times in non-switchers, resulting in inadequate numbers at risk over time to generate reliable treatment effect estimates both in switchers (for TSE) and in the adjusted population (for IPCW).

Our analysis provides evidence on how adjustment methods work in one real-world case study where the expected ‘truth’ is known, demonstrating for the first time how well these methods can work in a real-world setting. While it is reassuring that several of our analyses provided results that are close to the expected truths in this case study, it is important to acknowledge that several applications of methods produced results that appeared to somewhat over- or under-estimate the treatment effect.

We have shown that it is possible to explain why adjustment analyses perform poorly, but it may not be obvious a priori that poor performance will arise. This illustrates the importance of considering how adjustment methods are applied and their assumptions in combination with the characteristics of the data. This also emphasises the importance of following recently published reporting guidelines, to ensure that adjustment analyses can be reviewed adequately [38]. Our study provides valuable evidence on the practicalities and performance of adjustment methods, to supplement evidence from simulated scenarios.

## Conclusions

It is often necessary to adjust for treatment switching in RCTs to provide analyses that directly address decision problems faced in HTA. However, adjustment analyses may not provide accurate or reliable results. Our study provides a cautionary tale demonstrating that data, analyses, and assumptions should be scrutinised to assess the reliability of adjustment analyses, otherwise treatment effects could be importantly over- or under-estimated, resulting in poor reimbursement decisions.

### Electronic supplementary material

Below is the link to the electronic supplementary material.

**Supplementary Material 1: Appendix A:** Details on Application of Adjustment Methods. **Appendix B:** Patient Experience and Time-to-Switch Plots. **Appendix C:** Results From all Analyses. **Appendix D:** Full Coding and Results for all Analyses. **Appendix E:** Independent Ethics Committees for Study 20020408 and Study 20030194

## Data Availability

Amgen provided access to the results of studies 20020408 (NCT00113763) and 20030194 (NCT00113776) to the University of Sheffield via a data sharing agreement. Qualified researchers may request data from Amgen clinical studies. Complete details are available at http://www.amgen.com/datasharing. Please contact n.latimer@sheffield.ac.uk for more information.

## Abbreviations

BSC: Best supportive care
CI: Confidence interval
HR: Hazard ratio
HTA: Health technology assessment
IPCW: Inverse probability of censoring weights
IPE: Iterative parameter estimation
ITT: Intention-to-treat
KRAS: Kirsten rat sarcoma virus
MT: Mutant type
NICE: National institute for health and care excellence
OS: Overall survival
PFS: Progression-free survival
RCT: Randomised controlled trial
RPSFTM: Rank preserving structural failure time model
TSE: Two-stage estimation
TSEgest: Two-stage estimation with g-estimation
TSEsimp: Two-stage estimation with simple regression
WT: Wild type
