# Supplementary material for: A cautionary tale: an evaluation of the performance of treatment switching adjustment methods in a real world case study
Source: BMC Med Res Methodol. 2024 Jan 22;24:17. doi: 10.1186/s12874-024-02140-6 (PMC10802004; doi:10.1186/s12874-024-02140-6)
Supplement: Supplementary file 1 — Supplementary Material 1: Appendix A: Details on Application of Adjustment Methods. Appendix B: Patient Experience and Time-to-Switch Plots. Appendix C: Results From all Analyses. Appendix D: Full Coding and Results for all Analyses. Appendix E: Independent Ethics Committees for Study 20020408 and Study 20030194 [file 12874_2024_2140_MOESM1_ESM.docx]

**A Cautionary Tale: An Evaluation of the Performance of Treatment Switching Adjustment Methods in a Real World Case Study: Supplementary Materials:**

Contents

Page

Appendix A: Details on Application of Adjustment Methods.…………………... 2

Appendix B: Patient Experience and Time-to-Switch Plots.…………………….. 15

Appendix C: Results From all Analyses………………….………………............ 17

Appendix D: Full Coding and Results for all Analyses.…………………………. 23

Appendix E: Independent Ethics Committees for Study 20020408 and

Study 20030194……………………………………………………. 432

**Appendix A: Details on Application of Adjustment Methods**

Randomisation in Study 20020408 was stratified by ECOG performance status and region, and hazard ratios (HRs) for all reported analyses were estimated with Cox proportional hazards models adjusted for these factors.^1,2,3^ Therefore, to enable comparability, each of the adjustment analyses we report HRs that include adjustment for ECOG performance status and region.

***Inverse Probability of Censoring Weights***

The IPCW method censors switching patients at the time at which they switch treatments. However, the method then applies time-dependent weights to each remaining observation for each patient in order to account for any prognostic differences that may exist between switching and non- switching patients – thereby correcting for the selection bias induced by the initial censoring.^4^ The method relies upon a ‘no unmeasured confounders’ assumption – that is, data must be available for all baseline and time-dependent characteristics that influence the probability of switching, and the risk of death. There must also be no prognostic covariates that perfectly predict switching – known as the positivity assumption – because if, for example, all “good” prognosis patients switch treatments, no similar patients remain for the IPCW analysis to upweight. Previous research has shown that the method is prone to bias when switching proportions are very high (resulting in very small numbers of patients being allocated relatively high weights).^5-7^

The time-dependent weights used in IPCW represent the inverse probability of remaining uncensored (i.e. not switching to panitumumab) over time. The probability of remaining uncensored over time is estimated using logistic regression, including baseline and time-dependent variables that are likely to impact the switching decision and the survival outcome. Often “stabilised” weights are used, where the numerator of the weight (which equals ‘1’ in an unstabilised analysis) is replaced by the probability of remaining uncensored estimated without including time-dependent information.

Given the reliance of the IPCW method on the no unmeasured confounding assumption, it is important to consider the data collected in Study 20020408. Tables of the variables collected are presented in Tables A1-A12. These include information on laboratory tests, response status, ECOG, age, sex, prior treatment, time since diagnosis, quality of life, lesion characteristics, progression status, and adverse events. Tables A2-A12 list variables for which data were collected at baseline and over time. Data were collected at 8-week intervals in the first year, and then every 3 months until centrally reviewed disease progression. For some variables a small number of observations were available beyond disease progression, primarily due to differences in investigator-assessed and centrally reviewed progression times. The relative sparcity of data collected beyond disease progression means that the IPCW analyses are prone to bias if switching occurred substantially after the time of disease progression.

Table A1: “Baseline” dataset variables, Study 20020408

| Variable name | Label |
| --- | --- |
| SUBJID | Subject ID |
| AGE | Age in Years at Screening |
| SEX | Sex |
| KRAS | KRAS |
| B_ECOG | Baseline ECOG Performance Status |
| CHANY | Prior Chemotherapy |
| DIAGMONS | Months Since Primary Diagnosis |
| WBC | Baseline Peripheral WBC count (10^9/L) |
| ANC | Baseline Absolute Neutrophil Count (10^9/L) |
| NEUTPCT | Baseline Peripheral Neutrophil % |
| HGB | Baseline Hemoglobin (g/L) |
| PLT | Baseline Peripheral Platelet Count (10^9/L) |
| BILI | Baseline Bilirubin (umol/L) |
| MG | Baseline Magnesium (mmol/L) |
| AST | Baseline Aspartate Transaminase (U/L) |
| ASTULN | Baseline Aspartate Transaminase (ULN) (U/L) |
| CREAT | Baseline Creatinine (umol/L) |
| CREATULN | Baseline Creatinine (ULN) (umol/L) |
| ALB | Baseline Albumin (g/L) |
| LDH | Baseline Lactate Dehydrogenase (U/L) |
| DIAGTYPE | Primary Tumor Diagnosis |
| TREATMENT | Assigned Treatment |
| RACE | Race |
| EVALITT | Included in Subjects Enrolled set? |
| EVALSAFE | Included in Safety set? |
| EVALQOL | Included in Quality of Life set? |

Table A2: “ECOG” dataset variables, Study 20020408

| Variable name | Label |
| --- | --- |
| SUBJID | Subject ID |
| VISIT | Visit Name |
| VISITDY | Study Day Relative to Enrollment |
| ECOG | ECOG Performance Status |
| PHASE | Phase |

Table A3: “Best Overall Response” dataset variables, Study 20020408

| Variable name | Label |
| --- | --- |
| SUBJID | Subject ID |
| VISIT | Visit Name |
| Rsresp | Overall Response status |
| PHASE | Phase |

Table A4: “QOL-EQ5d” dataset variables, Study 20020408

| Variable name | Label |
| --- | --- |
| SUBJID | Subject ID |
| VISIT | Visit Name |
| ENRREFDY | Study Day Relative to Enrollment |
| PHASE | Phase |
| Qlscale | QOL scale or Item |
| Qlraw | Observed Score, Standardized if Needed |

Table A5: “QOL-EORTC” dataset variables, Study 20020408

| Variable name | Label |
| --- | --- |
| SUBJID | Subject ID |
| VISIT | Visit Name |
| ENRREFDY | Study Day Relative to Enrollment |
| PHASE | Phase |
| Qlscale | QOL scale or Item |
| Qlraw | Observed Score, Standardized if Needed |

Table A6: “Lesion” dataset variables, Study 20020408

| Variable name | Label |
| --- | --- |
| SUBJID | Subject ID |
| VISIT | Visit Name |
| LSREADER | Reader |
| LSCAT | Lesion Category (Target/Non-target) |
| LSSITE | Lesion Site |
| LSSLD | Sum of Long. Diam Within Lesion Type(mm) |
| LSSLDPCH | Percent Change From Baseline for SLD |
| PHASE | Phase |

Table A7: “Serious AE” dataset variables, Study 20020408

| Variable name | Label |
| --- | --- |
| SUBJID | Subject ID |
| AEPT | Preferred Term (MedDRA) |
| AESOC | System Organ Class (MedDRA) |
| AEREL | Related to Investigational Product? |
| AESER | Serious? |
| AESTDYI | Imputed Study Day of Start of Event |
| PHASE | Phase |

Table A8: “LAB” dataset variables, Study 20020408

| Variable name | Label |
| --- | --- |
| SUBJID | Subject ID |
| VISIT | Visit Name |
| LBTEST | Lab Test |
| LBSTRESN | Numeric Result in Analysis Std Units |
| LBSTUNIT | Analysis Std Units |
| ENRREFDY | Study Day Relative to Enrollment |
| PHASE | Phase |

Table A9: “Events” dataset variables, Study 20020408

| Variable name | Label |
| --- | --- |
| SUBJID | Subject ID |
| DTHDY | Death Day (if died before end study date for study 20020408) |
| DTHDYX | Death Day (if died before end study date for study 20020408, during 20020408 long-term follow-up, or during 20030194) |
| DTH | Death (if died before end study date for study 20020408) |
| DTHX | Death (if died before end study date for study 20020408, during 20020408 long-term follow-up, or during 20030194) |
| PFSDYCR | PFS Day (Central, RECIST) |
| PFSCR | PD on Study (Central, RECIST) or Death |
| PDDYCR | PD Day (Central, RECIST) |
| PDCR | PD (Central, RECIST) on Study |
| PFSDYLR | PFS Day (Invest, RECIST) |
| PFSLR | PD on Study (Invest, RECIST) or Death |
| PDDYLR | PD Day (Invest, RECIST) |
| PDLR | PD (Invest, RECIST) on Study |

Table A10: “Treatment Disposition” dataset variables, Study 20020408

| Variable name | Label |
| --- | --- |
| SUBJID | Subject ID |
| TREATMENT | Assigned Treatment |
| FDOSDY | Study Day of First Dose Relat to Enrlmnt |
| LDOSENDY | Study Day of Last Dose Relat to Enrlmnt |
| EOIP | Reason for Ending Inv Prod |

Table A11: “Additional therapy” dataset variables, Study 20020408

| Variable name | Label |
| --- | --- |
| SUBJID | Subject ID |
| ADSTDYI | Imputed Study Day of Therapy |
| ADENDYI | Imputed Study Day of End of Therapy |
| PHASE | Phase |
| ADTHRPY | Additional Therapy |
| ADCONT | Continuing? |
| ADCAT | Additional Therapy Categorization |

Table A12: “FUP” dataset variables, Study 20020408

| Variable name | Label |
| --- | --- |
| SUBJID | Subject ID |
| FUSTAT | Long-term FUP Status |
| FULASTDT | Last Contact or Death Date |
| FUPDDT | Date of Disease Progression |

In addition, datasets and variables for study 20030194 were provided (Tables A13-A18). Data were collected based upon local investigator assessments until disease progression. Study 20030194 was an extension of study 20020408 in which patients entered if they experienced disease progression and received panitumumab. Out of the 232 subjects randomised to BSC in study 20020408, 177 were found eligible for study 20030194 and 176 were administered panitumumab.

Table A13: “Disposit” dataset variables, Study 20030194

| Variable name | Label |
| --- | --- |
| SUBJID | Subject ID |
| COUNTRY | Country |
| REGION | Region |
| FDOSDY | Study day of first dose rel to enrlmnt |
| LDOSENDY | Study day of last dose rel to enrlmnt |
| ENRLDY | Study day of enrlment rel to 408 |
| DTHDY | Death Day (if died before end study date for study 20020408) |
| DTHDYX | Death Day (if died before end study date for study 20020408, during 20020408 long-term follow-up, or during 20030194) |
| DTH | Death (if died before end study date for study 20020408) |
| DTHX | Death (if died before end study date for study 20020408, during 20020408 long-term follow-up, or during 20030194) |
| LASTOSDY | Last day on study |

Table A14: “ECOG” dataset variables, Study 20030194

| Variable name | Label |
| --- | --- |
| SUBJID | Subject ID |
| VISITDY | Visit day relative to enrollment 194 |
| VISIT | Visit Name |
| PHASE | Phase |
| ECOG | ECOG Performance Status |

Table A15: “Response” dataset variables, Study 20030194

| Variable name | Label |
| --- | --- |
| SUBJID | Subject ID |
| VISITDY | Visit day relative to enrollment 194 |
| VISIT | Visit Name |
| PHASE | Phase |
| RSRESP | Overall Response Status |

Table A16: “Lesion” dataset variables, Study 20030194

| Variable name | Label |
| --- | --- |
| SUBJID | Subject ID |
| LSDY | Assessment day rel to enrollment 194 |
| VISIT | Visit Name |
| PHASE | Phase |
| LSREADER | Reader |
| LSCAT | Lesion Category (Target/Non-target) |
| LSSITE | Lesion Site |
| LSNEW | New Lesion? |
| LSSLD | Sum of Long. Diam Within Lesion Type(mm) |
| LSSLDPCH | Percent Change From Baseline for SLD |
| LSSLDPCN | Percent Change From Nadir for SLD |
| LSRESP | Lesion Response |

Table A17: “AE” dataset variables, Study 20030194

| Variable name | Label |
| --- | --- |
| SUBJID | Subject ID |
| PHASE | Phase |
| AESTDYI | Imputed Study Day of Start of Event |
| AEPT | Preferred Term (MedDRA) |
| AESOC | System Organ Class (MedDRA) |
| AESER | Serious? |
| AEREL | Related to Investigational Product? |
| AESEV | Grade/Severity |

Table A18: “LAB” dataset variables, Study 20030194

| Variable name | Label |
| --- | --- |
| SUBJID | Subject ID |
| VISIT | Visit Name |
| PHASE | Phase |
| LBTEST | Lab Test |
| LBSTRESN | Numeric Result in Analysis Std Units |
| LBSTUNIT | Analysis Standard Units |
| ENRREFDY | Study Day Relative to Enrollment |

Covariates to include in the switching models were determined through discussion with a clinical expert [AD], because our aim was not to identify variables that improve the predictive ability of our switching models, but to identify confounding variables – those that are causes of switching and survival. The clinical expert was taken through the data available, and the concept of directed acyclic graphs was introduced using simple examples. Variable selection was then based on an assessment of which variables were likely to be common causes of switching and survival. Two model specifications were tested – an ‘inclusive’ model, including all variables considered potentially important, and a ‘reduced’ model, including only those variables considered to be the most important. The inclusive models included variables for age, ECOG, region, primary tumour diagnosis, EQ-5D score, time of progression, best tumour response category, lesion size, serious adverse events, and laboratory values for bilirubin, aspartate transaminase (AST), creatinine, albumin, lactate dehydrogenase, carcinoembryonic antigen, alanine amino transferase, and alkaline phosphatase. Reduced models included variables for ECOG, region, primary tumour diagnosis, EQ-5D score, time of progression, best tumour response category, and lesion size.

We created additional variables to indicate whether data for each variable was missing. Missing data was not imputed, because in order to be a confounder a variable must be observable to decision makers (the clinician and/or patient). Daily intervals were created for each patient, with values for variables updated each time an observation was recorded. Time was included using restricted cubic splines.

Given the knowledge that only patients with WT KRAS benefit from panitumumab, only switchers with WT KRAS were censored at the time of switch: MT KRAS switchers were not censored. Therefore, for our primary IPCW analyses, models for the probability of treatment switching (for both the numerator and the denominator of the weight) were only applied to patients with WT KRAS. In addition, these models were only fitted to patients in the BSC group, because it is unnecessary to make adjustments to the group randomised to panitumumab. Models for the numerator were fitted to all time intervals for BSC patients with WT KRAS, but the model for the denominator was only fitted to time intervals after the point of investigator-assessed disease progression – taking into account time-dependent information and acknowledging the fact that the probability of switching before investigator-assessed progression was zero. Pre-progression intervals were assigned a value of 1 for the denominator of the weight. Patients randomised to panitumumab, and patients with MT KRAS, were assigned weights of 1. In addition, we conducted secondary analyses which included BSC WT KRAS *and* BSC MT KRAS patients in the models for the probability of treatment switching. This substantially increases the sample size for the weighting models, but requires the additional assumption that KRAS status is not predictive of survival other than through treatment with panitumumab.

We used a Cox proportional hazards model to estimate the HR adjusted for treatment switching estimated using the IPCW weights. We present results for analyses with stabilised and unstabilised weights, and with inclusive and reduced models, for Estimands 1 and 2. For Estimand 2, for our primary IPCW analyses, patients with MT KRAS in both randomised groups were excluded. For our secondary IPCW analyses, patients with MT KRAS in the group randomised to panitumumab were excluded, but MT KRAS patients in the BSC group were retained. We stabilised weights by using as the numerator of the weight the probability of remaining uncensored over time estimated by a model that includes baseline ECOG performance status and region.

***Two-Stage Estimation***

The two-stage estimation (TSE) method involves first estimating the treatment effect associated with switching, by comparing control group switching patients with control group non-switching patients after a disease related secondary baseline. It is assumed that the treatment has a multiplicative effect on survival times (the treatment effect is a “time ratio”), which is then used to derive counterfactual survival times for switchers.^5,7^ Once this has been done, observed experimental group survival times are compared to a combination of observed control group survival times (for non-switchers) and counterfactual control group survival times (for switchers), to estimate a treatment effect adjusted for switching. The simple version of the TSE method (denoted TSEsimp) uses simple regression to estimate this treatment effect,^5^ whereas a more complex version uses g-estimation (denoted TSEgest).^7^

While time-dependent covariates can be included in simple regression models, this is not sufficient to control for variables that are time-dependent confounders; including time-dependent covariates risks obscuring part of the treatment effect, whilst excluding these variables risks selection bias.^7^ Therefore, in our TSEsimp analyses, only values of variables measured at the secondary baseline (or before) are included in the model. TSEsimp therefore assumes that there is no unmeasured confounding at the secondary baseline time-point, and that no confounding occurs between the secondary baseline time-point and the time of switch. The TSEgest approach involves a model for switching, comparing switchers to non-switchers, which is used to relate treatment at each measurement observation to counterfactual survival time given a specific value for the treatment effect, controlling for all modelled confounders – the g-estimation procedure tests a range of values for the treatment effect, searching for one that results in independence between switch status and counterfactual (untreated) survival times (that is, switching is independent of survival, conditional on the variables included in the model). TSEgest therefore assumes that there is no unmeasured confounding, but does not need to assume that no confounding occurs between the secondary baseline time-point and the time of switch, provided that confounding can be adjusted for using data collected between those two time-points. TSEgest therefore only offers advantages over TSEsimp if switching is lagged after the chosen secondary baseline time-point, and if useful data are collected during this lag. Research has shown that both TSEsimp and TSEgest result in low bias when adjusting for treatment switching provided their assumptions hold, but TSEsimp is prone to substantial bias if prognostic changes occur between the secondary baseline time-point and the time of switch.^5-8^

Given that treatment switching was only permitted after investigator assessed disease progression in study 20020408, we used this time-point as the secondary baseline for our TSE analyses. The same ‘inclusive’ and ‘reduced’ model specifications described for IPCW were used, although the variables included in TSEsimp were not time-updated – values measured at the baseline of the study and at disease progression were used. Missing data was dealt with in the same way as described for IPCW. For TSEsimp an accelerated failure time (AFT) model is used to estimate the treatment effect in switchers. We tested Weibull, log-logistic, log normal and Generalised Gamma models. For TSEgest, a test is required to determine whether or not untreated survival times are unrelated to switch status. We used a Wald test with a sandwich variance to account for the dependence of observations within subjects. A search procedure must be adopted for the g-estimation process – we ran analyses using interval bisection and a grid search. Estimating counterfactual survival times using TSE can result in informative censoring, because for patients who switch treatments who do not die, adjusted censoring times are estimated. Estimating new censoring times for switchers but not for non-switchers can induce informative censoring, and therefore re-censoring may be useful.^9-13^ We conducted each of our TSE analyses both with and without re-censoring.

For consistency with our IPCW analyses, in our primary TSE analyses we compared BSC WT KRAS switchers to BSC WT KRAS non-switchers to estimate the effect of switching. However, TSEsimp does not involve a model for the probability of switching: the model is used simply to estimate the effect of switching. For this reason, and because we assume that MT KRAS patients do not benefit from panitumumab, it is reasonable to conduct a secondary TSEsimp analysis in which BSC WT KRAS switchers are compared to BSC WT KRAS non-switchers *and* BSC MT KRAS patients (irrespective of switch status) in order to estimate the effect of switching. This substantially increases the sample size for the model used to estimate the effect of switching, but requires the additional assumption that KRAS status is not predictive of survival other than through treatment with panitumumab. TSEgest does involve a model for switching, but rather than explicitly modelling the probability of switching according to baseline and time-dependent characteristics, the objective is to identify a value for the treatment effect that is independent of counterfactual survival times, conditional on the variables included in the model. For this reason, it is reasonable to also conduct secondary analyses for TSEgest, whereby BSC MT KRAS patients are retained in the model that estimates the treatment effect in switchers as described for TSEsimp.

For our primary TSE analyses, for Estimand 2, patients with MT KRAS in both randomised groups were simply excluded from the analyses. For our secondary TSE analyses, patients with MT KRAS in the group randomised to panitumumab were excluded for Estimand 2, but MT KRAS patients in the BSC group were not excluded: they were used in the estimation of the treatment effect associated with switching and retained for the estimation of the treatment effect of panitumumab adjusted for switching. Again, these secondary TSE analyses rely on the additional assumption that KRAS status is not predictive of survival other than through treatment with panitumumab.

Hence, for TSE we used the TSEsimp and TSEgest approaches to conduct primary and secondary analyses with inclusive and reduced models, for a range of AFT models (for TSEsimp), with interval bisection and a grid search (for TSEgest), with and without re-censoring, for estimands 1 and 2. TSE was used to adjust for switching, and then Cox proportional hazards models were used to estimate the HR associated with being randomised to panitumumab, adjusted for treatment switching.

***Rank Preserving Structural Failure Time Model***

Like the TSE method, the rank preserving structural failure time model (RPSFTM) method estimates counterfactual survival times – survival times that would have been observed if specified treatment switches had not occurred.^14^ Also like the TSE method, the RPSFTM uses an accelerated failure time model framework and assumes that treatment has a multiplicative effect on survival times. However, the important difference between the RPSFTM and TSE methods is that RPSFTM does not require the no unmeasured confounding assumption. Instead, RPSFTM relies upon assuming that there is a “common treatment effect” – that is, the time ratio associated with treatment is the same in switchers as it is in the group of patients originally randomised to the experimental group. It is also assumed that if no patients received treatment, survival times would have been equal, on average, between randomised groups – that is, randomisation has “worked”.

The method involves splitting the survival time for each patient into time spent “on” treatment, and time spent “off” treatment. G-estimation is used to identify the treatment effect that, when taking into account the time spent on and off treatment by each individual patient, would result in equal untreated survival times between randomised groups. This provides survival times for switchers that would have been observed if switching hadn’t taken place, and observed experimental group survival times can then be compared to a combination of observed control group survival times (for non-switchers) and counterfactual survival times (for switchers) in order to estimate the treatment effect adjusted for switching. Research has shown that the RPSFTM results in very low bias when adjusting for treatment switching, providing the treatment effect received by switching patients is similar to that received by patients initially randomised to the experimental treatment.^5-8^ However, significant bias can result if the treatment effect achieved by switching patients is very different to that achieved by patients initially randomised to the experimental treatment.

It is clear that in the context of Study 20020408, the RPSFTM method is problematic, due to its assumption of a common treatment effect. In reality, we know that there was substantial treatment effect heterogeneity, because WT KRAS patients benefit from panitumumab, whereas MT KRAS patients do not. We addressed this by specifying the RPSFTM such that MT KRAS patients received zero treatment effect whilst “on” treatment.

The RPSFTM can be applied on an “ever treated” basis, or on an “as treated” basis (also referred to as “treatment group” and “on treatment” analyses).^9^ On an “ever treated” basis, the “on” treatment indicator is set to “1” for all time-points beyond receiving experimental treatment, ignoring subsequent treatment discontinuation. On an “as treated” basis, the “on” treatment indicator is set to “1” only whilst experimental treatment is actually being received – it reverts back to “0” after treatment discontinuation. Typically the “as treated” analysis provides a larger estimate of the treatment effect time ratio, because the time ratio is attributed to a shorter time period (i.e., only the time actually spent on treatment, rather than all time after exposure to the treatment). However, the two approaches are likely to result in similar estimates of counterfactual survival times because the “as treated” analysis attributes a larger treatment effect to a shorter time period, and the “ever treated” analysis attributes a smaller treatment effect to a longer timer period. Strictly, the “ever treated” and “as treated” RPSFTM analyses make slightly different assumptions: the “as treated” analysis assumes that a treatment effect is only received whilst treatment is being received; the “ever treated” analysis assumes that the treatment effect may be retained beyond treatment discontinuation.

As for the TSE method, estimating counterfactual survival times using the RPSFTM can cause informative censoring problems, and therefore we ran all our RPSFTM analyses both with and without re-censoring. Within the RPSFTM model, g-estimation requires a g-test to determine whether or not untreated survival times between treatment groups are equal. Options include the logrank test, a Wilcoxon test, or a Wald test using a Cox model. However, only the Cox model can cope with covariates, and because our analyses must control for the factors included in previous analyses of Study 20020408 in order to be consistent, we conducted our analyses using the Cox model for the g-test. In addition, a search procedure must be adopted for the g-estimation process – we ran analyses using interval bisection and a grid search. Finally, the iterative parameter estimation (IPE) algorithm has also been used to adjust for treatment switching in RCTs.^15^ The IPE method uses the same counterfactual survival model as the RPSFTM, but uses a parametric modelling approach to determine the causal treatment effect instead of g-estimation. Treatment effect estimates from the two approaches are likely to be similar given that both use the same underlying model. This has been demonstrated in simulation studies.^5,6,8^ We re-ran each of our RPSFTM analyses using the IPE estimation procedure, using a Weibull parametric model.

Hence, we used the RPSFTM and IPE approaches to conduct analyses for estimands 1 and 2, with interval bisection and a grid search (for the RPSFTM), with and without re-censoring, and on an “as treated” and “ever treated” basis. The RPSFTM and IPE methods were used to adjust for switching, and then Cox proportional hazards models were used to estimate the HR associated with being randomised to panitumumab, adjusted for treatment switching.

**Appendix B: Patient Experience and Time-to-Switch Plots**Figure B1: Patient experience in Study 20020408

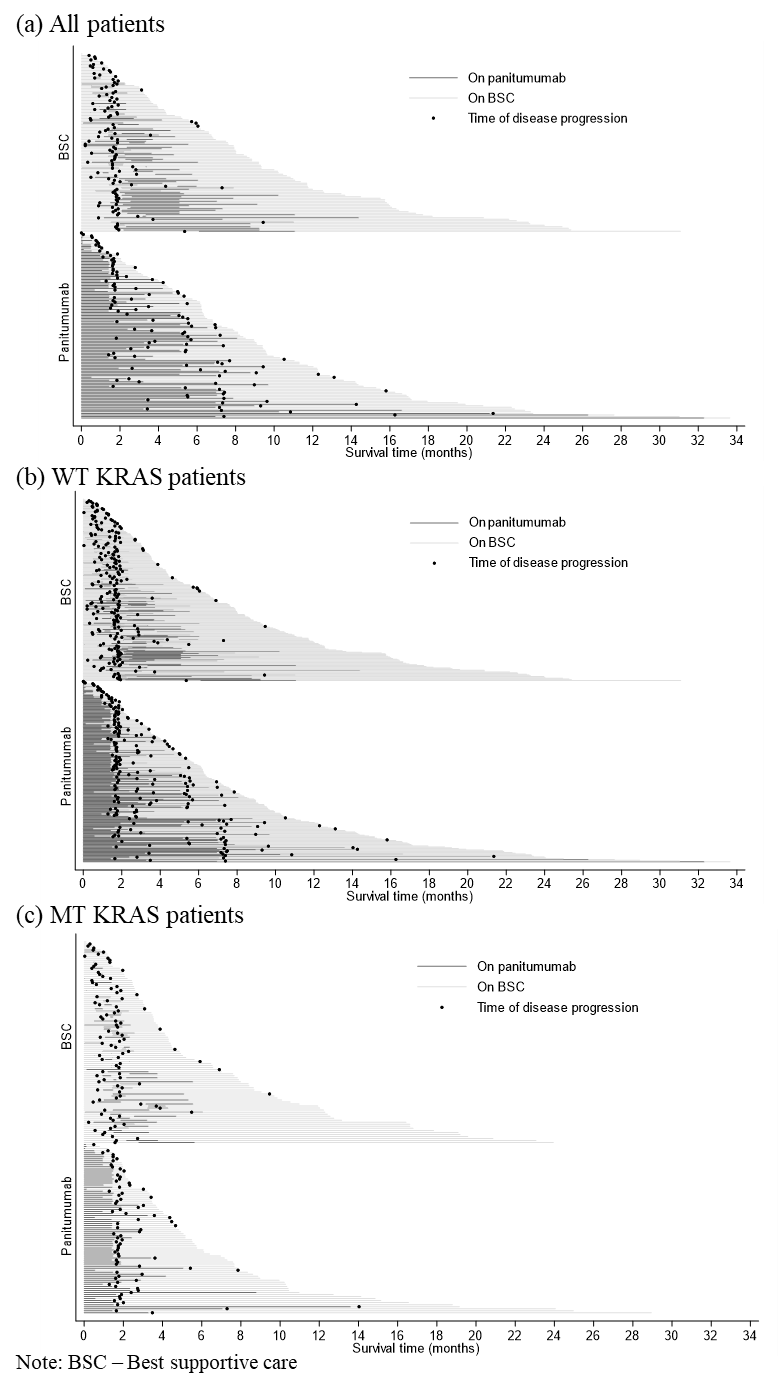


Figure B2: Time from progression to switch
(a) All patients

(b) By KRAS group

Note: KRAS – Kirsten Rat Sarcoma Virus; MT: Mutant type; WT: Wild type.

**Appendix C: Results From all Analyses**

Table C1: All analyses results, KRAS identifiable population

| Analysis | Estimand 1 (all patients) hazard ratio (95% CI) | Estimand 2 (KRAS WT) hazard ratio (95% CI) |
| --- | --- | --- |
| Expected “truth” | 0.77-0.78 (0.61 – 1.00) | 0.55-0.73 (0.41 – 0.93) |
| ITT | 0.97 (0.79 – 1.18) | 0.99 (0.76 – 1.30) |
| IPCW 1 (primary analysis, inclusive model, stabilised weights) | 0.75 (0.59 – 0.95) | 0.55 (0.28 – 1.09) |
| IPCW 2 (primary analysis, inclusive model, unstabilised weights) | 0.71 (0.54 – 0.93) | 0.46 (0.25 – 0.84) |
| IPCW 3 (primary analysis, reduced model, stabilised weights) | 0.75 (0.59 – 0.95) | 0.54 (0.29 – 0.99) |
| IPCW 4 (primary analysis, reduced model, unstabilised weights) | 0.72 (0.53 – 0.98) | 0.50 (0.28 – 0.87) |
| IPCW 5 (secondary analysis, inclusive model, stabilised weights) | 0.75 (0.59 – 0.96) | 0.65 (0.50 – 0.85) |
| IPCW 6 (secondary analysis, inclusive model, unstabilised weights) | 0.76 (0.60 – 0.97) | 0.66 (0.50 – 0.86) |
| IPCW 7 (secondary analysis, reduced model, stabilised weights) | 0.79 (0.63 – 1.00) | 0.69 (0.53 – 0.90) |
| IPCW 8 (secondary analysis, reduced model, unstabilised weights) | 0.80 (0.64 – 1.02) | 0.70 (0.54 – 0.91) |
| TSEsimp 1 (primary analysis, inclusive model, with re-censoring, Weibull model) | 0.64 (0.46 – 0.98) | 0.44 (0.24 – 0.95) |
| TSEsimp 2 (primary analysis, inclusive model, with re-censoring, log normal model) | 0.62 | 0.41 |
| TSEsimp 3 (primary analysis, inclusive model, without re-censoring, Weibull model) | 0.68 | 0.49 |
| TSEsimp 4 (primary analysis, inclusive model, without re-censoring, log normal model) | 0.67 | 0.47 |
| TSEsimp 5 (primary analysis, reduced model, with re-censoring, Weibull model) | 0.59 | 0.37 |
| TSEsimp 6 (primary analysis, reduced model, with re-censoring, Generalised Gamma model) | 0.60 | 0.38 |
| TSEsimp 7 (primary analysis, reduced model, without re-censoring, Weibull model) | 0.65 | 0.43 |
| TSEsimp 8 (primary analysis, reduced model, without re-censoring, Generalised Gamma model) | 0.66 | 0.44 |
| TSEsimp 9 (secondary analyses, inclusive model, with re-censoring, Weibull model) | 0.70 (0.54 – 0.90) | 0.59 (0.43 – 0.78) |
| TSEsimp 10 (secondary analyses, inclusive model, with re-censoring, log normal model) | 0.71 | 0.59 |
| TSEsimp 11 (secondary analyses, inclusive del, without re-censoring, Weibull model) | 0.74 | 0.63 |
| TSEsimp 12 (secondary analyses, inclusive model, without re-censoring, log normal model) | 0.74 | 0.63 |
| TSEsimp 13 (secondary analyses, reduced model, with re-censoring, Weibull model) | 0.76 (0.58 – 0.95) | 0.63 (0.46 – 0.83) |
| TSEsimp 14 (secondary analyses, reduced model, with re-censoring, Generalised Gamma model) | 0.74 | 0.62 |
| TSEsimp 15 (secondary analyses, reduced model, without re-censoring, Weibull model) | 0.78 | 0.66 |
| TSEsimp 16 (secondary analyses, reduced model, without re-censoring, Generalised Gamma model) | 0.77 | 0.66 |
| TSEgest 1 (primary analysis, inclusive model, with re-censoring, interval bisection) | 0.54 (0.30 – 1.51) | 0.31 (0.13 – 2.12) |
| TSEgest 2 (primary analysis, inclusive model, with re-censoring, grid search) | 0.54 | 0.31 |
| TSEgest 3 (primary analysis, inclusive model, without re-censoring, interval bisection) | 0.61 | 0.36 |
| TSEgest 4 (primary analysis, inclusive model, without re-censoring, grid search) | 0.61 | 0.36 |
| TSEgest 5 (primary analysis, reduced model, with re-censoring, interval bisection) | 0.59 | 0.37 |
| TSEgest 6 (primary analysis, reduced model, with re-censoring, grid search) | 0.60 | 0.37 |
| TSEgest 7 (primary analysis, reduced model, without re-censoring, interval bisection) | 0.64 | 0.42 |
| TSEgest 8 (primary analysis, reduced model, without re-censoring, grid search) | 0.64 | 0.42 |
| TSEgest 9 (secondary analyses, inclusive model, with re-censoring, interval bisection) | 0.79 (0.57 – 0.99) | 0.67 (0.46 – 0.86) |
| TSEgest 10 (secondary analyses, inclusive model, with re-censoring, grid search) | 0.79 | 0.67 |
| TSEgest 11 (secondary analyses, inclusive model, without re-censoring, interval bisection) | 0.82 | 0.71 |
| TSEgest 12 (secondary analyses, inclusive model, without re-censoring, grid search) | 0.82 | 0.71 |
| TSEgest 13 (secondary analyses, reduced model, with re-censoring, interval bisection) | 0.84 | 0.72 |
| TSEgest 14 (secondary analyses, reduced model, with re-censoring, grid search) | 0.84 | 0.72 |
| TSEgest 15 (secondary analyses, reduced model, without re-censoring, interval bisection) | 0.87 | 0.74 |
| TSEgest 16 (secondary analyses, reduced model, without re-censoring, grid search) | 0.87 | 0.74 |
| RPSFTM 1 (with re-censoring, ever treated, interval bisection) | 0.91 (0.49 – 1.68) | 0.87 (0.02 – 35.88) |
| RPSFTM 2 (with re-censoring, ever treated, grid search) | 0.90 (0.48 – 1.70) | 0.87 (0.02 – 38.80) |
| RPSFTM 3 (without re-censoring, ever treated, interval bisection) | 0.91 (0.52 – 1.61) | 0.89 (0.03 – 23.18) |
| RPSFTM 4 (without re-censoring, ever treated, grid search) | 0.91 (0.52 – 1.61) | 0.89 (0.03 – 23.18) |
| RPSFTM 5 (with re-censoring, as treated, interval bisection) | 0.93 (0.59 – 1.46) | 0.92 (0.11 – 7.85) |
| RPSFTM 6 (with re-censoring, as treated, grid search) | 0.93 (0.59 – 1.47) | 0.92 (0.10 – 8.21) |
| RPSFTM 7 (without re-censoring, as treated, interval bisection) | 0.91 (0.50 – 1.64) | 0.89 (0.04 – 21.34) |
| RPSFTM 8 (without re-censoring, as treated, grid search) | 0.91 (0.50 – 1.64) | 0.89 (0.04 – 21.52) |
| IPE 1 (with re-censoring, ever treated, Weibull model) | 0.94 (0.64 – 1.38) | 0.94 (0.17 – 5.27) |
| IPE 2 (without re-censoring, ever treated, Weibull model) | 0.94 (0.66 – 1.35) | 0.94 (0.19 – 4.75) |
| IPE 3 (with re-censoring, as treated, Weibull model) | 0.95 (0.72 – 1.27) | 0.96 (0.36 – 2.60) |
| IPE 4 (without re-censoring, as treated, Weibull model) | 0.96 (0.73 – 1.26) | 0.97 (0.39 – 2.37) |

Notes: KRAS - Kirsten Rat Sarcoma Virus; CI - Confidence Interval; WT - Wild Type; ITT - Intention to treat; IPCW - Inverse Probability of Censoring Weights; TSEsimp - Two-stage estimation with simple regression; TSEgest - Two-stage estimation with g-estimation; RPSFTM - Rank preserving structural failure time model. Confidence intervals only calculated for TSE analyses mentioned in the main paper. These were calculated by bootstrapping the entire adjustment analyses, with 5,000 samples. For TSEgest 1 the g-estimation process did not converge in 17% of samples. For TSEgest 9 the g-estimation process did not converge in 4% of samples.

**Appendix References**

[1] Van Cutsem E, Peeters M, Siena S, Humbles Y, Hendisz A, Neyns B et al. Open-Label Phase III Trial of Panitumumab Plus Best Supportive Care Compared With Best Supportive Care Alone in Patients With Chemotherapy-Refractory Metastatic Colorectal Cancer. Journal of Clinical Oncology 2007;25;13:1658-1664.

[2] Amado RG, Wolf M, Peeters M, Van Cutsem E, Siena S, Freeman DJ et al. Wild-Type KRAS Is Required for Panitumumab Efficacy in Patients With Metastatic Colorectal Cancer. Journal of Clinical Oncology 2008;26;10:1626-1634.

[3] Poulin-Costello M, Azoulay L, Van Cutsem E, Peeters M, Siena S and Wolf M. An analysis of the treatment effect of panitumumab on overall survival from a phase 3, randomized, controlled, multicenter trial (20020408) in patients with chemotherapy refractory metastatic colorectal cancer. Targeted Oncology 2013;8:127-136.

[4] Hernan MA, Brumback B, Robins JM. Marginal structural models to estimate the joint causal effect of nonrandomized treatments. Journal of the American Statistical Association. 2001;96(454):440–8.

[5] Latimer NR, Abrams KR, Lambert PC, Crowther MJ, Wailoo AJ, Morden JP, Akehurst RL, Campbell MJ. Adjusting for treatment switching in randomised controlled trials – A simulation study and a simplified two-stage method. Statistical Methods in Medical Research, 21 Nov 2014.

[6] Latimer, Nicholas R; Abrams, Keith R; Lambert, Paul C; et al. Assessing methods for dealing with treatment switching in clinical trials: A follow-up simulation study. Statistical methods in medical research  25 Apr 2016

[7] Latimer NR, White IR, Tilling K, Siebert U. Improved two-stage estimation to adjust for treatment switching in randomised trials: g-estimation to address time-dependent confounding. Statistical Methods in Medical Research, 30 Mar 2020.

[8] Morden JP, Lambert PC, Latimer N, Abrams KR, Wailoo AJ. Assessing statistical methods for dealing with treatment switching in randomised controlled trials: A simulation study. BMC Methodology 2011;11(4).

[9] Latimer NR, Abrams K, Lambert PC, Crowther MJ, Wailoo AJ, Morden JP, Akehurst RL, Campbell MJ. Adjusting survival time estimates to account for treatment switching in randomised controlled trials – an economic evaluation context: Methods, limitations and recommendations. Medical Decision Making 2014;34;3:387-402.

[10] Latimer NR, White IR, Abrams KR, Siebert U. Causal inference for long-term survival in randomised trials with treatment switching: Should re-censoring be applied when estimating counterfactual survival times? Statistical Methods in Medical Research 28(8):2475-2493 01 Aug 2019.

[11] White IR. Estimating treatment effects in randomized trials with treatment switching. Stat Med 2006; 25: 1619–1622.

[12] Robins JM. The analysis of randomized and non-randomized AIDS treatment trials using a new approach to causal inference in longitudinal studies. In: Sechrest L, Freeman H and Mulley A (eds) Health service research methodology: a focus on AIDS. Washington, DC: U.S. Public Health Service, National Center for Health Services Research, 1989, pp.113–159.

[13] Robins JM. Analytic methods for estimating HIV treatment and cofactor effects. In: Ostrow DG and Kessler R (eds) Methodological issues of AIDS mental health research. New York, NY: Plenum Publishing, 1993, pp.213–290.

[14] Robins, J.M., Tsiatis, A.A. Correcting for non-compliance in randomized trials using rank preserving structural failure time models. Communications in Statistics-Theory and Methods 1991; 20(8):2609-2631.

[15] Branson M and Whitehead J. Estimating a treatment effect in survival studies in which patients switch treatment. Statistics in Medicine 2002; 21: 2449–2463.

**Appendix D: Full Coding and Results for all Analyses**

Contents

Page

IPCW Analyses…………………………………………………………….. 24

TSEsimp Analyses…………………………………………………………. 71

TSEgest Analyses…………………………………………………………...190

RPSFTM Analyses………………………………………………………….327

IPE Analyses……………………………………………………………….. 384

Z Graphs and Kaplan-Meier Graphs from RPSFTM and IPE Analyses…... 413

**IPCW Analyses**

-------------------------------------------------------------------------------

name: <unnamed>

log: X:\ScHARR\Users\cm1nrl\Case studies\Amgen\Code\Final IPCW analyses

> for paper.smcl

log type: smcl

opened on: 26 Oct 2022, 17:50:25

. do "C:\Users\cm1nrl\AppData\Local\Temp\STD4384_000000.tmp"

. *** PANI ANALYSES FOR PAPER: ESTIMAND 1***

.

. *****************************************************

. ******************** ITT *************************

. *****************************************************

.

. use "X:\ScHARR\Users\cm1nrl\Case studies\Amgen\Data\Final merged dataset\base

> line_master", clear

(TREAT)

.

. ********************** estimand 1

. drop if krasi==.

(36 observations deleted)

. stset DTHDYX, id(SUBJID) failure (DTHX) scale(30.4375)

Survival-time data settings

ID variable: SUBJID

Failure event: DTHX!=0 & DTHX<.

Observed time interval: (DTHDYX[_n-1], DTHDYX]

Exit on or before: failure

Time for analysis: time/30.4375

--------------------------------------------------------------------------

427 total observations

0 exclusions

--------------------------------------------------------------------------

427 observations remaining, representing

427 subjects

391 failures in single-failure-per-subject data

3,345.101 total analysis time at risk and under observation

At risk from t = 0

Earliest observed entry t = 0

Last observed exit t = 33.64271

. stcox trtgrp regionstrat becogstrat

Failure _d: DTHX

Analysis time _t: DTHDYX/30.4375

ID variable: SUBJID

Iteration 0: log likelihood = -2020.2411

Iteration 1: log likelihood = -2010.7205

Iteration 2: log likelihood = -1995.5505

Iteration 3: log likelihood = -1995.196

Iteration 4: log likelihood = -1995.1956

Refining estimates:

Iteration 0: log likelihood = -1995.1956

Cox regression with Breslow method for ties

No. of subjects = 427 Number of obs = 427

No. of failures = 391

Time at risk = 3,345.1006

LR chi2(3) = 50.09

Log likelihood = -1995.1956 Prob > chi2 = 0.0000

------------------------------------------------------------------------------

_t | Haz. ratio Std. err. z P>|z| [95% conf. interval]

-------------+----------------------------------------------------------------

trtgrp | .9685531 .0983198 -0.31 0.753 .7938094 1.181764

regionstrat | .9987459 .0690249 -0.02 0.986 .8722221 1.143623

becogstrat | 3.229122 .4733157 8.00 0.000 2.422801 4.303792

------------------------------------------------------------------------------

.

. *****************************************************************************

> ********************

. *** IPCW 1 (primary analysis, full model, stabilised weights) Estimand 1 [113

> from IPCW file] ***

. *****************************************************************************

> ********************

.

. use "X:\ScHARR\Users\cm1nrl\Case studies\Amgen\Data\Interim merged datasets\t

> dc_dataset34.dta", clear

(TREAT)

.

. preserve

.

. * note, 8 patients switched before investigator observed progression. 7 were

> kras MT, so primary analyses will not adjust for these anyway.

. * protocol suggested switching permitted only after progression. So assume th

> ese patients switched due to some signs of progression.

. * need this, because IPCW models will fit much better if applied only to the

> time-periods where switching was "permitted".

. * so, for these 8 patients, replace PDDYLR to equal xotime

. * and replace progtdc to = 1 after this point for these patients

. sort SUBJID dthdyxtdc

. by SUBJID: replace PDDYLR = xotime if (PDDYLR>xotime & xotime!=.)

(1,252 real changes made)

. by SUBJID: replace progtdc = 1 if (PDDYLR <= dthdyxtdc & PDLR==1)

(984 real changes made)

.

. replace xotdc = 0 if krasi==1 & trtgrp==1

(14,415 real changes made)

. replace xo = . if krasi==1 & trtgrp==1 & xo==1

(18,500 real changes made, 18,500 to missing)

.

. by SUBJID: drop if (dthdyxtdc>xotime & xo==1)

(22,711 observations deleted)

. by SUBJID: replace lastobs = 0

(336 real changes made)

. by SUBJID: replace lastobs = 1 if _n==_N

(427 real changes made)

.

. *** IPCW step 1 with splines

.

. *** Use logistic regression to predict switching given baseline covariates (m

> odel S1)***

. *** First use rcsgen to create splines for a time-dependent interecept***

. *** Generate 6 knots based upon the event time distribution (can try other am

> ounts of knots)***

. rcsgen dthdyxtdc, df(5) if2(xotdc==1) gen(timexosp)

Variables timexosp1 to timexosp5 were created

. * where are the knots?

. di r(knots)

3 43 60 68 78 335

. logistic xotdc becogstrat regionstrat timexosp* if trtgrp==1 & krasi==2

Logistic regression Number of obs = 9,012

LR chi2(7) = 90.79

Prob > chi2 = 0.0000

Log likelihood = -463.33157 Pseudo R2 = 0.0892

------------------------------------------------------------------------------

xotdc | Odds ratio Std. err. z P>|z| [95% conf. interval]

-------------+----------------------------------------------------------------

becogstrat | 1.413203 .5717528 0.85 0.393 .6394783 3.12308

regionstrat | .971615 .1596466 -0.18 0.861 .7040975 1.340774

timexosp1 | 1.006221 .0293224 0.21 0.831 .9503603 1.065365

timexosp2 | .9999761 .0000891 -0.27 0.788 .9998014 1.000151

timexosp3 | .9996842 .0003131 -1.01 0.313 .9990706 1.000298

timexosp4 | 1.000578 .0003259 1.77 0.076 .9999396 1.001217

timexosp5 | .9997511 .0000914 -2.72 0.006 .999572 .9999302

_cons | .0027814 .0016502 -9.92 0.000 .0008695 .0088979

------------------------------------------------------------------------------

Note: _cons estimates baseline odds.

.

. *** assess fit of the model, for comparison to alternatives that may be run l

> ater***

. estat gof, group(10) table

note: obs collapsed on 10 quantiles of estimated probabilities.

Goodness-of-fit test after logistic model

Variable: xotdc

Table collapsed on quantiles of estimated probabilities

+--------------------------------------------------------+

| Group | Prob | Obs_1 | Exp_1 | Obs_0 | Exp_0 | Total |

|-------+--------+-------+-------+-------+-------+-------|

| 1 | 0.0027 | 2 | 1.6 | 905 | 905.4 | 907 |

| 2 | 0.0029 | 3 | 2.8 | 961 | 961.2 | 964 |

| 3 | 0.0032 | 4 | 2.7 | 888 | 889.3 | 892 |

| 4 | 0.0038 | 2 | 2.9 | 842 | 841.1 | 844 |

| 5 | 0.0045 | 4 | 4.0 | 963 | 963.0 | 967 |

|-------+--------+-------+-------+-------+-------+-------|

| 6 | 0.0060 | 5 | 4.3 | 831 | 831.7 | 836 |

| 7 | 0.0091 | 4 | 6.7 | 903 | 900.3 | 907 |

| 8 | 0.0163 | 10 | 11.9 | 939 | 937.1 | 949 |

| 9 | 0.0293 | 20 | 19.1 | 833 | 833.9 | 853 |

| 10 | 0.0623 | 37 | 35.0 | 856 | 858.0 | 893 |

+--------------------------------------------------------+

Number of observations = 9,012

Number of groups = 10

Hosmer–Lemeshow chi2(8) = 2.66

Prob > chi2 = 0.9539

. estat ic

Akaike's information criterion and Bayesian information criterion

-----------------------------------------------------------------------------

Model | N ll(null) ll(model) df AIC BIC

-------------+---------------------------------------------------------------

. | 9,012 -508.7252 -463.3316 8 942.6631 999.5136

-----------------------------------------------------------------------------

Note: BIC uses N = number of observations. See [R] BIC note.

.

. *** Estimate the probability of switching for each patient-observation includ

> ed in the regression.***

. predict pxo1 if e(sample), pr

(70,094 missing values generated)

.

. *** Use logistic regression to predict switching given baseline and time-upda

> ted covariates (model S12)***

. logistic xotdc AGE becogstrat diagtype BILIULN ASTULN CREATULN ALBULN LDHULN

> eq5dbase CEAULN eq5dmissb regionstrat PDDYLR eq5dtdc eq5dind eq5dmisslastvisi

> t ecogtdc bestresptdc respmisslastvisit LSSLDtdc LSSLDmisslastvisit AATULNtdc

> AATmisslastvisit ALBULNtdc ALBmisslastvisit ALKULNtdc ALKmisslastvisit ASTUL

> Ntdc ASTmisslastvisit CEAULNtdc CEAmisslastvisit CREATmisslastvisit LDHULNtdc

> LDHmisslastvisit BILIULNtdc BILImisslastvisit timexosp* if trtgrp==1 & krasi

> ==2 & progtdc>0

note: ALBmisslastvisit omitted because of collinearity.

note: ALKmisslastvisit omitted because of collinearity.

note: ASTmisslastvisit omitted because of collinearity.

note: CREATmisslastvisit omitted because of collinearity.

note: LDHmisslastvisit omitted because of collinearity.

note: BILImisslastvisit omitted because of collinearity.

Logistic regression Number of obs = 2,222

LR chi2(35) = 126.54

Prob > chi2 = 0.0000

Log likelihood = -316.61291 Pseudo R2 = 0.1666

-------------------------------------------------------------------------------

xotdc | Odds ratio Std. err. z P>|z| [95% conf. interval]

--------------+----------------------------------------------------------------

AGE | .9868856 .0131012 -0.99 0.320 .9615389 1.0129

becogstrat | .999556 .5734768 -0.00 0.999 .3246729 3.077289

diagtype | 1.383078 .4672014 0.96 0.337 .7133661 2.681521

BILIULN | 1.94632 .9552079 1.36 0.175 .7438146 5.092885

ASTULN | .2896219 .1378194 -2.60 0.009 .1139669 .7360105

CREATULN | 1.3873 1.708745 0.27 0.790 .1240912 15.50958

ALBULN | .7466926 .3967427 -0.55 0.582 .2635543 2.115503

LDHULN | 1.227953 .5967152 0.42 0.673 .4737468 3.182857

eq5dbase | .4617082 .3089724 -1.15 0.248 .1243794 1.713905

CEAULN | .768319 .3119722 -0.65 0.516 .3466693 1.702816

eq5dmissb | .9074362 .7676654 -0.11 0.909 .1728716 4.763307

regionstrat | .7623227 .169055 -1.22 0.221 .4935985 1.177345

PDDYLR | .988393 .0085777 -1.35 0.179 .9717232 1.005349

eq5dtdc | 1.435989 .8668345 0.60 0.549 .439869 4.687905

eq5dind | .4700634 .4971443 -0.71 0.475 .0591447 3.735913

eq5dmisslas~t | 1.581828 .5785153 1.25 0.210 .7724186 3.239407

ecogtdc | .6539933 .1389212 -2.00 0.046 .4312817 .9917118

bestresptdc | .9332468 .2265922 -0.28 0.776 .5798624 1.501994

respmisslas~t | 1.309429 .4766581 0.74 0.459 .6415462 2.672614

LSSLDtdc | .9988725 .0014529 -0.78 0.438 .9960288 1.001724

LSSLDmissla~t | .8816743 .4632389 -0.24 0.811 .3148327 2.469087

AATULNtdc | 1.310922 .4082315 0.87 0.385 .7120427 2.413503

AATmisslast~t | .0997322 .0791923 -2.90 0.004 .0210348 .4728592

ALBULNtdc | 1.986922 .7458566 1.83 0.067 .9520379 4.146745

ALBmisslast~t | 1 (omitted)

ALKULNtdc | 1.291451 .5387566 0.61 0.540 .5701395 2.925331

ALKmisslast~t | 1 (omitted)

ASTULNtdc | 1.739563 .7541859 1.28 0.202 .7437084 4.068907

ASTmisslast~t | 1 (omitted)

CEAULNtdc | .6231413 .4464003 -0.66 0.509 .153042 2.537246

CEAmisslast~t | .1624489 .0770435 -3.83 0.000 .0641244 .411538

CREATmissla~t | 1 (omitted)

LDHULNtdc | 1.555114 .7330146 0.94 0.349 .6173649 3.91726

LDHmisslast~t | 1 (omitted)

BILIULNtdc | .7099261 .2756174 -0.88 0.378 .3317018 1.519422

BILImisslas~t | 1 (omitted)

timexosp1 | 1.032927 .0548102 0.61 0.542 .930899 1.146138

timexosp2 | 1.000156 .000121 1.29 0.196 .9999192 1.000394

timexosp3 | .9993284 .0003967 -1.69 0.091 .9985512 1.000106

timexosp4 | 1.000742 .0004101 1.81 0.070 .9999381 1.001546

timexosp5 | .9997845 .0001169 -1.84 0.065 .9995554 1.000014

_cons | .4379219 .9264943 -0.39 0.696 .006927 27.68539

-------------------------------------------------------------------------------

Note: _cons estimates baseline odds.

. * Note, had to take ecogmisslastvisit out as !=0 perfectly predicted failure.

> Also removed saetdc and CREATULDtdc as these had crazy coefficients and SEs.

. * And, ALBmisslastvisit, ALKmisslastvisit, ASTmisslastvisit, CREATmisslastvis

> it, LDHmisslastvisit, BILImisslastvisit all omitted because of collinearity

.

. *** Assess model fit

. estat gof, group(10) table

note: obs collapsed on 10 quantiles of estimated probabilities.

Goodness-of-fit test after logistic model

Variable: xotdc

Table collapsed on quantiles of estimated probabilities

+--------------------------------------------------------+

| Group | Prob | Obs_1 | Exp_1 | Obs_0 | Exp_0 | Total |

|-------+--------+-------+-------+-------+-------+-------|

| 1 | 0.0019 | 1 | 0.3 | 222 | 222.7 | 223 |

| 2 | 0.0030 | 0 | 0.6 | 222 | 221.4 | 222 |

| 3 | 0.0045 | 1 | 0.8 | 221 | 221.2 | 222 |

| 4 | 0.0079 | 1 | 1.4 | 221 | 220.6 | 222 |

| 5 | 0.0197 | 5 | 2.6 | 217 | 219.4 | 222 |

|-------+--------+-------+-------+-------+-------+-------|

| 6 | 0.0381 | 6 | 6.8 | 217 | 216.2 | 223 |

| 7 | 0.0544 | 10 | 10.2 | 212 | 211.8 | 222 |

| 8 | 0.0699 | 6 | 13.7 | 216 | 208.3 | 222 |

| 9 | 0.1049 | 23 | 18.2 | 199 | 203.8 | 222 |

| 10 | 0.3902 | 38 | 36.5 | 184 | 185.5 | 222 |

+--------------------------------------------------------+

Number of observations = 2,222

Number of groups = 10

Hosmer–Lemeshow chi2(8) = 10.69

Prob > chi2 = 0.2197

. estat ic

Akaike's information criterion and Bayesian information criterion

-----------------------------------------------------------------------------

Model | N ll(null) ll(model) df AIC BIC

-------------+---------------------------------------------------------------

. | 2,222 -379.8832 -316.6129 36 705.2258 910.6477

-----------------------------------------------------------------------------

Note: BIC uses N = number of observations. See [R] BIC note.

.

. *** Estimate the probability of switching for each patient-observation includ

> ed in the regression.***

. predict pxo2 if e(sample), pr

(76,884 missing values generated)

.

. *** IPCW step 3 with splines

.

. *** Estimate the probabilities of remaining 'un-switched' and hence the weigh

> ts ***

. sort SUBJID dthdyxtdc

. replace pxo1=pxo1*xotdc+(1-pxo1)*(1-xotdc)

(8,921 real changes made)

. replace pxo1=1 if pxo1==.

(70,094 real changes made)

. **Now we estimate each individual's probability of their complete censoring h

> istory up to each day**

. sort SUBJID dthdyxtdc

. by SUBJID: replace pxo1=pxo1*pxo1[_n-1] if _n!=1

(8893 real changes made)

. rename pxo1 num

.

. replace pxo2=pxo2*xotdc+(1-pxo2)*(1-xotdc)

(2,131 real changes made)

. replace pxo2=1 if pxo2==.

(76,884 real changes made)

. sort SUBJID dthdyxtdc

. by SUBJID: replace pxo2 = pxo2*pxo2[_n-1] if _n!=1

(2106 real changes made)

. rename pxo2 denom

.

. gen weight = 1 / denom if trtgrp==1

(49,968 missing values generated)

. gen sweight = num / denom if trtgrp==1

(49,968 missing values generated)

.

. *** set the weights to 1 in the treatment arm and if kras==1.

. replace weight = 1 if trtgrp==2 | krasi==1

(49,968 real changes made)

. replace sweight = 1 if trtgrp==2 | krasi==1

(49,968 real changes made)

.

. *** summarise the weights, and inspect the data to be confident that you have

> computed the weights correctly.

. summ sweight if xotdc==0 & krasi==2

Variable | Obs Mean Std. dev. Min Max

-------------+---------------------------------------------------------

sweight | 42,300 .9496964 .2460547 .0457893 14.4128

.

. *** IPCW step 4 with splines

.

. * Stabilised weights

. stset dthdyxtdc deathtdc if xotdc==0 [pw=sweight], time0(time)

Survival-time data settings

Failure event: deathtdc!=0 & deathtdc<.

Observed time interval: (time, dthdyxtdc]

Exit on or before: failure

Weight: [pweight=sweight]

Keep observations

if exp: xotdc==0

--------------------------------------------------------------------------

79,106 total observations

91 ignored at outset because of if exp

--------------------------------------------------------------------------

79,015 observations remaining, representing

306 failures in single-record/single-failure data

79,015 total analysis time at risk and under observation

At risk from t = 0

Earliest observed entry t = 0

Last observed exit t = 1,024

. *sts, by(trt) name(IPCWs_spline,replace) title(IPCW analysis for OS) ///

> * xlabel(#8) risktable(,format(%4.0f)) note(Stabilised weights)

. stcox trtgrp becogstrat regionstrat

Failure _d: deathtdc

Analysis time _t: dthdyxtdc

Weight: [pweight=sweight]

(sum of wgt is 76,887.1589408219)

Iteration 0: log pseudolikelihood = -1561.0217

Iteration 1: log pseudolikelihood = -1554.4147

Iteration 2: log pseudolikelihood = -1527.8293

Iteration 3: log pseudolikelihood = -1527.5325

Iteration 4: log pseudolikelihood = -1527.5323

Refining estimates:

Iteration 0: log pseudolikelihood = -1527.5323

Cox regression with Breslow method for ties

No. of subjects = 76,887 Number of obs = 79,015

No. of failures = 304

Time at risk = 76,887.1589

Wald chi2(3) = 82.25

Log pseudolikelihood = -1527.5323 Prob > chi2 = 0.0000

------------------------------------------------------------------------------

| Robust

_t | Haz. ratio std. err. z P>|z| [95% conf. interval]

-------------+----------------------------------------------------------------

trtgrp | .7496878 .0900667 -2.40 0.016 .592404 .9487305

becogstrat | 4.240594 .6899493 8.88 0.000 3.082728 5.833351

regionstrat | 1.003762 .0780357 0.05 0.961 .8618976 1.168977

------------------------------------------------------------------------------

.

. *****************************************************************************

> **********************

. *** IPCW 2 (primary analysis, full model, unstabilised weights) Estimand 1 [1

> 14 from IPCW file] ***

. *****************************************************************************

> **********************

.

. summ weight if xotdc==0 & krasi==2

Variable | Obs Mean Std. dev. Min Max

-------------+---------------------------------------------------------

weight | 42,300 1.057068 .6678692 1 41.32536

.

. stset dthdyxtdc deathtdc if xotdc==0 [pw=weight], time0(time)

Survival-time data settings

Failure event: deathtdc!=0 & deathtdc<.

Observed time interval: (time, dthdyxtdc]

Exit on or before: failure

Weight: [pweight=weight]

Keep observations

if exp: xotdc==0

--------------------------------------------------------------------------

79,106 total observations

91 ignored at outset because of if exp

--------------------------------------------------------------------------

79,015 observations remaining, representing

306 failures in single-record/single-failure data

79,015 total analysis time at risk and under observation

At risk from t = 0

Earliest observed entry t = 0

Last observed exit t = 1,024

. * sts, by(trt) name(IPCWu_spline,replace) title(IPCW analysis for OS) ///

> * xlabel(#8) risktable(,format(%4.0f)) note(Unstabilised weights)

. stcox trtgrp becogstrat regionstrat

Failure _d: deathtdc

Analysis time _t: dthdyxtdc

Weight: [pweight=weight]

(sum of wgt is 81,428.9920712709)

Iteration 0: log pseudolikelihood = -1626.6485

Iteration 1: log pseudolikelihood = -1618.9394

Iteration 2: log pseudolikelihood = -1592.3765

Iteration 3: log pseudolikelihood = -1591.9965

Iteration 4: log pseudolikelihood = -1591.9961

Refining estimates:

Iteration 0: log pseudolikelihood = -1591.9961

Cox regression with Breslow method for ties

No. of subjects = 81,429 Number of obs = 79,015

No. of failures = 333

Time at risk = 81,428.9921

Wald chi2(3) = 84.43

Log pseudolikelihood = -1591.9961 Prob > chi2 = 0.0000

------------------------------------------------------------------------------

| Robust

_t | Haz. ratio std. err. z P>|z| [95% conf. interval]

-------------+----------------------------------------------------------------

trtgrp | .710441 .0961523 -2.53 0.012 .5449102 .9262561

becogstrat | 4.190489 .7049561 8.52 0.000 3.013485 5.827206

regionstrat | .9491709 .0804103 -0.62 0.538 .8039586 1.120612

------------------------------------------------------------------------------

.

. restore

.

. *****************************************************************************

> ***********************

. *** IPCW 3 (primary analysis, reduced model, stabilised weights) Estimand 1 [

> 115 from IPCW file] ***

. *****************************************************************************

> ***********************

.

. preserve

.

. sort SUBJID dthdyxtdc

. by SUBJID: replace PDDYLR = xotime if (PDDYLR>xotime & xotime!=.)

(1,252 real changes made)

. by SUBJID: replace progtdc = 1 if (PDDYLR <= dthdyxtdc & PDLR==1)

(984 real changes made)

.

. replace xotdc = 0 if krasi==1 & trtgrp==1

(14,415 real changes made)

. replace xo = . if krasi==1 & trtgrp==1 & xo==1

(18,500 real changes made, 18,500 to missing)

.

. by SUBJID: drop if (dthdyxtdc>xotime & xo==1)

(22,711 observations deleted)

. by SUBJID: replace lastobs = 0

(336 real changes made)

. by SUBJID: replace lastobs = 1 if _n==_N

(427 real changes made)

.

. *** IPCW step 1 with splines

.

. *** Use logistic regression to predict switching given baseline covariates (m

> odel S1)***

. *** First use rcsgen to create splines for a time-dependent interecept***

. *** Generate 6 knots based upon the event time distribution (can try other am

> ounts of knots)***

. rcsgen dthdyxtdc, df(5) if2(xotdc==1) gen(timexosp)

Variables timexosp1 to timexosp5 were created

. * where are the knots?

. di r(knots)

3 43 60 68 78 335

. logistic xotdc becogstrat regionstrat timexosp* if trtgrp==1 & krasi==2

Logistic regression Number of obs = 9,012

LR chi2(7) = 90.79

Prob > chi2 = 0.0000

Log likelihood = -463.33157 Pseudo R2 = 0.0892

------------------------------------------------------------------------------

xotdc | Odds ratio Std. err. z P>|z| [95% conf. interval]

-------------+----------------------------------------------------------------

becogstrat | 1.413203 .5717528 0.85 0.393 .6394783 3.12308

regionstrat | .971615 .1596466 -0.18 0.861 .7040975 1.340774

timexosp1 | 1.006221 .0293224 0.21 0.831 .9503603 1.065365

timexosp2 | .9999761 .0000891 -0.27 0.788 .9998014 1.000151

timexosp3 | .9996842 .0003131 -1.01 0.313 .9990706 1.000298

timexosp4 | 1.000578 .0003259 1.77 0.076 .9999396 1.001217

timexosp5 | .9997511 .0000914 -2.72 0.006 .999572 .9999302

_cons | .0027814 .0016502 -9.92 0.000 .0008695 .0088979

------------------------------------------------------------------------------

Note: _cons estimates baseline odds.

.

. *** assess fit of the model, for comparison to alternatives that may be run l

> ater***

. estat gof, group(10) table

note: obs collapsed on 10 quantiles of estimated probabilities.

Goodness-of-fit test after logistic model

Variable: xotdc

Table collapsed on quantiles of estimated probabilities

+--------------------------------------------------------+

| Group | Prob | Obs_1 | Exp_1 | Obs_0 | Exp_0 | Total |

|-------+--------+-------+-------+-------+-------+-------|

| 1 | 0.0027 | 2 | 1.6 | 905 | 905.4 | 907 |

| 2 | 0.0029 | 3 | 2.8 | 961 | 961.2 | 964 |

| 3 | 0.0032 | 4 | 2.7 | 888 | 889.3 | 892 |

| 4 | 0.0038 | 2 | 2.9 | 842 | 841.1 | 844 |

| 5 | 0.0045 | 4 | 4.0 | 963 | 963.0 | 967 |

|-------+--------+-------+-------+-------+-------+-------|

| 6 | 0.0060 | 5 | 4.3 | 831 | 831.7 | 836 |

| 7 | 0.0091 | 4 | 6.7 | 903 | 900.3 | 907 |

| 8 | 0.0163 | 10 | 11.9 | 939 | 937.1 | 949 |

| 9 | 0.0293 | 20 | 19.1 | 833 | 833.9 | 853 |

| 10 | 0.0623 | 37 | 35.0 | 856 | 858.0 | 893 |

+--------------------------------------------------------+

Number of observations = 9,012

Number of groups = 10

Hosmer–Lemeshow chi2(8) = 2.66

Prob > chi2 = 0.9539

. estat ic

Akaike's information criterion and Bayesian information criterion

-----------------------------------------------------------------------------

Model | N ll(null) ll(model) df AIC BIC

-------------+---------------------------------------------------------------

. | 9,012 -508.7252 -463.3316 8 942.6631 999.5136

-----------------------------------------------------------------------------

Note: BIC uses N = number of observations. See [R] BIC note.

.

. *** Estimate the probability of switching for each patient-observation includ

> ed in the regression.***

. predict pxo1 if e(sample), pr

(70,094 missing values generated)

.

. *** Use logistic regression to predict switching given baseline and time-upda

> ted covariates (model S12)***

. logistic xotdc becogstrat diagtype eq5dbase eq5dmissb regionstrat PDDYLR eq5d

> tdc eq5dind eq5dmisslastvisit ecogtdc bestresptdc respmisslastvisit LSSLDtdc

> LSSLDmisslastvisit timexosp* if trtgrp==1 & krasi==2 & progtdc>0

Logistic regression Number of obs = 2,222

LR chi2(19) = 57.61

Prob > chi2 = 0.0000

Log likelihood = -351.07638 Pseudo R2 = 0.0758

-------------------------------------------------------------------------------

xotdc | Odds ratio Std. err. z P>|z| [95% conf. interval]

--------------+----------------------------------------------------------------

becogstrat | .9625939 .4640241 -0.08 0.937 .3742108 2.47611

diagtype | 1.440167 .3672958 1.43 0.153 .8736251 2.374109

eq5dbase | .5741495 .326739 -0.98 0.330 .1882003 1.751579

eq5dmissb | 1.746788 1.386686 0.70 0.482 .3685656 8.278769

regionstrat | 1.019827 .1897275 0.11 0.916 .7082227 1.468531

PDDYLR | .9995503 .0073479 -0.06 0.951 .9852519 1.014056

eq5dtdc | 1.099867 .5497778 0.19 0.849 .412914 2.929682

eq5dind | 1.891018 1.782856 0.68 0.499 .2979759 12.00081

eq5dmisslas~t | .5037031 .1658408 -2.08 0.037 .2641936 .9603442

ecogtdc | .7080805 .1341065 -1.82 0.068 .4885058 1.02635

bestresptdc | .8689508 .1976484 -0.62 0.537 .556396 1.357083

respmisslas~t | 1.255648 .4049038 0.71 0.480 .6673972 2.362389

LSSLDtdc | .9997512 .0009465 -0.26 0.793 .9978979 1.001608

LSSLDmissla~t | .3378867 .1707705 -2.15 0.032 .1254779 .9098604

timexosp1 | .940444 .0388478 -1.49 0.137 .8673044 1.019751

timexosp2 | 1.000117 .0001055 1.11 0.266 .9999107 1.000324

timexosp3 | .9992126 .0003523 -2.23 0.025 .9985222 .9999034

timexosp4 | 1.000974 .0003643 2.68 0.007 1.00026 1.001688

timexosp5 | .9996917 .0001034 -2.98 0.003 .9994891 .9998943

_cons | .3751705 .5967109 -0.62 0.538 .0166107 8.473638

-------------------------------------------------------------------------------

Note: _cons estimates baseline odds.

. * Note, had to take ecogmisslastvisit out as !=0 perfectly predicted failure

> and 763 obs not used.

.

. *** Assess model fit

. estat gof, group(10) table

note: obs collapsed on 10 quantiles of estimated probabilities.

Goodness-of-fit test after logistic model

Variable: xotdc

Table collapsed on quantiles of estimated probabilities

+--------------------------------------------------------+

| Group | Prob | Obs_1 | Exp_1 | Obs_0 | Exp_0 | Total |

|-------+--------+-------+-------+-------+-------+-------|

| 1 | 0.0064 | 0 | 1.0 | 223 | 222.0 | 223 |

| 2 | 0.0122 | 4 | 2.1 | 218 | 219.9 | 222 |

| 3 | 0.0192 | 2 | 3.4 | 220 | 218.6 | 222 |

| 4 | 0.0270 | 10 | 5.1 | 212 | 216.9 | 222 |

| 5 | 0.0333 | 6 | 6.7 | 216 | 215.3 | 222 |

|-------+--------+-------+-------+-------+-------+-------|

| 6 | 0.0417 | 7 | 8.3 | 216 | 214.7 | 223 |

| 7 | 0.0500 | 8 | 10.2 | 214 | 211.8 | 222 |

| 8 | 0.0639 | 12 | 12.7 | 210 | 209.3 | 222 |

| 9 | 0.0827 | 16 | 16.1 | 206 | 205.9 | 222 |

| 10 | 0.3024 | 26 | 25.4 | 196 | 196.6 | 222 |

+--------------------------------------------------------+

Number of observations = 2,222

Number of groups = 10

Hosmer–Lemeshow chi2(8) = 8.85

Prob > chi2 = 0.3549

. estat ic

Akaike's information criterion and Bayesian information criterion

-----------------------------------------------------------------------------

Model | N ll(null) ll(model) df AIC BIC

-------------+---------------------------------------------------------------

. | 2,222 -379.8832 -351.0764 20 742.1528 856.276

-----------------------------------------------------------------------------

Note: BIC uses N = number of observations. See [R] BIC note.

.

. *** Estimate the probability of switching for each patient-observation includ

> ed in the regression.***

. predict pxo2 if e(sample), pr

(76,884 missing values generated)

.

. *** IPCW step 3 with splines

.

. *** Estimate the probabilities of remaining 'un-switched' and hence the weigh

> ts ***

. sort SUBJID dthdyxtdc

. replace pxo1=pxo1*xotdc+(1-pxo1)*(1-xotdc)

(8,921 real changes made)

. replace pxo1=1 if pxo1==.

(70,094 real changes made)

. **Now we estimate each individual's probability of their complete censoring h

> istory up to each day**

. sort SUBJID dthdyxtdc

. by SUBJID: replace pxo1=pxo1*pxo1[_n-1] if _n!=1

(8893 real changes made)

. rename pxo1 num

.

. replace pxo2=pxo2*xotdc+(1-pxo2)*(1-xotdc)

(2,131 real changes made)

. replace pxo2=1 if pxo2==.

(76,884 real changes made)

. sort SUBJID dthdyxtdc

. by SUBJID: replace pxo2 = pxo2*pxo2[_n-1] if _n!=1

(2106 real changes made)

. rename pxo2 denom

.

. gen weight = 1 / denom if trtgrp==1

(49,968 missing values generated)

. gen sweight = num / denom if trtgrp==1

(49,968 missing values generated)

.

. *** set the weights to 1 in the treatment arm and if kras==1.

. replace weight = 1 if trtgrp==2 | krasi==1

(49,968 real changes made)

. replace sweight = 1 if trtgrp==2 | krasi==1

(49,968 real changes made)

.

. *** summarise the weights, and inspect the data to be confident that you have

> computed the weights correctly.

. summ sweight if xotdc==0 & krasi==2

Variable | Obs Mean Std. dev. Min Max

-------------+---------------------------------------------------------

sweight | 42,300 .9785685 .7734948 .0457893 32.95991

. scatter sweight time if trtgrp==1 & xotdc==0, ytitle(weight)

.

. *** IPCW step 4 with splines

. * Stabilised weights

. stset dthdyxtdc deathtdc if xotdc==0 [pw=sweight], time0(time)

Survival-time data settings

Failure event: deathtdc!=0 & deathtdc<.

Observed time interval: (time, dthdyxtdc]

Exit on or before: failure

Weight: [pweight=sweight]

Keep observations

if exp: xotdc==0

--------------------------------------------------------------------------

79,106 total observations

91 ignored at outset because of if exp

--------------------------------------------------------------------------

79,015 observations remaining, representing

306 failures in single-record/single-failure data

79,015 total analysis time at risk and under observation

At risk from t = 0

Earliest observed entry t = 0

Last observed exit t = 1,024

.

. *sts, by(trt) name(IPCWs_spline,replace) title(IPCW analysis for OS) ///

> * xlabel(#8) risktable(,format(%4.0f)) note(Stabilised weights)

. stcox trtgrp becogstrat regionstrat

Failure _d: deathtdc

Analysis time _t: dthdyxtdc

Weight: [pweight=sweight]

(sum of wgt is 78,108.4466132447)

Iteration 0: log pseudolikelihood = -1566.0402

Iteration 1: log pseudolikelihood = -1561.3479

Iteration 2: log pseudolikelihood = -1530.4375

Iteration 3: log pseudolikelihood = -1530.136

Iteration 4: log pseudolikelihood = -1530.1358

Refining estimates:

Iteration 0: log pseudolikelihood = -1530.1358

Cox regression with Breslow method for ties

No. of subjects = 78,108 Number of obs = 79,015

No. of failures = 309

Time at risk = 78,108.4466

Wald chi2(3) = 81.60

Log pseudolikelihood = -1530.1358 Prob > chi2 = 0.0000

------------------------------------------------------------------------------

| Robust

_t | Haz. ratio std. err. z P>|z| [95% conf. interval]

-------------+----------------------------------------------------------------

trtgrp | .7520799 .090693 -2.36 0.018 .5937696 .9525987

becogstrat | 4.403484 .7360179 8.87 0.000 3.173385 6.110405

regionstrat | .9626756 .0755344 -0.48 0.628 .8254526 1.12271

------------------------------------------------------------------------------

.

.

. *****************************************************************************

> *************************

. *** IPCW 4 (primary analysis, reduced model, unstabilised weights) Estimand 1

> [116 from IPCW file] ***

. *****************************************************************************

> *************************

.

. summ weight if xotdc==0

Variable | Obs Mean Std. dev. Min Max

-------------+---------------------------------------------------------

weight | 79,015 1.122304 3.310647 1 222.6042

.

. stset dthdyxtdc deathtdc if xotdc==0 [pw=weight], time0(time)

Survival-time data settings

Failure event: deathtdc!=0 & deathtdc<.

Observed time interval: (time, dthdyxtdc]

Exit on or before: failure

Weight: [pweight=weight]

Keep observations

if exp: xotdc==0

--------------------------------------------------------------------------

79,106 total observations

91 ignored at outset because of if exp

--------------------------------------------------------------------------

79,015 observations remaining, representing

306 failures in single-record/single-failure data

79,015 total analysis time at risk and under observation

At risk from t = 0

Earliest observed entry t = 0

Last observed exit t = 1,024

. * sts, by(trt) name(IPCWu_spline,replace) title(IPCW analysis for OS) ///

> * xlabel(#8) risktable(,format(%4.0f)) note(Unstabilised weights)

. stcox trtgrp becogstrat regionstrat

Failure _d: deathtdc

Analysis time _t: dthdyxtdc

Weight: [pweight=weight]

(sum of wgt is 88,678.8479765654)

Iteration 0: log pseudolikelihood = -1616.1755

Iteration 1: log pseudolikelihood = -1611.8533

Iteration 2: log pseudolikelihood = -1576.6722

Iteration 3: log pseudolikelihood = -1576.1348

Iteration 4: log pseudolikelihood = -1576.1341

Refining estimates:

Iteration 0: log pseudolikelihood = -1576.1341

Cox regression with Breslow method for ties

No. of subjects = 88,679 Number of obs = 79,015

No. of failures = 360

Time at risk = 88,678.848

Wald chi2(3) = 90.12

Log pseudolikelihood = -1576.1341 Prob > chi2 = 0.0000

------------------------------------------------------------------------------

| Robust

_t | Haz. ratio std. err. z P>|z| [95% conf. interval]

-------------+----------------------------------------------------------------

trtgrp | .7185631 .1123412 -2.11 0.035 .5289161 .9762096

becogstrat | 4.442997 .7826268 8.47 0.000 3.145851 6.275001

regionstrat | .7908006 .0892709 -2.08 0.038 .6338371 .9866346

------------------------------------------------------------------------------

.

. restore

.

. *****************************************************************************

> ********************

. *** IPCW 5 (secondary analysis, full model, stabilised weights) Estimand 1 [1

> 17 from IPCW file] ***

. *****************************************************************************

> ********************

.

. preserve

.

. * note, 8 patients switched before investigator observed progression. 7 were

> kras MT, so primary analyses will not adjust for these anyway.

. * protocol suggested switching permitted only after progression. So assume th

> ese patients switched due to some signs of progression.

. * need this, because IPCW models will fit much better if applied only to the

> time-periods where switching was "permitted".

. * so, for these 8 patients, replace PDDYLR to equal xotime

. * and replace progtdc to = 1 after this point for these patients

. sort SUBJID dthdyxtdc

. by SUBJID: replace PDDYLR = xotime if (PDDYLR>xotime & xotime!=.)

(1,252 real changes made)

. by SUBJID: replace progtdc = 1 if (PDDYLR <= dthdyxtdc & PDLR==1)

(984 real changes made)

.

. replace xotdc = 0 if krasi==1 & trtgrp==1

(14,415 real changes made)

. replace xo = . if krasi==1 & trtgrp==1 & xo==1

(18,500 real changes made, 18,500 to missing)

.

. by SUBJID: drop if (dthdyxtdc>xotime & xo==1)

(22,711 observations deleted)

. by SUBJID: replace lastobs = 0

(336 real changes made)

. by SUBJID: replace lastobs = 1 if _n==_N

(427 real changes made)

.

. *** IPCW step 1 with splines

.

. *** Use logistic regression to predict switching given baseline covariates (m

> odel S1)***

. *** First use rcsgen to create splines for a time-dependent interecept***

. *** Generate 5 knots based upon the event time distribution (can try other am

> ounts of knots)***

. rcsgen dthdyxtdc, df(5) if2(xotdc==1) gen(timexosp)

Variables timexosp1 to timexosp5 were created

. * where are the knots?

. di r(knots)

3 43 60 68 78 335

. logistic xotdc becogstrat regionstrat timexosp* if trtgrp==1

Logistic regression Number of obs = 29,138

LR chi2(7) = 117.99

Prob > chi2 = 0.0000

Log likelihood = -556.8356 Pseudo R2 = 0.0958

------------------------------------------------------------------------------

xotdc | Odds ratio Std. err. z P>|z| [95% conf. interval]

-------------+----------------------------------------------------------------

becogstrat | .887737 .3529883 -0.30 0.765 .4072148 1.935286

regionstrat | .8924505 .1394269 -0.73 0.466 .6570547 1.212179

timexosp1 | 1.002154 .0290382 0.07 0.941 .946826 1.060715

timexosp2 | .999943 .0000884 -0.64 0.519 .9997697 1.000116

timexosp3 | .999838 .0003104 -0.52 0.602 .9992299 1.000447

timexosp4 | 1.000414 .0003231 1.28 0.200 .9997809 1.001047

timexosp5 | .9997936 .0000908 -2.27 0.023 .9996157 .9999716

_cons | .0017268 .0010132 -10.84 0.000 .0005468 .0054538

------------------------------------------------------------------------------

Note: _cons estimates baseline odds.

.

. *** assess fit of the model, for comparison to alternatives that may be run l

> ater***

. estat gof, group(10) table

note: obs collapsed on 10 quantiles of estimated probabilities.

Goodness-of-fit test after logistic model

Variable: xotdc

Table collapsed on quantiles of estimated probabilities

+---------------------------------------------------------+

| Group | Prob | Obs_1 | Exp_1 | Obs_0 | Exp_0 | Total |

|-------+--------+-------+-------+-------+--------+-------|

| 1 | 0.0002 | 1 | 0.5 | 2925 | 2925.5 | 2926 |

| 2 | 0.0002 | 1 | 0.5 | 2901 | 2901.5 | 2902 |

| 3 | 0.0004 | 1 | 0.8 | 2917 | 2917.2 | 2918 |

| 4 | 0.0014 | 2 | 2.5 | 2925 | 2924.5 | 2927 |

| 5 | 0.0018 | 5 | 4.7 | 2892 | 2892.3 | 2897 |

|-------+--------+-------+-------+-------+--------+-------|

| 6 | 0.0020 | 7 | 5.5 | 2936 | 2937.5 | 2943 |

| 7 | 0.0029 | 8 | 7.2 | 2994 | 2994.8 | 3002 |

| 8 | 0.0050 | 6 | 10.8 | 2802 | 2797.2 | 2808 |

| 9 | 0.0096 | 16 | 20.9 | 2923 | 2918.1 | 2939 |

| 10 | 0.0161 | 44 | 37.7 | 2832 | 2838.3 | 2876 |

+---------------------------------------------------------+

Number of observations = 29,138

Number of groups = 10

Hosmer–Lemeshow chi2(8) = 6.07

Prob > chi2 = 0.6390

. estat ic

Akaike's information criterion and Bayesian information criterion

-----------------------------------------------------------------------------

Model | N ll(null) ll(model) df AIC BIC

-------------+---------------------------------------------------------------

. | 29,138 -615.8312 -556.8356 8 1129.671 1195.91

-----------------------------------------------------------------------------

Note: BIC uses N = number of observations. See [R] BIC note.

.

. *** Estimate the probability of switching for each patient-observation includ

> ed in the regression.***

. predict pxo1 if e(sample), pr

(49,968 missing values generated)

.

. *** Use logistic regression to predict switching given baseline and time-upda

> ted covariates (model S12)***

. logistic xotdc AGE becogstrat diagtype BILIULN ASTULN CREATULN ALBULN LDHULN

> eq5dbase CEAULN eq5dmissb regionstrat PDDYLR eq5dtdc eq5dind eq5dmisslastvisi

> t ecogtdc bestresptdc respmisslastvisit LSSLDtdc LSSLDmisslastvisit AATULNtdc

> AATmisslastvisit ALBULNtdc ALBmisslastvisit ALKULNtdc ASTULNtdc ASTmisslastv

> isit CEAULNtdc CEAmisslastvisit CREATmisslastvisit LDHULNtdc LDHmisslastvisit

> BILIULNtdc BILImisslastvisit saetdc timexosp* if trtgrp==1 & progtdc>0

note: ALBmisslastvisit omitted because of collinearity.

note: ASTmisslastvisit omitted because of collinearity.

note: CREATmisslastvisit omitted because of collinearity.

note: LDHmisslastvisit omitted because of collinearity.

note: BILImisslastvisit omitted because of collinearity.

Logistic regression Number of obs = 18,272

LR chi2(36) = 291.54

Prob > chi2 = 0.0000

Log likelihood = -427.50728 Pseudo R2 = 0.2543

-------------------------------------------------------------------------------

xotdc | Odds ratio Std. err. z P>|z| [95% conf. interval]

--------------+----------------------------------------------------------------

AGE | .9843539 .0115378 -1.35 0.178 .9619981 1.007229

becogstrat | .694304 .347329 -0.73 0.466 .2604545 1.850834

diagtype | 1.000881 .2564561 0.00 0.997 .6057296 1.653812

BILIULN | 1.109153 .4369996 0.26 0.793 .5124163 2.40082

ASTULN | .5263829 .1908003 -1.77 0.077 .2586801 1.071126

CREATULN | .3448927 .3821792 -0.96 0.337 .039306 3.026281

ALBULN | 1.075858 .4553094 0.17 0.863 .4693765 2.465973

LDHULN | .9448671 .3446201 -0.16 0.876 .4622886 1.931205

eq5dbase | .5910094 .3552164 -0.88 0.382 .1819675 1.91953

CEAULN | .6513831 .2398149 -1.16 0.244 .31656 1.340346

eq5dmissb | 1.409686 1.107263 0.44 0.662 .3023644 6.57225

regionstrat | .9583462 .1633606 -0.25 0.803 .6861606 1.338502

PDDYLR | 1.029893 .0102458 2.96 0.003 1.010006 1.050171

eq5dtdc | 1.545653 .891714 0.75 0.450 .4989291 4.78834

eq5dind | 1.18072 1.102794 0.18 0.859 .1892881 7.364959

eq5dmisslas~t | .6447222 .2159288 -1.31 0.190 .3344185 1.242954

ecogtdc | .7605728 .150072 -1.39 0.165 .5166381 1.119683

bestresptdc | .8769905 .1850692 -0.62 0.534 .5799197 1.326239

respmisslas~t | 1.337101 .4201647 0.92 0.355 .7222483 2.475381

LSSLDtdc | .9995649 .0011004 -0.40 0.693 .9974105 1.001724

LSSLDmissla~t | .3757118 .1681304 -2.19 0.029 .1562945 .9031631

AATULNtdc | .970393 .2931235 -0.10 0.921 .5368191 1.754152

AATmisslast~t | .355137 .2396706 -1.53 0.125 .0946122 1.333045

ALBULNtdc | 1.376808 .4322348 1.02 0.308 .7441276 2.547414

ALBmisslast~t | 1 (omitted)

ALKULNtdc | 1.403437 .4952429 0.96 0.337 .7027841 2.802617

ASTULNtdc | 2.432125 .9427086 2.29 0.022 1.13777 5.198973

ASTmisslast~t | 1 (omitted)

CEAULNtdc | 1.43079 .8116244 0.63 0.528 .4706808 4.349358

CEAmisslast~t | .386767 .1585071 -2.32 0.020 .1732228 .8635623

CREATmissla~t | 1 (omitted)

LDHULNtdc | 1.526699 .5815109 1.11 0.267 .723662 3.220855

LDHmisslast~t | 1 (omitted)

BILIULNtdc | .6045854 .2198178 -1.38 0.166 .2964654 1.232938

BILImisslas~t | 1 (omitted)

saetdc | .2633589 .2730271 -1.29 0.198 .0345222 2.009084

timexosp1 | .9230061 .0381435 -1.94 0.053 .8511938 1.000877

timexosp2 | 1.000023 .0001049 0.22 0.828 .9998173 1.000228

timexosp3 | .9995718 .0003522 -1.22 0.224 .9988818 1.000262

timexosp4 | 1.000614 .000364 1.69 0.092 .9999007 1.001327

timexosp5 | .9997809 .0001029 -2.13 0.033 .9995793 .9999825

_cons | .2255134 .381266 -0.88 0.378 .0082051 6.198162

-------------------------------------------------------------------------------

Note: _cons estimates baseline odds.

Note: 348 failures and 0 successes completely determined.

. * Note, had to take ecogmisslastvisit out as !=0 perfectly predicted failure,

> and ALKmisslastvisit because missing SE hidden colinearity. Also removed CRE

> ATULNtdc as coefficient and SE crazy.

.

. *** Assess model fit

. estat gof, group(10) table

note: obs collapsed on 10 quantiles of estimated probabilities.

Goodness-of-fit test after logistic model

Variable: xotdc

Table collapsed on quantiles of estimated probabilities

+---------------------------------------------------------+

| Group | Prob | Obs_1 | Exp_1 | Obs_0 | Exp_0 | Total |

|-------+--------+-------+-------+-------+--------+-------|

| 1 | 0.0000 | 0 | 0.0 | 1828 | 1828.0 | 1828 |

| 2 | 0.0000 | 0 | 0.0 | 1827 | 1827.0 | 1827 |

| 3 | 0.0000 | 0 | 0.0 | 1827 | 1827.0 | 1827 |

| 4 | 0.0000 | 0 | 0.0 | 1827 | 1827.0 | 1827 |

| 5 | 0.0001 | 0 | 0.1 | 1827 | 1826.9 | 1827 |

|-------+--------+-------+-------+-------+--------+-------|

| 6 | 0.0003 | 0 | 0.4 | 1828 | 1827.6 | 1828 |

| 7 | 0.0011 | 2 | 1.1 | 1825 | 1825.9 | 1827 |

| 8 | 0.0053 | 5 | 5.1 | 1822 | 1821.9 | 1827 |

| 9 | 0.0178 | 24 | 19.0 | 1803 | 1808.0 | 1827 |

| 10 | 0.1651 | 60 | 65.2 | 1767 | 1761.8 | 1827 |

+---------------------------------------------------------+

Number of observations = 18,272

Number of groups = 10

Hosmer–Lemeshow chi2(8) = 2.95

Prob > chi2 = 0.9377

. estat ic

Akaike's information criterion and Bayesian information criterion

-----------------------------------------------------------------------------

Model | N ll(null) ll(model) df AIC BIC

-------------+---------------------------------------------------------------

. | 18,272 -573.2792 -427.5073 37 929.0146 1218.1

-----------------------------------------------------------------------------

Note: BIC uses N = number of observations. See [R] BIC note.

.

. *** Estimate the probability of switching for each patient-observation includ

> ed in the regression.***

. predict pxo2 if e(sample), pr

(60,834 missing values generated)

.

. *** IPCW step 3 with splines

.

. *** Estimate the probabilities of remaining 'un-switched' and hence the weigh

> ts ***

. sort SUBJID dthdyxtdc

. replace pxo1=pxo1*xotdc+(1-pxo1)*(1-xotdc)

(29,047 real changes made)

. replace pxo1=1 if pxo1==.

(49,968 real changes made)

. **Now we estimate each individual's probability of their complete censoring h

> istory up to each day**

. sort SUBJID dthdyxtdc

. by SUBJID: replace pxo1=pxo1*pxo1[_n-1] if _n!=1

(28919 real changes made)

. rename pxo1 num

.

. replace pxo2=pxo2*xotdc+(1-pxo2)*(1-xotdc)

(18,181 real changes made)

. replace pxo2=1 if pxo2==.

(60,834 real changes made)

. sort SUBJID dthdyxtdc

. by SUBJID: replace pxo2 = pxo2*pxo2[_n-1] if _n!=1

(18057 real changes made)

. rename pxo2 denom

.

. gen weight = 1 / denom if trtgrp==1

(49,968 missing values generated)

. gen sweight = num / denom if trtgrp==1

(49,968 missing values generated)

.

. *** set the weights to 1 in the treatment arm.

. replace weight = 1 if trtgrp==2

(49,968 real changes made)

. replace sweight = 1 if trtgrp==2

(49,968 real changes made)

.

. *** summarise the weights, and inspect the data to be confident that you have

> computed the weights correctly.

. summ sweight if xotdc==0

Variable | Obs Mean Std. dev. Min Max

-------------+---------------------------------------------------------

sweight | 79,015 .9836546 .2067353 .4666828 5.660593

.

. *** IPCW step 4 with splines

.

. * Stabilised weights

. stset dthdyxtdc deathtdc if xotdc==0 [pw=sweight], time0(time)

Survival-time data settings

Failure event: deathtdc!=0 & deathtdc<.

Observed time interval: (time, dthdyxtdc]

Exit on or before: failure

Weight: [pweight=sweight]

Keep observations

if exp: xotdc==0

--------------------------------------------------------------------------

79,106 total observations

91 ignored at outset because of if exp

--------------------------------------------------------------------------

79,015 observations remaining, representing

306 failures in single-record/single-failure data

79,015 total analysis time at risk and under observation

At risk from t = 0

Earliest observed entry t = 0

Last observed exit t = 1,024

. * sts, by(trt) name(IPCWs_spline,replace) title(IPCW analysis for OS) ///

> * xlabel(#8) risktable(,format(%4.0f)) note(Stabilised weights)

. stcox trtgrp becogstrat regionstrat

Failure _d: deathtdc

Analysis time _t: dthdyxtdc

Weight: [pweight=sweight]

(sum of wgt is 77,723.4666788876)

Iteration 0: log pseudolikelihood = -1556.7305

Iteration 1: log pseudolikelihood = -1550.911

Iteration 2: log pseudolikelihood = -1524.0158

Iteration 3: log pseudolikelihood = -1523.6952

Iteration 4: log pseudolikelihood = -1523.6949

Refining estimates:

Iteration 0: log pseudolikelihood = -1523.6949

Cox regression with Breslow method for ties

No. of subjects = 77,723 Number of obs = 79,015

No. of failures = 306

Time at risk = 77,723.4667

Wald chi2(3) = 85.24

Log pseudolikelihood = -1523.6949 Prob > chi2 = 0.0000

------------------------------------------------------------------------------

| Robust

_t | Haz. ratio std. err. z P>|z| [95% conf. interval]

-------------+----------------------------------------------------------------

trtgrp | .7527879 .092208 -2.32 0.020 .5921205 .9570511

becogstrat | 4.281107 .6889601 9.04 0.000 3.122999 5.868678

regionstrat | 1.004686 .0791015 0.06 0.953 .8610198 1.172324

------------------------------------------------------------------------------

.

.

. *****************************************************************************

> *************************

. *** IPCW 6 (secondary analysis, full model, unstabilised weights) Estimand 1

> [118 from IPCW file] ***

. *****************************************************************************

> *************************

.

. summ weight if xotdc==0

Variable | Obs Mean Std. dev. Min Max

-------------+---------------------------------------------------------

weight | 79,015 1.181974 .5163031 1 8.310938

.

. stset dthdyxtdc deathtdc if xotdc==0 [pw=weight], time0(time)

Survival-time data settings

Failure event: deathtdc!=0 & deathtdc<.

Observed time interval: (time, dthdyxtdc]

Exit on or before: failure

Weight: [pweight=weight]

Keep observations

if exp: xotdc==0

--------------------------------------------------------------------------

79,106 total observations

91 ignored at outset because of if exp

--------------------------------------------------------------------------

79,015 observations remaining, representing

306 failures in single-record/single-failure data

79,015 total analysis time at risk and under observation

At risk from t = 0

Earliest observed entry t = 0

Last observed exit t = 1,024

. * sts, by(trt) name(IPCWu_spline,replace) title(IPCW analysis for OS) ///

> * xlabel(#8) risktable(,format(%4.0f)) note(Unstabilised weights)

. stcox trtgrp becogstrat regionstrat

Failure _d: deathtdc

Analysis time _t: dthdyxtdc

Weight: [pweight=weight]

(sum of wgt is 93,393.7075513601)

Iteration 0: log pseudolikelihood = -1648.6404

Iteration 1: log pseudolikelihood = -1643.427

Iteration 2: log pseudolikelihood = -1618.0536

Iteration 3: log pseudolikelihood = -1617.6401

Iteration 4: log pseudolikelihood = -1617.6396

Refining estimates:

Iteration 0: log pseudolikelihood = -1617.6396

Cox regression with Breslow method for ties

No. of subjects = 93,394 Number of obs = 79,015

No. of failures = 390

Time at risk = 93,393.7076

Wald chi2(3) = 79.71

Log pseudolikelihood = -1617.6396 Prob > chi2 = 0.0000

------------------------------------------------------------------------------

| Robust

_t | Haz. ratio std. err. z P>|z| [95% conf. interval]

-------------+----------------------------------------------------------------

trtgrp | .7593725 .0930904 -2.25 0.025 .5971828 .9656114

becogstrat | 4.174208 .6917829 8.62 0.000 3.01652 5.776196

regionstrat | .9803923 .0824327 -0.24 0.814 .8314378 1.156032

------------------------------------------------------------------------------

.

. restore

.

. *****************************************************************************

> ********************

. *** IPCW 7 (secondary analysis, reduced model, stabilised weights) Estimand 1

> [119 from IPCW file] ***

. *****************************************************************************

> ********************

.

. preserve

.

. * note, 8 patients switched before investigator observed progression. 7 were

> kras MT, so primary analyses will not adjust for these anyway.

. * protocol suggested switching permitted only after progression. So assume th

> ese patients switched due to some signs of progression.

. * need this, because IPCW models will fit much better if applied only to the

> time-periods where switching was "permitted".

. * so, for these 8 patients, replace PDDYLR to equal xotime

. * and replace progtdc to = 1 after this point for these patients

. sort SUBJID dthdyxtdc

. by SUBJID: replace PDDYLR = xotime if (PDDYLR>xotime & xotime!=.)

(1,252 real changes made)

. by SUBJID: replace progtdc = 1 if (PDDYLR <= dthdyxtdc & PDLR==1)

(984 real changes made)

.

. replace xotdc = 0 if krasi==1 & trtgrp==1

(14,415 real changes made)

. replace xo = . if krasi==1 & trtgrp==1 & xo==1

(18,500 real changes made, 18,500 to missing)

.

. by SUBJID: drop if (dthdyxtdc>xotime & xo==1)

(22,711 observations deleted)

. by SUBJID: replace lastobs = 0

(336 real changes made)

. by SUBJID: replace lastobs = 1 if _n==_N

(427 real changes made)

.

. *** IPCW step 1 with splines

.

. *** Use logistic regression to predict switching given baseline covariates (m

> odel S1)***

. *** First use rcsgen to create splines for a time-dependent interecept***

. *** Generate 6 knots based upon the event time distribution (can try other am

> ounts of knots)***

. rcsgen dthdyxtdc, df(5) if2(xotdc==1) gen(timexosp)

Variables timexosp1 to timexosp5 were created

. * where are the knots?

. di r(knots)

3 43 60 68 78 335

. logistic xotdc becogstrat regionstrat timexosp* if trtgrp==1

Logistic regression Number of obs = 29,138

LR chi2(7) = 117.99

Prob > chi2 = 0.0000

Log likelihood = -556.8356 Pseudo R2 = 0.0958

------------------------------------------------------------------------------

xotdc | Odds ratio Std. err. z P>|z| [95% conf. interval]

-------------+----------------------------------------------------------------

becogstrat | .887737 .3529883 -0.30 0.765 .4072148 1.935286

regionstrat | .8924505 .1394269 -0.73 0.466 .6570547 1.212179

timexosp1 | 1.002154 .0290382 0.07 0.941 .946826 1.060715

timexosp2 | .999943 .0000884 -0.64 0.519 .9997697 1.000116

timexosp3 | .999838 .0003104 -0.52 0.602 .9992299 1.000447

timexosp4 | 1.000414 .0003231 1.28 0.200 .9997809 1.001047

timexosp5 | .9997936 .0000908 -2.27 0.023 .9996157 .9999716

_cons | .0017268 .0010132 -10.84 0.000 .0005468 .0054538

------------------------------------------------------------------------------

Note: _cons estimates baseline odds.

.

. *** assess fit of the model, for comparison to alternatives that may be run l

> ater***

. estat gof, group(10) table

note: obs collapsed on 10 quantiles of estimated probabilities.

Goodness-of-fit test after logistic model

Variable: xotdc

Table collapsed on quantiles of estimated probabilities

+---------------------------------------------------------+

| Group | Prob | Obs_1 | Exp_1 | Obs_0 | Exp_0 | Total |

|-------+--------+-------+-------+-------+--------+-------|

| 1 | 0.0002 | 1 | 0.5 | 2925 | 2925.5 | 2926 |

| 2 | 0.0002 | 1 | 0.5 | 2901 | 2901.5 | 2902 |

| 3 | 0.0004 | 1 | 0.8 | 2917 | 2917.2 | 2918 |

| 4 | 0.0014 | 2 | 2.5 | 2925 | 2924.5 | 2927 |

| 5 | 0.0018 | 5 | 4.7 | 2892 | 2892.3 | 2897 |

|-------+--------+-------+-------+-------+--------+-------|

| 6 | 0.0020 | 7 | 5.5 | 2936 | 2937.5 | 2943 |

| 7 | 0.0029 | 8 | 7.2 | 2994 | 2994.8 | 3002 |

| 8 | 0.0050 | 6 | 10.8 | 2802 | 2797.2 | 2808 |

| 9 | 0.0096 | 16 | 20.9 | 2923 | 2918.1 | 2939 |

| 10 | 0.0161 | 44 | 37.7 | 2832 | 2838.3 | 2876 |

+---------------------------------------------------------+

Number of observations = 29,138

Number of groups = 10

Hosmer–Lemeshow chi2(8) = 6.07

Prob > chi2 = 0.6390

. estat ic

Akaike's information criterion and Bayesian information criterion

-----------------------------------------------------------------------------

Model | N ll(null) ll(model) df AIC BIC

-------------+---------------------------------------------------------------

. | 29,138 -615.8312 -556.8356 8 1129.671 1195.91

-----------------------------------------------------------------------------

Note: BIC uses N = number of observations. See [R] BIC note.

.

. *** Estimate the probability of switching for each patient-observation includ

> ed in the regression.***

. predict pxo1 if e(sample), pr

(49,968 missing values generated)

.

. *** Use logistic regression to predict switching given baseline and time-upda

> ted covariates (model S12)***

. logistic xotdc becogstrat diagtype eq5dbase eq5dmissb regionstrat PDDYLR eq5d

> tdc eq5dind eq5dmisslastvisit ecogtdc bestresptdc respmisslastvisit LSSLDtdc

> LSSLDmisslastvisit timexosp* if trtgrp==1 & progtdc>0

Logistic regression Number of obs = 18,272

LR chi2(19) = 251.77

Prob > chi2 = 0.0000

Log likelihood = -447.39258 Pseudo R2 = 0.2196

-------------------------------------------------------------------------------

xotdc | Odds ratio Std. err. z P>|z| [95% conf. interval]

--------------+----------------------------------------------------------------

becogstrat | .6017097 .2823666 -1.08 0.279 .2398504 1.509502

diagtype | 1.049851 .2505912 0.20 0.839 .657586 1.676111

eq5dbase | .6141219 .3243276 -0.92 0.356 .2181331 1.728971

eq5dmissb | 1.291905 .9712174 0.34 0.733 .2960202 5.63819

regionstrat | .9549368 .1534423 -0.29 0.774 .6969487 1.308424

PDDYLR | 1.031388 .009716 3.28 0.001 1.01252 1.050608

eq5dtdc | 1.845916 .9838002 1.15 0.250 .6494642 5.246488

eq5dind | .9603732 .8573719 -0.05 0.964 .1669287 5.525214

eq5dmisslas~t | .4272395 .1374074 -2.64 0.008 .2274629 .8024761

ecogtdc | .9171834 .1602372 -0.49 0.621 .6512479 1.291713

bestresptdc | .850841 .1679782 -0.82 0.413 .5778286 1.252846

respmisslas~t | 1.244165 .3774427 0.72 0.471 .686513 2.254796

LSSLDtdc | 1.00058 .0008736 0.66 0.506 .9988695 1.002294

LSSLDmissla~t | .2826981 .1258291 -2.84 0.005 .1181551 .676384

timexosp1 | .8917623 .0337703 -3.03 0.002 .8279705 .960469

timexosp2 | .9999914 .0000986 -0.09 0.931 .9997982 1.000185

timexosp3 | .9995847 .0003351 -1.24 0.215 .998928 1.000242

timexosp4 | 1.000648 .000348 1.86 0.063 .9999661 1.00133

timexosp5 | .9997582 .0000987 -2.45 0.014 .9995647 .9999517

_cons | .2573698 .3429401 -1.02 0.308 .0188951 3.505634

-------------------------------------------------------------------------------

Note: _cons estimates baseline odds.

Note: 47 failures and 0 successes completely determined.

. * Note, had to take ecogmisslastvisit out as !=0 perfectly predicted failure

> and 763 obs not used.

.

. *** Assess model fit

. estat gof, group(10) table

note: obs collapsed on 10 quantiles of estimated probabilities.

Goodness-of-fit test after logistic model

Variable: xotdc

Table collapsed on quantiles of estimated probabilities

+---------------------------------------------------------+

| Group | Prob | Obs_1 | Exp_1 | Obs_0 | Exp_0 | Total |

|-------+--------+-------+-------+-------+--------+-------|

| 1 | 0.0000 | 0 | 0.0 | 1828 | 1828.0 | 1828 |

| 2 | 0.0000 | 0 | 0.0 | 1827 | 1827.0 | 1827 |

| 3 | 0.0000 | 0 | 0.0 | 1827 | 1827.0 | 1827 |

| 4 | 0.0001 | 0 | 0.1 | 1827 | 1826.9 | 1827 |

| 5 | 0.0002 | 0 | 0.2 | 1827 | 1826.8 | 1827 |

|-------+--------+-------+-------+-------+--------+-------|

| 6 | 0.0008 | 1 | 0.8 | 1827 | 1827.2 | 1828 |

| 7 | 0.0018 | 1 | 2.2 | 1826 | 1824.8 | 1827 |

| 8 | 0.0072 | 9 | 6.5 | 1818 | 1820.5 | 1827 |

| 9 | 0.0194 | 28 | 23.9 | 1799 | 1803.1 | 1827 |

| 10 | 0.1153 | 52 | 57.3 | 1775 | 1769.7 | 1827 |

+---------------------------------------------------------+

Number of observations = 18,272

Number of groups = 10

Hosmer–Lemeshow chi2(8) = 3.27

Prob > chi2 = 0.9162

. estat ic

Akaike's information criterion and Bayesian information criterion

-----------------------------------------------------------------------------

Model | N ll(null) ll(model) df AIC BIC

-------------+---------------------------------------------------------------

. | 18,272 -573.2792 -447.3926 20 934.7852 1091.048

-----------------------------------------------------------------------------

Note: BIC uses N = number of observations. See [R] BIC note.

.

. *** Estimate the probability of switching for each patient-observation includ

> ed in the regression.***

. predict pxo2 if e(sample), pr

(60,834 missing values generated)

.

. *** IPCW step 3 with splines

.

. *** Estimate the probabilities of remaining 'un-switched' and hence the weigh

> ts ***

. sort SUBJID dthdyxtdc

. replace pxo1=pxo1*xotdc+(1-pxo1)*(1-xotdc)

(29,047 real changes made)

. replace pxo1=1 if pxo1==.

(49,968 real changes made)

. **Now we estimate each individual's probability of their complete censoring h

> istory up to each day**

. sort SUBJID dthdyxtdc

. by SUBJID: replace pxo1=pxo1*pxo1[_n-1] if _n!=1

(28919 real changes made)

. rename pxo1 num

.

. replace pxo2=pxo2*xotdc+(1-pxo2)*(1-xotdc)

(18,181 real changes made)

. replace pxo2=1 if pxo2==.

(60,834 real changes made)

. sort SUBJID dthdyxtdc

. by SUBJID: replace pxo2 = pxo2*pxo2[_n-1] if _n!=1

(18057 real changes made)

. rename pxo2 denom

.

. gen weight = 1 / denom if trtgrp==1

(49,968 missing values generated)

. gen sweight = num / denom if trtgrp==1

(49,968 missing values generated)

.

. *** set the weights to 1 in the treatment arm.

. replace weight = 1 if trtgrp==2

(49,968 real changes made)

. replace sweight = 1 if trtgrp==2

(49,968 real changes made)

.

. *** summarise the weights, and inspect the data to be confident that you have

> computed the weights correctly.

. summ sweight if xotdc==0

Variable | Obs Mean Std. dev. Min Max

-------------+---------------------------------------------------------

sweight | 79,015 .9927507 .1583681 .4666828 2.397694

.

. *** IPCW step 4 with splines

.

. * Stabilised weights

. stset dthdyxtdc deathtdc if xotdc==0 [pw=sweight], time0(time)

Survival-time data settings

Failure event: deathtdc!=0 & deathtdc<.

Observed time interval: (time, dthdyxtdc]

Exit on or before: failure

Weight: [pweight=sweight]

Keep observations

if exp: xotdc==0

--------------------------------------------------------------------------

79,106 total observations

91 ignored at outset because of if exp

--------------------------------------------------------------------------

79,015 observations remaining, representing

306 failures in single-record/single-failure data

79,015 total analysis time at risk and under observation

At risk from t = 0

Earliest observed entry t = 0

Last observed exit t = 1,024

. * sts, by(trt) name(IPCWs_spline,replace) title(IPCW analysis for OS) ///

> * xlabel(#8) risktable(,format(%4.0f)) note(Stabilised weights)

. stcox trtgrp becogstrat regionstrat

Failure _d: deathtdc

Analysis time _t: dthdyxtdc

Weight: [pweight=sweight]

(sum of wgt is 78,442.1963531971)

Iteration 0: log pseudolikelihood = -1535.7447

Iteration 1: log pseudolikelihood = -1531.3672

Iteration 2: log pseudolikelihood = -1503.5115

Iteration 3: log pseudolikelihood = -1503.2018

Iteration 4: log pseudolikelihood = -1503.2015

Refining estimates:

Iteration 0: log pseudolikelihood = -1503.2015

Cox regression with Breslow method for ties

No. of subjects = 78,442 Number of obs = 79,015

No. of failures = 305

Time at risk = 78,442.1964

Wald chi2(3) = 85.39

Log pseudolikelihood = -1503.2015 Prob > chi2 = 0.0000

------------------------------------------------------------------------------

| Robust

_t | Haz. ratio std. err. z P>|z| [95% conf. interval]

-------------+----------------------------------------------------------------

trtgrp | .7942155 .0946981 -1.93 0.053 .6287031 1.003301

becogstrat | 4.31701 .6910427 9.14 0.000 3.154478 5.907974

regionstrat | 1.000702 .0780423 0.01 0.993 .8588582 1.165971

------------------------------------------------------------------------------

.

. *****************************************************************************

> *************************

. *** IPCW 8 (secondary analysis, reduced model, unstabilised weights) Estimand

> 1 [120 from IPCW file] ***

. *****************************************************************************

> *************************

.

. summ weight if xotdc==0

Variable | Obs Mean Std. dev. Min Max

-------------+---------------------------------------------------------

weight | 79,015 1.19961 .470138 1 4.192906

.

. stset dthdyxtdc deathtdc if xotdc==0 [pw=weight], time0(time)

Survival-time data settings

Failure event: deathtdc!=0 & deathtdc<.

Observed time interval: (time, dthdyxtdc]

Exit on or before: failure

Weight: [pweight=weight]

Keep observations

if exp: xotdc==0

--------------------------------------------------------------------------

79,106 total observations

91 ignored at outset because of if exp

--------------------------------------------------------------------------

79,015 observations remaining, representing

306 failures in single-record/single-failure data

79,015 total analysis time at risk and under observation

At risk from t = 0

Earliest observed entry t = 0

Last observed exit t = 1,024

. * sts, by(trt) name(IPCWu_spline,replace) title(IPCW analysis for OS) ///

> * xlabel(#8) risktable(,format(%4.0f)) note(Unstabilised weights)

. stcox trtgrp becogstrat regionstrat

Failure _d: deathtdc

Analysis time _t: dthdyxtdc

Weight: [pweight=weight]

(sum of wgt is 94,787.1723617315)

Iteration 0: log pseudolikelihood = -1608.8433

Iteration 1: log pseudolikelihood = -1605.5463

Iteration 2: log pseudolikelihood = -1579.0375

Iteration 3: log pseudolikelihood = -1578.6303

Iteration 4: log pseudolikelihood = -1578.6298

Refining estimates:

Iteration 0: log pseudolikelihood = -1578.6298

Cox regression with Breslow method for ties

No. of subjects = 94,787 Number of obs = 79,015

No. of failures = 386

Time at risk = 94,787.1724

Wald chi2(3) = 80.83

Log pseudolikelihood = -1578.6298 Prob > chi2 = 0.0000

------------------------------------------------------------------------------

| Robust

_t | Haz. ratio std. err. z P>|z| [95% conf. interval]

-------------+----------------------------------------------------------------

trtgrp | .8043674 .0961334 -1.82 0.069 .6363905 1.016682

becogstrat | 4.219455 .6883011 8.83 0.000 3.064811 5.809104

regionstrat | .9768953 .0804665 -0.28 0.777 .8312561 1.148051

------------------------------------------------------------------------------

.

. restore

.

end of do-file

. do "C:\Users\cm1nrl\AppData\Local\Temp\STD4384_000000.tmp"

. *** PANI ANALYSES FOR PAPER: ESTIMAND 2 ***

.

. *****************************************************

. ******************** ITT *************************

. *****************************************************

.

. use "X:\ScHARR\Users\cm1nrl\Case studies\Amgen\Data\Final merged dataset\base

> line_master", clear

(TREAT)

.

. ********************** estimand 2

. drop if krasi==.

(36 observations deleted)

. drop if krasi==1

(184 observations deleted)

. stset DTHDYX, id(SUBJID) failure (DTHX) scale(30.4375)

Survival-time data settings

ID variable: SUBJID

Failure event: DTHX!=0 & DTHX<.

Observed time interval: (DTHDYX[_n-1], DTHDYX]

Exit on or before: failure

Time for analysis: time/30.4375

--------------------------------------------------------------------------

243 total observations

0 exclusions

--------------------------------------------------------------------------

243 observations remaining, representing

243 subjects

217 failures in single-failure-per-subject data

2,138.858 total analysis time at risk and under observation

At risk from t = 0

Earliest observed entry t = 0

Last observed exit t = 33.64271

. stcox trtgrp regionstrat becogstrat

Failure _d: DTHX

Analysis time _t: DTHDYX/30.4375

ID variable: SUBJID

Iteration 0: log likelihood = -1002.4728

Iteration 1: log likelihood = -997.66169

Iteration 2: log likelihood = -989.77631

Iteration 3: log likelihood = -989.57079

Iteration 4: log likelihood = -989.57048

Refining estimates:

Iteration 0: log likelihood = -989.57048

Cox regression with Breslow method for ties

No. of subjects = 243 Number of obs = 243

No. of failures = 217

Time at risk = 2,138.8583

LR chi2(3) = 25.80

Log likelihood = -989.57048 Prob > chi2 = 0.0000

------------------------------------------------------------------------------

_t | Haz. ratio Std. err. z P>|z| [95% conf. interval]

-------------+----------------------------------------------------------------

trtgrp | .9901681 .1359122 -0.07 0.943 .756609 1.295825

regionstrat | .9410402 .086703 -0.66 0.510 .7855657 1.127285

becogstrat | 3.202117 .6524707 5.71 0.000 2.147803 4.773973

------------------------------------------------------------------------------

.

. *****************************************************************************

> ********************

. *** IPCW 1 (primary analysis, full model, stabilised weights) Estimand 2 [125

> from IPCW file] ***

. *****************************************************************************

> ********************

. use "X:\ScHARR\Users\cm1nrl\Case studies\Amgen\Data\Interim merged datasets\t

> dc_dataset34.dta", clear

(TREAT)

.

. preserve

.

. * estimand 2, with primary analysis: drop all kras MT patients

. drop if krasi==1

(36,715 observations deleted)

.

. sort SUBJID dthdyxtdc

. by SUBJID: replace PDDYLR = xotime if (PDDYLR>xotime & xotime!=.)

(181 real changes made)

. by SUBJID: replace progtdc = 1 if (PDDYLR <= dthdyxtdc & PDLR==1)

(178 real changes made)

.

. by SUBJID: drop if (dthdyxtdc>xotime & xo==1)

(22,711 observations deleted)

. by SUBJID: replace lastobs = 0

(152 real changes made)

. by SUBJID: replace lastobs = 1 if _n==_N

(243 real changes made)

.

. *** IPCW step 1 with splines

.

. *** Use logistic regression to predict switching given baseline covariates (m

> odel S1)***

. *** First use rcsgen to create splines for a time-dependent interecept***

. *** Generate 6 knots based upon the event time distribution (can try other am

> ounts of knots)***

. rcsgen dthdyxtdc, df(5) if2(xotdc==1) gen(timexosp)

Variables timexosp1 to timexosp5 were created

. * where are the knots?

. di r(knots)

3 43 60 68 78 335

. logistic xotdc becogstrat regionstrat timexosp* if trtgrp==1

Logistic regression Number of obs = 9,012

LR chi2(7) = 90.79

Prob > chi2 = 0.0000

Log likelihood = -463.33157 Pseudo R2 = 0.0892

------------------------------------------------------------------------------

xotdc | Odds ratio Std. err. z P>|z| [95% conf. interval]

-------------+----------------------------------------------------------------

becogstrat | 1.413203 .5717528 0.85 0.393 .6394783 3.12308

regionstrat | .971615 .1596466 -0.18 0.861 .7040975 1.340774

timexosp1 | 1.006221 .0293224 0.21 0.831 .9503603 1.065365

timexosp2 | .9999761 .0000891 -0.27 0.788 .9998014 1.000151

timexosp3 | .9996842 .0003131 -1.01 0.313 .9990706 1.000298

timexosp4 | 1.000578 .0003259 1.77 0.076 .9999396 1.001217

timexosp5 | .9997511 .0000914 -2.72 0.006 .999572 .9999302

_cons | .0027814 .0016502 -9.92 0.000 .0008695 .0088979

------------------------------------------------------------------------------

Note: _cons estimates baseline odds.

.

. *** assess fit of the model, for comparison to alternatives that may be run l

> ater***

. estat gof, group(10) table

note: obs collapsed on 10 quantiles of estimated probabilities.

Goodness-of-fit test after logistic model

Variable: xotdc

Table collapsed on quantiles of estimated probabilities

+--------------------------------------------------------+

| Group | Prob | Obs_1 | Exp_1 | Obs_0 | Exp_0 | Total |

|-------+--------+-------+-------+-------+-------+-------|

| 1 | 0.0027 | 2 | 1.6 | 905 | 905.4 | 907 |

| 2 | 0.0029 | 3 | 2.8 | 961 | 961.2 | 964 |

| 3 | 0.0032 | 4 | 2.7 | 888 | 889.3 | 892 |

| 4 | 0.0038 | 2 | 2.9 | 842 | 841.1 | 844 |

| 5 | 0.0045 | 4 | 4.0 | 963 | 963.0 | 967 |

|-------+--------+-------+-------+-------+-------+-------|

| 6 | 0.0060 | 5 | 4.3 | 831 | 831.7 | 836 |

| 7 | 0.0091 | 4 | 6.7 | 903 | 900.3 | 907 |

| 8 | 0.0163 | 10 | 11.9 | 939 | 937.1 | 949 |

| 9 | 0.0293 | 20 | 19.1 | 833 | 833.9 | 853 |

| 10 | 0.0623 | 37 | 35.0 | 856 | 858.0 | 893 |

+--------------------------------------------------------+

Number of observations = 9,012

Number of groups = 10

Hosmer–Lemeshow chi2(8) = 2.66

Prob > chi2 = 0.9539

. estat ic

Akaike's information criterion and Bayesian information criterion

-----------------------------------------------------------------------------

Model | N ll(null) ll(model) df AIC BIC

-------------+---------------------------------------------------------------

. | 9,012 -508.7252 -463.3316 8 942.6631 999.5136

-----------------------------------------------------------------------------

Note: BIC uses N = number of observations. See [R] BIC note.

.

. *** Estimate the probability of switching for each patient-observation includ

> ed in the regression.***

. predict pxo1 if e(sample), pr

(33,379 missing values generated)

.

. *** Use logistic regression to predict switching given baseline and time-upda

> ted covariates (model S12)***

. logistic xotdc AGE becogstrat diagtype BILIULN ASTULN CREATULN ALBULN LDHULN

> eq5dbase CEAULN eq5dmissb regionstrat PDDYLR eq5dtdc eq5dind eq5dmisslastvisi

> t ecogtdc bestresptdc respmisslastvisit LSSLDtdc LSSLDmisslastvisit AATULNtdc

> AATmisslastvisit ALBULNtdc ALBmisslastvisit ALKULNtdc ALKmisslastvisit ASTUL

> Ntdc ASTmisslastvisit CEAULNtdc CEAmisslastvisit CREATmisslastvisit LDHULNtdc

> LDHmisslastvisit BILIULNtdc BILImisslastvisit timexosp* if trtgrp==1 & progt

> dc>0

note: ALBmisslastvisit omitted because of collinearity.

note: ALKmisslastvisit omitted because of collinearity.

note: ASTmisslastvisit omitted because of collinearity.

note: CREATmisslastvisit omitted because of collinearity.

note: LDHmisslastvisit omitted because of collinearity.

note: BILImisslastvisit omitted because of collinearity.

Logistic regression Number of obs = 2,222

LR chi2(35) = 126.54

Prob > chi2 = 0.0000

Log likelihood = -316.61291 Pseudo R2 = 0.1666

-------------------------------------------------------------------------------

xotdc | Odds ratio Std. err. z P>|z| [95% conf. interval]

--------------+----------------------------------------------------------------

AGE | .9868856 .0131012 -0.99 0.320 .9615389 1.0129

becogstrat | .999556 .5734768 -0.00 0.999 .3246729 3.077289

diagtype | 1.383078 .4672014 0.96 0.337 .7133661 2.681521

BILIULN | 1.94632 .9552079 1.36 0.175 .7438146 5.092885

ASTULN | .2896219 .1378194 -2.60 0.009 .1139669 .7360105

CREATULN | 1.3873 1.708745 0.27 0.790 .1240912 15.50958

ALBULN | .7466926 .3967427 -0.55 0.582 .2635543 2.115503

LDHULN | 1.227953 .5967152 0.42 0.673 .4737468 3.182857

eq5dbase | .4617082 .3089724 -1.15 0.248 .1243794 1.713905

CEAULN | .768319 .3119722 -0.65 0.516 .3466693 1.702816

eq5dmissb | .9074362 .7676654 -0.11 0.909 .1728716 4.763307

regionstrat | .7623227 .169055 -1.22 0.221 .4935985 1.177345

PDDYLR | .988393 .0085777 -1.35 0.179 .9717232 1.005349

eq5dtdc | 1.435989 .8668345 0.60 0.549 .439869 4.687905

eq5dind | .4700634 .4971443 -0.71 0.475 .0591447 3.735913

eq5dmisslas~t | 1.581828 .5785153 1.25 0.210 .7724186 3.239407

ecogtdc | .6539933 .1389212 -2.00 0.046 .4312817 .9917118

bestresptdc | .9332468 .2265922 -0.28 0.776 .5798624 1.501994

respmisslas~t | 1.309429 .4766581 0.74 0.459 .6415462 2.672614

LSSLDtdc | .9988725 .0014529 -0.78 0.438 .9960288 1.001724

LSSLDmissla~t | .8816743 .4632389 -0.24 0.811 .3148327 2.469087

AATULNtdc | 1.310922 .4082315 0.87 0.385 .7120427 2.413503

AATmisslast~t | .0997322 .0791923 -2.90 0.004 .0210348 .4728592

ALBULNtdc | 1.986922 .7458566 1.83 0.067 .9520379 4.146745

ALBmisslast~t | 1 (omitted)

ALKULNtdc | 1.291451 .5387566 0.61 0.540 .5701395 2.925331

ALKmisslast~t | 1 (omitted)

ASTULNtdc | 1.739563 .7541859 1.28 0.202 .7437084 4.068907

ASTmisslast~t | 1 (omitted)

CEAULNtdc | .6231413 .4464003 -0.66 0.509 .153042 2.537246

CEAmisslast~t | .1624489 .0770435 -3.83 0.000 .0641244 .411538

CREATmissla~t | 1 (omitted)

LDHULNtdc | 1.555114 .7330146 0.94 0.349 .6173649 3.91726

LDHmisslast~t | 1 (omitted)

BILIULNtdc | .7099261 .2756174 -0.88 0.378 .3317018 1.519422

BILImisslas~t | 1 (omitted)

timexosp1 | 1.032927 .0548102 0.61 0.542 .930899 1.146138

timexosp2 | 1.000156 .000121 1.29 0.196 .9999192 1.000394

timexosp3 | .9993284 .0003967 -1.69 0.091 .9985512 1.000106

timexosp4 | 1.000742 .0004101 1.81 0.070 .9999381 1.001546

timexosp5 | .9997845 .0001169 -1.84 0.065 .9995554 1.000014

_cons | .4379219 .9264943 -0.39 0.696 .006927 27.68539

-------------------------------------------------------------------------------

Note: _cons estimates baseline odds.

. * Note, had to take ecogmisslastvisit out as !=0 perfectly predicted failure.

> Also removed saetdc and CREATULDtdc as these had crazy coefficients and SEs.

.

. *** Assess model fit

. estat gof, group(10) table

note: obs collapsed on 10 quantiles of estimated probabilities.

Goodness-of-fit test after logistic model

Variable: xotdc

Table collapsed on quantiles of estimated probabilities

+--------------------------------------------------------+

| Group | Prob | Obs_1 | Exp_1 | Obs_0 | Exp_0 | Total |

|-------+--------+-------+-------+-------+-------+-------|

| 1 | 0.0019 | 1 | 0.3 | 222 | 222.7 | 223 |

| 2 | 0.0030 | 0 | 0.6 | 222 | 221.4 | 222 |

| 3 | 0.0045 | 1 | 0.8 | 221 | 221.2 | 222 |

| 4 | 0.0079 | 1 | 1.4 | 221 | 220.6 | 222 |

| 5 | 0.0197 | 5 | 2.6 | 217 | 219.4 | 222 |

|-------+--------+-------+-------+-------+-------+-------|

| 6 | 0.0381 | 6 | 6.8 | 217 | 216.2 | 223 |

| 7 | 0.0544 | 10 | 10.2 | 212 | 211.8 | 222 |

| 8 | 0.0699 | 6 | 13.7 | 216 | 208.3 | 222 |

| 9 | 0.1049 | 23 | 18.2 | 199 | 203.8 | 222 |

| 10 | 0.3902 | 38 | 36.5 | 184 | 185.5 | 222 |

+--------------------------------------------------------+

Number of observations = 2,222

Number of groups = 10

Hosmer–Lemeshow chi2(8) = 10.69

Prob > chi2 = 0.2197

. estat ic

Akaike's information criterion and Bayesian information criterion

-----------------------------------------------------------------------------

Model | N ll(null) ll(model) df AIC BIC

-------------+---------------------------------------------------------------

. | 2,222 -379.8832 -316.6129 36 705.2258 910.6477

-----------------------------------------------------------------------------

Note: BIC uses N = number of observations. See [R] BIC note.

.

. *** Estimate the probability of switching for each patient-observation includ

> ed in the regression.***

. predict pxo2 if e(sample), pr

(40,169 missing values generated)

.

. *** IPCW step 3 with splines

.

. *** Estimate the probabilities of remaining 'un-switched' and hence the weigh

> ts ***

. sort SUBJID dthdyxtdc

. replace pxo1=pxo1*xotdc+(1-pxo1)*(1-xotdc)

(8,921 real changes made)

. replace pxo1=1 if pxo1==.

(33,379 real changes made)

. **Now we estimate each individual's probability of their complete censoring h

> istory up to each day**

. sort SUBJID dthdyxtdc

. by SUBJID: replace pxo1=pxo1*pxo1[_n-1] if _n!=1

(8893 real changes made)

. rename pxo1 num

.

. replace pxo2=pxo2*xotdc+(1-pxo2)*(1-xotdc)

(2,131 real changes made)

. replace pxo2=1 if pxo2==.

(40,169 real changes made)

. sort SUBJID dthdyxtdc

. by SUBJID: replace pxo2 = pxo2*pxo2[_n-1] if _n!=1

(2106 real changes made)

. rename pxo2 denom

.

. gen weight = 1 / denom if trtgrp==1

(33,379 missing values generated)

. gen sweight = num / denom if trtgrp==1

(33,379 missing values generated)

.

. *** set the weights to 1 in the treatment arm

. replace weight = 1 if trtgrp==2

(33,379 real changes made)

. replace sweight = 1 if trtgrp==2

(33,379 real changes made)

.

. *** summarise the weights, and inspect the data to be confident that you have

> computed the weights correctly.

. summ sweight if xotdc==0

Variable | Obs Mean Std. dev. Min Max

-------------+---------------------------------------------------------

sweight | 42,300 .9496964 .2460547 .0457893 14.4128

.

. *** IPCW step 4 with splines

.

. * Stabilised weights

. stset dthdyxtdc deathtdc if xotdc==0 [pw=sweight], time0(time)

Survival-time data settings

Failure event: deathtdc!=0 & deathtdc<.

Observed time interval: (time, dthdyxtdc]

Exit on or before: failure

Weight: [pweight=sweight]

Keep observations

if exp: xotdc==0

--------------------------------------------------------------------------

42,391 total observations

91 ignored at outset because of if exp

--------------------------------------------------------------------------

42,300 observations remaining, representing

132 failures in single-record/single-failure data

42,300 total analysis time at risk and under observation

At risk from t = 0

Earliest observed entry t = 0

Last observed exit t = 1,024

. *sts, by(trt) name(IPCWs_spline,replace) title(IPCW analysis for OS) ///

> * xlabel(#8) risktable(,format(%4.0f)) note(Stabilised weights)

. stcox trtgrp becogstrat regionstrat

Failure _d: deathtdc

Analysis time _t: dthdyxtdc

Weight: [pweight=sweight]

(sum of wgt is 40,172.1589408219)

Iteration 0: log pseudolikelihood = -591.07637

Iteration 1: log pseudolikelihood = -570.19959

Iteration 2: log pseudolikelihood = -569.55743

Iteration 3: log pseudolikelihood = -569.55717

Refining estimates:

Iteration 0: log pseudolikelihood = -569.55717

Cox regression with Breslow method for ties

No. of subjects = 40,172 Number of obs = 42,300

No. of failures = 130

Time at risk = 40,172.1589

Wald chi2(3) = 47.62

Log pseudolikelihood = -569.55717 Prob > chi2 = 0.0000

------------------------------------------------------------------------------

| Robust

_t | Haz. ratio std. err. z P>|z| [95% conf. interval]

-------------+----------------------------------------------------------------

trtgrp | .550081 .1911142 -1.72 0.085 .2784161 1.086823

becogstrat | 5.733474 1.475545 6.79 0.000 3.46223 9.494669

regionstrat | .9102429 .1033484 -0.83 0.408 .728639 1.137109

------------------------------------------------------------------------------

.

. *****************************************************************************

> **********************

. *** IPCW 2 (primary analysis, full model, unstabilised weights) Estimand 2 [1

> 26 from IPCW file] ***

. *****************************************************************************

> **********************

.

. summ weight if xotdc==0

Variable | Obs Mean Std. dev. Min Max

-------------+---------------------------------------------------------

weight | 42,300 1.057068 .6678692 1 41.32536

.

. stset dthdyxtdc deathtdc if xotdc==0 [pw=weight], time0(time)

Survival-time data settings

Failure event: deathtdc!=0 & deathtdc<.

Observed time interval: (time, dthdyxtdc]

Exit on or before: failure

Weight: [pweight=weight]

Keep observations

if exp: xotdc==0

--------------------------------------------------------------------------

42,391 total observations

91 ignored at outset because of if exp

--------------------------------------------------------------------------

42,300 observations remaining, representing

132 failures in single-record/single-failure data

42,300 total analysis time at risk and under observation

At risk from t = 0

Earliest observed entry t = 0

Last observed exit t = 1,024

. * sts, by(trt) name(IPCWu_spline,replace) title(IPCW analysis for OS) ///

> * xlabel(#8) risktable(,format(%4.0f)) note(Unstabilised weights)

. stcox trtgrp becogstrat regionstrat

Failure _d: deathtdc

Analysis time _t: dthdyxtdc

Weight: [pweight=weight]

(sum of wgt is 44,713.9920712709)

Iteration 0: log pseudolikelihood = -660.34542

Iteration 1: log pseudolikelihood = -655.3833

Iteration 2: log pseudolikelihood = -634.68208

Iteration 3: log pseudolikelihood = -634.60659

Iteration 4: log pseudolikelihood = -634.60657

Refining estimates:

Iteration 0: log pseudolikelihood = -634.60657

Cox regression with Breslow method for ties

No. of subjects = 44,714 Number of obs = 42,300

No. of failures = 159

Time at risk = 44,713.9921

Wald chi2(3) = 51.89

Log pseudolikelihood = -634.60657 Prob > chi2 = 0.0000

------------------------------------------------------------------------------

| Robust

_t | Haz. ratio std. err. z P>|z| [95% conf. interval]

-------------+----------------------------------------------------------------

trtgrp | .4620191 .1405667 -2.54 0.011 .2544993 .8387513

becogstrat | 5.420119 1.405328 6.52 0.000 3.260689 9.009659

regionstrat | .815292 .1131204 -1.47 0.141 .6211698 1.070079

------------------------------------------------------------------------------

.

. restore

.

. *****************************************************************************

> ***********************

. *** IPCW 3 (primary analysis, reduced model, stabilised weights) Estimand 2 [

> 127 from IPCW file] ***

. *****************************************************************************

> ***********************

.

. preserve

.

. * note, 8 patients switched before investigator observed progression. 7 were

> kras MT, so primary analyses will not adjust for these anyway.

. * protocol suggested switching permitted only after progression. So assume th

> ese patients switched due to some signs of progression.

. * need this, because IPCW models will fit much better if applied only to the

> time-periods where switching was "permitted".

. * so, for these 8 patients, replace PDDYLR to equal xotime

. * and replace progtdc to = 1 after this point for these patients

.

. * estimand 2, with primary analysis: drop all kras MT patients

. drop if krasi==1

(36,715 observations deleted)

.

. sort SUBJID dthdyxtdc

. by SUBJID: replace PDDYLR = xotime if (PDDYLR>xotime & xotime!=.)

(181 real changes made)

. by SUBJID: replace progtdc = 1 if (PDDYLR <= dthdyxtdc & PDLR==1)

(178 real changes made)

.

. by SUBJID: drop if (dthdyxtdc>xotime & xo==1)

(22,711 observations deleted)

. by SUBJID: replace lastobs = 0

(152 real changes made)

. by SUBJID: replace lastobs = 1 if _n==_N

(243 real changes made)

.

. *** IPCW step 1 with splines

.

. *** Use logistic regression to predict switching given baseline covariates (m

> odel S1)***

. *** First use rcsgen to create splines for a time-dependent interecept***

. *** Generate 6 knots based upon the event time distribution (can try other am

> ounts of knots)***

. rcsgen dthdyxtdc, df(5) if2(xotdc==1) gen(timexosp)

Variables timexosp1 to timexosp5 were created

. * where are the knots?

. di r(knots)

3 43 60 68 78 335

. logistic xotdc becogstrat regionstrat timexosp* if trtgrp==1

Logistic regression Number of obs = 9,012

LR chi2(7) = 90.79

Prob > chi2 = 0.0000

Log likelihood = -463.33157 Pseudo R2 = 0.0892

------------------------------------------------------------------------------

xotdc | Odds ratio Std. err. z P>|z| [95% conf. interval]

-------------+----------------------------------------------------------------

becogstrat | 1.413203 .5717528 0.85 0.393 .6394783 3.12308

regionstrat | .971615 .1596466 -0.18 0.861 .7040975 1.340774

timexosp1 | 1.006221 .0293224 0.21 0.831 .9503603 1.065365

timexosp2 | .9999761 .0000891 -0.27 0.788 .9998014 1.000151

timexosp3 | .9996842 .0003131 -1.01 0.313 .9990706 1.000298

timexosp4 | 1.000578 .0003259 1.77 0.076 .9999396 1.001217

timexosp5 | .9997511 .0000914 -2.72 0.006 .999572 .9999302

_cons | .0027814 .0016502 -9.92 0.000 .0008695 .0088979

------------------------------------------------------------------------------

Note: _cons estimates baseline odds.

.

. *** assess fit of the model, for comparison to alternatives that may be run l

> ater***

. estat gof, group(10) table

note: obs collapsed on 10 quantiles of estimated probabilities.

Goodness-of-fit test after logistic model

Variable: xotdc

Table collapsed on quantiles of estimated probabilities

+--------------------------------------------------------+

| Group | Prob | Obs_1 | Exp_1 | Obs_0 | Exp_0 | Total |

|-------+--------+-------+-------+-------+-------+-------|

| 1 | 0.0027 | 2 | 1.6 | 905 | 905.4 | 907 |

| 2 | 0.0029 | 3 | 2.8 | 961 | 961.2 | 964 |

| 3 | 0.0032 | 4 | 2.7 | 888 | 889.3 | 892 |

| 4 | 0.0038 | 2 | 2.9 | 842 | 841.1 | 844 |

| 5 | 0.0045 | 4 | 4.0 | 963 | 963.0 | 967 |

|-------+--------+-------+-------+-------+-------+-------|

| 6 | 0.0060 | 5 | 4.3 | 831 | 831.7 | 836 |

| 7 | 0.0091 | 4 | 6.7 | 903 | 900.3 | 907 |

| 8 | 0.0163 | 10 | 11.9 | 939 | 937.1 | 949 |

| 9 | 0.0293 | 20 | 19.1 | 833 | 833.9 | 853 |

| 10 | 0.0623 | 37 | 35.0 | 856 | 858.0 | 893 |

+--------------------------------------------------------+

Number of observations = 9,012

Number of groups = 10

Hosmer–Lemeshow chi2(8) = 2.66

Prob > chi2 = 0.9539

. estat ic

Akaike's information criterion and Bayesian information criterion

-----------------------------------------------------------------------------

Model | N ll(null) ll(model) df AIC BIC

-------------+---------------------------------------------------------------

. | 9,012 -508.7252 -463.3316 8 942.6631 999.5136

-----------------------------------------------------------------------------

Note: BIC uses N = number of observations. See [R] BIC note.

.

. *** Estimate the probability of switching for each patient-observation includ

> ed in the regression.***

. predict pxo1 if e(sample), pr

(33,379 missing values generated)

.

. *** Use logistic regression to predict switching given baseline and time-upda

> ted covariates (model S12)***

. logistic xotdc becogstrat diagtype eq5dbase eq5dmissb regionstrat PDDYLR eq5d

> tdc eq5dind eq5dmisslastvisit ecogtdc bestresptdc respmisslastvisit LSSLDtdc

> LSSLDmisslastvisit timexosp* if trtgrp==1 & progtdc>0

Logistic regression Number of obs = 2,222

LR chi2(19) = 57.61

Prob > chi2 = 0.0000

Log likelihood = -351.07638 Pseudo R2 = 0.0758

-------------------------------------------------------------------------------

xotdc | Odds ratio Std. err. z P>|z| [95% conf. interval]

--------------+----------------------------------------------------------------

becogstrat | .9625939 .4640241 -0.08 0.937 .3742108 2.47611

diagtype | 1.440167 .3672958 1.43 0.153 .8736251 2.374109

eq5dbase | .5741495 .326739 -0.98 0.330 .1882003 1.751579

eq5dmissb | 1.746788 1.386686 0.70 0.482 .3685656 8.278769

regionstrat | 1.019827 .1897275 0.11 0.916 .7082227 1.468531

PDDYLR | .9995503 .0073479 -0.06 0.951 .9852519 1.014056

eq5dtdc | 1.099867 .5497778 0.19 0.849 .412914 2.929682

eq5dind | 1.891018 1.782856 0.68 0.499 .2979759 12.00081

eq5dmisslas~t | .5037031 .1658408 -2.08 0.037 .2641936 .9603442

ecogtdc | .7080805 .1341065 -1.82 0.068 .4885058 1.02635

bestresptdc | .8689508 .1976484 -0.62 0.537 .556396 1.357083

respmisslas~t | 1.255648 .4049038 0.71 0.480 .6673972 2.362389

LSSLDtdc | .9997512 .0009465 -0.26 0.793 .9978979 1.001608

LSSLDmissla~t | .3378867 .1707705 -2.15 0.032 .1254779 .9098604

timexosp1 | .940444 .0388478 -1.49 0.137 .8673044 1.019751

timexosp2 | 1.000117 .0001055 1.11 0.266 .9999107 1.000324

timexosp3 | .9992126 .0003523 -2.23 0.025 .9985222 .9999034

timexosp4 | 1.000974 .0003643 2.68 0.007 1.00026 1.001688

timexosp5 | .9996917 .0001034 -2.98 0.003 .9994891 .9998943

_cons | .3751705 .5967109 -0.62 0.538 .0166107 8.473638

-------------------------------------------------------------------------------

Note: _cons estimates baseline odds.

. * Note, had to take ecogmisslastvisit out as !=0 perfectly predicted failure.

.

. *** Assess model fit

. estat gof, group(10) table

note: obs collapsed on 10 quantiles of estimated probabilities.

Goodness-of-fit test after logistic model

Variable: xotdc

Table collapsed on quantiles of estimated probabilities

+--------------------------------------------------------+

| Group | Prob | Obs_1 | Exp_1 | Obs_0 | Exp_0 | Total |

|-------+--------+-------+-------+-------+-------+-------|

| 1 | 0.0064 | 0 | 1.0 | 223 | 222.0 | 223 |

| 2 | 0.0122 | 4 | 2.1 | 218 | 219.9 | 222 |

| 3 | 0.0192 | 2 | 3.4 | 220 | 218.6 | 222 |

| 4 | 0.0270 | 10 | 5.1 | 212 | 216.9 | 222 |

| 5 | 0.0333 | 6 | 6.7 | 216 | 215.3 | 222 |

|-------+--------+-------+-------+-------+-------+-------|

| 6 | 0.0417 | 7 | 8.3 | 216 | 214.7 | 223 |

| 7 | 0.0500 | 8 | 10.2 | 214 | 211.8 | 222 |

| 8 | 0.0639 | 12 | 12.7 | 210 | 209.3 | 222 |

| 9 | 0.0827 | 16 | 16.1 | 206 | 205.9 | 222 |

| 10 | 0.3024 | 26 | 25.4 | 196 | 196.6 | 222 |

+--------------------------------------------------------+

Number of observations = 2,222

Number of groups = 10

Hosmer–Lemeshow chi2(8) = 8.85

Prob > chi2 = 0.3549

. estat ic

Akaike's information criterion and Bayesian information criterion

-----------------------------------------------------------------------------

Model | N ll(null) ll(model) df AIC BIC

-------------+---------------------------------------------------------------

. | 2,222 -379.8832 -351.0764 20 742.1528 856.276

-----------------------------------------------------------------------------

Note: BIC uses N = number of observations. See [R] BIC note.

.

. *** Estimate the probability of switching for each patient-observation includ

> ed in the regression.***

. predict pxo2 if e(sample), pr

(40,169 missing values generated)

.

. *** IPCW step 3 with splines

.

. *** Estimate the probabilities of remaining 'un-switched' and hence the weigh

> ts ***

. sort SUBJID dthdyxtdc

. replace pxo1=pxo1*xotdc+(1-pxo1)*(1-xotdc)

(8,921 real changes made)

. replace pxo1=1 if pxo1==.

(33,379 real changes made)

. **Now we estimate each individual's probability of their complete censoring h

> istory up to each day**

. sort SUBJID dthdyxtdc

. by SUBJID: replace pxo1=pxo1*pxo1[_n-1] if _n!=1

(8893 real changes made)

. rename pxo1 num

.

. replace pxo2=pxo2*xotdc+(1-pxo2)*(1-xotdc)

(2,131 real changes made)

. replace pxo2=1 if pxo2==.

(40,169 real changes made)

. sort SUBJID dthdyxtdc

. by SUBJID: replace pxo2 = pxo2*pxo2[_n-1] if _n!=1

(2106 real changes made)

. rename pxo2 denom

.

. gen weight = 1 / denom if trtgrp==1

(33,379 missing values generated)

. gen sweight = num / denom if trtgrp==1

(33,379 missing values generated)

.

. *** set the weights to 1 in the treatment arm

. replace weight = 1 if trtgrp==2

(33,379 real changes made)

. replace sweight = 1 if trtgrp==2

(33,379 real changes made)

.

. *** summarise the weights, and inspect the data to be confident that you have

> computed the weights correctly.

. summ sweight if xotdc==0

Variable | Obs Mean Std. dev. Min Max

-------------+---------------------------------------------------------

sweight | 42,300 .9785685 .7734948 .0457893 32.95991

.

. *** IPCW step 4 with splines

.

. * Stabilised weights

. stset dthdyxtdc deathtdc if xotdc==0 [pw=sweight], time0(time)

Survival-time data settings

Failure event: deathtdc!=0 & deathtdc<.

Observed time interval: (time, dthdyxtdc]

Exit on or before: failure

Weight: [pweight=sweight]

Keep observations

if exp: xotdc==0

--------------------------------------------------------------------------

42,391 total observations

91 ignored at outset because of if exp

--------------------------------------------------------------------------

42,300 observations remaining, representing

132 failures in single-record/single-failure data

42,300 total analysis time at risk and under observation

At risk from t = 0

Earliest observed entry t = 0

Last observed exit t = 1,024

. *sts, by(trt) name(IPCWs_spline,replace) title(IPCW analysis for OS) ///

> * xlabel(#8) risktable(,format(%4.0f)) note(Stabilised weights)

. stcox trtgrp becogstrat regionstrat

Failure _d: deathtdc

Analysis time _t: dthdyxtdc

Weight: [pweight=sweight]

(sum of wgt is 41,393.4466132447)

Iteration 0: log pseudolikelihood = -598.74681

Iteration 1: log pseudolikelihood = -574.45576

Iteration 2: log pseudolikelihood = -573.52624

Iteration 3: log pseudolikelihood = -573.52533

Refining estimates:

Iteration 0: log pseudolikelihood = -573.52533

Cox regression with Breslow method for ties

No. of subjects = 41,393 Number of obs = 42,300

No. of failures = 135

Time at risk = 41,393.4466

Wald chi2(3) = 50.07

Log pseudolikelihood = -573.52533 Prob > chi2 = 0.0000

------------------------------------------------------------------------------

| Robust

_t | Haz. ratio std. err. z P>|z| [95% conf. interval]

-------------+----------------------------------------------------------------

trtgrp | .5389768 .1676679 -1.99 0.047 .2929366 .9916686

becogstrat | 6.258834 1.661391 6.91 0.000 3.720011 10.53034

regionstrat | .8614075 .1002296 -1.28 0.200 .6857513 1.082058

------------------------------------------------------------------------------

.

. *****************************************************************************

> *************************

. *** IPCW 4 (primary analysis, reduced model, unstabilised weights) Estimand 2

> [128 from IPCW file] ***

. *****************************************************************************

> *************************

.

. summ weight if xotdc==0

Variable | Obs Mean Std. dev. Min Max

-------------+---------------------------------------------------------

weight | 42,300 1.22846 4.522124 1 222.6042

.

. stset dthdyxtdc deathtdc if xotdc==0 [pw=weight], time0(time)

Survival-time data settings

Failure event: deathtdc!=0 & deathtdc<.

Observed time interval: (time, dthdyxtdc]

Exit on or before: failure

Weight: [pweight=weight]

Keep observations

if exp: xotdc==0

--------------------------------------------------------------------------

42,391 total observations

91 ignored at outset because of if exp

--------------------------------------------------------------------------

42,300 observations remaining, representing

132 failures in single-record/single-failure data

42,300 total analysis time at risk and under observation

At risk from t = 0

Earliest observed entry t = 0

Last observed exit t = 1,024

. * sts, by(trt) name(IPCWu_spline,replace) title(IPCW analysis for OS) ///

> * xlabel(#8) risktable(,format(%4.0f)) note(Unstabilised weights)

. stcox trtgrp becogstrat regionstrat

Failure _d: deathtdc

Analysis time _t: dthdyxtdc

Weight: [pweight=weight]

(sum of wgt is 51,963.8479765654)

Iteration 0: log pseudolikelihood = -665.65039

Iteration 1: log pseudolikelihood = -636.53827

Iteration 2: log pseudolikelihood = -632.16918

Iteration 3: log pseudolikelihood = -632.11985

Iteration 4: log pseudolikelihood = -632.11984

Refining estimates:

Iteration 0: log pseudolikelihood = -632.11984

Cox regression with Breslow method for ties

No. of subjects = 51,964 Number of obs = 42,300

No. of failures = 186

Time at risk = 51,963.848

Wald chi2(3) = 58.13

Log pseudolikelihood = -632.11984 Prob > chi2 = 0.0000

------------------------------------------------------------------------------

| Robust

_t | Haz. ratio std. err. z P>|z| [95% conf. interval]

-------------+----------------------------------------------------------------

trtgrp | .4953092 .1429994 -2.43 0.015 .2812731 .8722171

becogstrat | 6.323308 1.730806 6.74 0.000 3.697897 10.81269

regionstrat | .6170005 .135864 -2.19 0.028 .4007286 .9499938

------------------------------------------------------------------------------

.

. restore

.

. *****************************************************************************

> *************************

. *** IPCW 5 (secondary analysis, full model, stabilised weights) Estimand 2 [1

> 29 from IPCW file] ***

. *****************************************************************************

> *************************

.

. preserve

.

. * note, 8 patients switched before investigator observed progression. 7 were

> kras MT, so primary analyses will not adjust for these anyway.

. * protocol suggested switching permitted only after progression. So assume th

> ese patients switched due to some signs of progression.

. * need this, because IPCW models will fit much better if applied only to the

> time-periods where switching was "permitted".

. * so, for these 8 patients, replace PDDYLR to equal xotime

. * and replace progtdc to = 1 after this point for these patients

.

. * estimand 2, with secondary analysis: drop all pani kras MT patients

. drop if krasi==1 & trtgrp==2

(16,589 observations deleted)

.

. sort SUBJID dthdyxtdc

. by SUBJID: replace PDDYLR = xotime if (PDDYLR>xotime & xotime!=.)

(1,252 real changes made)

. by SUBJID: replace progtdc = 1 if (PDDYLR <= dthdyxtdc & PDLR==1)

(984 real changes made)

.

. replace xotdc = 0 if krasi==1 & trtgrp==1

(14,415 real changes made)

. replace xo = . if krasi==1 & trtgrp==1 & xo==1

(18,500 real changes made, 18,500 to missing)

.

. by SUBJID: drop if (dthdyxtdc>xotime & xo==1)

(22,711 observations deleted)

. by SUBJID: replace lastobs = 0

(252 real changes made)

. by SUBJID: replace lastobs = 1 if _n==_N

(343 real changes made)

.

. *** IPCW step 1 with splines

.

. *** Use logistic regression to predict switching given baseline covariates (m

> odel S1)***

. *** First use rcsgen to create splines for a time-dependent interecept***

. *** Generate 6 knots based upon the event time distribution (can try other am

> ounts of knots)***

. rcsgen dthdyxtdc, df(5) if2(xotdc==1) gen(timexosp)

Variables timexosp1 to timexosp5 were created

. * where are the knots?

. di r(knots)

3 43 60 68 78 335

. logistic xotdc becogstrat regionstrat timexosp* if trtgrp==1

Logistic regression Number of obs = 29,138

LR chi2(7) = 117.99

Prob > chi2 = 0.0000

Log likelihood = -556.8356 Pseudo R2 = 0.0958

------------------------------------------------------------------------------

xotdc | Odds ratio Std. err. z P>|z| [95% conf. interval]

-------------+----------------------------------------------------------------

becogstrat | .887737 .3529883 -0.30 0.765 .4072148 1.935286

regionstrat | .8924505 .1394269 -0.73 0.466 .6570547 1.212179

timexosp1 | 1.002154 .0290382 0.07 0.941 .946826 1.060715

timexosp2 | .999943 .0000884 -0.64 0.519 .9997697 1.000116

timexosp3 | .999838 .0003104 -0.52 0.602 .9992299 1.000447

timexosp4 | 1.000414 .0003231 1.28 0.200 .9997809 1.001047

timexosp5 | .9997936 .0000908 -2.27 0.023 .9996157 .9999716

_cons | .0017268 .0010132 -10.84 0.000 .0005468 .0054538

------------------------------------------------------------------------------

Note: _cons estimates baseline odds.

.

. *** assess fit of the model, for comparison to alternatives that may be run l

> ater***

. estat gof, group(10) table

note: obs collapsed on 10 quantiles of estimated probabilities.

Goodness-of-fit test after logistic model

Variable: xotdc

Table collapsed on quantiles of estimated probabilities

+---------------------------------------------------------+

| Group | Prob | Obs_1 | Exp_1 | Obs_0 | Exp_0 | Total |

|-------+--------+-------+-------+-------+--------+-------|

| 1 | 0.0002 | 1 | 0.5 | 2925 | 2925.5 | 2926 |

| 2 | 0.0002 | 1 | 0.5 | 2901 | 2901.5 | 2902 |

| 3 | 0.0004 | 1 | 0.8 | 2917 | 2917.2 | 2918 |

| 4 | 0.0014 | 2 | 2.5 | 2925 | 2924.5 | 2927 |

| 5 | 0.0018 | 5 | 4.7 | 2892 | 2892.3 | 2897 |

|-------+--------+-------+-------+-------+--------+-------|

| 6 | 0.0020 | 7 | 5.5 | 2936 | 2937.5 | 2943 |

| 7 | 0.0029 | 8 | 7.2 | 2994 | 2994.8 | 3002 |

| 8 | 0.0050 | 6 | 10.8 | 2802 | 2797.2 | 2808 |

| 9 | 0.0096 | 16 | 20.9 | 2923 | 2918.1 | 2939 |

| 10 | 0.0161 | 44 | 37.7 | 2832 | 2838.3 | 2876 |

+---------------------------------------------------------+

Number of observations = 29,138

Number of groups = 10

Hosmer–Lemeshow chi2(8) = 6.07

Prob > chi2 = 0.6390

. estat ic

Akaike's information criterion and Bayesian information criterion

-----------------------------------------------------------------------------

Model | N ll(null) ll(model) df AIC BIC

-------------+---------------------------------------------------------------

. | 29,138 -615.8312 -556.8356 8 1129.671 1195.91

-----------------------------------------------------------------------------

Note: BIC uses N = number of observations. See [R] BIC note.

.

. *** Estimate the probability of switching for each patient-observation includ

> ed in the regression.***

. predict pxo1 if e(sample), pr

(33,379 missing values generated)

.

. *** Use logistic regression to predict switching given baseline and time-upda

> ted covariates (model S12)***

. logistic xotdc AGE becogstrat diagtype BILIULN ASTULN CREATULN ALBULN LDHULN

> eq5dbase CEAULN eq5dmissb regionstrat PDDYLR eq5dtdc eq5dind eq5dmisslastvisi

> t ecogtdc bestresptdc respmisslastvisit LSSLDtdc LSSLDmisslastvisit AATULNtdc

> AATmisslastvisit ALBULNtdc ALBmisslastvisit ALKULNtdc ASTULNtdc ASTmisslastv

> isit CEAULNtdc CEAmisslastvisit CREATmisslastvisit LDHULNtdc LDHmisslastvisit

> BILIULNtdc BILImisslastvisit saetdc timexosp* if trtgrp==1 & progtdc>0

note: ALBmisslastvisit omitted because of collinearity.

note: ASTmisslastvisit omitted because of collinearity.

note: CREATmisslastvisit omitted because of collinearity.

note: LDHmisslastvisit omitted because of collinearity.

note: BILImisslastvisit omitted because of collinearity.

Logistic regression Number of obs = 18,272

LR chi2(36) = 291.54

Prob > chi2 = 0.0000

Log likelihood = -427.50728 Pseudo R2 = 0.2543

-------------------------------------------------------------------------------

xotdc | Odds ratio Std. err. z P>|z| [95% conf. interval]

--------------+----------------------------------------------------------------

AGE | .9843539 .0115378 -1.35 0.178 .9619981 1.007229

becogstrat | .694304 .347329 -0.73 0.466 .2604545 1.850834

diagtype | 1.000881 .2564561 0.00 0.997 .6057296 1.653812

BILIULN | 1.109153 .4369996 0.26 0.793 .5124163 2.40082

ASTULN | .5263829 .1908003 -1.77 0.077 .2586801 1.071126

CREATULN | .3448927 .3821792 -0.96 0.337 .039306 3.026281

ALBULN | 1.075858 .4553094 0.17 0.863 .4693765 2.465973

LDHULN | .9448671 .3446201 -0.16 0.876 .4622886 1.931205

eq5dbase | .5910094 .3552164 -0.88 0.382 .1819675 1.91953

CEAULN | .6513831 .2398149 -1.16 0.244 .31656 1.340346

eq5dmissb | 1.409686 1.107263 0.44 0.662 .3023644 6.57225

regionstrat | .9583462 .1633606 -0.25 0.803 .6861606 1.338502

PDDYLR | 1.029893 .0102458 2.96 0.003 1.010006 1.050171

eq5dtdc | 1.545653 .891714 0.75 0.450 .4989291 4.78834

eq5dind | 1.18072 1.102794 0.18 0.859 .1892881 7.364959

eq5dmisslas~t | .6447222 .2159288 -1.31 0.190 .3344185 1.242954

ecogtdc | .7605728 .150072 -1.39 0.165 .5166381 1.119683

bestresptdc | .8769905 .1850692 -0.62 0.534 .5799197 1.326239

respmisslas~t | 1.337101 .4201647 0.92 0.355 .7222483 2.475381

LSSLDtdc | .9995649 .0011004 -0.40 0.693 .9974105 1.001724

LSSLDmissla~t | .3757118 .1681304 -2.19 0.029 .1562945 .9031631

AATULNtdc | .970393 .2931235 -0.10 0.921 .5368191 1.754152

AATmisslast~t | .355137 .2396706 -1.53 0.125 .0946122 1.333045

ALBULNtdc | 1.376808 .4322348 1.02 0.308 .7441276 2.547414

ALBmisslast~t | 1 (omitted)

ALKULNtdc | 1.403437 .4952429 0.96 0.337 .7027841 2.802617

ASTULNtdc | 2.432125 .9427086 2.29 0.022 1.13777 5.198973

ASTmisslast~t | 1 (omitted)

CEAULNtdc | 1.43079 .8116244 0.63 0.528 .4706808 4.349358

CEAmisslast~t | .386767 .1585071 -2.32 0.020 .1732228 .8635623

CREATmissla~t | 1 (omitted)

LDHULNtdc | 1.526699 .5815109 1.11 0.267 .723662 3.220855

LDHmisslast~t | 1 (omitted)

BILIULNtdc | .6045854 .2198178 -1.38 0.166 .2964654 1.232938

BILImisslas~t | 1 (omitted)

saetdc | .2633589 .2730271 -1.29 0.198 .0345222 2.009084

timexosp1 | .9230061 .0381435 -1.94 0.053 .8511938 1.000877

timexosp2 | 1.000023 .0001049 0.22 0.828 .9998173 1.000228

timexosp3 | .9995718 .0003522 -1.22 0.224 .9988818 1.000262

timexosp4 | 1.000614 .000364 1.69 0.092 .9999007 1.001327

timexosp5 | .9997809 .0001029 -2.13 0.033 .9995793 .9999825

_cons | .2255134 .381266 -0.88 0.378 .0082051 6.198162

-------------------------------------------------------------------------------

Note: _cons estimates baseline odds.

Note: 348 failures and 0 successes completely determined.

. * Note, had to take ecogmisslastvisit out as !=0 perfectly predicted failure,

> and ALKmisslastvisit because missing SE hidden colinearity. Also removed CRE

> ATULNtdc as coefficient and SE crazy.

.

. *** Assess model fit

. estat gof, group(10) table

note: obs collapsed on 10 quantiles of estimated probabilities.

Goodness-of-fit test after logistic model

Variable: xotdc

Table collapsed on quantiles of estimated probabilities

+---------------------------------------------------------+

| Group | Prob | Obs_1 | Exp_1 | Obs_0 | Exp_0 | Total |

|-------+--------+-------+-------+-------+--------+-------|

| 1 | 0.0000 | 0 | 0.0 | 1828 | 1828.0 | 1828 |

| 2 | 0.0000 | 0 | 0.0 | 1827 | 1827.0 | 1827 |

| 3 | 0.0000 | 0 | 0.0 | 1827 | 1827.0 | 1827 |

| 4 | 0.0000 | 0 | 0.0 | 1827 | 1827.0 | 1827 |

| 5 | 0.0001 | 0 | 0.1 | 1827 | 1826.9 | 1827 |

|-------+--------+-------+-------+-------+--------+-------|

| 6 | 0.0003 | 0 | 0.4 | 1828 | 1827.6 | 1828 |

| 7 | 0.0011 | 2 | 1.1 | 1825 | 1825.9 | 1827 |

| 8 | 0.0053 | 5 | 5.1 | 1822 | 1821.9 | 1827 |

| 9 | 0.0178 | 24 | 19.0 | 1803 | 1808.0 | 1827 |

| 10 | 0.1651 | 60 | 65.2 | 1767 | 1761.8 | 1827 |

+---------------------------------------------------------+

Number of observations = 18,272

Number of groups = 10

Hosmer–Lemeshow chi2(8) = 2.95

Prob > chi2 = 0.9377

. estat ic

Akaike's information criterion and Bayesian information criterion

-----------------------------------------------------------------------------

Model | N ll(null) ll(model) df AIC BIC

-------------+---------------------------------------------------------------

. | 18,272 -573.2792 -427.5073 37 929.0146 1218.1

-----------------------------------------------------------------------------

Note: BIC uses N = number of observations. See [R] BIC note.

.

. *** Estimate the probability of switching for each patient-observation includ

> ed in the regression.***

. predict pxo2 if e(sample), pr

(44,245 missing values generated)

.

. *** IPCW step 3 with splines

.

. *** Estimate the probabilities of remaining 'un-switched' and hence the weigh

> ts ***

. sort SUBJID dthdyxtdc

. replace pxo1=pxo1*xotdc+(1-pxo1)*(1-xotdc)

(29,047 real changes made)

. replace pxo1=1 if pxo1==.

(33,379 real changes made)

. **Now we estimate each individual's probability of their complete censoring h

> istory up to each day**

. sort SUBJID dthdyxtdc

. by SUBJID: replace pxo1=pxo1*pxo1[_n-1] if _n!=1

(28919 real changes made)

. rename pxo1 num

.

. replace pxo2=pxo2*xotdc+(1-pxo2)*(1-xotdc)

(18,181 real changes made)

. replace pxo2=1 if pxo2==.

(44,245 real changes made)

. sort SUBJID dthdyxtdc

. by SUBJID: replace pxo2 = pxo2*pxo2[_n-1] if _n!=1

(18057 real changes made)

. rename pxo2 denom

.

. gen weight = 1 / denom if trtgrp==1

(33,379 missing values generated)

. gen sweight = num / denom if trtgrp==1

(33,379 missing values generated)

.

. *** set the weights to 1 in the treatment arm and if kras==1.

. replace weight = 1 if trtgrp==2

(33,379 real changes made)

. replace sweight = 1 if trtgrp==2

(33,379 real changes made)

.

. *** summarise the weights, and inspect the data to be confident that you have

> computed the weights correctly.

. summ sweight if xotdc==0

Variable | Obs Mean Std. dev. Min Max

-------------+---------------------------------------------------------

sweight | 62,426 .979311 .2323947 .4666828 5.660593

.

. *** IPCW step 4 with splines

.

. * Stabilised weights

. stset dthdyxtdc deathtdc if xotdc==0 [pw=sweight], time0(time)

Survival-time data settings

Failure event: deathtdc!=0 & deathtdc<.

Observed time interval: (time, dthdyxtdc]

Exit on or before: failure

Weight: [pweight=sweight]

Keep observations

if exp: xotdc==0

--------------------------------------------------------------------------

62,517 total observations

91 ignored at outset because of if exp

--------------------------------------------------------------------------

62,426 observations remaining, representing

227 failures in single-record/single-failure data

62,426 total analysis time at risk and under observation

At risk from t = 0

Earliest observed entry t = 0

Last observed exit t = 1,024

. * sts, by(trt) name(IPCWs_spline,replace) title(IPCW analysis for OS) ///

> * xlabel(#8) risktable(,format(%4.0f)) note(Stabilised weights)

. stcox trtgrp becogstrat regionstrat

Failure _d: deathtdc

Analysis time _t: dthdyxtdc

Weight: [pweight=sweight]

(sum of wgt is 61,134.4666788876)

Iteration 0: log pseudolikelihood = -1102.2213

Iteration 1: log pseudolikelihood = -1098.9701

Iteration 2: log pseudolikelihood = -1069.9724

Iteration 3: log pseudolikelihood = -1069.9272

Iteration 4: log pseudolikelihood = -1069.9272

Refining estimates:

Iteration 0: log pseudolikelihood = -1069.9272

Cox regression with Breslow method for ties

No. of subjects = 61,134 Number of obs = 62,426

No. of failures = 227

Time at risk = 61,134.4667

Wald chi2(3) = 80.40

Log pseudolikelihood = -1069.9272 Prob > chi2 = 0.0000

------------------------------------------------------------------------------

| Robust

_t | Haz. ratio std. err. z P>|z| [95% conf. interval]

-------------+----------------------------------------------------------------

trtgrp | .6505698 .0897336 -3.12 0.002 .4964631 .8525125

becogstrat | 4.945608 .94653 8.35 0.000 3.398678 7.196635

regionstrat | .9526334 .0869546 -0.53 0.595 .7965807 1.139257

------------------------------------------------------------------------------

.

. *****************************************************************************

> *************************

. *** IPCW 6 (secondary analysis, full model, unstabilised weights) Estimand 2

> [130 from IPCW file] ***

. *****************************************************************************

> *************************

.

. summ weight if xotdc==0

Variable | Obs Mean Std. dev. Min Max

-------------+---------------------------------------------------------

weight | 62,426 1.230332 .5711998 1 8.310938

.

. stset dthdyxtdc deathtdc if xotdc==0 [pw=weight], time0(time)

Survival-time data settings

Failure event: deathtdc!=0 & deathtdc<.

Observed time interval: (time, dthdyxtdc]

Exit on or before: failure

Weight: [pweight=weight]

Keep observations

if exp: xotdc==0

--------------------------------------------------------------------------

62,517 total observations

91 ignored at outset because of if exp

--------------------------------------------------------------------------

62,426 observations remaining, representing

227 failures in single-record/single-failure data

62,426 total analysis time at risk and under observation

At risk from t = 0

Earliest observed entry t = 0

Last observed exit t = 1,024

. * sts, by(trt) name(IPCWu_spline,replace) title(IPCW analysis for OS) ///

> * xlabel(#8) risktable(,format(%4.0f)) note(Unstabilised weights)

. stcox trtgrp becogstrat regionstrat

Failure _d: deathtdc

Analysis time _t: dthdyxtdc

Weight: [pweight=weight]

(sum of wgt is 76,804.7075513601)

Iteration 0: log pseudolikelihood = -1199.9978

Iteration 1: log pseudolikelihood = -1196.5013

Iteration 2: log pseudolikelihood = -1170.9845

Iteration 3: log pseudolikelihood = -1170.7765

Iteration 4: log pseudolikelihood = -1170.7763

Refining estimates:

Iteration 0: log pseudolikelihood = -1170.7763

Cox regression with Breslow method for ties

No. of subjects = 76,805 Number of obs = 62,426

No. of failures = 311

Time at risk = 76,804.7076

Wald chi2(3) = 73.43

Log pseudolikelihood = -1170.7763 Prob > chi2 = 0.0000

------------------------------------------------------------------------------

| Robust

_t | Haz. ratio std. err. z P>|z| [95% conf. interval]

-------------+----------------------------------------------------------------

trtgrp | .6602225 .0905502 -3.03 0.002 .5045998 .8638405

becogstrat | 4.611379 .905209 7.79 0.000 3.138635 6.77518

regionstrat | .9361438 .0912044 -0.68 0.498 .773417 1.133108

------------------------------------------------------------------------------

.

. restore

.

. *****************************************************************************

> *************************

. *** IPCW 7 (secondary analysis, reduced model, stabilised weights) Estimand 2

> [131 from IPCW file] ***

. *****************************************************************************

> *************************

.

. preserve

.

. * note, 8 patients switched before investigator observed progression. 7 were

> kras MT, so primary analyses will not adjust for these anyway.

. * protocol suggested switching permitted only after progression. So assume th

> ese patients switched due to some signs of progression.

. * need this, because IPCW models will fit much better if applied only to the

> time-periods where switching was "permitted".

. * so, for these 8 patients, replace PDDYLR to equal xotime

. * and replace progtdc to = 1 after this point for these patients

.

. * estimand 2, with secondary analysis: drop all pani kras MT patients

. drop if krasi==1 & trtgrp==2

(16,589 observations deleted)

.

. sort SUBJID dthdyxtdc

. by SUBJID: replace PDDYLR = xotime if (PDDYLR>xotime & xotime!=.)

(1,252 real changes made)

. by SUBJID: replace progtdc = 1 if (PDDYLR <= dthdyxtdc & PDLR==1)

(984 real changes made)

.

. replace xotdc = 0 if krasi==1 & trtgrp==1

(14,415 real changes made)

. replace xo = . if krasi==1 & trtgrp==1 & xo==1

(18,500 real changes made, 18,500 to missing)

.

. by SUBJID: drop if (dthdyxtdc>xotime & xo==1)

(22,711 observations deleted)

. by SUBJID: replace lastobs = 0

(252 real changes made)

. by SUBJID: replace lastobs = 1 if _n==_N

(343 real changes made)

.

. *** IPCW step 1 with splines

.

. *** Use logistic regression to predict switching given baseline covariates (m

> odel S1)***

. *** First use rcsgen to create splines for a time-dependent interecept***

. *** Generate 6 knots based upon the event time distribution (can try other am

> ounts of knots)***

. rcsgen dthdyxtdc, df(5) if2(xotdc==1) gen(timexosp)

Variables timexosp1 to timexosp5 were created

. * where are the knots?

. di r(knots)

3 43 60 68 78 335

. logistic xotdc becogstrat regionstrat timexosp* if trtgrp==1

Logistic regression Number of obs = 29,138

LR chi2(7) = 117.99

Prob > chi2 = 0.0000

Log likelihood = -556.8356 Pseudo R2 = 0.0958

------------------------------------------------------------------------------

xotdc | Odds ratio Std. err. z P>|z| [95% conf. interval]

-------------+----------------------------------------------------------------

becogstrat | .887737 .3529883 -0.30 0.765 .4072148 1.935286

regionstrat | .8924505 .1394269 -0.73 0.466 .6570547 1.212179

timexosp1 | 1.002154 .0290382 0.07 0.941 .946826 1.060715

timexosp2 | .999943 .0000884 -0.64 0.519 .9997697 1.000116

timexosp3 | .999838 .0003104 -0.52 0.602 .9992299 1.000447

timexosp4 | 1.000414 .0003231 1.28 0.200 .9997809 1.001047

timexosp5 | .9997936 .0000908 -2.27 0.023 .9996157 .9999716

_cons | .0017268 .0010132 -10.84 0.000 .0005468 .0054538

------------------------------------------------------------------------------

Note: _cons estimates baseline odds.

.

. *** assess fit of the model, for comparison to alternatives that may be run l

> ater***

. estat gof, group(10) table

note: obs collapsed on 10 quantiles of estimated probabilities.

Goodness-of-fit test after logistic model

Variable: xotdc

Table collapsed on quantiles of estimated probabilities

+---------------------------------------------------------+

| Group | Prob | Obs_1 | Exp_1 | Obs_0 | Exp_0 | Total |

|-------+--------+-------+-------+-------+--------+-------|

| 1 | 0.0002 | 1 | 0.5 | 2925 | 2925.5 | 2926 |

| 2 | 0.0002 | 1 | 0.5 | 2901 | 2901.5 | 2902 |

| 3 | 0.0004 | 1 | 0.8 | 2917 | 2917.2 | 2918 |

| 4 | 0.0014 | 2 | 2.5 | 2925 | 2924.5 | 2927 |

| 5 | 0.0018 | 5 | 4.7 | 2892 | 2892.3 | 2897 |

|-------+--------+-------+-------+-------+--------+-------|

| 6 | 0.0020 | 7 | 5.5 | 2936 | 2937.5 | 2943 |

| 7 | 0.0029 | 8 | 7.2 | 2994 | 2994.8 | 3002 |

| 8 | 0.0050 | 6 | 10.8 | 2802 | 2797.2 | 2808 |

| 9 | 0.0096 | 16 | 20.9 | 2923 | 2918.1 | 2939 |

| 10 | 0.0161 | 44 | 37.7 | 2832 | 2838.3 | 2876 |

+---------------------------------------------------------+

Number of observations = 29,138

Number of groups = 10

Hosmer–Lemeshow chi2(8) = 6.07

Prob > chi2 = 0.6390

. estat ic

Akaike's information criterion and Bayesian information criterion

-----------------------------------------------------------------------------

Model | N ll(null) ll(model) df AIC BIC

-------------+---------------------------------------------------------------

. | 29,138 -615.8312 -556.8356 8 1129.671 1195.91

-----------------------------------------------------------------------------

Note: BIC uses N = number of observations. See [R] BIC note.

.

. *** Estimate the probability of switching for each patient-observation includ

> ed in the regression.***

. predict pxo1 if e(sample), pr

(33,379 missing values generated)

.

. *** Use logistic regression to predict switching given baseline and time-upda

> ted covariates (model S12)***

. logistic xotdc becogstrat diagtype eq5dbase eq5dmissb regionstrat PDDYLR eq5d

> tdc eq5dind eq5dmisslastvisit ecogtdc bestresptdc respmisslastvisit LSSLDtdc

> LSSLDmisslastvisit timexosp* if trtgrp==1 & progtdc>0

Logistic regression Number of obs = 18,272

LR chi2(19) = 251.77

Prob > chi2 = 0.0000

Log likelihood = -447.39258 Pseudo R2 = 0.2196

-------------------------------------------------------------------------------

xotdc | Odds ratio Std. err. z P>|z| [95% conf. interval]

--------------+----------------------------------------------------------------

becogstrat | .6017097 .2823666 -1.08 0.279 .2398504 1.509502

diagtype | 1.049851 .2505912 0.20 0.839 .657586 1.676111

eq5dbase | .6141219 .3243276 -0.92 0.356 .2181331 1.728971

eq5dmissb | 1.291905 .9712174 0.34 0.733 .2960202 5.63819

regionstrat | .9549368 .1534423 -0.29 0.774 .6969487 1.308424

PDDYLR | 1.031388 .009716 3.28 0.001 1.01252 1.050608

eq5dtdc | 1.845916 .9838002 1.15 0.250 .6494642 5.246488

eq5dind | .9603732 .8573719 -0.05 0.964 .1669287 5.525214

eq5dmisslas~t | .4272395 .1374074 -2.64 0.008 .2274629 .8024761

ecogtdc | .9171834 .1602372 -0.49 0.621 .6512479 1.291713

bestresptdc | .850841 .1679782 -0.82 0.413 .5778286 1.252846

respmisslas~t | 1.244165 .3774427 0.72 0.471 .686513 2.254796

LSSLDtdc | 1.00058 .0008736 0.66 0.506 .9988695 1.002294

LSSLDmissla~t | .2826981 .1258291 -2.84 0.005 .1181551 .676384

timexosp1 | .8917623 .0337703 -3.03 0.002 .8279705 .960469

timexosp2 | .9999914 .0000986 -0.09 0.931 .9997982 1.000185

timexosp3 | .9995847 .0003351 -1.24 0.215 .998928 1.000242

timexosp4 | 1.000648 .000348 1.86 0.063 .9999661 1.00133

timexosp5 | .9997582 .0000987 -2.45 0.014 .9995647 .9999517

_cons | .2573698 .3429401 -1.02 0.308 .0188951 3.505634

-------------------------------------------------------------------------------

Note: _cons estimates baseline odds.

Note: 47 failures and 0 successes completely determined.

. * Note, had to take ecogmisslastvisit out as !=0 perfectly predicted failure.

.

. *** Assess model fit

. estat gof, group(10) table

note: obs collapsed on 10 quantiles of estimated probabilities.

Goodness-of-fit test after logistic model

Variable: xotdc

Table collapsed on quantiles of estimated probabilities

+---------------------------------------------------------+

| Group | Prob | Obs_1 | Exp_1 | Obs_0 | Exp_0 | Total |

|-------+--------+-------+-------+-------+--------+-------|

| 1 | 0.0000 | 0 | 0.0 | 1828 | 1828.0 | 1828 |

| 2 | 0.0000 | 0 | 0.0 | 1827 | 1827.0 | 1827 |

| 3 | 0.0000 | 0 | 0.0 | 1827 | 1827.0 | 1827 |

| 4 | 0.0001 | 0 | 0.1 | 1827 | 1826.9 | 1827 |

| 5 | 0.0002 | 0 | 0.2 | 1827 | 1826.8 | 1827 |

|-------+--------+-------+-------+-------+--------+-------|

| 6 | 0.0008 | 1 | 0.8 | 1827 | 1827.2 | 1828 |

| 7 | 0.0018 | 1 | 2.2 | 1826 | 1824.8 | 1827 |

| 8 | 0.0072 | 9 | 6.5 | 1818 | 1820.5 | 1827 |

| 9 | 0.0194 | 28 | 23.9 | 1799 | 1803.1 | 1827 |

| 10 | 0.1153 | 52 | 57.3 | 1775 | 1769.7 | 1827 |

+---------------------------------------------------------+

Number of observations = 18,272

Number of groups = 10

Hosmer–Lemeshow chi2(8) = 3.27

Prob > chi2 = 0.9162

. estat ic

Akaike's information criterion and Bayesian information criterion

-----------------------------------------------------------------------------

Model | N ll(null) ll(model) df AIC BIC

-------------+---------------------------------------------------------------

. | 18,272 -573.2792 -447.3926 20 934.7852 1091.048

-----------------------------------------------------------------------------

Note: BIC uses N = number of observations. See [R] BIC note.

.

. *** Estimate the probability of switching for each patient-observation includ

> ed in the regression.***

. predict pxo2 if e(sample), pr

(44,245 missing values generated)

.

. *** IPCW step 3 with splines

.

. *** Estimate the probabilities of remaining 'un-switched' and hence the weigh

> ts ***

. sort SUBJID dthdyxtdc

. replace pxo1=pxo1*xotdc+(1-pxo1)*(1-xotdc)

(29,047 real changes made)

. replace pxo1=1 if pxo1==.

(33,379 real changes made)

. **Now we estimate each individual's probability of their complete censoring h

> istory up to each day**

. sort SUBJID dthdyxtdc

. by SUBJID: replace pxo1=pxo1*pxo1[_n-1] if _n!=1

(28919 real changes made)

. rename pxo1 num

.

. replace pxo2=pxo2*xotdc+(1-pxo2)*(1-xotdc)

(18,181 real changes made)

. replace pxo2=1 if pxo2==.

(44,245 real changes made)

. sort SUBJID dthdyxtdc

. by SUBJID: replace pxo2 = pxo2*pxo2[_n-1] if _n!=1

(18057 real changes made)

. rename pxo2 denom

.

. gen weight = 1 / denom if trtgrp==1

(33,379 missing values generated)

. gen sweight = num / denom if trtgrp==1

(33,379 missing values generated)

.

. *** set the weights to 1 in the treatment arm and if kras==1.

. replace weight = 1 if trtgrp==2

(33,379 real changes made)

. replace sweight = 1 if trtgrp==2

(33,379 real changes made)

.

. *** summarise the weights, and inspect the data to be confident that you have

> computed the weights correctly.

. summ sweight if xotdc==0

Variable | Obs Mean Std. dev. Min Max

-------------+---------------------------------------------------------

sweight | 62,426 .9908243 .1781228 .4666828 2.397694

.

. *** IPCW step 4 with splines

.

. * Stabilised weights

. stset dthdyxtdc deathtdc if xotdc==0 [pw=sweight], time0(time)

Survival-time data settings

Failure event: deathtdc!=0 & deathtdc<.

Observed time interval: (time, dthdyxtdc]

Exit on or before: failure

Weight: [pweight=sweight]

Keep observations

if exp: xotdc==0

--------------------------------------------------------------------------

62,517 total observations

91 ignored at outset because of if exp

--------------------------------------------------------------------------

62,426 observations remaining, representing

227 failures in single-record/single-failure data

62,426 total analysis time at risk and under observation

At risk from t = 0

Earliest observed entry t = 0

Last observed exit t = 1,024

. * sts, by(trt) name(IPCWs_spline,replace) title(IPCW analysis for OS) ///

> * xlabel(#8) risktable(,format(%4.0f)) note(Stabilised weights)

. stcox trtgrp becogstrat regionstrat

Failure _d: deathtdc

Analysis time _t: dthdyxtdc

Weight: [pweight=sweight]

(sum of wgt is 61,853.1963531971)

Iteration 0: log pseudolikelihood = -1083.1976

Iteration 1: log pseudolikelihood = -1082.1357

Iteration 2: log pseudolikelihood = -1051.5792

Iteration 3: log pseudolikelihood = -1051.5574

Iteration 4: log pseudolikelihood = -1051.5574

Refining estimates:

Iteration 0: log pseudolikelihood = -1051.5574

Cox regression with Breslow method for ties

No. of subjects = 61,853 Number of obs = 62,426

No. of failures = 226

Time at risk = 61,853.1964

Wald chi2(3) = 79.90

Log pseudolikelihood = -1051.5574 Prob > chi2 = 0.0000

------------------------------------------------------------------------------

| Robust

_t | Haz. ratio std. err. z P>|z| [95% conf. interval]

-------------+----------------------------------------------------------------

trtgrp | .6900808 .0929086 -2.76 0.006 .5300282 .8984647

becogstrat | 5.042411 .957194 8.52 0.000 3.475809 7.315107

regionstrat | .9506378 .0856569 -0.56 0.574 .7967423 1.134259

------------------------------------------------------------------------------

.

. *****************************************************************************

> *************************

. *** IPCW 8 (secondary analysis, reduced model, unstabilised weights) Estimand

> 2 [132 from IPCW file] ***

. *****************************************************************************

> *************************

.

. summ weight if xotdc==0

Variable | Obs Mean Std. dev. Min Max

-------------+---------------------------------------------------------

weight | 62,426 1.252654 .5161055 1 4.192906

.

. stset dthdyxtdc deathtdc if xotdc==0 [pw=weight], time0(time)

Survival-time data settings

Failure event: deathtdc!=0 & deathtdc<.

Observed time interval: (time, dthdyxtdc]

Exit on or before: failure

Weight: [pweight=weight]

Keep observations

if exp: xotdc==0

--------------------------------------------------------------------------

62,517 total observations

91 ignored at outset because of if exp

--------------------------------------------------------------------------

62,426 observations remaining, representing

227 failures in single-record/single-failure data

62,426 total analysis time at risk and under observation

At risk from t = 0

Earliest observed entry t = 0

Last observed exit t = 1,024

. * sts, by(trt) name(IPCWu_spline,replace) title(IPCW analysis for OS) ///

> * xlabel(#8) risktable(,format(%4.0f)) note(Unstabilised weights)

. stcox trtgrp becogstrat regionstrat

Failure _d: deathtdc

Analysis time _t: dthdyxtdc

Weight: [pweight=weight]

(sum of wgt is 78,198.1723617315)

Iteration 0: log pseudolikelihood = -1164.5157

Iteration 1: log pseudolikelihood = -1163.638

Iteration 2: log pseudolikelihood = -1136.3945

Iteration 3: log pseudolikelihood = -1136.2251

Iteration 4: log pseudolikelihood = -1136.225

Refining estimates:

Iteration 0: log pseudolikelihood = -1136.225

Cox regression with Breslow method for ties

No. of subjects = 78,198 Number of obs = 62,426

No. of failures = 307

Time at risk = 78,198.1724

Wald chi2(3) = 73.87

Log pseudolikelihood = -1136.225 Prob > chi2 = 0.0000

------------------------------------------------------------------------------

| Robust

_t | Haz. ratio std. err. z P>|z| [95% conf. interval]

-------------+----------------------------------------------------------------

trtgrp | .7015718 .0943104 -2.64 0.008 .5390727 .9130549

becogstrat | 4.717293 .9073516 8.06 0.000 3.235697 6.877299

regionstrat | .9350949 .0889195 -0.71 0.480 .7760929 1.126672

------------------------------------------------------------------------------

.

. restore

.

end of do-file

. log close

name: <unnamed>

log: X:\ScHARR\Users\cm1nrl\Case studies\Amgen\Code\Final IPCW analyses

> for paper.smcl

log type: smcl

closed on: 26 Oct 2022, 18:06:38

**TSEsimp Analyses**

--------------------------------------------------------------------------------------------

name: TSEsimpLog

log: X:\ScHARR\Users\cm1nrl\Case studies\Amgen\Code\Final methods do files for paper

> \Logs\TSEsimp.log

log type: text

opened on: 27 Oct 2022, 10:45:56

. do "X:\ScHARR\Users\cm1nrl\Case studies\Amgen\Code\Final methods do files for paper\All TS

> E analyses for paper estimand 1.do"

. *** TSEsimp PANI ANALYSES FOR PAPER: ESTIMAND 1 ***

.

.

. ******************************************************************************************

> ***************

. *** TSEsimp 1 (primary analysis, full model, with recens, Weibull) Estimand 1 [217 from TS

> Esimp file] ***

. ******************************************************************************************

> ***************

. use "X:\ScHARR\Users\cm1nrl\Case studies\Amgen\Data\Interim merged datasets\tdc_dataset34.

> dta", clear

(TREAT)

.

. preserve

.

. * note, 8 patients switched before investigator observed progression. 7 were kras MT, so p

> rimary analyses will not adjust for these anyway.

. * protocol suggested switching permitted only after progression. So assume these patients

> switched due to some signs of progression.

. * need this, because SNM models will fit much better if applied only to the time-periods w

> here switching was "permitted".

. * so, for these 8 patients, replace PDDYLR to equal xotime

. * and replace progtdc to = 1 after this point for these patients

. sort SUBJID dthdyxtdc

. by SUBJID: replace PDDYLR = xotime if (PDDYLR>xotime & xotime!=.)

(1,252 real changes made)

. by SUBJID: replace progtdc = 1 if (PDDYLR <= dthdyxtdc & PDLR==1)

(984 real changes made)

.

. replace xotdc = 0 if krasi==1 & trtgrp==1

(14,415 real changes made)

. replace xo = . if krasi==1 & trtgrp==1 & xo==1

(18,500 real changes made, 18,500 to missing)

.

. by SUBJID: replace lastobs = 0

(427 real changes made)

. by SUBJID: replace lastobs = 1 if _n==_N

(427 real changes made)

.

. *** Streg

. sort SUBJID dthdyxtdc

. drop if trtgrp==2

(49,968 observations deleted)

. drop if progtdc==0

(10,866 observations deleted)

. * drop anyone who died on same day as progression

. drop if PDDYLR == deathtime

(16 observations deleted)

. * for primary analysis, get treatment effect comparing WT switchers to WT non-switchers, i

> .e. exclude MT

. drop if krasi==1

(16,044 observations deleted)

. by SUBJID: gen obsno = _n

. by SUBJID: gen trtnew = 0

. by SUBJID: replace trtnew = 1 if xo==1 & dthdyxtdc>=xotime

(22802 real changes made)

. by SUBJID: egen minrisk=min(time)

. by SUBJID: replace dthdyxtdc=dthdyxtdc-minrisk

(24923 real changes made)

. by SUBJID: replace xotime=xotime-minrisk

(24117 real changes made)

. by SUBJID: replace time=time-minrisk

(24923 real changes made)

. by SUBJID: replace admin=admin-minrisk

(24923 real changes made)

.

. stset dthdyxtdc, failure(deathtdc) id(SUBJID)

Survival-time data settings

ID variable: SUBJID

Failure event: deathtdc!=0 & deathtdc<.

Observed time interval: (dthdyxtdc[_n-1], dthdyxtdc]

Exit on or before: failure

--------------------------------------------------------------------------

24,923 total observations

0 exclusions

--------------------------------------------------------------------------

24,923 observations remaining, representing

106 subjects

100 failures in single-failure-per-subject data

24,923 total analysis time at risk and under observation

At risk from t = 0

Earliest observed entry t = 0

Last observed exit t = 784

. streg trtnew AGE becogstrat diagtype BILIULN ASTULN CREATULN ALBULN LDHULN eq5dbase CEAULN

> eq5dmissb regionstrat PDDYLR eq5datprog eq5dind eq5dmissingatprog ecogatprog bestrespatpr

> og respmissingatprog LSSLDatprog LSSLDmissingatprog AATULNatprog AATmissingatprog ALBULNat

> prog ALBmissingatprog ALKULNatprog ALKmissingatprog ASTULNatprog ASTmissingatprog CEAULNat

> prog CEAmissingatprog CREATULNatprog CREATmissingatprog LDHULNatprog LDHmissingatprog BILI

> ULNatprog BILImissingatprog saeatprog, dist(weibull) time iterate(200)

Failure _d: deathtdc

Analysis time _t: dthdyxtdc

ID variable: SUBJID

note: ALBmissingatprog omitted because of collinearity.

note: ALKmissingatprog omitted because of collinearity.

note: ASTmissingatprog omitted because of collinearity.

note: CREATULNatprog omitted because of collinearity.

note: CREATmissingatprog omitted because of collinearity.

note: LDHmissingatprog omitted because of collinearity.

note: BILImissingatprog omitted because of collinearity.

Fitting constant-only model:

Iteration 0: log likelihood = -155.00396

Iteration 1: log likelihood = -153.4072

Iteration 2: log likelihood = -153.40372

Iteration 3: log likelihood = -153.40372

Fitting full model:

Iteration 0: log likelihood = -153.40372

Iteration 1: log likelihood = -121.70482

Iteration 2: log likelihood = -109.88398

Iteration 3: log likelihood = -108.2494

Iteration 4: log likelihood = -108.22721

Iteration 5: log likelihood = -108.22719

Iteration 6: log likelihood = -108.22719

Weibull AFT regression

No. of subjects = 106 Number of obs = 24,923

No. of failures = 100

Time at risk = 24,923

LR chi2(32) = 90.35

Log likelihood = -108.22719 Prob > chi2 = 0.0000

------------------------------------------------------------------------------------

_t | Coefficient Std. err. z P>|z| [95% conf. interval]

-------------------+----------------------------------------------------------------

trtnew | .7925562 .216738 3.66 0.000 .3677574 1.217355

AGE | -.0048295 .0070864 -0.68 0.496 -.0187185 .0090596

becogstrat | .5230229 .2813373 1.86 0.063 -.028388 1.074434

diagtype | .2206367 .1607451 1.37 0.170 -.094418 .5356914

BILIULN | .0355378 .2173681 0.16 0.870 -.3904958 .4615715

ASTULN | .573513 .2583067 2.22 0.026 .0672413 1.079785

CREATULN | -1.204197 .4134357 -2.91 0.004 -2.014516 -.3938775

ALBULN | -.4468884 .3860208 -1.16 0.247 -1.203475 .3096984

LDHULN | .3112406 .2954532 1.05 0.292 -.267837 .8903183

eq5dbase | -.8261484 .3899927 -2.12 0.034 -1.59052 -.0617767

CEAULN | -.2930274 .5818143 -0.50 0.615 -1.433363 .8473077

eq5dmissb | -.2718747 .4762672 -0.57 0.568 -1.205341 .6615919

regionstrat | .0517618 .1067807 0.48 0.628 -.1575245 .2610481

PDDYLR | .0138439 .0040986 3.38 0.001 .0058107 .0218771

eq5datprog | .0695035 .3785514 0.18 0.854 -.6724436 .8114505

eq5dind | .1851247 .6082364 0.30 0.761 -1.006997 1.377246

eq5dmissingatprog | -.0166687 .3018315 -0.06 0.956 -.6082476 .5749102

ecogatprog | -.4862551 .1328997 -3.66 0.000 -.7467337 -.2257765

bestrespatprog | .2345417 .1538745 1.52 0.127 -.0670468 .5361302

respmissingatprog | -.5821665 .2255813 -2.58 0.010 -1.024298 -.1400353

LSSLDatprog | -.0019712 .0007874 -2.50 0.012 -.0035146 -.0004278

LSSLDmissingatprog | -.1649233 .1740925 -0.95 0.343 -.5061384 .1762917

AATULNatprog | -.0258887 .1727636 -0.15 0.881 -.3644992 .3127218

AATmissingatprog | -.4600322 .2435557 -1.89 0.059 -.9373926 .0173282

ALBULNatprog | -.1491619 .3829531 -0.39 0.697 -.8997361 .6014123

ALBmissingatprog | 0 (omitted)

ALKULNatprog | -.1218627 .2242434 -0.54 0.587 -.5613717 .3176463

ALKmissingatprog | 0 (omitted)

ASTULNatprog | -.7328966 .258833 -2.83 0.005 -1.2402 -.2255933

ASTmissingatprog | 0 (omitted)

CEAULNatprog | .3808302 .5993283 0.64 0.525 -.7938317 1.555492

CEAmissingatprog | -.3042879 .2144844 -1.42 0.156 -.7246697 .1160938

CREATULNatprog | 0 (omitted)

CREATmissingatprog | 0 (omitted)

LDHULNatprog | .238702 .3197934 0.75 0.455 -.3880815 .8654855

LDHmissingatprog | 0 (omitted)

BILIULNatprog | -.595671 .2202507 -2.70 0.007 -1.027354 -.1639875

BILImissingatprog | 0 (omitted)

saeatprog | 1.303075 .6652494 1.96 0.050 -.0007896 2.60694

_cons | 5.040682 1.000408 5.04 0.000 3.079918 7.001445

-------------------+----------------------------------------------------------------

/ln_p | .6606433 .0842989 7.84 0.000 .4954204 .8258662

-------------------+----------------------------------------------------------------

p | 1.936037 .1632059 1.641188 2.283858

1/p | .5165189 .043542 .4378556 .6093147

------------------------------------------------------------------------------------

. * Same model as for IPCW denominator but without the TDC variables. Note, had to take ecog

> misslastvisit out as !=0 perfectly predicted failure.

. * And, ALBmisslastvisit, ALKmisslastvisit, ASTmisslastvisit, CREATmisslastvisit, LDHmissla

> stvisit, BILImisslastvisit all omitted because of collinearity

.

. scalar tsec_af = exp(_b[trtnew])

. di tsec_af

2.2090359

.

. restore

. sort SUBJID

. preserve

.

. ***Analysis on overall survival***

. collapse (max) trtgrp krasi xo regionstrat becogstrat dthdyxtdc xotime deathtdc admin, by(

> SUBJID)

. by SUBJID: replace xotime=0 if xotime==.

(259 real changes made)

. by SUBJID: replace xotime = 0 if krasi==1 & trtgrp==1 & xo==1

(77 real changes made)

. by SUBJID: replace xo = 0 if krasi==1 & trtgrp==1 & xo==1

(77 real changes made)

.

. ***below allows for recensoring***

. gen cfact = dthdyxtdc if trtgrp==2

(219 missing values generated)

. gen dcfact = deathtdc if trtgrp==2

(219 missing values generated)

.

. replace cfact = (xotime + ((dthdyxtdc-xotime)/(tsec_af))) if (trtgrp==1 & xotime>0)

(91 real changes made)

. replace cfact = dthdyxtdc if (trtgrp==1 & xotime==0)

(128 real changes made)

. gen OSadminc = admin/(tsec_af) if (trtgrp==1 & (tsec_af)>1.00)

(208 missing values generated)

. replace dcfact = deathtdc if trtgrp==1

(219 real changes made)

. replace dcfact=0 if (OSadminc<=cfact & trtgrp==1)

(11 real changes made)

. replace cfact = OSadminc if (OSadminc<=cfact & trtgrp==1)

(25 real changes made)

.

. ***do survival analysis on re-estimated survival times***

. stset cfact, failure(dcfact) id(SUBJID)

Survival-time data settings

ID variable: SUBJID

Failure event: dcfact!=0 & dcfact<.

Observed time interval: (cfact[_n-1], cfact]

Exit on or before: failure

--------------------------------------------------------------------------

427 total observations

0 exclusions

--------------------------------------------------------------------------

427 observations remaining, representing

427 subjects

380 failures in single-failure-per-subject data

86,180.154 total analysis time at risk and under observation

At risk from t = 0

Earliest observed entry t = 0

Last observed exit t = 1,024

. stcox trtgrp regionstrat becogstrat

Failure _d: dcfact

Analysis time _t: cfact

ID variable: SUBJID

Iteration 0: log likelihood = -1967.0667

Iteration 1: log likelihood = -1949.2442

Iteration 2: log likelihood = -1931.6905

Iteration 3: log likelihood = -1931.3452

Iteration 4: log likelihood = -1931.3449

Refining estimates:

Iteration 0: log likelihood = -1931.3449

Cox regression with Breslow method for ties

No. of subjects = 427 Number of obs = 427

No. of failures = 380

Time at risk = 86,180.1545

LR chi2(3) = 71.44

Log likelihood = -1931.3449 Prob > chi2 = 0.0000

------------------------------------------------------------------------------

_t | Haz. ratio Std. err. z P>|z| [95% conf. interval]

-------------+----------------------------------------------------------------

trtgrp | .6385509 .0686196 -4.17 0.000 .5172782 .7882553

regionstrat | 1.01377 .0708676 0.20 0.845 .8839668 1.162633

becogstrat | 3.478201 .5165495 8.39 0.000 2.599814 4.653365

------------------------------------------------------------------------------

.

. restore

.

. ******************************************************************************************

> ***************

. *** TSEsimp 2 (primary analysis, full model, with recens, lognormal) Estimand 1 ***

. ******************************************************************************************

> ***************

.

. preserve

.

. * note, 8 patients switched before investigator observed progression. 7 were kras MT, so p

> rimary analyses will not adjust for these anyway.

. * protocol suggested switching permitted only after progression. So assume these patients

> switched due to some signs of progression.

. * need this, because SNM models will fit much better if applied only to the time-periods w

> here switching was "permitted".

. * so, for these 8 patients, replace PDDYLR to equal xotime

. * and replace progtdc to = 1 after this point for these patients

. sort SUBJID dthdyxtdc

. by SUBJID: replace PDDYLR = xotime if (PDDYLR>xotime & xotime!=.)

(1,252 real changes made)

. by SUBJID: replace progtdc = 1 if (PDDYLR <= dthdyxtdc & PDLR==1)

(984 real changes made)

.

. replace xotdc = 0 if krasi==1 & trtgrp==1

(14,415 real changes made)

. replace xo = . if krasi==1 & trtgrp==1 & xo==1

(18,500 real changes made, 18,500 to missing)

.

. by SUBJID: replace lastobs = 0

(427 real changes made)

. by SUBJID: replace lastobs = 1 if _n==_N

(427 real changes made)

.

. *** Streg

. sort SUBJID dthdyxtdc

. drop if trtgrp==2

(49,968 observations deleted)

. drop if progtdc==0

(10,866 observations deleted)

. * drop anyone who died on same day as progression

. drop if PDDYLR == deathtime

(16 observations deleted)

. * for primary analysis, get treatment effect comparing WT switchers to WT non-switchers, i

> .e. exclude MT

. drop if krasi==1

(16,044 observations deleted)

. by SUBJID: gen obsno = _n

. by SUBJID: gen trtnew = 0

. by SUBJID: replace trtnew = 1 if xo==1 & dthdyxtdc>=xotime

(22802 real changes made)

. by SUBJID: egen minrisk=min(time)

. by SUBJID: replace dthdyxtdc=dthdyxtdc-minrisk

(24923 real changes made)

. by SUBJID: replace xotime=xotime-minrisk

(24117 real changes made)

. by SUBJID: replace time=time-minrisk

(24923 real changes made)

. by SUBJID: replace admin=admin-minrisk

(24923 real changes made)

.

. stset dthdyxtdc, failure(deathtdc) id(SUBJID)

Survival-time data settings

ID variable: SUBJID

Failure event: deathtdc!=0 & deathtdc<.

Observed time interval: (dthdyxtdc[_n-1], dthdyxtdc]

Exit on or before: failure

--------------------------------------------------------------------------

24,923 total observations

0 exclusions

--------------------------------------------------------------------------

24,923 observations remaining, representing

106 subjects

100 failures in single-failure-per-subject data

24,923 total analysis time at risk and under observation

At risk from t = 0

Earliest observed entry t = 0

Last observed exit t = 784

. streg trtnew AGE becogstrat diagtype BILIULN ASTULN CREATULN ALBULN LDHULN eq5dbase CEAULN

> eq5dmissb regionstrat PDDYLR eq5datprog eq5dind eq5dmissingatprog ecogatprog bestrespatpr

> og respmissingatprog LSSLDatprog LSSLDmissingatprog AATULNatprog AATmissingatprog ALBULNat

> prog ALBmissingatprog ALKULNatprog ALKmissingatprog ASTULNatprog ASTmissingatprog CEAULNat

> prog CEAmissingatprog CREATULNatprog CREATmissingatprog LDHULNatprog LDHmissingatprog BILI

> ULNatprog BILImissingatprog saeatprog, dist(lognormal) time iterate(200)

Failure _d: deathtdc

Analysis time _t: dthdyxtdc

ID variable: SUBJID

note: ALBmissingatprog omitted because of collinearity.

note: ALKmissingatprog omitted because of collinearity.

note: ASTmissingatprog omitted because of collinearity.

note: CREATULNatprog omitted because of collinearity.

note: CREATmissingatprog omitted because of collinearity.

note: LDHmissingatprog omitted because of collinearity.

note: BILImissingatprog omitted because of collinearity.

Fitting constant-only model:

Iteration 0: log likelihood = -222.34803

Iteration 1: log likelihood = -198.01902

Iteration 2: log likelihood = -161.56774

Iteration 3: log likelihood = -160.997

Iteration 4: log likelihood = -160.98957

Iteration 5: log likelihood = -160.98957

Fitting full model:

Iteration 0: log likelihood = -160.98957 (not concave)

Iteration 1: log likelihood = -137.01662

Iteration 2: log likelihood = -121.81787

Iteration 3: log likelihood = -118.32556

Iteration 4: log likelihood = -118.29361

Iteration 5: log likelihood = -118.2936

Lognormal AFT regression

No. of subjects = 106 Number of obs = 24,923

No. of failures = 100

Time at risk = 24,923

LR chi2(32) = 85.39

Log likelihood = -118.2936 Prob > chi2 = 0.0000

------------------------------------------------------------------------------------

_t | Coefficient Std. err. z P>|z| [95% conf. interval]

-------------------+----------------------------------------------------------------

trtnew | .8439232 .224195 3.76 0.000 .404509 1.283337

AGE | -.0028388 .0081509 -0.35 0.728 -.0188142 .0131367

becogstrat | .2509313 .3262883 0.77 0.442 -.388582 .8904446

diagtype | .0355247 .1880266 0.19 0.850 -.3330007 .4040502

BILIULN | .3498442 .273748 1.28 0.201 -.1866921 .8863805

ASTULN | .2321577 .2883656 0.81 0.421 -.3330284 .7973438

CREATULN | -.7470618 .5792957 -1.29 0.197 -1.882461 .3883369

ALBULN | -.7032057 .4959234 -1.42 0.156 -1.675198 .2687863

LDHULN | -.0953833 .3169391 -0.30 0.763 -.7165726 .525806

eq5dbase | -.9751098 .4580464 -2.13 0.033 -1.872864 -.0773555

CEAULN | .5604943 .7095468 0.79 0.430 -.8301918 1.951181

eq5dmissb | -.2254449 .5722768 -0.39 0.694 -1.347087 .8961971

regionstrat | .0606994 .122675 0.49 0.621 -.1797392 .301138

PDDYLR | .0079124 .0042986 1.84 0.066 -.0005126 .0163375

eq5datprog | .5125582 .4178718 1.23 0.220 -.3064555 1.331572

eq5dind | -.1556607 .7364155 -0.21 0.833 -1.599008 1.287687

eq5dmissingatprog | -.0253933 .3522659 -0.07 0.943 -.7158218 .6650353

ecogatprog | -.4815685 .1499394 -3.21 0.001 -.7754443 -.1876927

bestrespatprog | .1726948 .1895416 0.91 0.362 -.1987999 .5441895

respmissingatprog | -.4427165 .2593852 -1.71 0.088 -.9511021 .0656691

LSSLDatprog | -.0015657 .0009261 -1.69 0.091 -.0033808 .0002495

LSSLDmissingatprog | -.3441713 .200155 -1.72 0.086 -.7364679 .0481253

AATULNatprog | .0018967 .2029796 0.01 0.993 -.3959361 .3997295

AATmissingatprog | .0381771 .2961605 0.13 0.897 -.5422869 .6186411

ALBULNatprog | .2559409 .4799405 0.53 0.594 -.6847253 1.196607

ALBmissingatprog | 0 (omitted)

ALKULNatprog | .0494686 .2278724 0.22 0.828 -.397153 .4960902

ALKmissingatprog | 0 (omitted)

ASTULNatprog | -.5193147 .3094655 -1.68 0.093 -1.125856 .0872264

ASTmissingatprog | 0 (omitted)

CEAULNatprog | -.7102453 .7224072 -0.98 0.326 -2.126137 .7056467

CEAmissingatprog | -.0588987 .2524328 -0.23 0.816 -.5536579 .4358605

CREATULNatprog | 0 (omitted)

CREATmissingatprog | 0 (omitted)

LDHULNatprog | .3201592 .3540543 0.90 0.366 -.3737745 1.014093

LDHmissingatprog | 0 (omitted)

BILIULNatprog | -.3495642 .2835107 -1.23 0.218 -.9052349 .2061065

BILImissingatprog | 0 (omitted)

saeatprog | .7110613 .8878692 0.80 0.423 -1.02913 2.451253

_cons | 5.375161 1.243476 4.32 0.000 2.937993 7.81233

-------------------+----------------------------------------------------------------

/lnsigma | -.3294761 .0712512 -4.62 0.000 -.4691259 -.1898264

-------------------+----------------------------------------------------------------

sigma | .7193005 .051251 .6255488 .8271027

------------------------------------------------------------------------------------

. * Same model as for IPCW denominator but without the TDC variables. Note, had to take ecog

> misslastvisit out as !=0 perfectly predicted failure.

. * And, ALBmisslastvisit, ALKmisslastvisit, ASTmisslastvisit, CREATmisslastvisit, LDHmissla

> stvisit, BILImisslastvisit all omitted because of collinearity

.

. scalar tsec_af = exp(_b[trtnew])

. di tsec_af

2.3254723

.

. restore

. sort SUBJID

. preserve

.

. ***Analysis on overall survival***

. collapse (max) trtgrp krasi xo regionstrat becogstrat dthdyxtdc xotime deathtdc admin, by(

> SUBJID)

. by SUBJID: replace xotime=0 if xotime==.

(259 real changes made)

. by SUBJID: replace xotime = 0 if krasi==1 & trtgrp==1 & xo==1

(77 real changes made)

. by SUBJID: replace xo = 0 if krasi==1 & trtgrp==1 & xo==1

(77 real changes made)

.

. ***below allows for recensoring***

. gen cfact = dthdyxtdc if trtgrp==2

(219 missing values generated)

. gen dcfact = deathtdc if trtgrp==2

(219 missing values generated)

.

. replace cfact = (xotime + ((dthdyxtdc-xotime)/(tsec_af))) if (trtgrp==1 & xotime>0)

(91 real changes made)

. replace cfact = dthdyxtdc if (trtgrp==1 & xotime==0)

(128 real changes made)

. gen OSadminc = admin/(tsec_af) if (trtgrp==1 & (tsec_af)>1.00)

(208 missing values generated)

. replace dcfact = deathtdc if trtgrp==1

(219 real changes made)

. replace dcfact=0 if (OSadminc<=cfact & trtgrp==1)

(12 real changes made)

. replace cfact = OSadminc if (OSadminc<=cfact & trtgrp==1)

(26 real changes made)

.

. ***do survival analysis on re-estimated survival times***

. stset cfact, failure(dcfact) id(SUBJID)

Survival-time data settings

ID variable: SUBJID

Failure event: dcfact!=0 & dcfact<.

Observed time interval: (cfact[_n-1], cfact]

Exit on or before: failure

--------------------------------------------------------------------------

427 total observations

0 exclusions

--------------------------------------------------------------------------

427 observations remaining, representing

427 subjects

379 failures in single-failure-per-subject data

85,361.994 total analysis time at risk and under observation

At risk from t = 0

Earliest observed entry t = 0

Last observed exit t = 1,024

. stcox trtgrp regionstrat becogstrat

Failure _d: dcfact

Analysis time _t: cfact

ID variable: SUBJID

Iteration 0: log likelihood = -1961.5382

Iteration 1: log likelihood = -1942.6387

Iteration 2: log likelihood = -1925.1644

Iteration 3: log likelihood = -1924.8236

Iteration 4: log likelihood = -1924.8233

Refining estimates:

Iteration 0: log likelihood = -1924.8233

Cox regression with Breslow method for ties

No. of subjects = 427 Number of obs = 427

No. of failures = 379

Time at risk = 85,361.9938

LR chi2(3) = 73.43

Log likelihood = -1924.8233 Prob > chi2 = 0.0000

------------------------------------------------------------------------------

_t | Haz. ratio Std. err. z P>|z| [95% conf. interval]

-------------+----------------------------------------------------------------

trtgrp | .6206577 .067226 -4.40 0.000 .5019437 .7674486

regionstrat | 1.017469 .071123 0.25 0.804 .8871982 1.166868

becogstrat | 3.486213 .5179837 8.40 0.000 2.605445 4.664724

------------------------------------------------------------------------------

.

. restore

.

. ******************************************************************************************

> ***************

. *** TSEsimp 3 (primary analysis, full model, no recens, Weibull) Estimand 1 [219 from TSEs

> imp file] ***

. ******************************************************************************************

> ***************

.

. preserve

.

. * note, 8 patients switched before investigator observed progression. 7 were kras MT, so p

> rimary analyses will not adjust for these anyway.

. * protocol suggested switching permitted only after progression. So assume these patients

> switched due to some signs of progression.

. * need this, because SNM models will fit much better if applied only to the time-periods w

> here switching was "permitted".

. * so, for these 8 patients, replace PDDYLR to equal xotime

. * and replace progtdc to = 1 after this point for these patients

. sort SUBJID dthdyxtdc

. by SUBJID: replace PDDYLR = xotime if (PDDYLR>xotime & xotime!=.)

(1,252 real changes made)

. by SUBJID: replace progtdc = 1 if (PDDYLR <= dthdyxtdc & PDLR==1)

(984 real changes made)

.

. replace xotdc = 0 if krasi==1 & trtgrp==1

(14,415 real changes made)

. replace xo = . if krasi==1 & trtgrp==1 & xo==1

(18,500 real changes made, 18,500 to missing)

.

. by SUBJID: replace lastobs = 0

(427 real changes made)

. by SUBJID: replace lastobs = 1 if _n==_N

(427 real changes made)

.

. *** streg

. sort SUBJID dthdyxtdc

. drop if trtgrp==2

(49,968 observations deleted)

. drop if progtdc==0

(10,866 observations deleted)

. * drop anyone who died on same day as progression

. drop if PDDYLR == deathtime

(16 observations deleted)

. * for primary analysis, get treatment effect comparing WT switchers to WT non-switchers, i

> .e. exclude MT

. drop if krasi==1

(16,044 observations deleted)

. by SUBJID: gen obsno = _n

. by SUBJID: gen trtnew = 0

. by SUBJID: replace trtnew = 1 if xo==1 & dthdyxtdc>=xotime

(22802 real changes made)

. by SUBJID: egen minrisk=min(time)

. by SUBJID: replace dthdyxtdc=dthdyxtdc-minrisk

(24923 real changes made)

. by SUBJID: replace xotime=xotime-minrisk

(24117 real changes made)

. by SUBJID: replace time=time-minrisk

(24923 real changes made)

. by SUBJID: replace admin=admin-minrisk

(24923 real changes made)

.

. stset dthdyxtdc, failure(deathtdc) id(SUBJID)

Survival-time data settings

ID variable: SUBJID

Failure event: deathtdc!=0 & deathtdc<.

Observed time interval: (dthdyxtdc[_n-1], dthdyxtdc]

Exit on or before: failure

--------------------------------------------------------------------------

24,923 total observations

0 exclusions

--------------------------------------------------------------------------

24,923 observations remaining, representing

106 subjects

100 failures in single-failure-per-subject data

24,923 total analysis time at risk and under observation

At risk from t = 0

Earliest observed entry t = 0

Last observed exit t = 784

. streg trtnew AGE becogstrat diagtype BILIULN ASTULN CREATULN ALBULN LDHULN eq5dbase CEAULN

> eq5dmissb regionstrat PDDYLR eq5datprog eq5dind eq5dmissingatprog ecogatprog bestrespatpr

> og respmissingatprog LSSLDatprog LSSLDmissingatprog AATULNatprog AATmissingatprog ALBULNat

> prog ALBmissingatprog ALKULNatprog ALKmissingatprog ASTULNatprog ASTmissingatprog CEAULNat

> prog CEAmissingatprog CREATULNatprog CREATmissingatprog LDHULNatprog LDHmissingatprog BILI

> ULNatprog BILImissingatprog saeatprog, dist(weibull) time iterate(200)

Failure _d: deathtdc

Analysis time _t: dthdyxtdc

ID variable: SUBJID

note: ALBmissingatprog omitted because of collinearity.

note: ALKmissingatprog omitted because of collinearity.

note: ASTmissingatprog omitted because of collinearity.

note: CREATULNatprog omitted because of collinearity.

note: CREATmissingatprog omitted because of collinearity.

note: LDHmissingatprog omitted because of collinearity.

note: BILImissingatprog omitted because of collinearity.

Fitting constant-only model:

Iteration 0: log likelihood = -155.00396

Iteration 1: log likelihood = -153.4072

Iteration 2: log likelihood = -153.40372

Iteration 3: log likelihood = -153.40372

Fitting full model:

Iteration 0: log likelihood = -153.40372

Iteration 1: log likelihood = -121.70482

Iteration 2: log likelihood = -109.88398

Iteration 3: log likelihood = -108.2494

Iteration 4: log likelihood = -108.22721

Iteration 5: log likelihood = -108.22719

Iteration 6: log likelihood = -108.22719

Weibull AFT regression

No. of subjects = 106 Number of obs = 24,923

No. of failures = 100

Time at risk = 24,923

LR chi2(32) = 90.35

Log likelihood = -108.22719 Prob > chi2 = 0.0000

------------------------------------------------------------------------------------

_t | Coefficient Std. err. z P>|z| [95% conf. interval]

-------------------+----------------------------------------------------------------

trtnew | .7925562 .216738 3.66 0.000 .3677574 1.217355

AGE | -.0048295 .0070864 -0.68 0.496 -.0187185 .0090596

becogstrat | .5230229 .2813373 1.86 0.063 -.028388 1.074434

diagtype | .2206367 .1607451 1.37 0.170 -.094418 .5356914

BILIULN | .0355378 .2173681 0.16 0.870 -.3904958 .4615715

ASTULN | .573513 .2583067 2.22 0.026 .0672413 1.079785

CREATULN | -1.204197 .4134357 -2.91 0.004 -2.014516 -.3938775

ALBULN | -.4468884 .3860208 -1.16 0.247 -1.203475 .3096984

LDHULN | .3112406 .2954532 1.05 0.292 -.267837 .8903183

eq5dbase | -.8261484 .3899927 -2.12 0.034 -1.59052 -.0617767

CEAULN | -.2930274 .5818143 -0.50 0.615 -1.433363 .8473077

eq5dmissb | -.2718747 .4762672 -0.57 0.568 -1.205341 .6615919

regionstrat | .0517618 .1067807 0.48 0.628 -.1575245 .2610481

PDDYLR | .0138439 .0040986 3.38 0.001 .0058107 .0218771

eq5datprog | .0695035 .3785514 0.18 0.854 -.6724436 .8114505

eq5dind | .1851247 .6082364 0.30 0.761 -1.006997 1.377246

eq5dmissingatprog | -.0166687 .3018315 -0.06 0.956 -.6082476 .5749102

ecogatprog | -.4862551 .1328997 -3.66 0.000 -.7467337 -.2257765

bestrespatprog | .2345417 .1538745 1.52 0.127 -.0670468 .5361302

respmissingatprog | -.5821665 .2255813 -2.58 0.010 -1.024298 -.1400353

LSSLDatprog | -.0019712 .0007874 -2.50 0.012 -.0035146 -.0004278

LSSLDmissingatprog | -.1649233 .1740925 -0.95 0.343 -.5061384 .1762917

AATULNatprog | -.0258887 .1727636 -0.15 0.881 -.3644992 .3127218

AATmissingatprog | -.4600322 .2435557 -1.89 0.059 -.9373926 .0173282

ALBULNatprog | -.1491619 .3829531 -0.39 0.697 -.8997361 .6014123

ALBmissingatprog | 0 (omitted)

ALKULNatprog | -.1218627 .2242434 -0.54 0.587 -.5613717 .3176463

ALKmissingatprog | 0 (omitted)

ASTULNatprog | -.7328966 .258833 -2.83 0.005 -1.2402 -.2255933

ASTmissingatprog | 0 (omitted)

CEAULNatprog | .3808302 .5993283 0.64 0.525 -.7938317 1.555492

CEAmissingatprog | -.3042879 .2144844 -1.42 0.156 -.7246697 .1160938

CREATULNatprog | 0 (omitted)

CREATmissingatprog | 0 (omitted)

LDHULNatprog | .238702 .3197934 0.75 0.455 -.3880815 .8654855

LDHmissingatprog | 0 (omitted)

BILIULNatprog | -.595671 .2202507 -2.70 0.007 -1.027354 -.1639875

BILImissingatprog | 0 (omitted)

saeatprog | 1.303075 .6652494 1.96 0.050 -.0007896 2.60694

_cons | 5.040682 1.000408 5.04 0.000 3.079918 7.001445

-------------------+----------------------------------------------------------------

/ln_p | .6606433 .0842989 7.84 0.000 .4954204 .8258662

-------------------+----------------------------------------------------------------

p | 1.936037 .1632059 1.641188 2.283858

1/p | .5165189 .043542 .4378556 .6093147

------------------------------------------------------------------------------------

.

. * Same model as for IPCW denominator. Note, had to take ecogmisslastvisit out as !=0 perfe

> ctly predicted failure.

. * And, ALBmisslastvisit, ALKmisslastvisit, ASTmisslastvisit, CREATmisslastvisit, LDHmissla

> stvisit, BILImisslastvisit all omitted because of collinearity

.

. scalar tsec_af = exp(_b[trtnew])

. di tsec_af

2.2090359

.

. restore

. sort SUBJID

. preserve

.

. ***Analysis on overall survival***

. collapse (max) trtgrp krasi xo regionstrat becogstrat dthdyxtdc xotime deathtdc admin, by(

> SUBJID)

. by SUBJID: replace xotime=0 if xotime==.

(259 real changes made)

. by SUBJID: replace xotime = 0 if krasi==1 & trtgrp==1 & xo==1

(77 real changes made)

. by SUBJID: replace xo = 0 if krasi==1 & trtgrp==1 & xo==1

(77 real changes made)

.

. ***below no recensoring***

. gen cfact = dthdyxtdc if trtgrp==2

(219 missing values generated)

. gen dcfact = deathtdc if trtgrp==2

(219 missing values generated)

.

. replace cfact = (xotime + ((dthdyxtdc-xotime)/(tsec_af))) if (trtgrp==1 & xotime>0)

(91 real changes made)

. replace cfact = dthdyxtdc if (trtgrp==1 & xotime==0)

(128 real changes made)

. replace dcfact = deathtdc if trtgrp==1

(219 real changes made)

.

. ***do survival analysis on re-estimated survival times***

. stset cfact, failure(dcfact) id(SUBJID)

Survival-time data settings

ID variable: SUBJID

Failure event: dcfact!=0 & dcfact<.

Observed time interval: (cfact[_n-1], cfact]

Exit on or before: failure

--------------------------------------------------------------------------

427 total observations

0 exclusions

--------------------------------------------------------------------------

427 observations remaining, representing

427 subjects

391 failures in single-failure-per-subject data

89,386.955 total analysis time at risk and under observation

At risk from t = 0

Earliest observed entry t = 0

Last observed exit t = 1,024

.

. stcox trtgrp regionstrat becogstrat

Failure _d: dcfact

Analysis time _t: cfact

ID variable: SUBJID

Iteration 0: log likelihood = -2018.656

Iteration 1: log likelihood = -2002.7485

Iteration 2: log likelihood = -1985.0645

Iteration 3: log likelihood = -1984.7174

Iteration 4: log likelihood = -1984.717

Refining estimates:

Iteration 0: log likelihood = -1984.717

Cox regression with Breslow method for ties

No. of subjects = 427 Number of obs = 427

No. of failures = 391

Time at risk = 89,386.9554

LR chi2(3) = 67.88

Log likelihood = -1984.717 Prob > chi2 = 0.0000

------------------------------------------------------------------------------

_t | Haz. ratio Std. err. z P>|z| [95% conf. interval]

-------------+----------------------------------------------------------------

trtgrp | .6836045 .0700221 -3.71 0.000 .5592625 .8355916

regionstrat | .9980847 .0690585 -0.03 0.978 .871509 1.143044

becogstrat | 3.482154 .5172456 8.40 0.000 2.602609 4.658939

------------------------------------------------------------------------------

.

. restore

.

. ******************************************************************************************

> ***************

. *** TSEsimp 4 (primary analysis, full model, no recens, lognormal) Estimand 1 ***

. ******************************************************************************************

> ***************

.

. preserve

.

. * note, 8 patients switched before investigator observed progression. 7 were kras MT, so p

> rimary analyses will not adjust for these anyway.

. * protocol suggested switching permitted only after progression. So assume these patients

> switched due to some signs of progression.

. * need this, because SNM models will fit much better if applied only to the time-periods w

> here switching was "permitted".

. * so, for these 8 patients, replace PDDYLR to equal xotime

. * and replace progtdc to = 1 after this point for these patients

. sort SUBJID dthdyxtdc

. by SUBJID: replace PDDYLR = xotime if (PDDYLR>xotime & xotime!=.)

(1,252 real changes made)

. by SUBJID: replace progtdc = 1 if (PDDYLR <= dthdyxtdc & PDLR==1)

(984 real changes made)

.

. replace xotdc = 0 if krasi==1 & trtgrp==1

(14,415 real changes made)

. replace xo = . if krasi==1 & trtgrp==1 & xo==1

(18,500 real changes made, 18,500 to missing)

.

. by SUBJID: replace lastobs = 0

(427 real changes made)

. by SUBJID: replace lastobs = 1 if _n==_N

(427 real changes made)

.

. *** streg

. sort SUBJID dthdyxtdc

. drop if trtgrp==2

(49,968 observations deleted)

. drop if progtdc==0

(10,866 observations deleted)

. * drop anyone who died on same day as progression

. drop if PDDYLR == deathtime

(16 observations deleted)

. * for primary analysis, get treatment effect comparing WT switchers to WT non-switchers, i

> .e. exclude MT

. drop if krasi==1

(16,044 observations deleted)

. by SUBJID: gen obsno = _n

. by SUBJID: gen trtnew = 0

. by SUBJID: replace trtnew = 1 if xo==1 & dthdyxtdc>=xotime

(22802 real changes made)

. by SUBJID: egen minrisk=min(time)

. by SUBJID: replace dthdyxtdc=dthdyxtdc-minrisk

(24923 real changes made)

. by SUBJID: replace xotime=xotime-minrisk

(24117 real changes made)

. by SUBJID: replace time=time-minrisk

(24923 real changes made)

. by SUBJID: replace admin=admin-minrisk

(24923 real changes made)

.

. stset dthdyxtdc, failure(deathtdc) id(SUBJID)

Survival-time data settings

ID variable: SUBJID

Failure event: deathtdc!=0 & deathtdc<.

Observed time interval: (dthdyxtdc[_n-1], dthdyxtdc]

Exit on or before: failure

--------------------------------------------------------------------------

24,923 total observations

0 exclusions

--------------------------------------------------------------------------

24,923 observations remaining, representing

106 subjects

100 failures in single-failure-per-subject data

24,923 total analysis time at risk and under observation

At risk from t = 0

Earliest observed entry t = 0

Last observed exit t = 784

. streg trtnew AGE becogstrat diagtype BILIULN ASTULN CREATULN ALBULN LDHULN eq5dbase CEAULN

> eq5dmissb regionstrat PDDYLR eq5datprog eq5dind eq5dmissingatprog ecogatprog bestrespatpr

> og respmissingatprog LSSLDatprog LSSLDmissingatprog AATULNatprog AATmissingatprog ALBULNat

> prog ALBmissingatprog ALKULNatprog ALKmissingatprog ASTULNatprog ASTmissingatprog CEAULNat

> prog CEAmissingatprog CREATULNatprog CREATmissingatprog LDHULNatprog LDHmissingatprog BILI

> ULNatprog BILImissingatprog saeatprog, dist(lognormal) time iterate(200)

Failure _d: deathtdc

Analysis time _t: dthdyxtdc

ID variable: SUBJID

note: ALBmissingatprog omitted because of collinearity.

note: ALKmissingatprog omitted because of collinearity.

note: ASTmissingatprog omitted because of collinearity.

note: CREATULNatprog omitted because of collinearity.

note: CREATmissingatprog omitted because of collinearity.

note: LDHmissingatprog omitted because of collinearity.

note: BILImissingatprog omitted because of collinearity.

Fitting constant-only model:

Iteration 0: log likelihood = -222.34803

Iteration 1: log likelihood = -198.01902

Iteration 2: log likelihood = -161.56774

Iteration 3: log likelihood = -160.997

Iteration 4: log likelihood = -160.98957

Iteration 5: log likelihood = -160.98957

Fitting full model:

Iteration 0: log likelihood = -160.98957 (not concave)

Iteration 1: log likelihood = -137.01662

Iteration 2: log likelihood = -121.81787

Iteration 3: log likelihood = -118.32556

Iteration 4: log likelihood = -118.29361

Iteration 5: log likelihood = -118.2936

Lognormal AFT regression

No. of subjects = 106 Number of obs = 24,923

No. of failures = 100

Time at risk = 24,923

LR chi2(32) = 85.39

Log likelihood = -118.2936 Prob > chi2 = 0.0000

------------------------------------------------------------------------------------

_t | Coefficient Std. err. z P>|z| [95% conf. interval]

-------------------+----------------------------------------------------------------

trtnew | .8439232 .224195 3.76 0.000 .404509 1.283337

AGE | -.0028388 .0081509 -0.35 0.728 -.0188142 .0131367

becogstrat | .2509313 .3262883 0.77 0.442 -.388582 .8904446

diagtype | .0355247 .1880266 0.19 0.850 -.3330007 .4040502

BILIULN | .3498442 .273748 1.28 0.201 -.1866921 .8863805

ASTULN | .2321577 .2883656 0.81 0.421 -.3330284 .7973438

CREATULN | -.7470618 .5792957 -1.29 0.197 -1.882461 .3883369

ALBULN | -.7032057 .4959234 -1.42 0.156 -1.675198 .2687863

LDHULN | -.0953833 .3169391 -0.30 0.763 -.7165726 .525806

eq5dbase | -.9751098 .4580464 -2.13 0.033 -1.872864 -.0773555

CEAULN | .5604943 .7095468 0.79 0.430 -.8301918 1.951181

eq5dmissb | -.2254449 .5722768 -0.39 0.694 -1.347087 .8961971

regionstrat | .0606994 .122675 0.49 0.621 -.1797392 .301138

PDDYLR | .0079124 .0042986 1.84 0.066 -.0005126 .0163375

eq5datprog | .5125582 .4178718 1.23 0.220 -.3064555 1.331572

eq5dind | -.1556607 .7364155 -0.21 0.833 -1.599008 1.287687

eq5dmissingatprog | -.0253933 .3522659 -0.07 0.943 -.7158218 .6650353

ecogatprog | -.4815685 .1499394 -3.21 0.001 -.7754443 -.1876927

bestrespatprog | .1726948 .1895416 0.91 0.362 -.1987999 .5441895

respmissingatprog | -.4427165 .2593852 -1.71 0.088 -.9511021 .0656691

LSSLDatprog | -.0015657 .0009261 -1.69 0.091 -.0033808 .0002495

LSSLDmissingatprog | -.3441713 .200155 -1.72 0.086 -.7364679 .0481253

AATULNatprog | .0018967 .2029796 0.01 0.993 -.3959361 .3997295

AATmissingatprog | .0381771 .2961605 0.13 0.897 -.5422869 .6186411

ALBULNatprog | .2559409 .4799405 0.53 0.594 -.6847253 1.196607

ALBmissingatprog | 0 (omitted)

ALKULNatprog | .0494686 .2278724 0.22 0.828 -.397153 .4960902

ALKmissingatprog | 0 (omitted)

ASTULNatprog | -.5193147 .3094655 -1.68 0.093 -1.125856 .0872264

ASTmissingatprog | 0 (omitted)

CEAULNatprog | -.7102453 .7224072 -0.98 0.326 -2.126137 .7056467

CEAmissingatprog | -.0588987 .2524328 -0.23 0.816 -.5536579 .4358605

CREATULNatprog | 0 (omitted)

CREATmissingatprog | 0 (omitted)

LDHULNatprog | .3201592 .3540543 0.90 0.366 -.3737745 1.014093

LDHmissingatprog | 0 (omitted)

BILIULNatprog | -.3495642 .2835107 -1.23 0.218 -.9052349 .2061065

BILImissingatprog | 0 (omitted)

saeatprog | .7110613 .8878692 0.80 0.423 -1.02913 2.451253

_cons | 5.375161 1.243476 4.32 0.000 2.937993 7.81233

-------------------+----------------------------------------------------------------

/lnsigma | -.3294761 .0712512 -4.62 0.000 -.4691259 -.1898264

-------------------+----------------------------------------------------------------

sigma | .7193005 .051251 .6255488 .8271027

------------------------------------------------------------------------------------

.

. * Same model as for IPCW denominator. Note, had to take ecogmisslastvisit out as !=0 perfe

> ctly predicted failure.

. * And, ALBmisslastvisit, ALKmisslastvisit, ASTmisslastvisit, CREATmisslastvisit, LDHmissla

> stvisit, BILImisslastvisit all omitted because of collinearity

.

. scalar tsec_af = exp(_b[trtnew])

. di tsec_af

2.3254723

.

. restore

. sort SUBJID

. preserve

.

. ***Analysis on overall survival***

. collapse (max) trtgrp krasi xo regionstrat becogstrat dthdyxtdc xotime deathtdc admin, by(

> SUBJID)

. by SUBJID: replace xotime=0 if xotime==.

(259 real changes made)

. by SUBJID: replace xotime = 0 if krasi==1 & trtgrp==1 & xo==1

(77 real changes made)

. by SUBJID: replace xo = 0 if krasi==1 & trtgrp==1 & xo==1

(77 real changes made)

.

. ***below no recensoring***

. gen cfact = dthdyxtdc if trtgrp==2

(219 missing values generated)

. gen dcfact = deathtdc if trtgrp==2

(219 missing values generated)

.

. replace cfact = (xotime + ((dthdyxtdc-xotime)/(tsec_af))) if (trtgrp==1 & xotime>0)

(91 real changes made)

. replace cfact = dthdyxtdc if (trtgrp==1 & xotime==0)

(128 real changes made)

. replace dcfact = deathtdc if trtgrp==1

(219 real changes made)

.

. ***do survival analysis on re-estimated survival times***

. stset cfact, failure(dcfact) id(SUBJID)

Survival-time data settings

ID variable: SUBJID

Failure event: dcfact!=0 & dcfact<.

Observed time interval: (cfact[_n-1], cfact]

Exit on or before: failure

--------------------------------------------------------------------------

427 total observations

0 exclusions

--------------------------------------------------------------------------

427 observations remaining, representing

427 subjects

391 failures in single-failure-per-subject data

88,872.188 total analysis time at risk and under observation

At risk from t = 0

Earliest observed entry t = 0

Last observed exit t = 1,024

.

. stcox trtgrp regionstrat becogstrat

Failure _d: dcfact

Analysis time _t: cfact

ID variable: SUBJID

Iteration 0: log likelihood = -2018.3428

Iteration 1: log likelihood = -2001.7599

Iteration 2: log likelihood = -1984.1317

Iteration 3: log likelihood = -1983.7886

Iteration 4: log likelihood = -1983.7883

Refining estimates:

Iteration 0: log likelihood = -1983.7883

Cox regression with Breslow method for ties

No. of subjects = 427 Number of obs = 427

No. of failures = 391

Time at risk = 88,872.1881

LR chi2(3) = 69.11

Log likelihood = -1983.7883 Prob > chi2 = 0.0000

------------------------------------------------------------------------------

_t | Haz. ratio Std. err. z P>|z| [95% conf. interval]

-------------+----------------------------------------------------------------

trtgrp | .6720614 .0688943 -3.88 0.000 .5497315 .8216129

regionstrat | .9984275 .0691096 -0.02 0.982 .8717615 1.143498

becogstrat | 3.489415 .5185793 8.41 0.000 2.607662 4.669323

------------------------------------------------------------------------------

.

. restore

.

. ******************************************************************************************

> ***************

. *** TSEsimp 5 (primary analysis, reduced model, with recens, Weibull) Estimand 1 [221 from

> TSEsimp file] ***

. ******************************************************************************************

> ***************

. preserve

.

. * note, 8 patients switched before investigator observed progression. 7 were kras MT, so p

> rimary analyses will not adjust for these anyway.

. * protocol suggested switching permitted only after progression. So assume these patients

> switched due to some signs of progression.

. * need this, because SNM models will fit much better if applied only to the time-periods w

> here switching was "permitted".

. * so, for these 8 patients, replace PDDYLR to equal xotime

. * and replace progtdc to = 1 after this point for these patients

. sort SUBJID dthdyxtdc

. by SUBJID: replace PDDYLR = xotime if (PDDYLR>xotime & xotime!=.)

(1,252 real changes made)

. by SUBJID: replace progtdc = 1 if (PDDYLR <= dthdyxtdc & PDLR==1)

(984 real changes made)

.

. replace xotdc = 0 if krasi==1 & trtgrp==1

(14,415 real changes made)

. replace xo = . if krasi==1 & trtgrp==1 & xo==1

(18,500 real changes made, 18,500 to missing)

.

. by SUBJID: replace lastobs = 0

(427 real changes made)

. by SUBJID: replace lastobs = 1 if _n==_N

(427 real changes made)

.

. *** streg

. sort SUBJID dthdyxtdc

. drop if trtgrp==2

(49,968 observations deleted)

. drop if progtdc==0

(10,866 observations deleted)

. * drop anyone who died on same day as progression

. drop if PDDYLR == deathtime

(16 observations deleted)

. * for primary analysis, get treatment effect comparing WT switchers to WT non-switchers, i

> .e. exclude MT

. drop if krasi==1

(16,044 observations deleted)

. by SUBJID: gen obsno = _n

. by SUBJID: gen trtnew = 0

. by SUBJID: replace trtnew = 1 if xo==1 & dthdyxtdc>=xotime

(22802 real changes made)

. by SUBJID: egen minrisk=min(time)

. by SUBJID: replace dthdyxtdc=dthdyxtdc-minrisk

(24923 real changes made)

. by SUBJID: replace xotime=xotime-minrisk

(24117 real changes made)

. by SUBJID: replace time=time-minrisk

(24923 real changes made)

. by SUBJID: replace admin=admin-minrisk

(24923 real changes made)

.

. stset dthdyxtdc, failure(deathtdc) id(SUBJID)

Survival-time data settings

ID variable: SUBJID

Failure event: deathtdc!=0 & deathtdc<.

Observed time interval: (dthdyxtdc[_n-1], dthdyxtdc]

Exit on or before: failure

--------------------------------------------------------------------------

24,923 total observations

0 exclusions

--------------------------------------------------------------------------

24,923 observations remaining, representing

106 subjects

100 failures in single-failure-per-subject data

24,923 total analysis time at risk and under observation

At risk from t = 0

Earliest observed entry t = 0

Last observed exit t = 784

. streg trtnew becogstrat diagtype eq5dbase eq5dmissb regionstrat PDDYLR eq5datprog eq5dind

> eq5dmissingatprog ecogatprog bestrespatprog respmissingatprog LSSLDatprog LSSLDmissingatpr

> og, dist(weibull) time iterate(200)

Failure _d: deathtdc

Analysis time _t: dthdyxtdc

ID variable: SUBJID

Fitting constant-only model:

Iteration 0: log likelihood = -155.00396

Iteration 1: log likelihood = -153.4072

Iteration 2: log likelihood = -153.40372

Iteration 3: log likelihood = -153.40372

Fitting full model:

Iteration 0: log likelihood = -153.40372

Iteration 1: log likelihood = -136.61921

Iteration 2: log likelihood = -124.21975

Iteration 3: log likelihood = -123.98834

Iteration 4: log likelihood = -123.98772

Iteration 5: log likelihood = -123.98772

Weibull AFT regression

No. of subjects = 106 Number of obs = 24,923

No. of failures = 100

Time at risk = 24,923

LR chi2(15) = 58.83

Log likelihood = -123.98772 Prob > chi2 = 0.0000

------------------------------------------------------------------------------------

_t | Coefficient Std. err. z P>|z| [95% conf. interval]

-------------------+----------------------------------------------------------------

trtnew | .9398798 .2216702 4.24 0.000 .5054141 1.374346

becogstrat | .2740033 .2863727 0.96 0.339 -.2872768 .8352835

diagtype | .0582467 .1484566 0.39 0.695 -.2327229 .3492162

eq5dbase | -.8388337 .3828969 -2.19 0.028 -1.589298 -.0883696

eq5dmissb | -.908241 .4555363 -1.99 0.046 -1.801076 -.0154063

regionstrat | -.0122116 .1021417 -0.12 0.905 -.2124056 .1879825

PDDYLR | .0104706 .003504 2.99 0.003 .003603 .0173383

eq5datprog | .1290311 .3465396 0.37 0.710 -.5501741 .8082363

eq5dind | -.3294473 .6215387 -0.53 0.596 -1.547641 .8887461

eq5dmissingatprog | .1331492 .2937642 0.45 0.650 -.4426181 .7089165

ecogatprog | -.4896557 .1358889 -3.60 0.000 -.7559931 -.2233184

bestrespatprog | .2451107 .1612552 1.52 0.129 -.0709438 .5611651

respmissingatprog | -.3421478 .2340022 -1.46 0.144 -.8007837 .1164881

LSSLDatprog | -.0013835 .0006297 -2.20 0.028 -.0026177 -.0001494

LSSLDmissingatprog | -.2766897 .1614798 -1.71 0.087 -.5931843 .0398049

_cons | 5.10881 .8325282 6.14 0.000 3.477085 6.740535

-------------------+----------------------------------------------------------------

/ln_p | .4852643 .0799426 6.07 0.000 .3285797 .6419489

-------------------+----------------------------------------------------------------

p | 1.624604 .1298751 1.388994 1.90018

1/p | .6155345 .0492074 .5262658 .7199456

------------------------------------------------------------------------------------

. * Same model as for IPCW denominator except without tdc terms. Note, had to take ecogmissl

> astvisit out as !=0 perfectly predicted failure.

.

. scalar tsec_af = exp(_b[trtnew])

. di tsec_af

2.5596738

.

. restore

. sort SUBJID

. preserve

.

. ***Analysis on overall survival***

. collapse (max) trtgrp krasi xo regionstrat becogstrat dthdyxtdc xotime deathtdc admin, by(

> SUBJID)

. by SUBJID: replace xotime=0 if xotime==.

(259 real changes made)

. by SUBJID: replace xotime = 0 if krasi==1 & trtgrp==1 & xo==1

(77 real changes made)

. by SUBJID: replace xo = 0 if krasi==1 & trtgrp==1 & xo==1

(77 real changes made)

.

. ***below allows for recensoring***

. gen cfact = dthdyxtdc if trtgrp==2

(219 missing values generated)

. gen dcfact = deathtdc if trtgrp==2

(219 missing values generated)

.

. replace cfact = (xotime + ((dthdyxtdc-xotime)/(tsec_af))) if (trtgrp==1 & xotime>0)

(91 real changes made)

. replace cfact = dthdyxtdc if (trtgrp==1 & xotime==0)

(128 real changes made)

. gen OSadminc = admin/(tsec_af) if (trtgrp==1 & (tsec_af)>1.00)

(208 missing values generated)

. replace dcfact = deathtdc if trtgrp==1

(219 real changes made)

. replace dcfact=0 if (OSadminc<=cfact & trtgrp==1)

(15 real changes made)

. replace cfact = OSadminc if (OSadminc<=cfact & trtgrp==1)

(29 real changes made)

.

. ***do survival analysis on re-estimated survival times***

. stset cfact, failure(dcfact) id(SUBJID)

Survival-time data settings

ID variable: SUBJID

Failure event: dcfact!=0 & dcfact<.

Observed time interval: (cfact[_n-1], cfact]

Exit on or before: failure

--------------------------------------------------------------------------

427 total observations

0 exclusions

--------------------------------------------------------------------------

427 observations remaining, representing

427 subjects

376 failures in single-failure-per-subject data

83,867.572 total analysis time at risk and under observation

At risk from t = 0

Earliest observed entry t = 0

Last observed exit t = 1,024

.

. stcox trtgrp regionstrat becogstrat

Failure _d: dcfact

Analysis time _t: cfact

ID variable: SUBJID

Iteration 0: log likelihood = -1946.0363

Iteration 1: log likelihood = -1925.1633

Iteration 2: log likelihood = -1907.9243

Iteration 3: log likelihood = -1907.5981

Iteration 4: log likelihood = -1907.5978

Refining estimates:

Iteration 0: log likelihood = -1907.5978

Cox regression with Breslow method for ties

No. of subjects = 427 Number of obs = 427

No. of failures = 376

Time at risk = 83,867.5721

LR chi2(3) = 76.88

Log likelihood = -1907.5978 Prob > chi2 = 0.0000

------------------------------------------------------------------------------

_t | Haz. ratio Std. err. z P>|z| [95% conf. interval]

-------------+----------------------------------------------------------------

trtgrp | .5914861 .0650629 -4.77 0.000 .4767747 .7337969

regionstrat | 1.027336 .0719083 0.39 0.700 .8956386 1.178399

becogstrat | 3.513558 .5229042 8.44 0.000 2.624625 4.703562

------------------------------------------------------------------------------

.

. restore

.

. ******************************************************************************************

> ***************

. *** TSEsimp 6 (primary analysis, reduced model, with recens, Gen Gamma) Estimand 1 ***

. ******************************************************************************************

> ***************

. preserve

.

. * note, 8 patients switched before investigator observed progression. 7 were kras MT, so p

> rimary analyses will not adjust for these anyway.

. * protocol suggested switching permitted only after progression. So assume these patients

> switched due to some signs of progression.

. * need this, because SNM models will fit much better if applied only to the time-periods w

> here switching was "permitted".

. * so, for these 8 patients, replace PDDYLR to equal xotime

. * and replace progtdc to = 1 after this point for these patients

. sort SUBJID dthdyxtdc

. by SUBJID: replace PDDYLR = xotime if (PDDYLR>xotime & xotime!=.)

(1,252 real changes made)

. by SUBJID: replace progtdc = 1 if (PDDYLR <= dthdyxtdc & PDLR==1)

(984 real changes made)

.

. replace xotdc = 0 if krasi==1 & trtgrp==1

(14,415 real changes made)

. replace xo = . if krasi==1 & trtgrp==1 & xo==1

(18,500 real changes made, 18,500 to missing)

.

. by SUBJID: replace lastobs = 0

(427 real changes made)

. by SUBJID: replace lastobs = 1 if _n==_N

(427 real changes made)

.

. *** streg

. sort SUBJID dthdyxtdc

. drop if trtgrp==2

(49,968 observations deleted)

. drop if progtdc==0

(10,866 observations deleted)

. * drop anyone who died on same day as progression

. drop if PDDYLR == deathtime

(16 observations deleted)

. * for primary analysis, get treatment effect comparing WT switchers to WT non-switchers, i

> .e. exclude MT

. drop if krasi==1

(16,044 observations deleted)

. by SUBJID: gen obsno = _n

. by SUBJID: gen trtnew = 0

. by SUBJID: replace trtnew = 1 if xo==1 & dthdyxtdc>=xotime

(22802 real changes made)

. by SUBJID: egen minrisk=min(time)

. by SUBJID: replace dthdyxtdc=dthdyxtdc-minrisk

(24923 real changes made)

. by SUBJID: replace xotime=xotime-minrisk

(24117 real changes made)

. by SUBJID: replace time=time-minrisk

(24923 real changes made)

. by SUBJID: replace admin=admin-minrisk

(24923 real changes made)

.

. stset dthdyxtdc, failure(deathtdc) id(SUBJID)

Survival-time data settings

ID variable: SUBJID

Failure event: deathtdc!=0 & deathtdc<.

Observed time interval: (dthdyxtdc[_n-1], dthdyxtdc]

Exit on or before: failure

--------------------------------------------------------------------------

24,923 total observations

0 exclusions

--------------------------------------------------------------------------

24,923 observations remaining, representing

106 subjects

100 failures in single-failure-per-subject data

24,923 total analysis time at risk and under observation

At risk from t = 0

Earliest observed entry t = 0

Last observed exit t = 784

. streg trtnew becogstrat diagtype eq5dbase eq5dmissb regionstrat PDDYLR eq5datprog eq5dind

> eq5dmissingatprog ecogatprog bestrespatprog respmissingatprog LSSLDatprog LSSLDmissingatpr

> og, dist(ggamma) time iterate(200)

Failure _d: deathtdc

Analysis time _t: dthdyxtdc

ID variable: SUBJID

Fitting constant-only model:

Iteration 0: log likelihood = -580.23762 (not concave)

Iteration 1: log likelihood = -226.73916 (not concave)

Iteration 2: log likelihood = -160.10683

Iteration 3: log likelihood = -156.87958

Iteration 4: log likelihood = -153.47437

Iteration 5: log likelihood = -153.32015

Iteration 6: log likelihood = -153.31691

Iteration 7: log likelihood = -153.31691

Fitting full model:

Iteration 0: log likelihood = -153.31691 (not concave)

Iteration 1: log likelihood = -139.92273 (not concave)

Iteration 2: log likelihood = -134.69516 (not concave)

Iteration 3: log likelihood = -132.09858 (not concave)

Iteration 4: log likelihood = -129.45604

Iteration 5: log likelihood = -126.09495

Iteration 6: log likelihood = -124.22244

Iteration 7: log likelihood = -123.97936

Iteration 8: log likelihood = -123.96834

Iteration 9: log likelihood = -123.96833

Generalized gamma AFT regression

No. of subjects = 106 Number of obs = 24,923

No. of failures = 100

Time at risk = 24,923

LR chi2(15) = 58.70

Log likelihood = -123.96833 Prob > chi2 = 0.0000

------------------------------------------------------------------------------------

_t | Coefficient Std. err. z P>|z| [95% conf. interval]

-------------------+----------------------------------------------------------------

trtnew | .9202868 .2501288 3.68 0.000 .4300433 1.41053

becogstrat | .3145031 .3656033 0.86 0.390 -.4020662 1.031073

diagtype | .0811653 .1935202 0.42 0.675 -.2981274 .460458

eq5dbase | -.8475395 .3802808 -2.23 0.026 -1.592876 -.1022028

eq5dmissb | -.9329829 .4640518 -2.01 0.044 -1.842508 -.0234582

regionstrat | -.0296866 .1402058 -0.21 0.832 -.3044849 .2451116

PDDYLR | .0108213 .0039915 2.71 0.007 .0029982 .0186445

eq5datprog | .1081325 .3683005 0.29 0.769 -.6137232 .8299882

eq5dind | -.2872059 .6715222 -0.43 0.669 -1.603365 1.028953

eq5dmissingatprog | .154406 .3281451 0.47 0.638 -.4887465 .7975585

ecogatprog | -.498531 .1446274 -3.45 0.001 -.7819954 -.2150666

bestrespatprog | .2550848 .1673917 1.52 0.128 -.072997 .5831665

respmissingatprog | -.3347191 .2352875 -1.42 0.155 -.7958742 .126436

LSSLDatprog | -.0012841 .0008177 -1.57 0.116 -.0028867 .0003185

LSSLDmissingatprog | -.2729895 .1612406 -1.69 0.090 -.5890153 .0430364

_cons | 5.050585 .8878638 5.69 0.000 3.310404 6.790766

-------------------+----------------------------------------------------------------

/lnsigma | -.5277874 .2617886 -2.02 0.044 -1.040884 -.0146911

/kappa | 1.138928 .7880484 1.45 0.148 -.405618 2.683475

-------------------+----------------------------------------------------------------

sigma | .5899088 .1544314 .3531425 .9854163

------------------------------------------------------------------------------------

. * Same model as for IPCW denominator except without tdc terms. Note, had to take ecogmissl

> astvisit out as !=0 perfectly predicted failure.

.

. scalar tsec_af = exp(_b[trtnew])

. di tsec_af

2.5100102

.

. restore

. sort SUBJID

. preserve

.

. ***Analysis on overall survival***

. collapse (max) trtgrp krasi xo regionstrat becogstrat dthdyxtdc xotime deathtdc admin, by(

> SUBJID)

. by SUBJID: replace xotime=0 if xotime==.

(259 real changes made)

. by SUBJID: replace xotime = 0 if krasi==1 & trtgrp==1 & xo==1

(77 real changes made)

. by SUBJID: replace xo = 0 if krasi==1 & trtgrp==1 & xo==1

(77 real changes made)

.

. ***below allows for recensoring***

. gen cfact = dthdyxtdc if trtgrp==2

(219 missing values generated)

. gen dcfact = deathtdc if trtgrp==2

(219 missing values generated)

.

. replace cfact = (xotime + ((dthdyxtdc-xotime)/(tsec_af))) if (trtgrp==1 & xotime>0)

(91 real changes made)

. replace cfact = dthdyxtdc if (trtgrp==1 & xotime==0)

(128 real changes made)

. gen OSadminc = admin/(tsec_af) if (trtgrp==1 & (tsec_af)>1.00)

(208 missing values generated)

. replace dcfact = deathtdc if trtgrp==1

(219 real changes made)

. replace dcfact=0 if (OSadminc<=cfact & trtgrp==1)

(15 real changes made)

. replace cfact = OSadminc if (OSadminc<=cfact & trtgrp==1)

(29 real changes made)

.

. ***do survival analysis on re-estimated survival times***

. stset cfact, failure(dcfact) id(SUBJID)

Survival-time data settings

ID variable: SUBJID

Failure event: dcfact!=0 & dcfact<.

Observed time interval: (cfact[_n-1], cfact]

Exit on or before: failure

--------------------------------------------------------------------------

427 total observations

0 exclusions

--------------------------------------------------------------------------

427 observations remaining, representing

427 subjects

376 failures in single-failure-per-subject data

84,167.503 total analysis time at risk and under observation

At risk from t = 0

Earliest observed entry t = 0

Last observed exit t = 1,024

.

. stcox trtgrp regionstrat becogstrat

Failure _d: dcfact

Analysis time _t: cfact

ID variable: SUBJID

Iteration 0: log likelihood = -1946.9122

Iteration 1: log likelihood = -1926.7812

Iteration 2: log likelihood = -1909.5071

Iteration 3: log likelihood = -1909.1787

Iteration 4: log likelihood = -1909.1783

Refining estimates:

Iteration 0: log likelihood = -1909.1783

Cox regression with Breslow method for ties

No. of subjects = 427 Number of obs = 427

No. of failures = 376

Time at risk = 84,167.5029

LR chi2(3) = 75.47

Log likelihood = -1909.1783 Prob > chi2 = 0.0000

------------------------------------------------------------------------------

_t | Haz. ratio Std. err. z P>|z| [95% conf. interval]

-------------+----------------------------------------------------------------

trtgrp | .602342 .0660077 -4.63 0.000 .4859193 .7466587

regionstrat | 1.028191 .071959 0.40 0.691 .896399 1.179359

becogstrat | 3.507203 .5217931 8.43 0.000 2.62012 4.694621

------------------------------------------------------------------------------

.

. restore

.

. ******************************************************************************************

> ***************

. *** TSEsimp 7 (primary analysis, reduced model, no recens, Weibull) Estimand 1 (Analysis 2

> 23 from TSEsimp)***

. ******************************************************************************************

> ***************

.

. preserve

.

. * note, 8 patients switched before investigator observed progression. 7 were kras MT, so p

> rimary analyses will not adjust for these anyway.

. * protocol suggested switching permitted only after progression. So assume these patients

> switched due to some signs of progression.

. * need this, because SNM models will fit much better if applied only to the time-periods w

> here switching was "permitted".

. * so, for these 8 patients, replace PDDYLR to equal xotime

. * and replace progtdc to = 1 after this point for these patients

. sort SUBJID dthdyxtdc

. by SUBJID: replace PDDYLR = xotime if (PDDYLR>xotime & xotime!=.)

(1,252 real changes made)

. by SUBJID: replace progtdc = 1 if (PDDYLR <= dthdyxtdc & PDLR==1)

(984 real changes made)

.

. replace xotdc = 0 if krasi==1 & trtgrp==1

(14,415 real changes made)

. replace xo = . if krasi==1 & trtgrp==1 & xo==1

(18,500 real changes made, 18,500 to missing)

.

. by SUBJID: replace lastobs = 0

(427 real changes made)

. by SUBJID: replace lastobs = 1 if _n==_N

(427 real changes made)

.

. *** streg

. sort SUBJID dthdyxtdc

. drop if trtgrp==2

(49,968 observations deleted)

. drop if progtdc==0

(10,866 observations deleted)

. * drop anyone who died on same day as progression

. drop if PDDYLR == deathtime

(16 observations deleted)

. * for primary analysis, get treatment effect comparing WT switchers to WT non-switchers, i

> .e. exclude MT

. drop if krasi==1

(16,044 observations deleted)

. by SUBJID: gen obsno = _n

. by SUBJID: gen trtnew = 0

. by SUBJID: replace trtnew = 1 if xo==1 & dthdyxtdc>=xotime

(22802 real changes made)

. by SUBJID: egen minrisk=min(time)

. by SUBJID: replace dthdyxtdc=dthdyxtdc-minrisk

(24923 real changes made)

. by SUBJID: replace xotime=xotime-minrisk

(24117 real changes made)

. by SUBJID: replace time=time-minrisk

(24923 real changes made)

. by SUBJID: replace admin=admin-minrisk

(24923 real changes made)

.

. stset dthdyxtdc, failure(deathtdc) id(SUBJID)

Survival-time data settings

ID variable: SUBJID

Failure event: deathtdc!=0 & deathtdc<.

Observed time interval: (dthdyxtdc[_n-1], dthdyxtdc]

Exit on or before: failure

--------------------------------------------------------------------------

24,923 total observations

0 exclusions

--------------------------------------------------------------------------

24,923 observations remaining, representing

106 subjects

100 failures in single-failure-per-subject data

24,923 total analysis time at risk and under observation

At risk from t = 0

Earliest observed entry t = 0

Last observed exit t = 784

. streg trtnew becogstrat diagtype eq5dbase eq5dmissb regionstrat PDDYLR eq5datprog eq5dind

> eq5dmissingatprog ecogatprog bestrespatprog respmissingatprog LSSLDatprog LSSLDmissingatpr

> og, dist(weibull) time iterate(200)

Failure _d: deathtdc

Analysis time _t: dthdyxtdc

ID variable: SUBJID

Fitting constant-only model:

Iteration 0: log likelihood = -155.00396

Iteration 1: log likelihood = -153.4072

Iteration 2: log likelihood = -153.40372

Iteration 3: log likelihood = -153.40372

Fitting full model:

Iteration 0: log likelihood = -153.40372

Iteration 1: log likelihood = -136.61921

Iteration 2: log likelihood = -124.21975

Iteration 3: log likelihood = -123.98834

Iteration 4: log likelihood = -123.98772

Iteration 5: log likelihood = -123.98772

Weibull AFT regression

No. of subjects = 106 Number of obs = 24,923

No. of failures = 100

Time at risk = 24,923

LR chi2(15) = 58.83

Log likelihood = -123.98772 Prob > chi2 = 0.0000

------------------------------------------------------------------------------------

_t | Coefficient Std. err. z P>|z| [95% conf. interval]

-------------------+----------------------------------------------------------------

trtnew | .9398798 .2216702 4.24 0.000 .5054141 1.374346

becogstrat | .2740033 .2863727 0.96 0.339 -.2872768 .8352835

diagtype | .0582467 .1484566 0.39 0.695 -.2327229 .3492162

eq5dbase | -.8388337 .3828969 -2.19 0.028 -1.589298 -.0883696

eq5dmissb | -.908241 .4555363 -1.99 0.046 -1.801076 -.0154063

regionstrat | -.0122116 .1021417 -0.12 0.905 -.2124056 .1879825

PDDYLR | .0104706 .003504 2.99 0.003 .003603 .0173383

eq5datprog | .1290311 .3465396 0.37 0.710 -.5501741 .8082363

eq5dind | -.3294473 .6215387 -0.53 0.596 -1.547641 .8887461

eq5dmissingatprog | .1331492 .2937642 0.45 0.650 -.4426181 .7089165

ecogatprog | -.4896557 .1358889 -3.60 0.000 -.7559931 -.2233184

bestrespatprog | .2451107 .1612552 1.52 0.129 -.0709438 .5611651

respmissingatprog | -.3421478 .2340022 -1.46 0.144 -.8007837 .1164881

LSSLDatprog | -.0013835 .0006297 -2.20 0.028 -.0026177 -.0001494

LSSLDmissingatprog | -.2766897 .1614798 -1.71 0.087 -.5931843 .0398049

_cons | 5.10881 .8325282 6.14 0.000 3.477085 6.740535

-------------------+----------------------------------------------------------------

/ln_p | .4852643 .0799426 6.07 0.000 .3285797 .6419489

-------------------+----------------------------------------------------------------

p | 1.624604 .1298751 1.388994 1.90018

1/p | .6155345 .0492074 .5262658 .7199456

------------------------------------------------------------------------------------

.

. * Same model as for IPCW denominator. Note, had to take ecogmisslastvisit out as !=0 perfe

> ctly predicted failure

.

. scalar tsec_af = exp(_b[trtnew])

. di tsec_af

2.5596738

.

. restore

. sort SUBJID

. preserve

.

. ***Analysis on overall survival***

. collapse (max) trtgrp krasi xo regionstrat becogstrat dthdyxtdc xotime deathtdc admin, by(

> SUBJID)

. by SUBJID: replace xotime=0 if xotime==.

(259 real changes made)

. by SUBJID: replace xotime = 0 if krasi==1 & trtgrp==1 & xo==1

(77 real changes made)

. by SUBJID: replace xo = 0 if krasi==1 & trtgrp==1 & xo==1

(77 real changes made)

.

. ***below no recensoring***

. gen cfact = dthdyxtdc if trtgrp==2

(219 missing values generated)

. gen dcfact = deathtdc if trtgrp==2

(219 missing values generated)

.

. replace cfact = (xotime + ((dthdyxtdc-xotime)/(tsec_af))) if (trtgrp==1 & xotime>0)

(91 real changes made)

. replace cfact = dthdyxtdc if (trtgrp==1 & xotime==0)

(128 real changes made)

. replace dcfact = deathtdc if trtgrp==1

(219 real changes made)

.

. ***do survival analysis on re-estimated survival times***

. stset cfact, failure(dcfact) id(SUBJID)

Survival-time data settings

ID variable: SUBJID

Failure event: dcfact!=0 & dcfact<.

Observed time interval: (cfact[_n-1], cfact]

Exit on or before: failure

--------------------------------------------------------------------------

427 total observations

0 exclusions

--------------------------------------------------------------------------

427 observations remaining, representing

427 subjects

391 failures in single-failure-per-subject data

87,978.615 total analysis time at risk and under observation

At risk from t = 0

Earliest observed entry t = 0

Last observed exit t = 1,024

.

. stcox trtgrp regionstrat becogstrat

Failure _d: dcfact

Analysis time _t: cfact

ID variable: SUBJID

Iteration 0: log likelihood = -2017.9591

Iteration 1: log likelihood = -1999.9982

Iteration 2: log likelihood = -1982.5235

Iteration 3: log likelihood = -1982.1932

Iteration 4: log likelihood = -1982.1929

Refining estimates:

Iteration 0: log likelihood = -1982.1929

Cox regression with Breslow method for ties

No. of subjects = 427 Number of obs = 427

No. of failures = 391

Time at risk = 87,978.6148

LR chi2(3) = 71.53

Log likelihood = -1982.1929 Prob > chi2 = 0.0000

------------------------------------------------------------------------------

_t | Haz. ratio Std. err. z P>|z| [95% conf. interval]

-------------+----------------------------------------------------------------

trtgrp | .6513055 .0668454 -4.18 0.000 .5326274 .7964271

regionstrat | 1.000301 .0693058 0.00 0.997 .8732837 1.145793

becogstrat | 3.513428 .5229985 8.44 0.000 2.624362 4.703687

------------------------------------------------------------------------------

.

. restore

.

. ******************************************************************************************

> ***************

. *** TSEsimp 8 (primary analysis, reduced model, no recens, Gen Gamma) Estimand 1 ***

. ******************************************************************************************

> ***************

.

. preserve

.

. * note, 8 patients switched before investigator observed progression. 7 were kras MT, so p

> rimary analyses will not adjust for these anyway.

. * protocol suggested switching permitted only after progression. So assume these patients

> switched due to some signs of progression.

. * need this, because SNM models will fit much better if applied only to the time-periods w

> here switching was "permitted".

. * so, for these 8 patients, replace PDDYLR to equal xotime

. * and replace progtdc to = 1 after this point for these patients

. sort SUBJID dthdyxtdc

. by SUBJID: replace PDDYLR = xotime if (PDDYLR>xotime & xotime!=.)

(1,252 real changes made)

. by SUBJID: replace progtdc = 1 if (PDDYLR <= dthdyxtdc & PDLR==1)

(984 real changes made)

.

. replace xotdc = 0 if krasi==1 & trtgrp==1

(14,415 real changes made)

. replace xo = . if krasi==1 & trtgrp==1 & xo==1

(18,500 real changes made, 18,500 to missing)

.

. by SUBJID: replace lastobs = 0

(427 real changes made)

. by SUBJID: replace lastobs = 1 if _n==_N

(427 real changes made)

.

. *** streg

. sort SUBJID dthdyxtdc

. drop if trtgrp==2

(49,968 observations deleted)

. drop if progtdc==0

(10,866 observations deleted)

. * drop anyone who died on same day as progression

. drop if PDDYLR == deathtime

(16 observations deleted)

. * for primary analysis, get treatment effect comparing WT switchers to WT non-switchers, i

> .e. exclude MT

. drop if krasi==1

(16,044 observations deleted)

. by SUBJID: gen obsno = _n

. by SUBJID: gen trtnew = 0

. by SUBJID: replace trtnew = 1 if xo==1 & dthdyxtdc>=xotime

(22802 real changes made)

. by SUBJID: egen minrisk=min(time)

. by SUBJID: replace dthdyxtdc=dthdyxtdc-minrisk

(24923 real changes made)

. by SUBJID: replace xotime=xotime-minrisk

(24117 real changes made)

. by SUBJID: replace time=time-minrisk

(24923 real changes made)

. by SUBJID: replace admin=admin-minrisk

(24923 real changes made)

.

. stset dthdyxtdc, failure(deathtdc) id(SUBJID)

Survival-time data settings

ID variable: SUBJID

Failure event: deathtdc!=0 & deathtdc<.

Observed time interval: (dthdyxtdc[_n-1], dthdyxtdc]

Exit on or before: failure

--------------------------------------------------------------------------

24,923 total observations

0 exclusions

--------------------------------------------------------------------------

24,923 observations remaining, representing

106 subjects

100 failures in single-failure-per-subject data

24,923 total analysis time at risk and under observation

At risk from t = 0

Earliest observed entry t = 0

Last observed exit t = 784

. streg trtnew becogstrat diagtype eq5dbase eq5dmissb regionstrat PDDYLR eq5datprog eq5dind

> eq5dmissingatprog ecogatprog bestrespatprog respmissingatprog LSSLDatprog LSSLDmissingatpr

> og, dist(ggamma) time iterate(200)

Failure _d: deathtdc

Analysis time _t: dthdyxtdc

ID variable: SUBJID

Fitting constant-only model:

Iteration 0: log likelihood = -580.23762 (not concave)

Iteration 1: log likelihood = -226.73916 (not concave)

Iteration 2: log likelihood = -160.10683

Iteration 3: log likelihood = -156.87958

Iteration 4: log likelihood = -153.47437

Iteration 5: log likelihood = -153.32015

Iteration 6: log likelihood = -153.31691

Iteration 7: log likelihood = -153.31691

Fitting full model:

Iteration 0: log likelihood = -153.31691 (not concave)

Iteration 1: log likelihood = -139.92273 (not concave)

Iteration 2: log likelihood = -134.69516 (not concave)

Iteration 3: log likelihood = -132.09858 (not concave)

Iteration 4: log likelihood = -129.45604

Iteration 5: log likelihood = -126.09495

Iteration 6: log likelihood = -124.22244

Iteration 7: log likelihood = -123.97936

Iteration 8: log likelihood = -123.96834

Iteration 9: log likelihood = -123.96833

Generalized gamma AFT regression

No. of subjects = 106 Number of obs = 24,923

No. of failures = 100

Time at risk = 24,923

LR chi2(15) = 58.70

Log likelihood = -123.96833 Prob > chi2 = 0.0000

------------------------------------------------------------------------------------

_t | Coefficient Std. err. z P>|z| [95% conf. interval]

-------------------+----------------------------------------------------------------

trtnew | .9202868 .2501288 3.68 0.000 .4300433 1.41053

becogstrat | .3145031 .3656033 0.86 0.390 -.4020662 1.031073

diagtype | .0811653 .1935202 0.42 0.675 -.2981274 .460458

eq5dbase | -.8475395 .3802808 -2.23 0.026 -1.592876 -.1022028

eq5dmissb | -.9329829 .4640518 -2.01 0.044 -1.842508 -.0234582

regionstrat | -.0296866 .1402058 -0.21 0.832 -.3044849 .2451116

PDDYLR | .0108213 .0039915 2.71 0.007 .0029982 .0186445

eq5datprog | .1081325 .3683005 0.29 0.769 -.6137232 .8299882

eq5dind | -.2872059 .6715222 -0.43 0.669 -1.603365 1.028953

eq5dmissingatprog | .154406 .3281451 0.47 0.638 -.4887465 .7975585

ecogatprog | -.498531 .1446274 -3.45 0.001 -.7819954 -.2150666

bestrespatprog | .2550848 .1673917 1.52 0.128 -.072997 .5831665

respmissingatprog | -.3347191 .2352875 -1.42 0.155 -.7958742 .126436

LSSLDatprog | -.0012841 .0008177 -1.57 0.116 -.0028867 .0003185

LSSLDmissingatprog | -.2729895 .1612406 -1.69 0.090 -.5890153 .0430364

_cons | 5.050585 .8878638 5.69 0.000 3.310404 6.790766

-------------------+----------------------------------------------------------------

/lnsigma | -.5277874 .2617886 -2.02 0.044 -1.040884 -.0146911

/kappa | 1.138928 .7880484 1.45 0.148 -.405618 2.683475

-------------------+----------------------------------------------------------------

sigma | .5899088 .1544314 .3531425 .9854163

------------------------------------------------------------------------------------

.

. * Same model as for IPCW denominator. Note, had to take ecogmisslastvisit out as !=0 perfe

> ctly predicted failure

.

. scalar tsec_af = exp(_b[trtnew])

. di tsec_af

2.5100102

.

. restore

. sort SUBJID

. preserve

.

. ***Analysis on overall survival***

. collapse (max) trtgrp krasi xo regionstrat becogstrat dthdyxtdc xotime deathtdc admin, by(

> SUBJID)

. by SUBJID: replace xotime=0 if xotime==.

(259 real changes made)

. by SUBJID: replace xotime = 0 if krasi==1 & trtgrp==1 & xo==1

(77 real changes made)

. by SUBJID: replace xo = 0 if krasi==1 & trtgrp==1 & xo==1

(77 real changes made)

.

. ***below no recensoring***

. gen cfact = dthdyxtdc if trtgrp==2

(219 missing values generated)

. gen dcfact = deathtdc if trtgrp==2

(219 missing values generated)

.

. replace cfact = (xotime + ((dthdyxtdc-xotime)/(tsec_af))) if (trtgrp==1 & xotime>0)

(91 real changes made)

. replace cfact = dthdyxtdc if (trtgrp==1 & xotime==0)

(128 real changes made)

. replace dcfact = deathtdc if trtgrp==1

(219 real changes made)

.

. ***do survival analysis on re-estimated survival times***

. stset cfact, failure(dcfact) id(SUBJID)

Survival-time data settings

ID variable: SUBJID

Failure event: dcfact!=0 & dcfact<.

Observed time interval: (cfact[_n-1], cfact]

Exit on or before: failure

--------------------------------------------------------------------------

427 total observations

0 exclusions

--------------------------------------------------------------------------

427 observations remaining, representing

427 subjects

391 failures in single-failure-per-subject data

88,154.17 total analysis time at risk and under observation

At risk from t = 0

Earliest observed entry t = 0

Last observed exit t = 1,024

.

. stcox trtgrp regionstrat becogstrat

Failure _d: dcfact

Analysis time _t: cfact

ID variable: SUBJID

Iteration 0: log likelihood = -2018.0343

Iteration 1: log likelihood = -2000.3523

Iteration 2: log likelihood = -1982.8721

Iteration 3: log likelihood = -1982.5402

Iteration 4: log likelihood = -1982.5399

Refining estimates:

Iteration 0: log likelihood = -1982.5399

Cox regression with Breslow method for ties

No. of subjects = 427 Number of obs = 427

No. of failures = 391

Time at risk = 88,154.1704

LR chi2(3) = 70.99

Log likelihood = -1982.5399 Prob > chi2 = 0.0000

------------------------------------------------------------------------------

_t | Haz. ratio Std. err. z P>|z| [95% conf. interval]

-------------+----------------------------------------------------------------

trtgrp | .655632 .06727 -4.11 0.000 .5361967 .801671

regionstrat | 1.000714 .0693161 0.01 0.992 .8736757 1.146225

becogstrat | 3.508228 .5220786 8.43 0.000 2.620691 4.696342

------------------------------------------------------------------------------

.

. restore

.

. ******************************************************************************************

> ***************

. *** TSEsimp 9 (secondary analysis, full model, with recens, Weibull) Estimand 1 [225 from

> TSEsimp file] ***

. ******************************************************************************************

> ***************

.

. preserve

.

. * note, 8 patients switched before investigator observed progression. 7 were kras MT, so p

> rimary analyses will not adjust for these anyway.

. * protocol suggested switching permitted only after progression. So assume these patients

> switched due to some signs of progression.

. * need this, because SNM models will fit much better if applied only to the time-periods w

> here switching was "permitted".

. * so, for these 8 patients, replace PDDYLR to equal xotime

. * and replace progtdc to = 1 after this point for these patients

. sort SUBJID dthdyxtdc

. by SUBJID: replace PDDYLR = xotime if (PDDYLR>xotime & xotime!=.)

(1,252 real changes made)

. by SUBJID: replace progtdc = 1 if (PDDYLR <= dthdyxtdc & PDLR==1)

(984 real changes made)

.

. replace xotdc = 0 if krasi==1 & trtgrp==1

(14,415 real changes made)

. replace xo = . if krasi==1 & trtgrp==1 & xo==1

(18,500 real changes made, 18,500 to missing)

.

. by SUBJID: replace lastobs = 0

(427 real changes made)

. by SUBJID: replace lastobs = 1 if _n==_N

(427 real changes made)

.

. *** Streg

. sort SUBJID dthdyxtdc

. drop if trtgrp==2

(49,968 observations deleted)

. drop if progtdc==0

(10,866 observations deleted)

. * drop anyone who died on same day as progression

. drop if PDDYLR == deathtime

(16 observations deleted)

. by SUBJID: gen obsno = _n

. by SUBJID: gen trtnew = 0

. by SUBJID: replace trtnew = 1 if xo==1 & dthdyxtdc>=xotime

(22802 real changes made)

. by SUBJID: egen minrisk=min(time)

. by SUBJID: replace dthdyxtdc=dthdyxtdc-minrisk

(40930 real changes made)

. by SUBJID: replace xotime=xotime-minrisk

(39325 real changes made)

. by SUBJID: replace time=time-minrisk

(40930 real changes made)

. by SUBJID: replace admin=admin-minrisk

(40930 real changes made)

.

. * now we're comparing switching in WT BSC to WT non-switchers AND BSC MT.

.

. stset dthdyxtdc, failure(deathtdc) id(SUBJID)

Survival-time data settings

ID variable: SUBJID

Failure event: deathtdc!=0 & deathtdc<.

Observed time interval: (dthdyxtdc[_n-1], dthdyxtdc]

Exit on or before: failure

--------------------------------------------------------------------------

40,967 total observations

0 exclusions

--------------------------------------------------------------------------

40,967 observations remaining, representing

199 subjects

189 failures in single-failure-per-subject data

40,967 total analysis time at risk and under observation

At risk from t = 0

Earliest observed entry t = 0

Last observed exit t = 784

. streg trtnew AGE becogstrat diagtype BILIULN ASTULN ALBULN LDHULN eq5dbase CEAULN eq5dmiss

> b regionstrat PDDYLR eq5datprog eq5dind eq5dmissingatprog ecogatprog bestrespatprog respmi

> ssingatprog LSSLDatprog LSSLDmissingatprog AATULNatprog AATmissingatprog ALBULNatprog ALBm

> issingatprog ALKULNatprog ALKmissingatprog ASTULNatprog ASTmissingatprog CEAULNatprog CEAm

> issingatprog CREATmissingatprog LDHULNatprog LDHmissingatprog BILIULNatprog BILImissingatp

> rog saeatprog, dist(weibull) time iterate(200)

Failure _d: deathtdc

Analysis time _t: dthdyxtdc

ID variable: SUBJID

note: ALBmissingatprog omitted because of collinearity.

note: ALKmissingatprog omitted because of collinearity.

note: ASTmissingatprog omitted because of collinearity.

note: CREATmissingatprog omitted because of collinearity.

note: LDHmissingatprog omitted because of collinearity.

note: BILImissingatprog omitted because of collinearity.

Fitting constant-only model:

Iteration 0: log likelihood = -296.89063

Iteration 1: log likelihood = -295.78605

Iteration 2: log likelihood = -295.78531

Iteration 3: log likelihood = -295.78531

Fitting full model:

Iteration 0: log likelihood = -295.78531

Iteration 1: log likelihood = -265.46545

Iteration 2: log likelihood = -226.34545

Iteration 3: log likelihood = -225.50329

Iteration 4: log likelihood = -225.49795

Iteration 5: log likelihood = -225.49795

Weibull AFT regression

No. of subjects = 199 Number of obs = 40,967

No. of failures = 189

Time at risk = 40,967

LR chi2(31) = 140.57

Log likelihood = -225.49795 Prob > chi2 = 0.0000

------------------------------------------------------------------------------------

_t | Coefficient Std. err. z P>|z| [95% conf. interval]

-------------------+----------------------------------------------------------------

trtnew | .5907008 .1090638 5.42 0.000 .3769397 .8044619

AGE | .0023342 .0050928 0.46 0.647 -.0076475 .0123159

becogstrat | -.0970586 .1943831 -0.50 0.618 -.4780424 .2839253

diagtype | -.0399297 .1126404 -0.35 0.723 -.2607009 .1808414

BILIULN | .0101438 .1973675 0.05 0.959 -.3766893 .396977

ASTULN | .0792041 .2038133 0.39 0.698 -.3202626 .4786709

ALBULN | -.6033309 .2233348 -2.70 0.007 -1.041059 -.1656028

LDHULN | .0173277 .2387567 0.07 0.942 -.4506269 .4852823

eq5dbase | -.6770544 .3015438 -2.25 0.025 -1.268069 -.0860396

CEAULN | -.3420254 .5670659 -0.60 0.546 -1.453454 .7694034

eq5dmissb | -.1275406 .4143498 -0.31 0.758 -.9396513 .6845702

regionstrat | .002904 .0767403 0.04 0.970 -.1475043 .1533123

PDDYLR | .0004668 .0022316 0.21 0.834 -.0039071 .0048407

eq5datprog | -.2133997 .3309501 -0.64 0.519 -.86205 .4352505

eq5dind | .5817992 .5245131 1.11 0.267 -.4462276 1.609826

eq5dmissingatprog | .2293841 .2404916 0.95 0.340 -.2419708 .7007391

ecogatprog | -.5630389 .097532 -5.77 0.000 -.7541981 -.3718796

bestrespatprog | -.0790805 .1259105 -0.63 0.530 -.3258606 .1676996

respmissingatprog | .0275174 .1625683 0.17 0.866 -.2911106 .3461454

LSSLDatprog | -.0015289 .0005857 -2.61 0.009 -.0026768 -.000381

LSSLDmissingatprog | -.2836148 .1234444 -2.30 0.022 -.5255614 -.0416681

AATULNatprog | -.052209 .15183 -0.34 0.731 -.3497904 .2453724

AATmissingatprog | -.2253102 .2019125 -1.12 0.264 -.6210514 .1704311

ALBULNatprog | .1918803 .2123017 0.90 0.366 -.2242235 .6079841

ALBmissingatprog | 0 (omitted)

ALKULNatprog | -.0539988 .1459427 -0.37 0.711 -.3400412 .2320436

ALKmissingatprog | 0 (omitted)

ASTULNatprog | -.3917853 .2236322 -1.75 0.080 -.8300964 .0465259

ASTmissingatprog | 0 (omitted)

CEAULNatprog | .3932288 .5792605 0.68 0.497 -.7421009 1.528558

CEAmissingatprog | .2195723 .1351222 1.62 0.104 -.0452623 .484407

CREATmissingatprog | 0 (omitted)

LDHULNatprog | -.2672082 .2506336 -1.07 0.286 -.7584411 .2240246

LDHmissingatprog | 0 (omitted)

BILIULNatprog | .0189546 .1927492 0.10 0.922 -.3588269 .3967362

BILImissingatprog | 0 (omitted)

saeatprog | -.0181394 .3618406 -0.05 0.960 -.7273341 .6910552

_cons | 6.368741 .7788874 8.18 0.000 4.842149 7.895332

-------------------+----------------------------------------------------------------

/ln_p | .4710848 .0580024 8.12 0.000 .3574023 .5847673

-------------------+----------------------------------------------------------------

p | 1.601731 .0929042 1.429611 1.794573

1/p | .6243246 .0362123 .5572355 .699491

------------------------------------------------------------------------------------

.

. * Same model as for IPCW denominator. Note, had to take ecogmisslastvisit out as !=0 perfe

> ctly predicted failure. Also removed CREATULN and CREATULNatprog due to crazy coefficients

> and SE

. * And, ALBmisslastvisit, ALKmisslastvisit, ASTmisslastvisit, CREATmisslastvisit, LDHmissla

> stvisit, BILImisslastvisit all omitted because of collinearity

.

. scalar tsec_af = exp(_b[trtnew])

. di tsec_af

1.8052532

. restore

. sort SUBJID

. preserve

.

. ***Analysis on overall survival***

. collapse (max) trtgrp krasi xo regionstrat becogstrat dthdyxtdc xotime deathtdc admin, by(

> SUBJID)

. by SUBJID: replace xotime=0 if xotime==.

(259 real changes made)

. by SUBJID: replace xotime = 0 if krasi==1 & trtgrp==1 & xo==1

(77 real changes made)

. by SUBJID: replace xo = 0 if krasi==1 & trtgrp==1 & xo==1

(77 real changes made)

.

. ***below allows for recensoring***

. gen cfact = dthdyxtdc if trtgrp==2

(219 missing values generated)

. gen dcfact = deathtdc if trtgrp==2

(219 missing values generated)

.

. replace cfact = (xotime + ((dthdyxtdc-xotime)/(tsec_af))) if (trtgrp==1 & xotime>0)

(91 real changes made)

. replace cfact = dthdyxtdc if (trtgrp==1 & xotime==0)

(128 real changes made)

. gen OSadminc = admin/(tsec_af) if (trtgrp==1 & (tsec_af)>1.00)

(208 missing values generated)

. replace dcfact = deathtdc if trtgrp==1

(219 real changes made)

. replace dcfact=0 if (OSadminc<=cfact & trtgrp==1)

(6 real changes made)

. replace cfact = OSadminc if (OSadminc<=cfact & trtgrp==1)

(20 real changes made)

.

. ***do survival analysis on re-estimated survival times***

. stset cfact, failure(dcfact) id(SUBJID)

Survival-time data settings

ID variable: SUBJID

Failure event: dcfact!=0 & dcfact<.

Observed time interval: (cfact[_n-1], cfact]

Exit on or before: failure

--------------------------------------------------------------------------

427 total observations

0 exclusions

--------------------------------------------------------------------------

427 observations remaining, representing

427 subjects

385 failures in single-failure-per-subject data

89,491.898 total analysis time at risk and under observation

At risk from t = 0

Earliest observed entry t = 0

Last observed exit t = 1,024

.

. stcox trtgrp regionstrat becogstrat

Failure _d: dcfact

Analysis time _t: cfact

ID variable: SUBJID

Iteration 0: log likelihood = -1990.9106

Iteration 1: log likelihood = -1976.3162

Iteration 2: log likelihood = -1958.7021

Iteration 3: log likelihood = -1958.3358

Iteration 4: log likelihood = -1958.3354

Refining estimates:

Iteration 0: log likelihood = -1958.3354

Cox regression with Breslow method for ties

No. of subjects = 427 Number of obs = 427

No. of failures = 385

Time at risk = 89,491.8981

LR chi2(3) = 65.15

Log likelihood = -1958.3354 Prob > chi2 = 0.0000

------------------------------------------------------------------------------

_t | Haz. ratio Std. err. z P>|z| [95% conf. interval]

-------------+----------------------------------------------------------------

trtgrp | .7020369 .0735406 -3.38 0.001 .5717339 .8620372

regionstrat | .9946646 .069514 -0.08 0.939 .8673389 1.140682

becogstrat | 3.436441 .5090285 8.33 0.000 2.570533 4.594037

------------------------------------------------------------------------------

.

. restore

.

. ******************************************************************************************

> ***************

. *** TSEsimp 10 (secondary analysis, full model, with recens, lognormal) Estimand 1 ***

. ******************************************************************************************

> ***************

.

. preserve

.

. * note, 8 patients switched before investigator observed progression. 7 were kras MT, so p

> rimary analyses will not adjust for these anyway.

. * protocol suggested switching permitted only after progression. So assume these patients

> switched due to some signs of progression.

. * need this, because SNM models will fit much better if applied only to the time-periods w

> here switching was "permitted".

. * so, for these 8 patients, replace PDDYLR to equal xotime

. * and replace progtdc to = 1 after this point for these patients

. sort SUBJID dthdyxtdc

. by SUBJID: replace PDDYLR = xotime if (PDDYLR>xotime & xotime!=.)

(1,252 real changes made)

. by SUBJID: replace progtdc = 1 if (PDDYLR <= dthdyxtdc & PDLR==1)

(984 real changes made)

.

. replace xotdc = 0 if krasi==1 & trtgrp==1

(14,415 real changes made)

. replace xo = . if krasi==1 & trtgrp==1 & xo==1

(18,500 real changes made, 18,500 to missing)

.

. by SUBJID: replace lastobs = 0

(427 real changes made)

. by SUBJID: replace lastobs = 1 if _n==_N

(427 real changes made)

.

. *** streg

. sort SUBJID dthdyxtdc

. drop if trtgrp==2

(49,968 observations deleted)

. drop if progtdc==0

(10,866 observations deleted)

. * drop anyone who died on same day as progression

. drop if PDDYLR == deathtime

(16 observations deleted)

. by SUBJID: gen obsno = _n

. by SUBJID: gen trtnew = 0

. by SUBJID: replace trtnew = 1 if xo==1 & dthdyxtdc>=xotime

(22802 real changes made)

. by SUBJID: egen minrisk=min(time)

. by SUBJID: replace dthdyxtdc=dthdyxtdc-minrisk

(40930 real changes made)

. by SUBJID: replace xotime=xotime-minrisk

(39325 real changes made)

. by SUBJID: replace time=time-minrisk

(40930 real changes made)

. by SUBJID: replace admin=admin-minrisk

(40930 real changes made)

.

. stset dthdyxtdc, failure(deathtdc) id(SUBJID)

Survival-time data settings

ID variable: SUBJID

Failure event: deathtdc!=0 & deathtdc<.

Observed time interval: (dthdyxtdc[_n-1], dthdyxtdc]

Exit on or before: failure

--------------------------------------------------------------------------

40,967 total observations

0 exclusions

--------------------------------------------------------------------------

40,967 observations remaining, representing

199 subjects

189 failures in single-failure-per-subject data

40,967 total analysis time at risk and under observation

At risk from t = 0

Earliest observed entry t = 0

Last observed exit t = 784

. streg trtnew AGE becogstrat diagtype BILIULN ASTULN ALBULN LDHULN eq5dbase CEAULN eq5dmiss

> b regionstrat PDDYLR eq5datprog eq5dind eq5dmissingatprog ecogatprog bestrespatprog respmi

> ssingatprog LSSLDatprog LSSLDmissingatprog AATULNatprog AATmissingatprog ALBULNatprog ALBm

> issingatprog ALKULNatprog ALKmissingatprog ASTULNatprog ASTmissingatprog CEAULNatprog CEAm

> issingatprog CREATmissingatprog LDHULNatprog LDHmissingatprog BILIULNatprog BILImissingatp

> rog saeatprog, dist(lognormal) time iterate(200)

Failure _d: deathtdc

Analysis time _t: dthdyxtdc

ID variable: SUBJID

note: ALBmissingatprog omitted because of collinearity.

note: ALKmissingatprog omitted because of collinearity.

note: ASTmissingatprog omitted because of collinearity.

note: CREATmissingatprog omitted because of collinearity.

note: LDHmissingatprog omitted because of collinearity.

note: BILImissingatprog omitted because of collinearity.

Fitting constant-only model:

Iteration 0: log likelihood = -382.12484

Iteration 1: log likelihood = -368.3964

Iteration 2: log likelihood = -301.72354

Iteration 3: log likelihood = -300.2444

Iteration 4: log likelihood = -300.24438

Fitting full model:

Iteration 0: log likelihood = -300.24438 (not concave)

Iteration 1: log likelihood = -249.45742

Iteration 2: log likelihood = -244.32187

Iteration 3: log likelihood = -233.1762

Iteration 4: log likelihood = -233.15376

Iteration 5: log likelihood = -233.15376

Lognormal AFT regression

No. of subjects = 199 Number of obs = 40,967

No. of failures = 189

Time at risk = 40,967

LR chi2(31) = 134.18

Log likelihood = -233.15376 Prob > chi2 = 0.0000

------------------------------------------------------------------------------------

_t | Coefficient Std. err. z P>|z| [95% conf. interval]

-------------------+----------------------------------------------------------------

trtnew | .5792934 .1250228 4.63 0.000 .3342532 .8243335

AGE | .0047406 .0059787 0.79 0.428 -.0069775 .0164586

becogstrat | -.1450553 .2306573 -0.63 0.529 -.5971354 .3070247

diagtype | .1113023 .1320935 0.84 0.399 -.1475961 .3702008

BILIULN | .2117962 .2286262 0.93 0.354 -.236303 .6598953

ASTULN | .0174814 .2181179 0.08 0.936 -.4100218 .4449847

ALBULN | -.4651265 .28158 -1.65 0.099 -1.017013 .0867601

LDHULN | -.1330151 .2642478 -0.50 0.615 -.6509312 .384901

eq5dbase | -.6465554 .3423404 -1.89 0.059 -1.31753 .0244194

CEAULN | .62903 .6674839 0.94 0.346 -.6792145 1.937274

eq5dmissb | -.0248735 .4663847 -0.05 0.957 -.9389707 .8892238

regionstrat | .0374664 .0893452 0.42 0.675 -.1376469 .2125798

PDDYLR | .0032216 .002826 1.14 0.254 -.0023173 .0087604

eq5datprog | .1447121 .3533371 0.41 0.682 -.5478159 .8372402

eq5dind | .355763 .5902788 0.60 0.547 -.8011623 1.512688

eq5dmissingatprog | .1250215 .2647598 0.47 0.637 -.3938982 .6439411

ecogatprog | -.5254142 .1096986 -4.79 0.000 -.7404194 -.3104089

bestrespatprog | -.0043501 .1363084 -0.03 0.975 -.2715095 .2628094

respmissingatprog | -.0808645 .1843651 -0.44 0.661 -.4422135 .2804845

LSSLDatprog | -.0012891 .0006631 -1.94 0.052 -.0025887 .0000106

LSSLDmissingatprog | -.2217882 .1400761 -1.58 0.113 -.4963323 .0527559

AATULNatprog | -.0311472 .1633063 -0.19 0.849 -.3512217 .2889273

AATmissingatprog | -.133214 .2317506 -0.57 0.565 -.587437 .3210089

ALBULNatprog | .0458205 .2635714 0.17 0.862 -.47077 .562411

ALBmissingatprog | 0 (omitted)

ALKULNatprog | -.0764007 .1590405 -0.48 0.631 -.3881144 .2353129

ALKmissingatprog | 0 (omitted)

ASTULNatprog | -.4228104 .2384677 -1.77 0.076 -.8901985 .0445778

ASTmissingatprog | 0 (omitted)

CEAULNatprog | -.5967751 .6781085 -0.88 0.379 -1.925843 .7322932

CEAmissingatprog | .1263866 .1599176 0.79 0.429 -.1870461 .4398193

CREATmissingatprog | 0 (omitted)

LDHULNatprog | .0066299 .2749009 0.02 0.981 -.5321659 .5454257

LDHmissingatprog | 0 (omitted)

BILIULNatprog | -.217063 .2242505 -0.97 0.333 -.6565859 .2224599

BILImissingatprog | 0 (omitted)

saeatprog | .1344786 .4362247 0.31 0.758 -.720506 .9894633

_cons | 5.242157 .8846816 5.93 0.000 3.508213 6.976101

-------------------+----------------------------------------------------------------

/lnsigma | -.2408314 .051049 -4.72 0.000 -.3408856 -.1407772

-------------------+----------------------------------------------------------------

sigma | .7859741 .0401232 .7111402 .8686828

------------------------------------------------------------------------------------

.

. * Same model as for IPCW denominator except for TDC variables. Note, had to take ecogmissl

> astvisit out as !=0 perfectly predicted failure. Also removed CREATULN and CREATULNatprog

> due to crazy coefficients and SE

. * And, ALBmisslastvisit, ALKmisslastvisit, ASTmisslastvisit, CREATmisslastvisit, LDHmissla

> stvisit, BILImisslastvisit all omitted because of collinearity

.

. scalar tsec_af = exp(_b[trtnew])

. di tsec_af

1.7847768

. restore

. sort SUBJID

. preserve

.

. ***Analysis on overall survival***

. collapse (max) trtgrp krasi xo regionstrat becogstrat dthdyxtdc xotime deathtdc admin, by(

> SUBJID)

. by SUBJID: replace xotime=0 if xotime==.

(259 real changes made)

. by SUBJID: replace xotime = 0 if krasi==1 & trtgrp==1 & xo==1

(77 real changes made)

. by SUBJID: replace xo = 0 if krasi==1 & trtgrp==1 & xo==1

(77 real changes made)

.

. ***below allows for recensoring***

. gen cfact = dthdyxtdc if trtgrp==2

(219 missing values generated)

. gen dcfact = deathtdc if trtgrp==2

(219 missing values generated)

.

. replace cfact = (xotime + ((dthdyxtdc-xotime)/(tsec_af))) if (trtgrp==1 & xotime>0)

(91 real changes made)

. replace cfact = dthdyxtdc if (trtgrp==1 & xotime==0)

(128 real changes made)

. gen OSadminc = admin/(tsec_af) if (trtgrp==1 & (tsec_af)>1.00)

(208 missing values generated)

. replace dcfact = deathtdc if trtgrp==1

(219 real changes made)

. replace dcfact=0 if (OSadminc<=cfact & trtgrp==1)

(6 real changes made)

. replace cfact = OSadminc if (OSadminc<=cfact & trtgrp==1)

(20 real changes made)

.

. ***do survival analysis on re-estimated survival times***

. stset cfact, failure(dcfact) id(SUBJID)

Survival-time data settings

ID variable: SUBJID

Failure event: dcfact!=0 & dcfact<.

Observed time interval: (cfact[_n-1], cfact]

Exit on or before: failure

--------------------------------------------------------------------------

427 total observations

0 exclusions

--------------------------------------------------------------------------

427 observations remaining, representing

427 subjects

385 failures in single-failure-per-subject data

89,691.477 total analysis time at risk and under observation

At risk from t = 0

Earliest observed entry t = 0

Last observed exit t = 1,024

.

. stcox trtgrp regionstrat becogstrat

Failure _d: dcfact

Analysis time _t: cfact

ID variable: SUBJID

Iteration 0: log likelihood = -1991.091

Iteration 1: log likelihood = -1976.7116

Iteration 2: log likelihood = -1959.0202

Iteration 3: log likelihood = -1958.6522

Iteration 4: log likelihood = -1958.6518

Refining estimates:

Iteration 0: log likelihood = -1958.6518

Cox regression with Breslow method for ties

No. of subjects = 427 Number of obs = 427

No. of failures = 385

Time at risk = 89,691.4775

LR chi2(3) = 64.88

Log likelihood = -1958.6518 Prob > chi2 = 0.0000

------------------------------------------------------------------------------

_t | Haz. ratio Std. err. z P>|z| [95% conf. interval]

-------------+----------------------------------------------------------------

trtgrp | .706101 .0739192 -3.32 0.001 .5751188 .8669142

regionstrat | .9951777 .0695535 -0.07 0.945 .8677802 1.141278

becogstrat | 3.440269 .5096469 8.34 0.000 2.573322 4.59929

------------------------------------------------------------------------------

.

. restore

.

. ******************************************************************************************

> ***************

. *** TSEsimp 11 (secondary analysis, full model, no recens, Weibull) Estimand 1 [227 from T

> SEsimp file] ***

. ******************************************************************************************

> ***************

.

. preserve

.

. * note, 8 patients switched before investigator observed progression. 7 were kras MT, so p

> rimary analyses will not adjust for these anyway.

. * protocol suggested switching permitted only after progression. So assume these patients

> switched due to some signs of progression.

. * need this, because SNM models will fit much better if applied only to the time-periods w

> here switching was "permitted".

. * so, for these 8 patients, replace PDDYLR to equal xotime

. * and replace progtdc to = 1 after this point for these patients

. sort SUBJID dthdyxtdc

. by SUBJID: replace PDDYLR = xotime if (PDDYLR>xotime & xotime!=.)

(1,252 real changes made)

. by SUBJID: replace progtdc = 1 if (PDDYLR <= dthdyxtdc & PDLR==1)

(984 real changes made)

.

. replace xotdc = 0 if krasi==1 & trtgrp==1

(14,415 real changes made)

. replace xo = . if krasi==1 & trtgrp==1 & xo==1

(18,500 real changes made, 18,500 to missing)

.

. by SUBJID: replace lastobs = 0

(427 real changes made)

. by SUBJID: replace lastobs = 1 if _n==_N

(427 real changes made)

.

. *** streg

. sort SUBJID dthdyxtdc

. drop if trtgrp==2

(49,968 observations deleted)

. drop if progtdc==0

(10,866 observations deleted)

. * drop anyone who died on same day as progression

. drop if PDDYLR == deathtime

(16 observations deleted)

. by SUBJID: gen obsno = _n

. by SUBJID: gen trtnew = 0

. by SUBJID: replace trtnew = 1 if xo==1 & dthdyxtdc>=xotime

(22802 real changes made)

. by SUBJID: egen minrisk=min(time)

. by SUBJID: replace dthdyxtdc=dthdyxtdc-minrisk

(40930 real changes made)

. by SUBJID: replace xotime=xotime-minrisk

(39325 real changes made)

. by SUBJID: replace time=time-minrisk

(40930 real changes made)

. by SUBJID: replace admin=admin-minrisk

(40930 real changes made)

.

. stset dthdyxtdc, failure(deathtdc) id(SUBJID)

Survival-time data settings

ID variable: SUBJID

Failure event: deathtdc!=0 & deathtdc<.

Observed time interval: (dthdyxtdc[_n-1], dthdyxtdc]

Exit on or before: failure

--------------------------------------------------------------------------

40,967 total observations

0 exclusions

--------------------------------------------------------------------------

40,967 observations remaining, representing

199 subjects

189 failures in single-failure-per-subject data

40,967 total analysis time at risk and under observation

At risk from t = 0

Earliest observed entry t = 0

Last observed exit t = 784

. streg trtnew AGE becogstrat diagtype BILIULN ASTULN ALBULN LDHULN eq5dbase CEAULN eq5dmiss

> b regionstrat PDDYLR eq5datprog eq5dind eq5dmissingatprog ecogatprog bestrespatprog respmi

> ssingatprog LSSLDatprog LSSLDmissingatprog AATULNatprog AATmissingatprog ALBULNatprog ALBm

> issingatprog ALKULNatprog ALKmissingatprog ASTULNatprog ASTmissingatprog CEAULNatprog CEAm

> issingatprog CREATmissingatprog LDHULNatprog LDHmissingatprog BILIULNatprog BILImissingatp

> rog saeatprog, dist(weibull) time iterate(200)

Failure _d: deathtdc

Analysis time _t: dthdyxtdc

ID variable: SUBJID

note: ALBmissingatprog omitted because of collinearity.

note: ALKmissingatprog omitted because of collinearity.

note: ASTmissingatprog omitted because of collinearity.

note: CREATmissingatprog omitted because of collinearity.

note: LDHmissingatprog omitted because of collinearity.

note: BILImissingatprog omitted because of collinearity.

Fitting constant-only model:

Iteration 0: log likelihood = -296.89063

Iteration 1: log likelihood = -295.78605

Iteration 2: log likelihood = -295.78531

Iteration 3: log likelihood = -295.78531

Fitting full model:

Iteration 0: log likelihood = -295.78531

Iteration 1: log likelihood = -265.46545

Iteration 2: log likelihood = -226.34545

Iteration 3: log likelihood = -225.50329

Iteration 4: log likelihood = -225.49795

Iteration 5: log likelihood = -225.49795

Weibull AFT regression

No. of subjects = 199 Number of obs = 40,967

No. of failures = 189

Time at risk = 40,967

LR chi2(31) = 140.57

Log likelihood = -225.49795 Prob > chi2 = 0.0000

------------------------------------------------------------------------------------

_t | Coefficient Std. err. z P>|z| [95% conf. interval]

-------------------+----------------------------------------------------------------

trtnew | .5907008 .1090638 5.42 0.000 .3769397 .8044619

AGE | .0023342 .0050928 0.46 0.647 -.0076475 .0123159

becogstrat | -.0970586 .1943831 -0.50 0.618 -.4780424 .2839253

diagtype | -.0399297 .1126404 -0.35 0.723 -.2607009 .1808414

BILIULN | .0101438 .1973675 0.05 0.959 -.3766893 .396977

ASTULN | .0792041 .2038133 0.39 0.698 -.3202626 .4786709

ALBULN | -.6033309 .2233348 -2.70 0.007 -1.041059 -.1656028

LDHULN | .0173277 .2387567 0.07 0.942 -.4506269 .4852823

eq5dbase | -.6770544 .3015438 -2.25 0.025 -1.268069 -.0860396

CEAULN | -.3420254 .5670659 -0.60 0.546 -1.453454 .7694034

eq5dmissb | -.1275406 .4143498 -0.31 0.758 -.9396513 .6845702

regionstrat | .002904 .0767403 0.04 0.970 -.1475043 .1533123

PDDYLR | .0004668 .0022316 0.21 0.834 -.0039071 .0048407

eq5datprog | -.2133997 .3309501 -0.64 0.519 -.86205 .4352505

eq5dind | .5817992 .5245131 1.11 0.267 -.4462276 1.609826

eq5dmissingatprog | .2293841 .2404916 0.95 0.340 -.2419708 .7007391

ecogatprog | -.5630389 .097532 -5.77 0.000 -.7541981 -.3718796

bestrespatprog | -.0790805 .1259105 -0.63 0.530 -.3258606 .1676996

respmissingatprog | .0275174 .1625683 0.17 0.866 -.2911106 .3461454

LSSLDatprog | -.0015289 .0005857 -2.61 0.009 -.0026768 -.000381

LSSLDmissingatprog | -.2836148 .1234444 -2.30 0.022 -.5255614 -.0416681

AATULNatprog | -.052209 .15183 -0.34 0.731 -.3497904 .2453724

AATmissingatprog | -.2253102 .2019125 -1.12 0.264 -.6210514 .1704311

ALBULNatprog | .1918803 .2123017 0.90 0.366 -.2242235 .6079841

ALBmissingatprog | 0 (omitted)

ALKULNatprog | -.0539988 .1459427 -0.37 0.711 -.3400412 .2320436

ALKmissingatprog | 0 (omitted)

ASTULNatprog | -.3917853 .2236322 -1.75 0.080 -.8300964 .0465259

ASTmissingatprog | 0 (omitted)

CEAULNatprog | .3932288 .5792605 0.68 0.497 -.7421009 1.528558

CEAmissingatprog | .2195723 .1351222 1.62 0.104 -.0452623 .484407

CREATmissingatprog | 0 (omitted)

LDHULNatprog | -.2672082 .2506336 -1.07 0.286 -.7584411 .2240246

LDHmissingatprog | 0 (omitted)

BILIULNatprog | .0189546 .1927492 0.10 0.922 -.3588269 .3967362

BILImissingatprog | 0 (omitted)

saeatprog | -.0181394 .3618406 -0.05 0.960 -.7273341 .6910552

_cons | 6.368741 .7788874 8.18 0.000 4.842149 7.895332

-------------------+----------------------------------------------------------------

/ln_p | .4710848 .0580024 8.12 0.000 .3574023 .5847673

-------------------+----------------------------------------------------------------

p | 1.601731 .0929042 1.429611 1.794573

1/p | .6243246 .0362123 .5572355 .699491

------------------------------------------------------------------------------------

.

. * Same model as for IPCW denominator except for TDC variables. Note, had to take ecogmissl

> astvisit out as !=0 perfectly predicted failure. Also removed CREATULN and CREATULNatprog

> due to crazy coefficients and SE

. * And, ALBmisslastvisit, ALKmisslastvisit, ASTmisslastvisit, CREATmisslastvisit, LDHmissla

> stvisit, BILImisslastvisit all omitted because of collinearity

.

. scalar tsec_af = exp(_b[trtnew])

. di tsec_af

1.8052532

.

. restore

. sort SUBJID

. preserve

.

. ***Analysis on overall survival***

. collapse (max) trtgrp krasi xo regionstrat becogstrat dthdyxtdc xotime deathtdc admin, by(

> SUBJID)

. by SUBJID: replace xotime=0 if xotime==.

(259 real changes made)

. by SUBJID: replace xotime = 0 if krasi==1 & trtgrp==1 & xo==1

(77 real changes made)

. by SUBJID: replace xo = 0 if krasi==1 & trtgrp==1 & xo==1

(77 real changes made)

.

. ***below no recensoring***

. gen cfact = dthdyxtdc if trtgrp==2

(219 missing values generated)

. gen dcfact = deathtdc if trtgrp==2

(219 missing values generated)

.

. replace cfact = (xotime + ((dthdyxtdc-xotime)/(tsec_af))) if (trtgrp==1 & xotime>0)

(91 real changes made)

. replace cfact = dthdyxtdc if (trtgrp==1 & xotime==0)

(128 real changes made)

. replace dcfact = deathtdc if trtgrp==1

(219 real changes made)

.

. ***do survival analysis on re-estimated survival times***

. stset cfact, failure(dcfact) id(SUBJID)

Survival-time data settings

ID variable: SUBJID

Failure event: dcfact!=0 & dcfact<.

Observed time interval: (cfact[_n-1], cfact]

Exit on or before: failure

--------------------------------------------------------------------------

427 total observations

0 exclusions

--------------------------------------------------------------------------

427 observations remaining, representing

427 subjects

391 failures in single-failure-per-subject data

91,686.507 total analysis time at risk and under observation

At risk from t = 0

Earliest observed entry t = 0

Last observed exit t = 1,024

.

. stcox trtgrp regionstrat becogstrat

Failure _d: dcfact

Analysis time _t: cfact

ID variable: SUBJID

Iteration 0: log likelihood = -2019.8346

Iteration 1: log likelihood = -2006.512

Iteration 2: log likelihood = -1988.7711

Iteration 3: log likelihood = -1988.4019

Iteration 4: log likelihood = -1988.4015

Refining estimates:

Iteration 0: log likelihood = -1988.4015

Cox regression with Breslow method for ties

No. of subjects = 427 Number of obs = 427

No. of failures = 391

Time at risk = 91,686.5071

LR chi2(3) = 62.87

Log likelihood = -1988.4015 Prob > chi2 = 0.0000

------------------------------------------------------------------------------

_t | Haz. ratio Std. err. z P>|z| [95% conf. interval]

-------------+----------------------------------------------------------------

trtgrp | .7360042 .0751362 -3.00 0.003 .6025373 .899035

regionstrat | .9995229 .0690493 -0.01 0.994 .8729509 1.144447

becogstrat | 3.442208 .5098943 8.34 0.000 2.57483 4.601778

------------------------------------------------------------------------------

.

. restore

.

. ******************************************************************************************

> ***************

. *** TSEsimp 12 (secondary analysis, full model, no recens, lognormal) Estimand 1 ***

. ******************************************************************************************

> ***************

.

. preserve

.

. * note, 8 patients switched before investigator observed progression. 7 were kras MT, so p

> rimary analyses will not adjust for these anyway.

. * protocol suggested switching permitted only after progression. So assume these patients

> switched due to some signs of progression.

. * need this, because SNM models will fit much better if applied only to the time-periods w

> here switching was "permitted".

. * so, for these 8 patients, replace PDDYLR to equal xotime

. * and replace progtdc to = 1 after this point for these patients

. sort SUBJID dthdyxtdc

. by SUBJID: replace PDDYLR = xotime if (PDDYLR>xotime & xotime!=.)

(1,252 real changes made)

. by SUBJID: replace progtdc = 1 if (PDDYLR <= dthdyxtdc & PDLR==1)

(984 real changes made)

.

. replace xotdc = 0 if krasi==1 & trtgrp==1

(14,415 real changes made)

. replace xo = . if krasi==1 & trtgrp==1 & xo==1

(18,500 real changes made, 18,500 to missing)

.

. by SUBJID: replace lastobs = 0

(427 real changes made)

. by SUBJID: replace lastobs = 1 if _n==_N

(427 real changes made)

.

. *** streg

. sort SUBJID dthdyxtdc

. drop if trtgrp==2

(49,968 observations deleted)

. drop if progtdc==0

(10,866 observations deleted)

. * drop anyone who died on same day as progression

. drop if PDDYLR == deathtime

(16 observations deleted)

. by SUBJID: gen obsno = _n

. by SUBJID: gen trtnew = 0

. by SUBJID: replace trtnew = 1 if xo==1 & dthdyxtdc>=xotime

(22802 real changes made)

. by SUBJID: egen minrisk=min(time)

. by SUBJID: replace dthdyxtdc=dthdyxtdc-minrisk

(40930 real changes made)

. by SUBJID: replace xotime=xotime-minrisk

(39325 real changes made)

. by SUBJID: replace time=time-minrisk

(40930 real changes made)

. by SUBJID: replace admin=admin-minrisk

(40930 real changes made)

.

. stset dthdyxtdc, failure(deathtdc) id(SUBJID)

Survival-time data settings

ID variable: SUBJID

Failure event: deathtdc!=0 & deathtdc<.

Observed time interval: (dthdyxtdc[_n-1], dthdyxtdc]

Exit on or before: failure

--------------------------------------------------------------------------

40,967 total observations

0 exclusions

--------------------------------------------------------------------------

40,967 observations remaining, representing

199 subjects

189 failures in single-failure-per-subject data

40,967 total analysis time at risk and under observation

At risk from t = 0

Earliest observed entry t = 0

Last observed exit t = 784

. streg trtnew AGE becogstrat diagtype BILIULN ASTULN ALBULN LDHULN eq5dbase CEAULN eq5dmiss

> b regionstrat PDDYLR eq5datprog eq5dind eq5dmissingatprog ecogatprog bestrespatprog respmi

> ssingatprog LSSLDatprog LSSLDmissingatprog AATULNatprog AATmissingatprog ALBULNatprog ALBm

> issingatprog ALKULNatprog ALKmissingatprog ASTULNatprog ASTmissingatprog CEAULNatprog CEAm

> issingatprog CREATmissingatprog LDHULNatprog LDHmissingatprog BILIULNatprog BILImissingatp

> rog saeatprog, dist(lognormal) time iterate(200)

Failure _d: deathtdc

Analysis time _t: dthdyxtdc

ID variable: SUBJID

note: ALBmissingatprog omitted because of collinearity.

note: ALKmissingatprog omitted because of collinearity.

note: ASTmissingatprog omitted because of collinearity.

note: CREATmissingatprog omitted because of collinearity.

note: LDHmissingatprog omitted because of collinearity.

note: BILImissingatprog omitted because of collinearity.

Fitting constant-only model:

Iteration 0: log likelihood = -382.12484

Iteration 1: log likelihood = -368.3964

Iteration 2: log likelihood = -301.72354

Iteration 3: log likelihood = -300.2444

Iteration 4: log likelihood = -300.24438

Fitting full model:

Iteration 0: log likelihood = -300.24438 (not concave)

Iteration 1: log likelihood = -249.45742

Iteration 2: log likelihood = -244.32187

Iteration 3: log likelihood = -233.1762

Iteration 4: log likelihood = -233.15376

Iteration 5: log likelihood = -233.15376

Lognormal AFT regression

No. of subjects = 199 Number of obs = 40,967

No. of failures = 189

Time at risk = 40,967

LR chi2(31) = 134.18

Log likelihood = -233.15376 Prob > chi2 = 0.0000

------------------------------------------------------------------------------------

_t | Coefficient Std. err. z P>|z| [95% conf. interval]

-------------------+----------------------------------------------------------------

trtnew | .5792934 .1250228 4.63 0.000 .3342532 .8243335

AGE | .0047406 .0059787 0.79 0.428 -.0069775 .0164586

becogstrat | -.1450553 .2306573 -0.63 0.529 -.5971354 .3070247

diagtype | .1113023 .1320935 0.84 0.399 -.1475961 .3702008

BILIULN | .2117962 .2286262 0.93 0.354 -.236303 .6598953

ASTULN | .0174814 .2181179 0.08 0.936 -.4100218 .4449847

ALBULN | -.4651265 .28158 -1.65 0.099 -1.017013 .0867601

LDHULN | -.1330151 .2642478 -0.50 0.615 -.6509312 .384901

eq5dbase | -.6465554 .3423404 -1.89 0.059 -1.31753 .0244194

CEAULN | .62903 .6674839 0.94 0.346 -.6792145 1.937274

eq5dmissb | -.0248735 .4663847 -0.05 0.957 -.9389707 .8892238

regionstrat | .0374664 .0893452 0.42 0.675 -.1376469 .2125798

PDDYLR | .0032216 .002826 1.14 0.254 -.0023173 .0087604

eq5datprog | .1447121 .3533371 0.41 0.682 -.5478159 .8372402

eq5dind | .355763 .5902788 0.60 0.547 -.8011623 1.512688

eq5dmissingatprog | .1250215 .2647598 0.47 0.637 -.3938982 .6439411

ecogatprog | -.5254142 .1096986 -4.79 0.000 -.7404194 -.3104089

bestrespatprog | -.0043501 .1363084 -0.03 0.975 -.2715095 .2628094

respmissingatprog | -.0808645 .1843651 -0.44 0.661 -.4422135 .2804845

LSSLDatprog | -.0012891 .0006631 -1.94 0.052 -.0025887 .0000106

LSSLDmissingatprog | -.2217882 .1400761 -1.58 0.113 -.4963323 .0527559

AATULNatprog | -.0311472 .1633063 -0.19 0.849 -.3512217 .2889273

AATmissingatprog | -.133214 .2317506 -0.57 0.565 -.587437 .3210089

ALBULNatprog | .0458205 .2635714 0.17 0.862 -.47077 .562411

ALBmissingatprog | 0 (omitted)

ALKULNatprog | -.0764007 .1590405 -0.48 0.631 -.3881144 .2353129

ALKmissingatprog | 0 (omitted)

ASTULNatprog | -.4228104 .2384677 -1.77 0.076 -.8901985 .0445778

ASTmissingatprog | 0 (omitted)

CEAULNatprog | -.5967751 .6781085 -0.88 0.379 -1.925843 .7322932

CEAmissingatprog | .1263866 .1599176 0.79 0.429 -.1870461 .4398193

CREATmissingatprog | 0 (omitted)

LDHULNatprog | .0066299 .2749009 0.02 0.981 -.5321659 .5454257

LDHmissingatprog | 0 (omitted)

BILIULNatprog | -.217063 .2242505 -0.97 0.333 -.6565859 .2224599

BILImissingatprog | 0 (omitted)

saeatprog | .1344786 .4362247 0.31 0.758 -.720506 .9894633

_cons | 5.242157 .8846816 5.93 0.000 3.508213 6.976101

-------------------+----------------------------------------------------------------

/lnsigma | -.2408314 .051049 -4.72 0.000 -.3408856 -.1407772

-------------------+----------------------------------------------------------------

sigma | .7859741 .0401232 .7111402 .8686828

------------------------------------------------------------------------------------

.

. * Same model as for IPCW denominator except for TDC variables. Note, had to take ecogmissl

> astvisit out as !=0 perfectly predicted failure. Also removed CREATULN and CREATULNatprog

> due to crazy coefficients and SE

. * And, ALBmisslastvisit, ALKmisslastvisit, ASTmisslastvisit, CREATmisslastvisit, LDHmissla

> stvisit, BILImisslastvisit all omitted because of collinearity

.

. scalar tsec_af = exp(_b[trtnew])

. di tsec_af

1.7847768

.

. restore

. sort SUBJID

. preserve

.

. ***Analysis on overall survival***

. collapse (max) trtgrp krasi xo regionstrat becogstrat dthdyxtdc xotime deathtdc admin, by(

> SUBJID)

. by SUBJID: replace xotime=0 if xotime==.

(259 real changes made)

. by SUBJID: replace xotime = 0 if krasi==1 & trtgrp==1 & xo==1

(77 real changes made)

. by SUBJID: replace xo = 0 if krasi==1 & trtgrp==1 & xo==1

(77 real changes made)

.

. ***below no recensoring***

. gen cfact = dthdyxtdc if trtgrp==2

(219 missing values generated)

. gen dcfact = deathtdc if trtgrp==2

(219 missing values generated)

.

. replace cfact = (xotime + ((dthdyxtdc-xotime)/(tsec_af))) if (trtgrp==1 & xotime>0)

(91 real changes made)

. replace cfact = dthdyxtdc if (trtgrp==1 & xotime==0)

(128 real changes made)

. replace dcfact = deathtdc if trtgrp==1

(219 real changes made)

.

. ***do survival analysis on re-estimated survival times***

. stset cfact, failure(dcfact) id(SUBJID)

Survival-time data settings

ID variable: SUBJID

Failure event: dcfact!=0 & dcfact<.

Observed time interval: (cfact[_n-1], cfact]

Exit on or before: failure

--------------------------------------------------------------------------

427 total observations

0 exclusions

--------------------------------------------------------------------------

427 observations remaining, representing

427 subjects

391 failures in single-failure-per-subject data

91,830.84 total analysis time at risk and under observation

At risk from t = 0

Earliest observed entry t = 0

Last observed exit t = 1,024

.

. stcox trtgrp regionstrat becogstrat

Failure _d: dcfact

Analysis time _t: cfact

ID variable: SUBJID

Iteration 0: log likelihood = -2019.8026

Iteration 1: log likelihood = -2006.6121

Iteration 2: log likelihood = -1988.801

Iteration 3: log likelihood = -1988.4304

Iteration 4: log likelihood = -1988.4299

Refining estimates:

Iteration 0: log likelihood = -1988.4299

Cox regression with Breslow method for ties

No. of subjects = 427 Number of obs = 427

No. of failures = 391

Time at risk = 91,830.8404

LR chi2(3) = 62.75

Log likelihood = -1988.4299 Prob > chi2 = 0.0000

------------------------------------------------------------------------------

_t | Haz. ratio Std. err. z P>|z| [95% conf. interval]

-------------+----------------------------------------------------------------

trtgrp | .7385362 .0753885 -2.97 0.003 .6046201 .9021131

regionstrat | .999805 .069064 -0.00 0.998 .8732056 1.144759

becogstrat | 3.445753 .5104724 8.35 0.000 2.577405 4.606655

------------------------------------------------------------------------------

.

. restore

.

. ******************************************************************************************

> ***************

. *** TSEsimp 13 (secondary analysis, reduced model, with recens, Weibull) Estimand 1 [229 f

> rom TSEsimp file] ***

. ******************************************************************************************

> ***************

.

. preserve

.

. * note, 8 patients switched before investigator observed progression. 7 were kras MT, so p

> rimary analyses will not adjust for these anyway.

. * protocol suggested switching permitted only after progression. So assume these patients

> switched due to some signs of progression.

. * need this, because SNM models will fit much better if applied only to the time-periods w

> here switching was "permitted".

. * so, for these 8 patients, replace PDDYLR to equal xotime

. * and replace progtdc to = 1 after this point for these patients

. sort SUBJID dthdyxtdc

. by SUBJID: replace PDDYLR = xotime if (PDDYLR>xotime & xotime!=.)

(1,252 real changes made)

. by SUBJID: replace progtdc = 1 if (PDDYLR <= dthdyxtdc & PDLR==1)

(984 real changes made)

.

. replace xotdc = 0 if krasi==1 & trtgrp==1

(14,415 real changes made)

. replace xo = . if krasi==1 & trtgrp==1 & xo==1

(18,500 real changes made, 18,500 to missing)

.

. by SUBJID: replace lastobs = 0

(427 real changes made)

. by SUBJID: replace lastobs = 1 if _n==_N

(427 real changes made)

.

. *** streg

. sort SUBJID dthdyxtdc

. drop if trtgrp==2

(49,968 observations deleted)

. drop if progtdc==0

(10,866 observations deleted)

. * drop anyone who died on same day as progression

. drop if PDDYLR == deathtime

(16 observations deleted)

. by SUBJID: gen obsno = _n

. by SUBJID: gen trtnew = 0

. by SUBJID: replace trtnew = 1 if xo==1 & dthdyxtdc>=xotime

(22802 real changes made)

. by SUBJID: egen minrisk=min(time)

. by SUBJID: replace dthdyxtdc=dthdyxtdc-minrisk

(40930 real changes made)

. by SUBJID: replace xotime=xotime-minrisk

(39325 real changes made)

. by SUBJID: replace time=time-minrisk

(40930 real changes made)

. by SUBJID: replace admin=admin-minrisk

(40930 real changes made)

.

. stset dthdyxtdc, failure(deathtdc) id(SUBJID)

Survival-time data settings

ID variable: SUBJID

Failure event: deathtdc!=0 & deathtdc<.

Observed time interval: (dthdyxtdc[_n-1], dthdyxtdc]

Exit on or before: failure

--------------------------------------------------------------------------

40,967 total observations

0 exclusions

--------------------------------------------------------------------------

40,967 observations remaining, representing

199 subjects

189 failures in single-failure-per-subject data

40,967 total analysis time at risk and under observation

At risk from t = 0

Earliest observed entry t = 0

Last observed exit t = 784

. streg trtnew becogstrat diagtype eq5dbase eq5dmissb regionstrat PDDYLR eq5datprog eq5dind

> eq5dmissingatprog ecogatprog bestrespatprog respmissingatprog LSSLDatprog LSSLDmissingatpr

> og, dist(weibull) time iterate(200)

Failure _d: deathtdc

Analysis time _t: dthdyxtdc

ID variable: SUBJID

Fitting constant-only model:

Iteration 0: log likelihood = -296.89063

Iteration 1: log likelihood = -295.78605

Iteration 2: log likelihood = -295.78531

Iteration 3: log likelihood = -295.78531

Fitting full model:

Iteration 0: log likelihood = -295.78531

Iteration 1: log likelihood = -260.98574

Iteration 2: log likelihood = -244.50908

Iteration 3: log likelihood = -244.37535

Iteration 4: log likelihood = -244.37528

Iteration 5: log likelihood = -244.37528

Weibull AFT regression

No. of subjects = 199 Number of obs = 40,967

No. of failures = 189

Time at risk = 40,967

LR chi2(15) = 102.82

Log likelihood = -244.37528 Prob > chi2 = 0.0000

------------------------------------------------------------------------------------

_t | Coefficient Std. err. z P>|z| [95% conf. interval]

-------------------+----------------------------------------------------------------

trtnew | .4678558 .111635 4.19 0.000 .2490553 .6866563

becogstrat | -.177278 .1938358 -0.91 0.360 -.5571891 .2026331

diagtype | .0201755 .1166955 0.17 0.863 -.2085434 .2488945

eq5dbase | -.8870191 .3262514 -2.72 0.007 -1.52646 -.2475782

eq5dmissb | -.5123451 .4436071 -1.15 0.248 -1.381799 .3571087

regionstrat | .0118113 .077831 0.15 0.879 -.1407347 .1643572

PDDYLR | .0060667 .0024155 2.51 0.012 .0013324 .0108009

eq5datprog | .1700265 .3209956 0.53 0.596 -.4591133 .7991663

eq5dind | .1592496 .524292 0.30 0.761 -.8683438 1.186843

eq5dmissingatprog | .1797486 .2200635 0.82 0.414 -.2515678 .6110651

ecogatprog | -.5793794 .1014151 -5.71 0.000 -.7781494 -.3806094

bestrespatprog | .0703588 .1182159 0.60 0.552 -.1613401 .3020577

respmissingatprog | .0239631 .1597233 0.15 0.881 -.2890888 .3370149

LSSLDatprog | -.0022052 .0005159 -4.27 0.000 -.0032164 -.0011941

LSSLDmissingatprog | -.2209522 .1219961 -1.81 0.070 -.4600602 .0181558

_cons | 5.929556 .7044069 8.42 0.000 4.548944 7.310168

-------------------+----------------------------------------------------------------

/ln_p | .3441976 .0558751 6.16 0.000 .2346844 .4537109

-------------------+----------------------------------------------------------------

p | 1.410857 .0788319 1.26451 1.574143

1/p | .7087888 .0396037 .6352664 .7908204

------------------------------------------------------------------------------------

.

. * Same model as for IPCW denominator. Note, had to take ecogmisslastvisit out as !=0 perfe

> ctly predicted failure.

.

. scalar tsec_af = exp(_b[trtnew])

. di tsec_af

1.5965672

.

. restore

. sort SUBJID

. preserve

.

. ***Analysis on overall survival***

. collapse (max) trtgrp krasi xo regionstrat becogstrat dthdyxtdc xotime deathtdc admin, by(

> SUBJID)

. by SUBJID: replace xotime=0 if xotime==.

(259 real changes made)

. by SUBJID: replace xotime = 0 if krasi==1 & trtgrp==1 & xo==1

(77 real changes made)

. by SUBJID: replace xo = 0 if krasi==1 & trtgrp==1 & xo==1

(77 real changes made)

.

. ***below allows for recensoring***

. gen cfact = dthdyxtdc if trtgrp==2

(219 missing values generated)

. gen dcfact = deathtdc if trtgrp==2

(219 missing values generated)

.

. replace cfact = (xotime + ((dthdyxtdc-xotime)/(tsec_af))) if (trtgrp==1 & xotime>0)

(91 real changes made)

. replace cfact = dthdyxtdc if (trtgrp==1 & xotime==0)

(128 real changes made)

. gen OSadminc = admin/(tsec_af) if (trtgrp==1 & (tsec_af)>1.00)

(208 missing values generated)

. replace dcfact = deathtdc if trtgrp==1

(219 real changes made)

. replace dcfact=0 if (OSadminc<=cfact & trtgrp==1)

(5 real changes made)

. replace cfact = OSadminc if (OSadminc<=cfact & trtgrp==1)

(19 real changes made)

.

. ***do survival analysis on re-estimated survival times***

. stset cfact, failure(dcfact) id(SUBJID)

Survival-time data settings

ID variable: SUBJID

Failure event: dcfact!=0 & dcfact<.

Observed time interval: (cfact[_n-1], cfact]

Exit on or before: failure

--------------------------------------------------------------------------

427 total observations

0 exclusions

--------------------------------------------------------------------------

427 observations remaining, representing

427 subjects

386 failures in single-failure-per-subject data

91,702.162 total analysis time at risk and under observation

At risk from t = 0

Earliest observed entry t = 0

Last observed exit t = 1,024

.

. stcox trtgrp regionstrat becogstrat

Failure _d: dcfact

Analysis time _t: cfact

ID variable: SUBJID

Iteration 0: log likelihood = -1996.5186

Iteration 1: log likelihood = -1984.1595

Iteration 2: log likelihood = -1966.5319

Iteration 3: log likelihood = -1966.1478

Iteration 4: log likelihood = -1966.1473

Refining estimates:

Iteration 0: log likelihood = -1966.1473

Cox regression with Breslow method for ties

No. of subjects = 427 Number of obs = 427

No. of failures = 386

Time at risk = 91,702.1618

LR chi2(3) = 60.74

Log likelihood = -1966.1473 Prob > chi2 = 0.0000

------------------------------------------------------------------------------

_t | Haz. ratio Std. err. z P>|z| [95% conf. interval]

-------------+----------------------------------------------------------------

trtgrp | .7561462 .0784689 -2.69 0.007 .616982 .9266998

regionstrat | .9963084 .0693241 -0.05 0.958 .8692933 1.141882

becogstrat | 3.401792 .5027875 8.28 0.000 2.546241 4.544813

------------------------------------------------------------------------------

.

. restore

.

. ******************************************************************************************

> ***************

. *** TSEsimp 14 (secondary analysis, reduced model, with recens, Gen Gamma) Estimand 1 ***

. ******************************************************************************************

> ***************

.

. preserve

.

. * note, 8 patients switched before investigator observed progression. 7 were kras MT, so p

> rimary analyses will not adjust for these anyway.

. * protocol suggested switching permitted only after progression. So assume these patients

> switched due to some signs of progression.

. * need this, because SNM models will fit much better if applied only to the time-periods w

> here switching was "permitted".

. * so, for these 8 patients, replace PDDYLR to equal xotime

. * and replace progtdc to = 1 after this point for these patients

. sort SUBJID dthdyxtdc

. by SUBJID: replace PDDYLR = xotime if (PDDYLR>xotime & xotime!=.)

(1,252 real changes made)

. by SUBJID: replace progtdc = 1 if (PDDYLR <= dthdyxtdc & PDLR==1)

(984 real changes made)

.

. replace xotdc = 0 if krasi==1 & trtgrp==1

(14,415 real changes made)

. replace xo = . if krasi==1 & trtgrp==1 & xo==1

(18,500 real changes made, 18,500 to missing)

.

. by SUBJID: replace lastobs = 0

(427 real changes made)

. by SUBJID: replace lastobs = 1 if _n==_N

(427 real changes made)

.

. *** streg

. sort SUBJID dthdyxtdc

. drop if trtgrp==2

(49,968 observations deleted)

. drop if progtdc==0

(10,866 observations deleted)

. * drop anyone who died on same day as progression

. drop if PDDYLR == deathtime

(16 observations deleted)

. by SUBJID: gen obsno = _n

. by SUBJID: gen trtnew = 0

. by SUBJID: replace trtnew = 1 if xo==1 & dthdyxtdc>=xotime

(22802 real changes made)

. by SUBJID: egen minrisk=min(time)

. by SUBJID: replace dthdyxtdc=dthdyxtdc-minrisk

(40930 real changes made)

. by SUBJID: replace xotime=xotime-minrisk

(39325 real changes made)

. by SUBJID: replace time=time-minrisk

(40930 real changes made)

. by SUBJID: replace admin=admin-minrisk

(40930 real changes made)

.

. stset dthdyxtdc, failure(deathtdc) id(SUBJID)

Survival-time data settings

ID variable: SUBJID

Failure event: deathtdc!=0 & deathtdc<.

Observed time interval: (dthdyxtdc[_n-1], dthdyxtdc]

Exit on or before: failure

--------------------------------------------------------------------------

40,967 total observations

0 exclusions

--------------------------------------------------------------------------

40,967 observations remaining, representing

199 subjects

189 failures in single-failure-per-subject data

40,967 total analysis time at risk and under observation

At risk from t = 0

Earliest observed entry t = 0

Last observed exit t = 784

. streg trtnew becogstrat diagtype eq5dbase eq5dmissb regionstrat PDDYLR eq5datprog eq5dind

> eq5dmissingatprog ecogatprog bestrespatprog respmissingatprog LSSLDatprog LSSLDmissingatpr

> og, dist(ggamma) time iterate(200)

Failure _d: deathtdc

Analysis time _t: dthdyxtdc

ID variable: SUBJID

Fitting constant-only model:

Iteration 0: log likelihood = -1090.3658 (not concave)

Iteration 1: log likelihood = -428.22643 (not concave)

Iteration 2: log likelihood = -306.47474

Iteration 3: log likelihood = -295.5221

Iteration 4: log likelihood = -294.9627

Iteration 5: log likelihood = -294.95851

Iteration 6: log likelihood = -294.95851

Fitting full model:

Iteration 0: log likelihood = -294.95851

Iteration 1: log likelihood = -258.87447 (not concave)

Iteration 2: log likelihood = -248.74876

Iteration 3: log likelihood = -246.44867

Iteration 4: log likelihood = -243.01672

Iteration 5: log likelihood = -242.94666

Iteration 6: log likelihood = -242.9466

Iteration 7: log likelihood = -242.9466

Generalized gamma AFT regression

No. of subjects = 199 Number of obs = 40,967

No. of failures = 189

Time at risk = 40,967

LR chi2(15) = 104.02

Log likelihood = -242.9466 Prob > chi2 = 0.0000

------------------------------------------------------------------------------------

_t | Coefficient Std. err. z P>|z| [95% conf. interval]

-------------------+----------------------------------------------------------------

trtnew | .4916452 .1169846 4.20 0.000 .2623596 .7209309

becogstrat | -.1915053 .2033122 -0.94 0.346 -.5899898 .2069792

diagtype | .0690384 .1247711 0.55 0.580 -.1755085 .3135854

eq5dbase | -.7942125 .3325157 -2.39 0.017 -1.445931 -.1424937

eq5dmissb | -.4676044 .4481365 -1.04 0.297 -1.345936 .4107269

regionstrat | .0219666 .081971 0.27 0.789 -.1386937 .1826269

PDDYLR | .0063867 .0024881 2.57 0.010 .00151 .0112634

eq5datprog | .1525926 .3250952 0.47 0.639 -.4845823 .7897674

eq5dind | .1424181 .535228 0.27 0.790 -.9066095 1.191446

eq5dmissingatprog | .1892362 .2200921 0.86 0.390 -.2421363 .6206088

ecogatprog | -.5761579 .1035153 -5.57 0.000 -.7790442 -.3732716

bestrespatprog | .0667369 .1223313 0.55 0.585 -.1730281 .3065018

respmissingatprog | -.0156826 .17116 -0.09 0.927 -.3511502 .3197849

LSSLDatprog | -.0023818 .00054 -4.41 0.000 -.0034402 -.0013235

LSSLDmissingatprog | -.2207066 .1260407 -1.75 0.080 -.4677418 .0263287

_cons | 5.732734 .7273685 7.88 0.000 4.307118 7.15835

-------------------+----------------------------------------------------------------

/lnsigma | -.271031 .0664514 -4.08 0.000 -.4012733 -.1407886

/kappa | .63543 .2067478 3.07 0.002 .2302116 1.040648

-------------------+----------------------------------------------------------------

sigma | .7625929 .0506754 .6694671 .8686729

------------------------------------------------------------------------------------

.

. * Same model as for IPCW denominator. Note, had to take ecogmisslastvisit out as !=0 perfe

> ctly predicted failure.

.

. scalar tsec_af = exp(_b[trtnew])

. di tsec_af

1.635004

.

. restore

. sort SUBJID

. preserve

.

. ***Analysis on overall survival***

. collapse (max) trtgrp krasi xo regionstrat becogstrat dthdyxtdc xotime deathtdc admin, by(

> SUBJID)

. by SUBJID: replace xotime=0 if xotime==.

(259 real changes made)

. by SUBJID: replace xotime = 0 if krasi==1 & trtgrp==1 & xo==1

(77 real changes made)

. by SUBJID: replace xo = 0 if krasi==1 & trtgrp==1 & xo==1

(77 real changes made)

.

. ***below allows for recensoring***

. gen cfact = dthdyxtdc if trtgrp==2

(219 missing values generated)

. gen dcfact = deathtdc if trtgrp==2

(219 missing values generated)

.

. replace cfact = (xotime + ((dthdyxtdc-xotime)/(tsec_af))) if (trtgrp==1 & xotime>0)

(91 real changes made)

. replace cfact = dthdyxtdc if (trtgrp==1 & xotime==0)

(128 real changes made)

. gen OSadminc = admin/(tsec_af) if (trtgrp==1 & (tsec_af)>1.00)

(208 missing values generated)

. replace dcfact = deathtdc if trtgrp==1

(219 real changes made)

. replace dcfact=0 if (OSadminc<=cfact & trtgrp==1)

(5 real changes made)

. replace cfact = OSadminc if (OSadminc<=cfact & trtgrp==1)

(19 real changes made)

.

. ***do survival analysis on re-estimated survival times***

. stset cfact, failure(dcfact) id(SUBJID)

Survival-time data settings

ID variable: SUBJID

Failure event: dcfact!=0 & dcfact<.

Observed time interval: (cfact[_n-1], cfact]

Exit on or before: failure

--------------------------------------------------------------------------

427 total observations

0 exclusions

--------------------------------------------------------------------------

427 observations remaining, representing

427 subjects

386 failures in single-failure-per-subject data

91,255.258 total analysis time at risk and under observation

At risk from t = 0

Earliest observed entry t = 0

Last observed exit t = 1,024

.

. stcox trtgrp regionstrat becogstrat

Failure _d: dcfact

Analysis time _t: cfact

ID variable: SUBJID

Iteration 0: log likelihood = -1995.9311

Iteration 1: log likelihood = -1983.1382

Iteration 2: log likelihood = -1965.5683

Iteration 3: log likelihood = -1965.1859

Iteration 4: log likelihood = -1965.1854

Refining estimates:

Iteration 0: log likelihood = -1965.1854

Cox regression with Breslow method for ties

No. of subjects = 427 Number of obs = 427

No. of failures = 386

Time at risk = 91,255.2576

LR chi2(3) = 61.49

Log likelihood = -1965.1854 Prob > chi2 = 0.0000

------------------------------------------------------------------------------

_t | Haz. ratio Std. err. z P>|z| [95% conf. interval]

-------------+----------------------------------------------------------------

trtgrp | .7444725 .0773967 -2.84 0.005 .6072342 .9127274

regionstrat | .9947208 .0692075 -0.08 0.939 .8679185 1.140049

becogstrat | 3.399784 .5024923 8.28 0.000 2.544735 4.542134

------------------------------------------------------------------------------

.

. restore

.

. ******************************************************************************************

> ***************

. *** TSEsimp 15 (secondary analysis, reduced model, no recens, Weibull) Estimand 1 [231 fro

> m TSEsimp file] ***

. ******************************************************************************************

> ***************

.

. preserve

.

. * note, 8 patients switched before investigator observed progression. 7 were kras MT, so p

> rimary analyses will not adjust for these anyway.

. * protocol suggested switching permitted only after progression. So assume these patients

> switched due to some signs of progression.

. * need this, because SNM models will fit much better if applied only to the time-periods w

> here switching was "permitted".

. * so, for these 8 patients, replace PDDYLR to equal xotime

. * and replace progtdc to = 1 after this point for these patients

. sort SUBJID dthdyxtdc

. by SUBJID: replace PDDYLR = xotime if (PDDYLR>xotime & xotime!=.)

(1,252 real changes made)

. by SUBJID: replace progtdc = 1 if (PDDYLR <= dthdyxtdc & PDLR==1)

(984 real changes made)

.

. replace xotdc = 0 if krasi==1 & trtgrp==1

(14,415 real changes made)

. replace xo = . if krasi==1 & trtgrp==1 & xo==1

(18,500 real changes made, 18,500 to missing)

.

. by SUBJID: replace lastobs = 0

(427 real changes made)

. by SUBJID: replace lastobs = 1 if _n==_N

(427 real changes made)

.

. *** streg

. sort SUBJID dthdyxtdc

. drop if trtgrp==2

(49,968 observations deleted)

. drop if progtdc==0

(10,866 observations deleted)

. * drop anyone who died on same day as progression

. drop if PDDYLR == deathtime

(16 observations deleted)

. by SUBJID: gen obsno = _n

. by SUBJID: gen trtnew = 0

. by SUBJID: replace trtnew = 1 if xo==1 & dthdyxtdc>=xotime

(22802 real changes made)

. by SUBJID: egen minrisk=min(time)

. by SUBJID: replace dthdyxtdc=dthdyxtdc-minrisk

(40930 real changes made)

. by SUBJID: replace xotime=xotime-minrisk

(39325 real changes made)

. by SUBJID: replace time=time-minrisk

(40930 real changes made)

. by SUBJID: replace admin=admin-minrisk

(40930 real changes made)

.

. stset dthdyxtdc, failure(deathtdc) id(SUBJID)

Survival-time data settings

ID variable: SUBJID

Failure event: deathtdc!=0 & deathtdc<.

Observed time interval: (dthdyxtdc[_n-1], dthdyxtdc]

Exit on or before: failure

--------------------------------------------------------------------------

40,967 total observations

0 exclusions

--------------------------------------------------------------------------

40,967 observations remaining, representing

199 subjects

189 failures in single-failure-per-subject data

40,967 total analysis time at risk and under observation

At risk from t = 0

Earliest observed entry t = 0

Last observed exit t = 784

. streg trtnew becogstrat diagtype eq5dbase eq5dmissb regionstrat PDDYLR eq5datprog eq5dind

> eq5dmissingatprog ecogatprog bestrespatprog respmissingatprog LSSLDatprog LSSLDmissingatpr

> og, dist(weibull) time iterate(200)

Failure _d: deathtdc

Analysis time _t: dthdyxtdc

ID variable: SUBJID

Fitting constant-only model:

Iteration 0: log likelihood = -296.89063

Iteration 1: log likelihood = -295.78605

Iteration 2: log likelihood = -295.78531

Iteration 3: log likelihood = -295.78531

Fitting full model:

Iteration 0: log likelihood = -295.78531

Iteration 1: log likelihood = -260.98574

Iteration 2: log likelihood = -244.50908

Iteration 3: log likelihood = -244.37535

Iteration 4: log likelihood = -244.37528

Iteration 5: log likelihood = -244.37528

Weibull AFT regression

No. of subjects = 199 Number of obs = 40,967

No. of failures = 189

Time at risk = 40,967

LR chi2(15) = 102.82

Log likelihood = -244.37528 Prob > chi2 = 0.0000

------------------------------------------------------------------------------------

_t | Coefficient Std. err. z P>|z| [95% conf. interval]

-------------------+----------------------------------------------------------------

trtnew | .4678558 .111635 4.19 0.000 .2490553 .6866563

becogstrat | -.177278 .1938358 -0.91 0.360 -.5571891 .2026331

diagtype | .0201755 .1166955 0.17 0.863 -.2085434 .2488945

eq5dbase | -.8870191 .3262514 -2.72 0.007 -1.52646 -.2475782

eq5dmissb | -.5123451 .4436071 -1.15 0.248 -1.381799 .3571087

regionstrat | .0118113 .077831 0.15 0.879 -.1407347 .1643572

PDDYLR | .0060667 .0024155 2.51 0.012 .0013324 .0108009

eq5datprog | .1700265 .3209956 0.53 0.596 -.4591133 .7991663

eq5dind | .1592496 .524292 0.30 0.761 -.8683438 1.186843

eq5dmissingatprog | .1797486 .2200635 0.82 0.414 -.2515678 .6110651

ecogatprog | -.5793794 .1014151 -5.71 0.000 -.7781494 -.3806094

bestrespatprog | .0703588 .1182159 0.60 0.552 -.1613401 .3020577

respmissingatprog | .0239631 .1597233 0.15 0.881 -.2890888 .3370149

LSSLDatprog | -.0022052 .0005159 -4.27 0.000 -.0032164 -.0011941

LSSLDmissingatprog | -.2209522 .1219961 -1.81 0.070 -.4600602 .0181558

_cons | 5.929556 .7044069 8.42 0.000 4.548944 7.310168

-------------------+----------------------------------------------------------------

/ln_p | .3441976 .0558751 6.16 0.000 .2346844 .4537109

-------------------+----------------------------------------------------------------

p | 1.410857 .0788319 1.26451 1.574143

1/p | .7087888 .0396037 .6352664 .7908204

------------------------------------------------------------------------------------

.

. * Same model as for IPCW denominator except TDC variables. Note, had to take ecogmisslastv

> isit out as !=0 perfectly predicted failure

.

. scalar tsec_af = exp(_b[trtnew])

. di tsec_af

1.5965672

.

. restore

. sort SUBJID

. preserve

.

. ***Analysis on overall survival***

. collapse (max) trtgrp krasi xo regionstrat becogstrat dthdyxtdc xotime deathtdc admin, by(

> SUBJID)

. by SUBJID: replace xotime=0 if xotime==.

(259 real changes made)

. by SUBJID: replace xotime = 0 if krasi==1 & trtgrp==1 & xo==1

(77 real changes made)

. by SUBJID: replace xo = 0 if krasi==1 & trtgrp==1 & xo==1

(77 real changes made)

.

. ***below no recensoring***

. gen cfact = dthdyxtdc if trtgrp==2

(219 missing values generated)

. gen dcfact = deathtdc if trtgrp==2

(219 missing values generated)

.

. replace cfact = (xotime + ((dthdyxtdc-xotime)/(tsec_af))) if (trtgrp==1 & xotime>0)

(91 real changes made)

. replace cfact = dthdyxtdc if (trtgrp==1 & xotime==0)

(128 real changes made)

. replace dcfact = deathtdc if trtgrp==1

(219 real changes made)

.

. ***do survival analysis on re-estimated survival times***

. stset cfact, failure(dcfact) id(SUBJID)

Survival-time data settings

ID variable: SUBJID

Failure event: dcfact!=0 & dcfact<.

Observed time interval: (cfact[_n-1], cfact]

Exit on or before: failure

--------------------------------------------------------------------------

427 total observations

0 exclusions

--------------------------------------------------------------------------

427 observations remaining, representing

427 subjects

391 failures in single-failure-per-subject data

93,330.895 total analysis time at risk and under observation

At risk from t = 0

Earliest observed entry t = 0

Last observed exit t = 1,024

.

. stcox trtgrp regionstrat becogstrat

Failure _d: dcfact

Analysis time _t: cfact

ID variable: SUBJID

Iteration 0: log likelihood = -2020.2098

Iteration 1: log likelihood = -2008.4495

Iteration 2: log likelihood = -1990.7414

Iteration 3: log likelihood = -1990.3559

Iteration 4: log likelihood = -1990.3554

Refining estimates:

Iteration 0: log likelihood = -1990.3554

Cox regression with Breslow method for ties

No. of subjects = 427 Number of obs = 427

No. of failures = 391

Time at risk = 93,330.8949

LR chi2(3) = 59.71

Log likelihood = -1990.3554 Prob > chi2 = 0.0000

------------------------------------------------------------------------------

_t | Haz. ratio Std. err. z P>|z| [95% conf. interval]

-------------+----------------------------------------------------------------

trtgrp | .7769288 .079201 -2.48 0.013 .6362222 .9487541

regionstrat | .9962769 .0688193 -0.05 0.957 .8701259 1.140717

becogstrat | 3.40817 .5037877 8.30 0.000 2.55093 4.553484

------------------------------------------------------------------------------

.

. restore

.

. ******************************************************************************************

> ***************

. *** TSEsimp 16 (secondary analysis, reduced model, no recens, Gen Gamma) Estimand 1 ***

. ******************************************************************************************

> ***************

.

. preserve

.

. * note, 8 patients switched before investigator observed progression. 7 were kras MT, so p

> rimary analyses will not adjust for these anyway.

. * protocol suggested switching permitted only after progression. So assume these patients

> switched due to some signs of progression.

. * need this, because SNM models will fit much better if applied only to the time-periods w

> here switching was "permitted".

. * so, for these 8 patients, replace PDDYLR to equal xotime

. * and replace progtdc to = 1 after this point for these patients

. sort SUBJID dthdyxtdc

. by SUBJID: replace PDDYLR = xotime if (PDDYLR>xotime & xotime!=.)

(1,252 real changes made)

. by SUBJID: replace progtdc = 1 if (PDDYLR <= dthdyxtdc & PDLR==1)

(984 real changes made)

.

. replace xotdc = 0 if krasi==1 & trtgrp==1

(14,415 real changes made)

. replace xo = . if krasi==1 & trtgrp==1 & xo==1

(18,500 real changes made, 18,500 to missing)

.

. by SUBJID: replace lastobs = 0

(427 real changes made)

. by SUBJID: replace lastobs = 1 if _n==_N

(427 real changes made)

.

. *** streg

. sort SUBJID dthdyxtdc

. drop if trtgrp==2

(49,968 observations deleted)

. drop if progtdc==0

(10,866 observations deleted)

. * drop anyone who died on same day as progression

. drop if PDDYLR == deathtime

(16 observations deleted)

. by SUBJID: gen obsno = _n

. by SUBJID: gen trtnew = 0

. by SUBJID: replace trtnew = 1 if xo==1 & dthdyxtdc>=xotime

(22802 real changes made)

. by SUBJID: egen minrisk=min(time)

. by SUBJID: replace dthdyxtdc=dthdyxtdc-minrisk

(40930 real changes made)

. by SUBJID: replace xotime=xotime-minrisk

(39325 real changes made)

. by SUBJID: replace time=time-minrisk

(40930 real changes made)

. by SUBJID: replace admin=admin-minrisk

(40930 real changes made)

.

. stset dthdyxtdc, failure(deathtdc) id(SUBJID)

Survival-time data settings

ID variable: SUBJID

Failure event: deathtdc!=0 & deathtdc<.

Observed time interval: (dthdyxtdc[_n-1], dthdyxtdc]

Exit on or before: failure

--------------------------------------------------------------------------

40,967 total observations

0 exclusions

--------------------------------------------------------------------------

40,967 observations remaining, representing

199 subjects

189 failures in single-failure-per-subject data

40,967 total analysis time at risk and under observation

At risk from t = 0

Earliest observed entry t = 0

Last observed exit t = 784

. streg trtnew becogstrat diagtype eq5dbase eq5dmissb regionstrat PDDYLR eq5datprog eq5dind

> eq5dmissingatprog ecogatprog bestrespatprog respmissingatprog LSSLDatprog LSSLDmissingatpr

> og, dist(ggamma) time iterate(200)

Failure _d: deathtdc

Analysis time _t: dthdyxtdc

ID variable: SUBJID

Fitting constant-only model:

Iteration 0: log likelihood = -1090.3658 (not concave)

Iteration 1: log likelihood = -428.22643 (not concave)

Iteration 2: log likelihood = -306.47474

Iteration 3: log likelihood = -295.5221

Iteration 4: log likelihood = -294.9627

Iteration 5: log likelihood = -294.95851

Iteration 6: log likelihood = -294.95851

Fitting full model:

Iteration 0: log likelihood = -294.95851

Iteration 1: log likelihood = -258.87447 (not concave)

Iteration 2: log likelihood = -248.74876

Iteration 3: log likelihood = -246.44867

Iteration 4: log likelihood = -243.01672

Iteration 5: log likelihood = -242.94666

Iteration 6: log likelihood = -242.9466

Iteration 7: log likelihood = -242.9466

Generalized gamma AFT regression

No. of subjects = 199 Number of obs = 40,967

No. of failures = 189

Time at risk = 40,967

LR chi2(15) = 104.02

Log likelihood = -242.9466 Prob > chi2 = 0.0000

------------------------------------------------------------------------------------

_t | Coefficient Std. err. z P>|z| [95% conf. interval]

-------------------+----------------------------------------------------------------

trtnew | .4916452 .1169846 4.20 0.000 .2623596 .7209309

becogstrat | -.1915053 .2033122 -0.94 0.346 -.5899898 .2069792

diagtype | .0690384 .1247711 0.55 0.580 -.1755085 .3135854

eq5dbase | -.7942125 .3325157 -2.39 0.017 -1.445931 -.1424937

eq5dmissb | -.4676044 .4481365 -1.04 0.297 -1.345936 .4107269

regionstrat | .0219666 .081971 0.27 0.789 -.1386937 .1826269

PDDYLR | .0063867 .0024881 2.57 0.010 .00151 .0112634

eq5datprog | .1525926 .3250952 0.47 0.639 -.4845823 .7897674

eq5dind | .1424181 .535228 0.27 0.790 -.9066095 1.191446

eq5dmissingatprog | .1892362 .2200921 0.86 0.390 -.2421363 .6206088

ecogatprog | -.5761579 .1035153 -5.57 0.000 -.7790442 -.3732716

bestrespatprog | .0667369 .1223313 0.55 0.585 -.1730281 .3065018

respmissingatprog | -.0156826 .17116 -0.09 0.927 -.3511502 .3197849

LSSLDatprog | -.0023818 .00054 -4.41 0.000 -.0034402 -.0013235

LSSLDmissingatprog | -.2207066 .1260407 -1.75 0.080 -.4677418 .0263287

_cons | 5.732734 .7273685 7.88 0.000 4.307118 7.15835

-------------------+----------------------------------------------------------------

/lnsigma | -.271031 .0664514 -4.08 0.000 -.4012733 -.1407886

/kappa | .63543 .2067478 3.07 0.002 .2302116 1.040648

-------------------+----------------------------------------------------------------

sigma | .7625929 .0506754 .6694671 .8686729

------------------------------------------------------------------------------------

.

. * Same model as for IPCW denominator except TDC variables. Note, had to take ecogmisslastv

> isit out as !=0 perfectly predicted failure

.

. scalar tsec_af = exp(_b[trtnew])

. di tsec_af

1.635004

.

. restore

. sort SUBJID

. preserve

.

. ***Analysis on overall survival***

. collapse (max) trtgrp krasi xo regionstrat becogstrat dthdyxtdc xotime deathtdc admin, by(

> SUBJID)

. by SUBJID: replace xotime=0 if xotime==.

(259 real changes made)

. by SUBJID: replace xotime = 0 if krasi==1 & trtgrp==1 & xo==1

(77 real changes made)

. by SUBJID: replace xo = 0 if krasi==1 & trtgrp==1 & xo==1

(77 real changes made)

.

. ***below no recensoring***

. gen cfact = dthdyxtdc if trtgrp==2

(219 missing values generated)

. gen dcfact = deathtdc if trtgrp==2

(219 missing values generated)

.

. replace cfact = (xotime + ((dthdyxtdc-xotime)/(tsec_af))) if (trtgrp==1 & xotime>0)

(91 real changes made)

. replace cfact = dthdyxtdc if (trtgrp==1 & xotime==0)

(128 real changes made)

. replace dcfact = deathtdc if trtgrp==1

(219 real changes made)

.

. ***do survival analysis on re-estimated survival times***

. stset cfact, failure(dcfact) id(SUBJID)

Survival-time data settings

ID variable: SUBJID

Failure event: dcfact!=0 & dcfact<.

Observed time interval: (cfact[_n-1], cfact]

Exit on or before: failure

--------------------------------------------------------------------------

427 total observations

0 exclusions

--------------------------------------------------------------------------

427 observations remaining, representing

427 subjects

391 failures in single-failure-per-subject data

92,996.486 total analysis time at risk and under observation

At risk from t = 0

Earliest observed entry t = 0

Last observed exit t = 1,024

.

. stcox trtgrp regionstrat becogstrat

Failure _d: dcfact

Analysis time _t: cfact

ID variable: SUBJID

Iteration 0: log likelihood = -2020.124

Iteration 1: log likelihood = -2008.09

Iteration 2: log likelihood = -1990.4386

Iteration 3: log likelihood = -1990.0549

Iteration 4: log likelihood = -1990.0545

Refining estimates:

Iteration 0: log likelihood = -1990.0545

Cox regression with Breslow method for ties

No. of subjects = 427 Number of obs = 427

No. of failures = 391

Time at risk = 92,996.4861

LR chi2(3) = 60.14

Log likelihood = -1990.0545 Prob > chi2 = 0.0000

------------------------------------------------------------------------------

_t | Haz. ratio Std. err. z P>|z| [95% conf. interval]

-------------+----------------------------------------------------------------

trtgrp | .7693341 .0784439 -2.57 0.010 .6299754 .9395207

regionstrat | .9954241 .0687688 -0.07 0.947 .8693666 1.13976

becogstrat | 3.406379 .5035262 8.29 0.000 2.549585 4.5511

------------------------------------------------------------------------------

.

. restore

.

.

end of do-file

. do "X:\ScHARR\Users\cm1nrl\Case studies\Amgen\Code\Final methods do files for paper\All TS

> E analyses for paper estimand 2.do"

. *** TSEsimp PANI ANALYSES FOR PAPER: ESTIMAND 2 ***

.

.

. ******************************************************************************************

> ***************

. *** TSEsimp 1 (primary analysis, full model, with recens, Weibull) Estimand 2 [249 from TS

> Esimp file] ***

. ******************************************************************************************

> ***************

. use "X:\ScHARR\Users\cm1nrl\Case studies\Amgen\Data\Interim merged datasets\tdc_dataset34.

> dta", clear

(TREAT)

.

. preserve

.

. * note, 8 patients switched before investigator observed progression. 7 were kras MT, so p

> rimary analyses will not adjust for these anyway.

. * protocol suggested switching permitted only after progression. So assume these patients

> switched due to some signs of progression.

. * need this, because SNM models will fit much better if applied only to the time-periods w

> here switching was "permitted".

. * so, for these 8 patients, replace PDDYLR to equal xotime

. * and replace progtdc to = 1 after this point for these patients

. sort SUBJID dthdyxtdc

. by SUBJID: replace PDDYLR = xotime if (PDDYLR>xotime & xotime!=.)

(1,252 real changes made)

. by SUBJID: replace progtdc = 1 if (PDDYLR <= dthdyxtdc & PDLR==1)

(984 real changes made)

.

. by SUBJID: replace lastobs = 0

(427 real changes made)

. by SUBJID: replace lastobs = 1 if _n==_N

(427 real changes made)

.

. *** Streg

. sort SUBJID dthdyxtdc

. drop if trtgrp==2

(49,968 observations deleted)

. drop if progtdc==0

(10,866 observations deleted)

. * drop anyone who died on same day as progression

. drop if PDDYLR == deathtime

(16 observations deleted)

. * for primary analysis, get treatment effect comparing WT switchers to WT non-switchers, i

> .e. exclude MT

. drop if krasi==1

(16,044 observations deleted)

. by SUBJID: gen obsno = _n

. by SUBJID: gen trtnew = 0

. by SUBJID: replace trtnew = 1 if xo==1 & dthdyxtdc>=xotime

(22802 real changes made)

. by SUBJID: egen minrisk=min(time)

. by SUBJID: replace dthdyxtdc=dthdyxtdc-minrisk

(24923 real changes made)

. by SUBJID: replace xotime=xotime-minrisk

(24117 real changes made)

. by SUBJID: replace time=time-minrisk

(24923 real changes made)

. by SUBJID: replace admin=admin-minrisk

(24923 real changes made)

.

. stset dthdyxtdc, failure(deathtdc) id(SUBJID)

Survival-time data settings

ID variable: SUBJID

Failure event: deathtdc!=0 & deathtdc<.

Observed time interval: (dthdyxtdc[_n-1], dthdyxtdc]

Exit on or before: failure

--------------------------------------------------------------------------

24,923 total observations

0 exclusions

--------------------------------------------------------------------------

24,923 observations remaining, representing

106 subjects

100 failures in single-failure-per-subject data

24,923 total analysis time at risk and under observation

At risk from t = 0

Earliest observed entry t = 0

Last observed exit t = 784

. streg trtnew AGE becogstrat diagtype BILIULN ASTULN CREATULN ALBULN LDHULN eq5dbase CEAULN

> eq5dmissb regionstrat PDDYLR eq5datprog eq5dind eq5dmissingatprog ecogatprog bestrespatpr

> og respmissingatprog LSSLDatprog LSSLDmissingatprog AATULNatprog AATmissingatprog ALBULNat

> prog ALBmissingatprog ALKULNatprog ALKmissingatprog ASTULNatprog ASTmissingatprog CEAULNat

> prog CEAmissingatprog CREATULNatprog CREATmissingatprog LDHULNatprog LDHmissingatprog BILI

> ULNatprog BILImissingatprog saeatprog, dist(weibull) time iterate(200)

Failure _d: deathtdc

Analysis time _t: dthdyxtdc

ID variable: SUBJID

note: ALBmissingatprog omitted because of collinearity.

note: ALKmissingatprog omitted because of collinearity.

note: ASTmissingatprog omitted because of collinearity.

note: CREATULNatprog omitted because of collinearity.

note: CREATmissingatprog omitted because of collinearity.

note: LDHmissingatprog omitted because of collinearity.

note: BILImissingatprog omitted because of collinearity.

Fitting constant-only model:

Iteration 0: log likelihood = -155.00396

Iteration 1: log likelihood = -153.4072

Iteration 2: log likelihood = -153.40372

Iteration 3: log likelihood = -153.40372

Fitting full model:

Iteration 0: log likelihood = -153.40372

Iteration 1: log likelihood = -121.70482

Iteration 2: log likelihood = -109.88398

Iteration 3: log likelihood = -108.2494

Iteration 4: log likelihood = -108.22721

Iteration 5: log likelihood = -108.22719

Iteration 6: log likelihood = -108.22719

Weibull AFT regression

No. of subjects = 106 Number of obs = 24,923

No. of failures = 100

Time at risk = 24,923

LR chi2(32) = 90.35

Log likelihood = -108.22719 Prob > chi2 = 0.0000

------------------------------------------------------------------------------------

_t | Coefficient Std. err. z P>|z| [95% conf. interval]

-------------------+----------------------------------------------------------------

trtnew | .7925562 .216738 3.66 0.000 .3677574 1.217355

AGE | -.0048295 .0070864 -0.68 0.496 -.0187185 .0090596

becogstrat | .5230229 .2813373 1.86 0.063 -.028388 1.074434

diagtype | .2206367 .1607451 1.37 0.170 -.094418 .5356914

BILIULN | .0355378 .2173681 0.16 0.870 -.3904958 .4615715

ASTULN | .573513 .2583067 2.22 0.026 .0672413 1.079785

CREATULN | -1.204197 .4134357 -2.91 0.004 -2.014516 -.3938775

ALBULN | -.4468884 .3860208 -1.16 0.247 -1.203475 .3096984

LDHULN | .3112406 .2954532 1.05 0.292 -.267837 .8903183

eq5dbase | -.8261484 .3899927 -2.12 0.034 -1.59052 -.0617767

CEAULN | -.2930274 .5818143 -0.50 0.615 -1.433363 .8473077

eq5dmissb | -.2718747 .4762672 -0.57 0.568 -1.205341 .6615919

regionstrat | .0517618 .1067807 0.48 0.628 -.1575245 .2610481

PDDYLR | .0138439 .0040986 3.38 0.001 .0058107 .0218771

eq5datprog | .0695035 .3785514 0.18 0.854 -.6724436 .8114505

eq5dind | .1851247 .6082364 0.30 0.761 -1.006997 1.377246

eq5dmissingatprog | -.0166687 .3018315 -0.06 0.956 -.6082476 .5749102

ecogatprog | -.4862551 .1328997 -3.66 0.000 -.7467337 -.2257765

bestrespatprog | .2345417 .1538745 1.52 0.127 -.0670468 .5361302

respmissingatprog | -.5821665 .2255813 -2.58 0.010 -1.024298 -.1400353

LSSLDatprog | -.0019712 .0007874 -2.50 0.012 -.0035146 -.0004278

LSSLDmissingatprog | -.1649233 .1740925 -0.95 0.343 -.5061384 .1762917

AATULNatprog | -.0258887 .1727636 -0.15 0.881 -.3644992 .3127218

AATmissingatprog | -.4600322 .2435557 -1.89 0.059 -.9373926 .0173282

ALBULNatprog | -.1491619 .3829531 -0.39 0.697 -.8997361 .6014123

ALBmissingatprog | 0 (omitted)

ALKULNatprog | -.1218627 .2242434 -0.54 0.587 -.5613717 .3176463

ALKmissingatprog | 0 (omitted)

ASTULNatprog | -.7328966 .258833 -2.83 0.005 -1.2402 -.2255933

ASTmissingatprog | 0 (omitted)

CEAULNatprog | .3808302 .5993283 0.64 0.525 -.7938317 1.555492

CEAmissingatprog | -.3042879 .2144844 -1.42 0.156 -.7246697 .1160938

CREATULNatprog | 0 (omitted)

CREATmissingatprog | 0 (omitted)

LDHULNatprog | .238702 .3197934 0.75 0.455 -.3880815 .8654855

LDHmissingatprog | 0 (omitted)

BILIULNatprog | -.595671 .2202507 -2.70 0.007 -1.027354 -.1639875

BILImissingatprog | 0 (omitted)

saeatprog | 1.303075 .6652494 1.96 0.050 -.0007896 2.60694

_cons | 5.040682 1.000408 5.04 0.000 3.079918 7.001445

-------------------+----------------------------------------------------------------

/ln_p | .6606433 .0842989 7.84 0.000 .4954204 .8258662

-------------------+----------------------------------------------------------------

p | 1.936037 .1632059 1.641188 2.283858

1/p | .5165189 .043542 .4378556 .6093147

------------------------------------------------------------------------------------

. * Same model as for IPCW denominator but without the TDC variables. Note, had to take ecog

> misslastvisit out as !=0 perfectly predicted failure.

. * And, ALBmisslastvisit, ALKmisslastvisit, ASTmisslastvisit, CREATmisslastvisit, LDHmissla

> stvisit, BILImisslastvisit all omitted because of collinearity

.

. scalar tsec_af = exp(_b[trtnew])

. di tsec_af

2.2090359

.

. restore

. sort SUBJID

. preserve

.

. ***Analysis on overall survival***

. collapse (max) trtgrp krasi xo regionstrat becogstrat dthdyxtdc xotime deathtdc admin, by(

> SUBJID)

. by SUBJID: replace xotime=0 if xotime==.

(259 real changes made)

. drop if krasi==1

(184 observations deleted)

. ***below allows for recensoring***

. gen cfact = dthdyxtdc if trtgrp==2

(119 missing values generated)

. gen dcfact = deathtdc if trtgrp==2

(119 missing values generated)

.

. replace cfact = (xotime + ((dthdyxtdc-xotime)/(tsec_af))) if (trtgrp==1 & xotime>0)

(91 real changes made)

. replace cfact = dthdyxtdc if (trtgrp==1 & xotime==0)

(28 real changes made)

. gen OSadminc = admin/(tsec_af) if (trtgrp==1 & (tsec_af)>1.00)

(124 missing values generated)

. replace dcfact = deathtdc if trtgrp==1

(119 real changes made)

. replace dcfact=0 if (OSadminc<=cfact & trtgrp==1)

(0 real changes made)

. replace cfact = OSadminc if (OSadminc<=cfact & trtgrp==1)

(9 real changes made)

.

. ***do survival analysis on re-estimated survival times***

. stset cfact, failure(dcfact) id(SUBJID)

Survival-time data settings

ID variable: SUBJID

Failure event: dcfact!=0 & dcfact<.

Observed time interval: (cfact[_n-1], cfact]

Exit on or before: failure

--------------------------------------------------------------------------

243 total observations

0 exclusions

--------------------------------------------------------------------------

243 observations remaining, representing

243 subjects

217 failures in single-failure-per-subject data

51,821.977 total analysis time at risk and under observation

At risk from t = 0

Earliest observed entry t = 0

Last observed exit t = 1,024

. stcox trtgrp regionstrat becogstrat

Failure _d: dcfact

Analysis time _t: cfact

ID variable: SUBJID

Iteration 0: log likelihood = -998.06939

Iteration 1: log likelihood = -978.34473

Iteration 2: log likelihood = -968.0538

Iteration 3: log likelihood = -967.79785

Iteration 4: log likelihood = -967.79743

Refining estimates:

Iteration 0: log likelihood = -967.79743

Cox regression with Breslow method for ties

No. of subjects = 243 Number of obs = 243

No. of failures = 217

Time at risk = 51,821.977

LR chi2(3) = 60.54

Log likelihood = -967.79743 Prob > chi2 = 0.0000

------------------------------------------------------------------------------

_t | Haz. ratio Std. err. z P>|z| [95% conf. interval]

-------------+----------------------------------------------------------------

trtgrp | .4358616 .0649464 -5.57 0.000 .325472 .5836919

regionstrat | .930736 .0857706 -0.78 0.436 .7769361 1.114982

becogstrat | 3.397421 .6976283 5.96 0.000 2.271764 5.08084

------------------------------------------------------------------------------

.

. restore

.

. ******************************************************************************************

> ***************

. *** TSEsimp 2 (primary analysis, full model, with recens, lognormal) Estimand 2 ***

. ******************************************************************************************

> ***************

.

. preserve

.

. * note, 8 patients switched before investigator observed progression. 7 were kras MT, so p

> rimary analyses will not adjust for these anyway.

. * protocol suggested switching permitted only after progression. So assume these patients

> switched due to some signs of progression.

. * need this, because SNM models will fit much better if applied only to the time-periods w

> here switching was "permitted".

. * so, for these 8 patients, replace PDDYLR to equal xotime

. * and replace progtdc to = 1 after this point for these patients

. sort SUBJID dthdyxtdc

. by SUBJID: replace PDDYLR = xotime if (PDDYLR>xotime & xotime!=.)

(1,252 real changes made)

. by SUBJID: replace progtdc = 1 if (PDDYLR <= dthdyxtdc & PDLR==1)

(984 real changes made)

.

. replace xotdc = 0 if krasi==1 & trtgrp==1

(14,415 real changes made)

. replace xo = . if krasi==1 & trtgrp==1 & xo==1

(18,500 real changes made, 18,500 to missing)

.

. by SUBJID: replace lastobs = 0

(427 real changes made)

. by SUBJID: replace lastobs = 1 if _n==_N

(427 real changes made)

.

. *** Streg

. sort SUBJID dthdyxtdc

. drop if trtgrp==2

(49,968 observations deleted)

. drop if progtdc==0

(10,866 observations deleted)

. * drop anyone who died on same day as progression

. drop if PDDYLR == deathtime

(16 observations deleted)

. * for primary analysis, get treatment effect comparing WT switchers to WT non-switchers, i

> .e. exclude MT

. drop if krasi==1

(16,044 observations deleted)

. by SUBJID: gen obsno = _n

. by SUBJID: gen trtnew = 0

. by SUBJID: replace trtnew = 1 if xo==1 & dthdyxtdc>=xotime

(22802 real changes made)

. by SUBJID: egen minrisk=min(time)

. by SUBJID: replace dthdyxtdc=dthdyxtdc-minrisk

(24923 real changes made)

. by SUBJID: replace xotime=xotime-minrisk

(24117 real changes made)

. by SUBJID: replace time=time-minrisk

(24923 real changes made)

. by SUBJID: replace admin=admin-minrisk

(24923 real changes made)

.

. stset dthdyxtdc, failure(deathtdc) id(SUBJID)

Survival-time data settings

ID variable: SUBJID

Failure event: deathtdc!=0 & deathtdc<.

Observed time interval: (dthdyxtdc[_n-1], dthdyxtdc]

Exit on or before: failure

--------------------------------------------------------------------------

24,923 total observations

0 exclusions

--------------------------------------------------------------------------

24,923 observations remaining, representing

106 subjects

100 failures in single-failure-per-subject data

24,923 total analysis time at risk and under observation

At risk from t = 0

Earliest observed entry t = 0

Last observed exit t = 784

. streg trtnew AGE becogstrat diagtype BILIULN ASTULN CREATULN ALBULN LDHULN eq5dbase CEAULN

> eq5dmissb regionstrat PDDYLR eq5datprog eq5dind eq5dmissingatprog ecogatprog bestrespatpr

> og respmissingatprog LSSLDatprog LSSLDmissingatprog AATULNatprog AATmissingatprog ALBULNat

> prog ALBmissingatprog ALKULNatprog ALKmissingatprog ASTULNatprog ASTmissingatprog CEAULNat

> prog CEAmissingatprog CREATULNatprog CREATmissingatprog LDHULNatprog LDHmissingatprog BILI

> ULNatprog BILImissingatprog saeatprog, dist(lognormal) time iterate(200)

Failure _d: deathtdc

Analysis time _t: dthdyxtdc

ID variable: SUBJID

note: ALBmissingatprog omitted because of collinearity.

note: ALKmissingatprog omitted because of collinearity.

note: ASTmissingatprog omitted because of collinearity.

note: CREATULNatprog omitted because of collinearity.

note: CREATmissingatprog omitted because of collinearity.

note: LDHmissingatprog omitted because of collinearity.

note: BILImissingatprog omitted because of collinearity.

Fitting constant-only model:

Iteration 0: log likelihood = -222.34803

Iteration 1: log likelihood = -198.01902

Iteration 2: log likelihood = -161.56774

Iteration 3: log likelihood = -160.997

Iteration 4: log likelihood = -160.98957

Iteration 5: log likelihood = -160.98957

Fitting full model:

Iteration 0: log likelihood = -160.98957 (not concave)

Iteration 1: log likelihood = -137.01662

Iteration 2: log likelihood = -121.81787

Iteration 3: log likelihood = -118.32556

Iteration 4: log likelihood = -118.29361

Iteration 5: log likelihood = -118.2936

Lognormal AFT regression

No. of subjects = 106 Number of obs = 24,923

No. of failures = 100

Time at risk = 24,923

LR chi2(32) = 85.39

Log likelihood = -118.2936 Prob > chi2 = 0.0000

------------------------------------------------------------------------------------

_t | Coefficient Std. err. z P>|z| [95% conf. interval]

-------------------+----------------------------------------------------------------

trtnew | .8439232 .224195 3.76 0.000 .404509 1.283337

AGE | -.0028388 .0081509 -0.35 0.728 -.0188142 .0131367

becogstrat | .2509313 .3262883 0.77 0.442 -.388582 .8904446

diagtype | .0355247 .1880266 0.19 0.850 -.3330007 .4040502

BILIULN | .3498442 .273748 1.28 0.201 -.1866921 .8863805

ASTULN | .2321577 .2883656 0.81 0.421 -.3330284 .7973438

CREATULN | -.7470618 .5792957 -1.29 0.197 -1.882461 .3883369

ALBULN | -.7032057 .4959234 -1.42 0.156 -1.675198 .2687863

LDHULN | -.0953833 .3169391 -0.30 0.763 -.7165726 .525806

eq5dbase | -.9751098 .4580464 -2.13 0.033 -1.872864 -.0773555

CEAULN | .5604943 .7095468 0.79 0.430 -.8301918 1.951181

eq5dmissb | -.2254449 .5722768 -0.39 0.694 -1.347087 .8961971

regionstrat | .0606994 .122675 0.49 0.621 -.1797392 .301138

PDDYLR | .0079124 .0042986 1.84 0.066 -.0005126 .0163375

eq5datprog | .5125582 .4178718 1.23 0.220 -.3064555 1.331572

eq5dind | -.1556607 .7364155 -0.21 0.833 -1.599008 1.287687

eq5dmissingatprog | -.0253933 .3522659 -0.07 0.943 -.7158218 .6650353

ecogatprog | -.4815685 .1499394 -3.21 0.001 -.7754443 -.1876927

bestrespatprog | .1726948 .1895416 0.91 0.362 -.1987999 .5441895

respmissingatprog | -.4427165 .2593852 -1.71 0.088 -.9511021 .0656691

LSSLDatprog | -.0015657 .0009261 -1.69 0.091 -.0033808 .0002495

LSSLDmissingatprog | -.3441713 .200155 -1.72 0.086 -.7364679 .0481253

AATULNatprog | .0018967 .2029796 0.01 0.993 -.3959361 .3997295

AATmissingatprog | .0381771 .2961605 0.13 0.897 -.5422869 .6186411

ALBULNatprog | .2559409 .4799405 0.53 0.594 -.6847253 1.196607

ALBmissingatprog | 0 (omitted)

ALKULNatprog | .0494686 .2278724 0.22 0.828 -.397153 .4960902

ALKmissingatprog | 0 (omitted)

ASTULNatprog | -.5193147 .3094655 -1.68 0.093 -1.125856 .0872264

ASTmissingatprog | 0 (omitted)

CEAULNatprog | -.7102453 .7224072 -0.98 0.326 -2.126137 .7056467

CEAmissingatprog | -.0588987 .2524328 -0.23 0.816 -.5536579 .4358605

CREATULNatprog | 0 (omitted)

CREATmissingatprog | 0 (omitted)

LDHULNatprog | .3201592 .3540543 0.90 0.366 -.3737745 1.014093

LDHmissingatprog | 0 (omitted)

BILIULNatprog | -.3495642 .2835107 -1.23 0.218 -.9052349 .2061065

BILImissingatprog | 0 (omitted)

saeatprog | .7110613 .8878692 0.80 0.423 -1.02913 2.451253

_cons | 5.375161 1.243476 4.32 0.000 2.937993 7.81233

-------------------+----------------------------------------------------------------

/lnsigma | -.3294761 .0712512 -4.62 0.000 -.4691259 -.1898264

-------------------+----------------------------------------------------------------

sigma | .7193005 .051251 .6255488 .8271027

------------------------------------------------------------------------------------

. * Same model as for IPCW denominator but without the TDC variables. Note, had to take ecog

> misslastvisit out as !=0 perfectly predicted failure.

. * And, ALBmisslastvisit, ALKmisslastvisit, ASTmisslastvisit, CREATmisslastvisit, LDHmissla

> stvisit, BILImisslastvisit all omitted because of collinearity

.

. scalar tsec_af = exp(_b[trtnew])

. di tsec_af

2.3254723

.

. restore

. sort SUBJID

. preserve

.

. ***Analysis on overall survival***

. collapse (max) trtgrp krasi xo regionstrat becogstrat dthdyxtdc xotime deathtdc admin, by(

> SUBJID)

. by SUBJID: replace xotime=0 if xotime==.

(259 real changes made)

. drop if krasi==1

(184 observations deleted)

. ***below allows for recensoring***

. gen cfact = dthdyxtdc if trtgrp==2

(119 missing values generated)

. gen dcfact = deathtdc if trtgrp==2

(119 missing values generated)

.

. replace cfact = (xotime + ((dthdyxtdc-xotime)/(tsec_af))) if (trtgrp==1 & xotime>0)

(91 real changes made)

. replace cfact = dthdyxtdc if (trtgrp==1 & xotime==0)

(28 real changes made)

. gen OSadminc = admin/(tsec_af) if (trtgrp==1 & (tsec_af)>1.00)

(124 missing values generated)

. replace dcfact = deathtdc if trtgrp==1

(119 real changes made)

. replace dcfact=0 if (OSadminc<=cfact & trtgrp==1)

(0 real changes made)

. replace cfact = OSadminc if (OSadminc<=cfact & trtgrp==1)

(9 real changes made)

.

. ***do survival analysis on re-estimated survival times***

. stset cfact, failure(dcfact) id(SUBJID)

Survival-time data settings

ID variable: SUBJID

Failure event: dcfact!=0 & dcfact<.

Observed time interval: (cfact[_n-1], cfact]

Exit on or before: failure

--------------------------------------------------------------------------

243 total observations

0 exclusions

--------------------------------------------------------------------------

243 observations remaining, representing

243 subjects

217 failures in single-failure-per-subject data

51,272.009 total analysis time at risk and under observation

At risk from t = 0

Earliest observed entry t = 0

Last observed exit t = 1,024

. stcox trtgrp regionstrat becogstrat

Failure _d: dcfact

Analysis time _t: cfact

ID variable: SUBJID

Iteration 0: log likelihood = -998.02931

Iteration 1: log likelihood = -976.70069

Iteration 2: log likelihood = -966.30721

Iteration 3: log likelihood = -966.04828

Iteration 4: log likelihood = -966.04785

Refining estimates:

Iteration 0: log likelihood = -966.04785

Cox regression with Breslow method for ties

No. of subjects = 243 Number of obs = 243

No. of failures = 217

Time at risk = 51,272.0094

LR chi2(3) = 63.96

Log likelihood = -966.04785 Prob > chi2 = 0.0000

------------------------------------------------------------------------------

_t | Haz. ratio Std. err. z P>|z| [95% conf. interval]

-------------+----------------------------------------------------------------

trtgrp | .4147454 .062399 -5.85 0.000 .3088282 .5569883

regionstrat | .927977 .0854996 -0.81 0.417 .7746604 1.111637

becogstrat | 3.424486 .7037212 5.99 0.000 2.28916 5.122886

------------------------------------------------------------------------------

.

. restore

.

. ******************************************************************************************

> ***************

. *** TSEsimp 3 (primary analysis, full model, no recens, Weibull) Estimand 2 [251 from TSEs

> imp file] ***

. ******************************************************************************************

> ***************

.

. preserve

.

. * note, 8 patients switched before investigator observed progression. 7 were kras MT, so p

> rimary analyses will not adjust for these anyway.

. * protocol suggested switching permitted only after progression. So assume these patients

> switched due to some signs of progression.

. * need this, because SNM models will fit much better if applied only to the time-periods w

> here switching was "permitted".

. * so, for these 8 patients, replace PDDYLR to equal xotime

. * and replace progtdc to = 1 after this point for these patients

. sort SUBJID dthdyxtdc

. by SUBJID: replace PDDYLR = xotime if (PDDYLR>xotime & xotime!=.)

(1,252 real changes made)

. by SUBJID: replace progtdc = 1 if (PDDYLR <= dthdyxtdc & PDLR==1)

(984 real changes made)

.

. by SUBJID: replace lastobs = 0

(427 real changes made)

. by SUBJID: replace lastobs = 1 if _n==_N

(427 real changes made)

.

. *** SNM

. sort SUBJID dthdyxtdc

. drop if trtgrp==2

(49,968 observations deleted)

. drop if progtdc==0

(10,866 observations deleted)

. * drop anyone who died on same day as progression

. drop if PDDYLR == deathtime

(16 observations deleted)

. * for primary analysis, get treatment effect comparing WT switchers to WT non-switchers, i

> .e. exclude MT

. drop if krasi==1

(16,044 observations deleted)

. by SUBJID: gen obsno = _n

. by SUBJID: gen trtnew = 0

. by SUBJID: replace trtnew = 1 if xo==1 & dthdyxtdc>=xotime

(22802 real changes made)

. by SUBJID: egen minrisk=min(time)

. by SUBJID: replace dthdyxtdc=dthdyxtdc-minrisk

(24923 real changes made)

. by SUBJID: replace xotime=xotime-minrisk

(24117 real changes made)

. by SUBJID: replace time=time-minrisk

(24923 real changes made)

. by SUBJID: replace admin=admin-minrisk

(24923 real changes made)

.

. * need trtlag indicator, as switch is perfect predictor of being a switcher in the next ob

> servation

. by SUBJID: gen trtlag = trtnew[_n-1]

(106 missing values generated)

. replace trtlag=0 if trtlag==.

(106 real changes made)

.

. stset dthdyxtdc, failure(deathtdc) id(SUBJID)

Survival-time data settings

ID variable: SUBJID

Failure event: deathtdc!=0 & deathtdc<.

Observed time interval: (dthdyxtdc[_n-1], dthdyxtdc]

Exit on or before: failure

--------------------------------------------------------------------------

24,923 total observations

0 exclusions

--------------------------------------------------------------------------

24,923 observations remaining, representing

106 subjects

100 failures in single-failure-per-subject data

24,923 total analysis time at risk and under observation

At risk from t = 0

Earliest observed entry t = 0

Last observed exit t = 784

. streg trtnew AGE becogstrat diagtype BILIULN ASTULN CREATULN ALBULN LDHULN eq5dbase CEAULN

> eq5dmissb regionstrat PDDYLR eq5datprog eq5dind eq5dmissingatprog ecogatprog bestrespatpr

> og respmissingatprog LSSLDatprog LSSLDmissingatprog AATULNatprog AATmissingatprog ALBULNat

> prog ALBmissingatprog ALKULNatprog ALKmissingatprog ASTULNatprog ASTmissingatprog CEAULNat

> prog CEAmissingatprog CREATULNatprog CREATmissingatprog LDHULNatprog LDHmissingatprog BILI

> ULNatprog BILImissingatprog saeatprog, dist(weibull) time iterate(200)

Failure _d: deathtdc

Analysis time _t: dthdyxtdc

ID variable: SUBJID

note: ALBmissingatprog omitted because of collinearity.

note: ALKmissingatprog omitted because of collinearity.

note: ASTmissingatprog omitted because of collinearity.

note: CREATULNatprog omitted because of collinearity.

note: CREATmissingatprog omitted because of collinearity.

note: LDHmissingatprog omitted because of collinearity.

note: BILImissingatprog omitted because of collinearity.

Fitting constant-only model:

Iteration 0: log likelihood = -155.00396

Iteration 1: log likelihood = -153.4072

Iteration 2: log likelihood = -153.40372

Iteration 3: log likelihood = -153.40372

Fitting full model:

Iteration 0: log likelihood = -153.40372

Iteration 1: log likelihood = -121.70482

Iteration 2: log likelihood = -109.88398

Iteration 3: log likelihood = -108.2494

Iteration 4: log likelihood = -108.22721

Iteration 5: log likelihood = -108.22719

Iteration 6: log likelihood = -108.22719

Weibull AFT regression

No. of subjects = 106 Number of obs = 24,923

No. of failures = 100

Time at risk = 24,923

LR chi2(32) = 90.35

Log likelihood = -108.22719 Prob > chi2 = 0.0000

------------------------------------------------------------------------------------

_t | Coefficient Std. err. z P>|z| [95% conf. interval]

-------------------+----------------------------------------------------------------

trtnew | .7925562 .216738 3.66 0.000 .3677574 1.217355

AGE | -.0048295 .0070864 -0.68 0.496 -.0187185 .0090596

becogstrat | .5230229 .2813373 1.86 0.063 -.028388 1.074434

diagtype | .2206367 .1607451 1.37 0.170 -.094418 .5356914

BILIULN | .0355378 .2173681 0.16 0.870 -.3904958 .4615715

ASTULN | .573513 .2583067 2.22 0.026 .0672413 1.079785

CREATULN | -1.204197 .4134357 -2.91 0.004 -2.014516 -.3938775

ALBULN | -.4468884 .3860208 -1.16 0.247 -1.203475 .3096984

LDHULN | .3112406 .2954532 1.05 0.292 -.267837 .8903183

eq5dbase | -.8261484 .3899927 -2.12 0.034 -1.59052 -.0617767

CEAULN | -.2930274 .5818143 -0.50 0.615 -1.433363 .8473077

eq5dmissb | -.2718747 .4762672 -0.57 0.568 -1.205341 .6615919

regionstrat | .0517618 .1067807 0.48 0.628 -.1575245 .2610481

PDDYLR | .0138439 .0040986 3.38 0.001 .0058107 .0218771

eq5datprog | .0695035 .3785514 0.18 0.854 -.6724436 .8114505

eq5dind | .1851247 .6082364 0.30 0.761 -1.006997 1.377246

eq5dmissingatprog | -.0166687 .3018315 -0.06 0.956 -.6082476 .5749102

ecogatprog | -.4862551 .1328997 -3.66 0.000 -.7467337 -.2257765

bestrespatprog | .2345417 .1538745 1.52 0.127 -.0670468 .5361302

respmissingatprog | -.5821665 .2255813 -2.58 0.010 -1.024298 -.1400353

LSSLDatprog | -.0019712 .0007874 -2.50 0.012 -.0035146 -.0004278

LSSLDmissingatprog | -.1649233 .1740925 -0.95 0.343 -.5061384 .1762917

AATULNatprog | -.0258887 .1727636 -0.15 0.881 -.3644992 .3127218

AATmissingatprog | -.4600322 .2435557 -1.89 0.059 -.9373926 .0173282

ALBULNatprog | -.1491619 .3829531 -0.39 0.697 -.8997361 .6014123

ALBmissingatprog | 0 (omitted)

ALKULNatprog | -.1218627 .2242434 -0.54 0.587 -.5613717 .3176463

ALKmissingatprog | 0 (omitted)

ASTULNatprog | -.7328966 .258833 -2.83 0.005 -1.2402 -.2255933

ASTmissingatprog | 0 (omitted)

CEAULNatprog | .3808302 .5993283 0.64 0.525 -.7938317 1.555492

CEAmissingatprog | -.3042879 .2144844 -1.42 0.156 -.7246697 .1160938

CREATULNatprog | 0 (omitted)

CREATmissingatprog | 0 (omitted)

LDHULNatprog | .238702 .3197934 0.75 0.455 -.3880815 .8654855

LDHmissingatprog | 0 (omitted)

BILIULNatprog | -.595671 .2202507 -2.70 0.007 -1.027354 -.1639875

BILImissingatprog | 0 (omitted)

saeatprog | 1.303075 .6652494 1.96 0.050 -.0007896 2.60694

_cons | 5.040682 1.000408 5.04 0.000 3.079918 7.001445

-------------------+----------------------------------------------------------------

/ln_p | .6606433 .0842989 7.84 0.000 .4954204 .8258662

-------------------+----------------------------------------------------------------

p | 1.936037 .1632059 1.641188 2.283858

1/p | .5165189 .043542 .4378556 .6093147

------------------------------------------------------------------------------------

.

. * Same model as for IPCW denominator. Note, had to take ecogmisslastvisit out as !=0 perfe

> ctly predicted failure.

. * And, ALBmisslastvisit, ALKmisslastvisit, ASTmisslastvisit, CREATmisslastvisit, LDHmissla

> stvisit, BILImisslastvisit all omitted because of collinearity

.

. scalar tsec_af = exp(_b[trtnew])

. di tsec_af

2.2090359

.

. restore

. sort SUBJID

. preserve

.

. ***Analysis on overall survival***

. collapse (max) trtgrp krasi xo regionstrat becogstrat dthdyxtdc xotime deathtdc admin, by(

> SUBJID)

. by SUBJID: replace xotime=0 if xotime==.

(259 real changes made)

. drop if krasi==1

(184 observations deleted)

.

. ***below no recensoring***

. gen cfact = dthdyxtdc if trtgrp==2

(119 missing values generated)

. gen dcfact = deathtdc if trtgrp==2

(119 missing values generated)

.

. replace cfact = (xotime + ((dthdyxtdc-xotime)/(tsec_af))) if (trtgrp==1 & xotime>0)

(91 real changes made)

. replace cfact = dthdyxtdc if (trtgrp==1 & xotime==0)

(28 real changes made)

. replace dcfact = deathtdc if trtgrp==1

(119 real changes made)

.

. ***do survival analysis on re-estimated survival times***

. stset cfact, failure(dcfact) id(SUBJID)

Survival-time data settings

ID variable: SUBJID

Failure event: dcfact!=0 & dcfact<.

Observed time interval: (cfact[_n-1], cfact]

Exit on or before: failure

--------------------------------------------------------------------------

243 total observations

0 exclusions

--------------------------------------------------------------------------

243 observations remaining, representing

243 subjects

217 failures in single-failure-per-subject data

52,671.955 total analysis time at risk and under observation

At risk from t = 0

Earliest observed entry t = 0

Last observed exit t = 1,024

.

. stcox trtgrp regionstrat becogstrat

Failure _d: dcfact

Analysis time _t: cfact

ID variable: SUBJID

Iteration 0: log likelihood = -1001.9382

Iteration 1: log likelihood = -985.7533

Iteration 2: log likelihood = -975.47568

Iteration 3: log likelihood = -975.22364

Iteration 4: log likelihood = -975.22323

Refining estimates:

Iteration 0: log likelihood = -975.22323

Cox regression with Breslow method for ties

No. of subjects = 243 Number of obs = 243

No. of failures = 217

Time at risk = 52,671.9554

LR chi2(3) = 53.43

Log likelihood = -975.22323 Prob > chi2 = 0.0000

------------------------------------------------------------------------------

_t | Haz. ratio Std. err. z P>|z| [95% conf. interval]

-------------+----------------------------------------------------------------

trtgrp | .4943363 .0703404 -4.95 0.000 .3740269 .6533445

regionstrat | .9352008 .0867718 -0.72 0.470 .7796989 1.121716

becogstrat | 3.415131 .7011134 5.98 0.000 2.283804 5.106882

------------------------------------------------------------------------------

.

. restore

.

. ******************************************************************************************

> ***************

. *** TSEsimp 4 (primary analysis, full model, no recens, lognormal) Estimand 2 ***

. ******************************************************************************************

> ***************

.

. preserve

.

. * note, 8 patients switched before investigator observed progression. 7 were kras MT, so p

> rimary analyses will not adjust for these anyway.

. * protocol suggested switching permitted only after progression. So assume these patients

> switched due to some signs of progression.

. * need this, because SNM models will fit much better if applied only to the time-periods w

> here switching was "permitted".

. * so, for these 8 patients, replace PDDYLR to equal xotime

. * and replace progtdc to = 1 after this point for these patients

. sort SUBJID dthdyxtdc

. by SUBJID: replace PDDYLR = xotime if (PDDYLR>xotime & xotime!=.)

(1,252 real changes made)

. by SUBJID: replace progtdc = 1 if (PDDYLR <= dthdyxtdc & PDLR==1)

(984 real changes made)

.

. replace xotdc = 0 if krasi==1 & trtgrp==1

(14,415 real changes made)

. replace xo = . if krasi==1 & trtgrp==1 & xo==1

(18,500 real changes made, 18,500 to missing)

.

. by SUBJID: replace lastobs = 0

(427 real changes made)

. by SUBJID: replace lastobs = 1 if _n==_N

(427 real changes made)

.

. *** SNM

. sort SUBJID dthdyxtdc

. drop if trtgrp==2

(49,968 observations deleted)

. drop if progtdc==0

(10,866 observations deleted)

. * drop anyone who died on same day as progression

. drop if PDDYLR == deathtime

(16 observations deleted)

. * for primary analysis, get treatment effect comparing WT switchers to WT non-switchers, i

> .e. exclude MT

. drop if krasi==1

(16,044 observations deleted)

. by SUBJID: gen obsno = _n

. by SUBJID: gen trtnew = 0

. by SUBJID: replace trtnew = 1 if xo==1 & dthdyxtdc>=xotime

(22802 real changes made)

. by SUBJID: egen minrisk=min(time)

. by SUBJID: replace dthdyxtdc=dthdyxtdc-minrisk

(24923 real changes made)

. by SUBJID: replace xotime=xotime-minrisk

(24117 real changes made)

. by SUBJID: replace time=time-minrisk

(24923 real changes made)

. by SUBJID: replace admin=admin-minrisk

(24923 real changes made)

.

. * need trtlag indicator, as switch is perfect predictor of being a switcher in the next ob

> servation

. by SUBJID: gen trtlag = trtnew[_n-1]

(106 missing values generated)

. replace trtlag=0 if trtlag==.

(106 real changes made)

.

. stset dthdyxtdc, failure(deathtdc) id(SUBJID)

Survival-time data settings

ID variable: SUBJID

Failure event: deathtdc!=0 & deathtdc<.

Observed time interval: (dthdyxtdc[_n-1], dthdyxtdc]

Exit on or before: failure

--------------------------------------------------------------------------

24,923 total observations

0 exclusions

--------------------------------------------------------------------------

24,923 observations remaining, representing

106 subjects

100 failures in single-failure-per-subject data

24,923 total analysis time at risk and under observation

At risk from t = 0

Earliest observed entry t = 0

Last observed exit t = 784

. streg trtnew AGE becogstrat diagtype BILIULN ASTULN CREATULN ALBULN LDHULN eq5dbase CEAULN

> eq5dmissb regionstrat PDDYLR eq5datprog eq5dind eq5dmissingatprog ecogatprog bestrespatpr

> og respmissingatprog LSSLDatprog LSSLDmissingatprog AATULNatprog AATmissingatprog ALBULNat

> prog ALBmissingatprog ALKULNatprog ALKmissingatprog ASTULNatprog ASTmissingatprog CEAULNat

> prog CEAmissingatprog CREATULNatprog CREATmissingatprog LDHULNatprog LDHmissingatprog BILI

> ULNatprog BILImissingatprog saeatprog, dist(lognormal) time iterate(200)

Failure _d: deathtdc

Analysis time _t: dthdyxtdc

ID variable: SUBJID

note: ALBmissingatprog omitted because of collinearity.

note: ALKmissingatprog omitted because of collinearity.

note: ASTmissingatprog omitted because of collinearity.

note: CREATULNatprog omitted because of collinearity.

note: CREATmissingatprog omitted because of collinearity.

note: LDHmissingatprog omitted because of collinearity.

note: BILImissingatprog omitted because of collinearity.

Fitting constant-only model:

Iteration 0: log likelihood = -222.34803

Iteration 1: log likelihood = -198.01902

Iteration 2: log likelihood = -161.56774

Iteration 3: log likelihood = -160.997

Iteration 4: log likelihood = -160.98957

Iteration 5: log likelihood = -160.98957

Fitting full model:

Iteration 0: log likelihood = -160.98957 (not concave)

Iteration 1: log likelihood = -137.01662

Iteration 2: log likelihood = -121.81787

Iteration 3: log likelihood = -118.32556

Iteration 4: log likelihood = -118.29361

Iteration 5: log likelihood = -118.2936

Lognormal AFT regression

No. of subjects = 106 Number of obs = 24,923

No. of failures = 100

Time at risk = 24,923

LR chi2(32) = 85.39

Log likelihood = -118.2936 Prob > chi2 = 0.0000

------------------------------------------------------------------------------------

_t | Coefficient Std. err. z P>|z| [95% conf. interval]

-------------------+----------------------------------------------------------------

trtnew | .8439232 .224195 3.76 0.000 .404509 1.283337

AGE | -.0028388 .0081509 -0.35 0.728 -.0188142 .0131367

becogstrat | .2509313 .3262883 0.77 0.442 -.388582 .8904446

diagtype | .0355247 .1880266 0.19 0.850 -.3330007 .4040502

BILIULN | .3498442 .273748 1.28 0.201 -.1866921 .8863805

ASTULN | .2321577 .2883656 0.81 0.421 -.3330284 .7973438

CREATULN | -.7470618 .5792957 -1.29 0.197 -1.882461 .3883369

ALBULN | -.7032057 .4959234 -1.42 0.156 -1.675198 .2687863

LDHULN | -.0953833 .3169391 -0.30 0.763 -.7165726 .525806

eq5dbase | -.9751098 .4580464 -2.13 0.033 -1.872864 -.0773555

CEAULN | .5604943 .7095468 0.79 0.430 -.8301918 1.951181

eq5dmissb | -.2254449 .5722768 -0.39 0.694 -1.347087 .8961971

regionstrat | .0606994 .122675 0.49 0.621 -.1797392 .301138

PDDYLR | .0079124 .0042986 1.84 0.066 -.0005126 .0163375

eq5datprog | .5125582 .4178718 1.23 0.220 -.3064555 1.331572

eq5dind | -.1556607 .7364155 -0.21 0.833 -1.599008 1.287687

eq5dmissingatprog | -.0253933 .3522659 -0.07 0.943 -.7158218 .6650353

ecogatprog | -.4815685 .1499394 -3.21 0.001 -.7754443 -.1876927

bestrespatprog | .1726948 .1895416 0.91 0.362 -.1987999 .5441895

respmissingatprog | -.4427165 .2593852 -1.71 0.088 -.9511021 .0656691

LSSLDatprog | -.0015657 .0009261 -1.69 0.091 -.0033808 .0002495

LSSLDmissingatprog | -.3441713 .200155 -1.72 0.086 -.7364679 .0481253

AATULNatprog | .0018967 .2029796 0.01 0.993 -.3959361 .3997295

AATmissingatprog | .0381771 .2961605 0.13 0.897 -.5422869 .6186411

ALBULNatprog | .2559409 .4799405 0.53 0.594 -.6847253 1.196607

ALBmissingatprog | 0 (omitted)

ALKULNatprog | .0494686 .2278724 0.22 0.828 -.397153 .4960902

ALKmissingatprog | 0 (omitted)

ASTULNatprog | -.5193147 .3094655 -1.68 0.093 -1.125856 .0872264

ASTmissingatprog | 0 (omitted)

CEAULNatprog | -.7102453 .7224072 -0.98 0.326 -2.126137 .7056467

CEAmissingatprog | -.0588987 .2524328 -0.23 0.816 -.5536579 .4358605

CREATULNatprog | 0 (omitted)

CREATmissingatprog | 0 (omitted)

LDHULNatprog | .3201592 .3540543 0.90 0.366 -.3737745 1.014093

LDHmissingatprog | 0 (omitted)

BILIULNatprog | -.3495642 .2835107 -1.23 0.218 -.9052349 .2061065

BILImissingatprog | 0 (omitted)

saeatprog | .7110613 .8878692 0.80 0.423 -1.02913 2.451253

_cons | 5.375161 1.243476 4.32 0.000 2.937993 7.81233

-------------------+----------------------------------------------------------------

/lnsigma | -.3294761 .0712512 -4.62 0.000 -.4691259 -.1898264

-------------------+----------------------------------------------------------------

sigma | .7193005 .051251 .6255488 .8271027

------------------------------------------------------------------------------------

.

. * Same model as for IPCW denominator. Note, had to take ecogmisslastvisit out as !=0 perfe

> ctly predicted failure.

. * And, ALBmisslastvisit, ALKmisslastvisit, ASTmisslastvisit, CREATmisslastvisit, LDHmissla

> stvisit, BILImisslastvisit all omitted because of collinearity

.

. scalar tsec_af = exp(_b[trtnew])

. di tsec_af

2.3254723

.

. restore

. sort SUBJID

. preserve

.

. ***Analysis on overall survival***

. collapse (max) trtgrp krasi xo regionstrat becogstrat dthdyxtdc xotime deathtdc admin, by(

> SUBJID)

. by SUBJID: replace xotime=0 if xotime==.

(259 real changes made)

. drop if krasi==1

(184 observations deleted)

.

. ***below no recensoring***

. gen cfact = dthdyxtdc if trtgrp==2

(119 missing values generated)

. gen dcfact = deathtdc if trtgrp==2

(119 missing values generated)

.

. replace cfact = (xotime + ((dthdyxtdc-xotime)/(tsec_af))) if (trtgrp==1 & xotime>0)

(91 real changes made)

. replace cfact = dthdyxtdc if (trtgrp==1 & xotime==0)

(28 real changes made)

. replace dcfact = deathtdc if trtgrp==1

(119 real changes made)

.

. ***do survival analysis on re-estimated survival times***

. stset cfact, failure(dcfact) id(SUBJID)

Survival-time data settings

ID variable: SUBJID

Failure event: dcfact!=0 & dcfact<.

Observed time interval: (cfact[_n-1], cfact]

Exit on or before: failure

--------------------------------------------------------------------------

243 total observations

0 exclusions

--------------------------------------------------------------------------

243 observations remaining, representing

243 subjects

217 failures in single-failure-per-subject data

52,157.188 total analysis time at risk and under observation

At risk from t = 0

Earliest observed entry t = 0

Last observed exit t = 1,024

.

. stcox trtgrp regionstrat becogstrat

Failure _d: dcfact

Analysis time _t: cfact

ID variable: SUBJID

Iteration 0: log likelihood = -1001.6233

Iteration 1: log likelihood = -983.87589

Iteration 2: log likelihood = -973.49218

Iteration 3: log likelihood = -973.23692

Iteration 4: log likelihood = -973.23651

Refining estimates:

Iteration 0: log likelihood = -973.23651

Cox regression with Breslow method for ties

No. of subjects = 243 Number of obs = 243

No. of failures = 217

Time at risk = 52,157.1881

LR chi2(3) = 56.77

Log likelihood = -973.23651 Prob > chi2 = 0.0000

------------------------------------------------------------------------------

_t | Haz. ratio Std. err. z P>|z| [95% conf. interval]

-------------+----------------------------------------------------------------

trtgrp | .4700579 .0674246 -5.26 0.000 .3548591 .622654

regionstrat | .9297029 .0863822 -0.78 0.433 .7749182 1.115405

becogstrat | 3.44383 .7075039 6.02 0.000 2.302343 5.151259

------------------------------------------------------------------------------

.

. restore

.

. ******************************************************************************************

> ***************

. *** TSEsimp 5 (primary analysis, reduced model, with recens, Weibull) Estimand 2 [253 from

> TSEsimp file] ***

. ******************************************************************************************

> ***************

. preserve

.

. * note, 8 patients switched before investigator observed progression. 7 were kras MT, so p

> rimary analyses will not adjust for these anyway.

. * protocol suggested switching permitted only after progression. So assume these patients

> switched due to some signs of progression.

. * need this, because SNM models will fit much better if applied only to the time-periods w

> here switching was "permitted".

. * so, for these 8 patients, replace PDDYLR to equal xotime

. * and replace progtdc to = 1 after this point for these patients

. sort SUBJID dthdyxtdc

. by SUBJID: replace PDDYLR = xotime if (PDDYLR>xotime & xotime!=.)

(1,252 real changes made)

. by SUBJID: replace progtdc = 1 if (PDDYLR <= dthdyxtdc & PDLR==1)

(984 real changes made)

.

. by SUBJID: replace lastobs = 0

(427 real changes made)

. by SUBJID: replace lastobs = 1 if _n==_N

(427 real changes made)

.

. *** streg

. sort SUBJID dthdyxtdc

. drop if trtgrp==2

(49,968 observations deleted)

. drop if progtdc==0

(10,866 observations deleted)

. * drop anyone who died on same day as progression

. drop if PDDYLR == deathtime

(16 observations deleted)

. * for primary analysis, get treatment effect comparing WT switchers to WT non-switchers, i

> .e. exclude MT

. drop if krasi==1

(16,044 observations deleted)

. by SUBJID: gen obsno = _n

. by SUBJID: gen trtnew = 0

. by SUBJID: replace trtnew = 1 if xo==1 & dthdyxtdc>=xotime

(22802 real changes made)

. by SUBJID: egen minrisk=min(time)

. by SUBJID: replace dthdyxtdc=dthdyxtdc-minrisk

(24923 real changes made)

. by SUBJID: replace xotime=xotime-minrisk

(24117 real changes made)

. by SUBJID: replace time=time-minrisk

(24923 real changes made)

. by SUBJID: replace admin=admin-minrisk

(24923 real changes made)

.

. stset dthdyxtdc, failure(deathtdc) id(SUBJID)

Survival-time data settings

ID variable: SUBJID

Failure event: deathtdc!=0 & deathtdc<.

Observed time interval: (dthdyxtdc[_n-1], dthdyxtdc]

Exit on or before: failure

--------------------------------------------------------------------------

24,923 total observations

0 exclusions

--------------------------------------------------------------------------

24,923 observations remaining, representing

106 subjects

100 failures in single-failure-per-subject data

24,923 total analysis time at risk and under observation

At risk from t = 0

Earliest observed entry t = 0

Last observed exit t = 784

. streg trtnew becogstrat diagtype eq5dbase eq5dmissb regionstrat PDDYLR eq5datprog eq5dind

> eq5dmissingatprog ecogatprog bestrespatprog respmissingatprog LSSLDatprog LSSLDmissingatpr

> og, dist(weibull) time iterate(200)

Failure _d: deathtdc

Analysis time _t: dthdyxtdc

ID variable: SUBJID

Fitting constant-only model:

Iteration 0: log likelihood = -155.00396

Iteration 1: log likelihood = -153.4072

Iteration 2: log likelihood = -153.40372

Iteration 3: log likelihood = -153.40372

Fitting full model:

Iteration 0: log likelihood = -153.40372

Iteration 1: log likelihood = -136.61921

Iteration 2: log likelihood = -124.21975

Iteration 3: log likelihood = -123.98834

Iteration 4: log likelihood = -123.98772

Iteration 5: log likelihood = -123.98772

Weibull AFT regression

No. of subjects = 106 Number of obs = 24,923

No. of failures = 100

Time at risk = 24,923

LR chi2(15) = 58.83

Log likelihood = -123.98772 Prob > chi2 = 0.0000

------------------------------------------------------------------------------------

_t | Coefficient Std. err. z P>|z| [95% conf. interval]

-------------------+----------------------------------------------------------------

trtnew | .9398798 .2216702 4.24 0.000 .5054141 1.374346

becogstrat | .2740033 .2863727 0.96 0.339 -.2872768 .8352835

diagtype | .0582467 .1484566 0.39 0.695 -.2327229 .3492162

eq5dbase | -.8388337 .3828969 -2.19 0.028 -1.589298 -.0883696

eq5dmissb | -.908241 .4555363 -1.99 0.046 -1.801076 -.0154063

regionstrat | -.0122116 .1021417 -0.12 0.905 -.2124056 .1879825

PDDYLR | .0104706 .003504 2.99 0.003 .003603 .0173383

eq5datprog | .1290311 .3465396 0.37 0.710 -.5501741 .8082363

eq5dind | -.3294473 .6215387 -0.53 0.596 -1.547641 .8887461

eq5dmissingatprog | .1331492 .2937642 0.45 0.650 -.4426181 .7089165

ecogatprog | -.4896557 .1358889 -3.60 0.000 -.7559931 -.2233184

bestrespatprog | .2451107 .1612552 1.52 0.129 -.0709438 .5611651

respmissingatprog | -.3421478 .2340022 -1.46 0.144 -.8007837 .1164881

LSSLDatprog | -.0013835 .0006297 -2.20 0.028 -.0026177 -.0001494

LSSLDmissingatprog | -.2766897 .1614798 -1.71 0.087 -.5931843 .0398049

_cons | 5.10881 .8325282 6.14 0.000 3.477085 6.740535

-------------------+----------------------------------------------------------------

/ln_p | .4852643 .0799426 6.07 0.000 .3285797 .6419489

-------------------+----------------------------------------------------------------

p | 1.624604 .1298751 1.388994 1.90018

1/p | .6155345 .0492074 .5262658 .7199456

------------------------------------------------------------------------------------

. * Same model as for IPCW denominator except without tdc terms. Note, had to take ecogmissl

> astvisit out as !=0 perfectly predicted failure.

.

. scalar tsec_af = exp(_b[trtnew])

. di tsec_af

2.5596738

.

. restore

. sort SUBJID

. preserve

.

. ***Analysis on overall survival***

. collapse (max) trtgrp krasi xo regionstrat becogstrat dthdyxtdc xotime deathtdc admin, by(

> SUBJID)

. by SUBJID: replace xotime=0 if xotime==.

(259 real changes made)

. drop if krasi==1

(184 observations deleted)

.

. ***below allows for recensoring***

. gen cfact = dthdyxtdc if trtgrp==2

(119 missing values generated)

. gen dcfact = deathtdc if trtgrp==2

(119 missing values generated)

.

. replace cfact = (xotime + ((dthdyxtdc-xotime)/(tsec_af))) if (trtgrp==1 & xotime>0)

(91 real changes made)

. replace cfact = dthdyxtdc if (trtgrp==1 & xotime==0)

(28 real changes made)

. gen OSadminc = admin/(tsec_af) if (trtgrp==1 & (tsec_af)>1.00)

(124 missing values generated)

. replace dcfact = deathtdc if trtgrp==1

(119 real changes made)

. replace dcfact=0 if (OSadminc<=cfact & trtgrp==1)

(1 real change made)

. replace cfact = OSadminc if (OSadminc<=cfact & trtgrp==1)

(10 real changes made)

.

. ***do survival analysis on re-estimated survival times***

. stset cfact, failure(dcfact) id(SUBJID)

Survival-time data settings

ID variable: SUBJID

Failure event: dcfact!=0 & dcfact<.

Observed time interval: (cfact[_n-1], cfact]

Exit on or before: failure

--------------------------------------------------------------------------

243 total observations

0 exclusions

--------------------------------------------------------------------------

243 observations remaining, representing

243 subjects

216 failures in single-failure-per-subject data

50,315.199 total analysis time at risk and under observation

At risk from t = 0

Earliest observed entry t = 0

Last observed exit t = 1,024

.

. stcox trtgrp regionstrat becogstrat

Failure _d: dcfact

Analysis time _t: cfact

ID variable: SUBJID

Iteration 0: log likelihood = -993.37992

Iteration 1: log likelihood = -968.39831

Iteration 2: log likelihood = -958.07919

Iteration 3: log likelihood = -957.85291

Iteration 4: log likelihood = -957.8526

Refining estimates:

Iteration 0: log likelihood = -957.8526

Cox regression with Breslow method for ties

No. of subjects = 243 Number of obs = 243

No. of failures = 216

Time at risk = 50,315.1993

LR chi2(3) = 71.05

Log likelihood = -957.8526 Prob > chi2 = 0.0000

------------------------------------------------------------------------------

_t | Haz. ratio Std. err. z P>|z| [95% conf. interval]

-------------+----------------------------------------------------------------

trtgrp | .3735925 .0576218 -6.38 0.000 .2761286 .5054578

regionstrat | .9361205 .0861585 -0.72 0.473 .7816081 1.121178

becogstrat | 3.580779 .7390815 6.18 0.000 2.389392 5.366209

------------------------------------------------------------------------------

.

. restore

.

. ******************************************************************************************

> ***************

. *** TSEsimp 6 (primary analysis, reduced model, with recens, Gen Gamma) Estimand 2 ***

. ******************************************************************************************

> ***************

. preserve

.

. * note, 8 patients switched before investigator observed progression. 7 were kras MT, so p

> rimary analyses will not adjust for these anyway.

. * protocol suggested switching permitted only after progression. So assume these patients

> switched due to some signs of progression.

. * need this, because SNM models will fit much better if applied only to the time-periods w

> here switching was "permitted".

. * so, for these 8 patients, replace PDDYLR to equal xotime

. * and replace progtdc to = 1 after this point for these patients

. sort SUBJID dthdyxtdc

. by SUBJID: replace PDDYLR = xotime if (PDDYLR>xotime & xotime!=.)

(1,252 real changes made)

. by SUBJID: replace progtdc = 1 if (PDDYLR <= dthdyxtdc & PDLR==1)

(984 real changes made)

.

. by SUBJID: replace lastobs = 0

(427 real changes made)

. by SUBJID: replace lastobs = 1 if _n==_N

(427 real changes made)

.

. *** streg

. sort SUBJID dthdyxtdc

. drop if trtgrp==2

(49,968 observations deleted)

. drop if progtdc==0

(10,866 observations deleted)

. * drop anyone who died on same day as progression

. drop if PDDYLR == deathtime

(16 observations deleted)

. * for primary analysis, get treatment effect comparing WT switchers to WT non-switchers, i

> .e. exclude MT

. drop if krasi==1

(16,044 observations deleted)

. by SUBJID: gen obsno = _n

. by SUBJID: gen trtnew = 0

. by SUBJID: replace trtnew = 1 if xo==1 & dthdyxtdc>=xotime

(22802 real changes made)

. by SUBJID: egen minrisk=min(time)

. by SUBJID: replace dthdyxtdc=dthdyxtdc-minrisk

(24923 real changes made)

. by SUBJID: replace xotime=xotime-minrisk

(24117 real changes made)

. by SUBJID: replace time=time-minrisk

(24923 real changes made)

. by SUBJID: replace admin=admin-minrisk

(24923 real changes made)

.

. stset dthdyxtdc, failure(deathtdc) id(SUBJID)

Survival-time data settings

ID variable: SUBJID

Failure event: deathtdc!=0 & deathtdc<.

Observed time interval: (dthdyxtdc[_n-1], dthdyxtdc]

Exit on or before: failure

--------------------------------------------------------------------------

24,923 total observations

0 exclusions

--------------------------------------------------------------------------

24,923 observations remaining, representing

106 subjects

100 failures in single-failure-per-subject data

24,923 total analysis time at risk and under observation

At risk from t = 0

Earliest observed entry t = 0

Last observed exit t = 784

. streg trtnew becogstrat diagtype eq5dbase eq5dmissb regionstrat PDDYLR eq5datprog eq5dind

> eq5dmissingatprog ecogatprog bestrespatprog respmissingatprog LSSLDatprog LSSLDmissingatpr

> og, dist(ggamma) time iterate(200)

Failure _d: deathtdc

Analysis time _t: dthdyxtdc

ID variable: SUBJID

Fitting constant-only model:

Iteration 0: log likelihood = -580.23762 (not concave)

Iteration 1: log likelihood = -226.73916 (not concave)

Iteration 2: log likelihood = -160.10683

Iteration 3: log likelihood = -156.87958

Iteration 4: log likelihood = -153.47437

Iteration 5: log likelihood = -153.32015

Iteration 6: log likelihood = -153.31691

Iteration 7: log likelihood = -153.31691

Fitting full model:

Iteration 0: log likelihood = -153.31691 (not concave)

Iteration 1: log likelihood = -139.92273 (not concave)

Iteration 2: log likelihood = -134.69516 (not concave)

Iteration 3: log likelihood = -132.09858 (not concave)

Iteration 4: log likelihood = -129.45604

Iteration 5: log likelihood = -126.09495

Iteration 6: log likelihood = -124.22244

Iteration 7: log likelihood = -123.97936

Iteration 8: log likelihood = -123.96834

Iteration 9: log likelihood = -123.96833

Generalized gamma AFT regression

No. of subjects = 106 Number of obs = 24,923

No. of failures = 100

Time at risk = 24,923

LR chi2(15) = 58.70

Log likelihood = -123.96833 Prob > chi2 = 0.0000

------------------------------------------------------------------------------------

_t | Coefficient Std. err. z P>|z| [95% conf. interval]

-------------------+----------------------------------------------------------------

trtnew | .9202868 .2501288 3.68 0.000 .4300433 1.41053

becogstrat | .3145031 .3656033 0.86 0.390 -.4020662 1.031073

diagtype | .0811653 .1935202 0.42 0.675 -.2981274 .460458

eq5dbase | -.8475395 .3802808 -2.23 0.026 -1.592876 -.1022028

eq5dmissb | -.9329829 .4640518 -2.01 0.044 -1.842508 -.0234582

regionstrat | -.0296866 .1402058 -0.21 0.832 -.3044849 .2451116

PDDYLR | .0108213 .0039915 2.71 0.007 .0029982 .0186445

eq5datprog | .1081325 .3683005 0.29 0.769 -.6137232 .8299882

eq5dind | -.2872059 .6715222 -0.43 0.669 -1.603365 1.028953

eq5dmissingatprog | .154406 .3281451 0.47 0.638 -.4887465 .7975585

ecogatprog | -.498531 .1446274 -3.45 0.001 -.7819954 -.2150666

bestrespatprog | .2550848 .1673917 1.52 0.128 -.072997 .5831665

respmissingatprog | -.3347191 .2352875 -1.42 0.155 -.7958742 .126436

LSSLDatprog | -.0012841 .0008177 -1.57 0.116 -.0028867 .0003185

LSSLDmissingatprog | -.2729895 .1612406 -1.69 0.090 -.5890153 .0430364

_cons | 5.050585 .8878638 5.69 0.000 3.310404 6.790766

-------------------+----------------------------------------------------------------

/lnsigma | -.5277874 .2617886 -2.02 0.044 -1.040884 -.0146911

/kappa | 1.138928 .7880484 1.45 0.148 -.405618 2.683475

-------------------+----------------------------------------------------------------

sigma | .5899088 .1544314 .3531425 .9854163

------------------------------------------------------------------------------------

. * Same model as for IPCW denominator except without tdc terms. Note, had to take ecogmissl

> astvisit out as !=0 perfectly predicted failure.

.

. scalar tsec_af = exp(_b[trtnew])

. di tsec_af

2.5100102

.

. restore

. sort SUBJID

. preserve

.

. ***Analysis on overall survival***

. collapse (max) trtgrp krasi xo regionstrat becogstrat dthdyxtdc xotime deathtdc admin, by(

> SUBJID)

. by SUBJID: replace xotime=0 if xotime==.

(259 real changes made)

. drop if krasi==1

(184 observations deleted)

.

. ***below allows for recensoring***

. gen cfact = dthdyxtdc if trtgrp==2

(119 missing values generated)

. gen dcfact = deathtdc if trtgrp==2

(119 missing values generated)

.

. replace cfact = (xotime + ((dthdyxtdc-xotime)/(tsec_af))) if (trtgrp==1 & xotime>0)

(91 real changes made)

. replace cfact = dthdyxtdc if (trtgrp==1 & xotime==0)

(28 real changes made)

. gen OSadminc = admin/(tsec_af) if (trtgrp==1 & (tsec_af)>1.00)

(124 missing values generated)

. replace dcfact = deathtdc if trtgrp==1

(119 real changes made)

. replace dcfact=0 if (OSadminc<=cfact & trtgrp==1)

(1 real change made)

. replace cfact = OSadminc if (OSadminc<=cfact & trtgrp==1)

(10 real changes made)

.

. ***do survival analysis on re-estimated survival times***

. stset cfact, failure(dcfact) id(SUBJID)

Survival-time data settings

ID variable: SUBJID

Failure event: dcfact!=0 & dcfact<.

Observed time interval: (cfact[_n-1], cfact]

Exit on or before: failure

--------------------------------------------------------------------------

243 total observations

0 exclusions

--------------------------------------------------------------------------

243 observations remaining, representing

243 subjects

216 failures in single-failure-per-subject data

50,503.865 total analysis time at risk and under observation

At risk from t = 0

Earliest observed entry t = 0

Last observed exit t = 1,024

.

. stcox trtgrp regionstrat becogstrat

Failure _d: dcfact

Analysis time _t: cfact

ID variable: SUBJID

Iteration 0: log likelihood = -993.63229

Iteration 1: log likelihood = -969.72119

Iteration 2: log likelihood = -959.37174

Iteration 3: log likelihood = -959.13988

Iteration 4: log likelihood = -959.13955

Refining estimates:

Iteration 0: log likelihood = -959.13955

Cox regression with Breslow method for ties

No. of subjects = 243 Number of obs = 243

No. of failures = 216

Time at risk = 50,503.8649

LR chi2(3) = 68.99

Log likelihood = -959.13955 Prob > chi2 = 0.0000

------------------------------------------------------------------------------

_t | Haz. ratio Std. err. z P>|z| [95% conf. interval]

-------------+----------------------------------------------------------------

trtgrp | .3848269 .0588923 -6.24 0.000 .2851025 .5194334

regionstrat | .9384539 .0863525 -0.69 0.490 .7835903 1.123924

becogstrat | 3.55029 .7320999 6.14 0.000 2.369948 5.318495

------------------------------------------------------------------------------

.

. restore

.

. ******************************************************************************************

> ***************

. *** TSEsimp 7 (primary analysis, reduced model, no recens, Weibull) Estimand 2 (Analysis 2

> 55 from TSEsimp)***

. ******************************************************************************************

> ***************

.

. preserve

.

. * note, 8 patients switched before investigator observed progression. 7 were kras MT, so p

> rimary analyses will not adjust for these anyway.

. * protocol suggested switching permitted only after progression. So assume these patients

> switched due to some signs of progression.

. * need this, because SNM models will fit much better if applied only to the time-periods w

> here switching was "permitted".

. * so, for these 8 patients, replace PDDYLR to equal xotime

. * and replace progtdc to = 1 after this point for these patients

. sort SUBJID dthdyxtdc

. by SUBJID: replace PDDYLR = xotime if (PDDYLR>xotime & xotime!=.)

(1,252 real changes made)

. by SUBJID: replace progtdc = 1 if (PDDYLR <= dthdyxtdc & PDLR==1)

(984 real changes made)

.

. by SUBJID: replace lastobs = 0

(427 real changes made)

. by SUBJID: replace lastobs = 1 if _n==_N

(427 real changes made)

.

. *** streg

. sort SUBJID dthdyxtdc

. drop if trtgrp==2

(49,968 observations deleted)

. drop if progtdc==0

(10,866 observations deleted)

. * drop anyone who died on same day as progression

. drop if PDDYLR == deathtime

(16 observations deleted)

. * for primary analysis, get treatment effect comparing WT switchers to WT non-switchers, i

> .e. exclude MT

. drop if krasi==1

(16,044 observations deleted)

. by SUBJID: gen obsno = _n

. by SUBJID: gen trtnew = 0

. by SUBJID: replace trtnew = 1 if xo==1 & dthdyxtdc>=xotime

(22802 real changes made)

. by SUBJID: egen minrisk=min(time)

. by SUBJID: replace dthdyxtdc=dthdyxtdc-minrisk

(24923 real changes made)

. by SUBJID: replace xotime=xotime-minrisk

(24117 real changes made)

. by SUBJID: replace time=time-minrisk

(24923 real changes made)

. by SUBJID: replace admin=admin-minrisk

(24923 real changes made)

.

. stset dthdyxtdc, failure(deathtdc) id(SUBJID)

Survival-time data settings

ID variable: SUBJID

Failure event: deathtdc!=0 & deathtdc<.

Observed time interval: (dthdyxtdc[_n-1], dthdyxtdc]

Exit on or before: failure

--------------------------------------------------------------------------

24,923 total observations

0 exclusions

--------------------------------------------------------------------------

24,923 observations remaining, representing

106 subjects

100 failures in single-failure-per-subject data

24,923 total analysis time at risk and under observation

At risk from t = 0

Earliest observed entry t = 0

Last observed exit t = 784

. streg trtnew becogstrat diagtype eq5dbase eq5dmissb regionstrat PDDYLR eq5datprog eq5dind

> eq5dmissingatprog ecogatprog bestrespatprog respmissingatprog LSSLDatprog LSSLDmissingatpr

> og, dist(weibull) time iterate(200)

Failure _d: deathtdc

Analysis time _t: dthdyxtdc

ID variable: SUBJID

Fitting constant-only model:

Iteration 0: log likelihood = -155.00396

Iteration 1: log likelihood = -153.4072

Iteration 2: log likelihood = -153.40372

Iteration 3: log likelihood = -153.40372

Fitting full model:

Iteration 0: log likelihood = -153.40372

Iteration 1: log likelihood = -136.61921

Iteration 2: log likelihood = -124.21975

Iteration 3: log likelihood = -123.98834

Iteration 4: log likelihood = -123.98772

Iteration 5: log likelihood = -123.98772

Weibull AFT regression

No. of subjects = 106 Number of obs = 24,923

No. of failures = 100

Time at risk = 24,923

LR chi2(15) = 58.83

Log likelihood = -123.98772 Prob > chi2 = 0.0000

------------------------------------------------------------------------------------

_t | Coefficient Std. err. z P>|z| [95% conf. interval]

-------------------+----------------------------------------------------------------

trtnew | .9398798 .2216702 4.24 0.000 .5054141 1.374346

becogstrat | .2740033 .2863727 0.96 0.339 -.2872768 .8352835

diagtype | .0582467 .1484566 0.39 0.695 -.2327229 .3492162

eq5dbase | -.8388337 .3828969 -2.19 0.028 -1.589298 -.0883696

eq5dmissb | -.908241 .4555363 -1.99 0.046 -1.801076 -.0154063

regionstrat | -.0122116 .1021417 -0.12 0.905 -.2124056 .1879825

PDDYLR | .0104706 .003504 2.99 0.003 .003603 .0173383

eq5datprog | .1290311 .3465396 0.37 0.710 -.5501741 .8082363

eq5dind | -.3294473 .6215387 -0.53 0.596 -1.547641 .8887461

eq5dmissingatprog | .1331492 .2937642 0.45 0.650 -.4426181 .7089165

ecogatprog | -.4896557 .1358889 -3.60 0.000 -.7559931 -.2233184

bestrespatprog | .2451107 .1612552 1.52 0.129 -.0709438 .5611651

respmissingatprog | -.3421478 .2340022 -1.46 0.144 -.8007837 .1164881

LSSLDatprog | -.0013835 .0006297 -2.20 0.028 -.0026177 -.0001494

LSSLDmissingatprog | -.2766897 .1614798 -1.71 0.087 -.5931843 .0398049

_cons | 5.10881 .8325282 6.14 0.000 3.477085 6.740535

-------------------+----------------------------------------------------------------

/ln_p | .4852643 .0799426 6.07 0.000 .3285797 .6419489

-------------------+----------------------------------------------------------------

p | 1.624604 .1298751 1.388994 1.90018

1/p | .6155345 .0492074 .5262658 .7199456

------------------------------------------------------------------------------------

.

. * Same model as for IPCW denominator. Note, had to take ecogmisslastvisit out as !=0 perfe

> ctly predicted failure

.

. scalar tsec_af = exp(_b[trtnew])

. di tsec_af

2.5596738

.

. restore

. sort SUBJID

. preserve

.

. ***Analysis on overall survival***

. collapse (max) trtgrp krasi xo regionstrat becogstrat dthdyxtdc xotime deathtdc admin, by(

> SUBJID)

. by SUBJID: replace xotime=0 if xotime==.

(259 real changes made)

. drop if krasi==1

(184 observations deleted)

.

. ***below no recensoring***

. gen cfact = dthdyxtdc if trtgrp==2

(119 missing values generated)

. gen dcfact = deathtdc if trtgrp==2

(119 missing values generated)

.

. replace cfact = (xotime + ((dthdyxtdc-xotime)/(tsec_af))) if (trtgrp==1 & xotime>0)

(91 real changes made)

. replace cfact = dthdyxtdc if (trtgrp==1 & xotime==0)

(28 real changes made)

. replace dcfact = deathtdc if trtgrp==1

(119 real changes made)

.

. ***do survival analysis on re-estimated survival times***

. stset cfact, failure(dcfact) id(SUBJID)

Survival-time data settings

ID variable: SUBJID

Failure event: dcfact!=0 & dcfact<.

Observed time interval: (cfact[_n-1], cfact]

Exit on or before: failure

--------------------------------------------------------------------------

243 total observations

0 exclusions

--------------------------------------------------------------------------

243 observations remaining, representing

243 subjects

217 failures in single-failure-per-subject data

51,263.615 total analysis time at risk and under observation

At risk from t = 0

Earliest observed entry t = 0

Last observed exit t = 1,024

.

. stcox trtgrp regionstrat becogstrat

Failure _d: dcfact

Analysis time _t: cfact

ID variable: SUBJID

Iteration 0: log likelihood = -1001.374

Iteration 1: log likelihood = -980.50935

Iteration 2: log likelihood = -970.13586

Iteration 3: log likelihood = -969.91078

Iteration 4: log likelihood = -969.91047

Refining estimates:

Iteration 0: log likelihood = -969.91047

Cox regression with Breslow method for ties

No. of subjects = 243 Number of obs = 243

No. of failures = 217

Time at risk = 51,263.6148

LR chi2(3) = 62.93

Log likelihood = -969.91047 Prob > chi2 = 0.0000

------------------------------------------------------------------------------

_t | Haz. ratio Std. err. z P>|z| [95% conf. interval]

-------------+----------------------------------------------------------------

trtgrp | .4316025 .0625414 -5.80 0.000 .3248931 .5733599

regionstrat | .9348605 .0869324 -0.72 0.469 .7791011 1.12176

becogstrat | 3.586752 .7399864 6.19 0.000 2.393806 5.374197

------------------------------------------------------------------------------

.

. restore

.

. ******************************************************************************************

> ***************

. *** TSEsimp 8 (primary analysis, reduced model, no recens, Gen Gamma) Estimand 2 ***

. ******************************************************************************************

> ***************

.

. preserve

.

. * note, 8 patients switched before investigator observed progression. 7 were kras MT, so p

> rimary analyses will not adjust for these anyway.

. * protocol suggested switching permitted only after progression. So assume these patients

> switched due to some signs of progression.

. * need this, because SNM models will fit much better if applied only to the time-periods w

> here switching was "permitted".

. * so, for these 8 patients, replace PDDYLR to equal xotime

. * and replace progtdc to = 1 after this point for these patients

. sort SUBJID dthdyxtdc

. by SUBJID: replace PDDYLR = xotime if (PDDYLR>xotime & xotime!=.)

(1,252 real changes made)

. by SUBJID: replace progtdc = 1 if (PDDYLR <= dthdyxtdc & PDLR==1)

(984 real changes made)

.

. by SUBJID: replace lastobs = 0

(427 real changes made)

. by SUBJID: replace lastobs = 1 if _n==_N

(427 real changes made)

.

. *** streg

. sort SUBJID dthdyxtdc

. drop if trtgrp==2

(49,968 observations deleted)

. drop if progtdc==0

(10,866 observations deleted)

. * drop anyone who died on same day as progression

. drop if PDDYLR == deathtime

(16 observations deleted)

. * for primary analysis, get treatment effect comparing WT switchers to WT non-switchers, i

> .e. exclude MT

. drop if krasi==1

(16,044 observations deleted)

. by SUBJID: gen obsno = _n

. by SUBJID: gen trtnew = 0

. by SUBJID: replace trtnew = 1 if xo==1 & dthdyxtdc>=xotime

(22802 real changes made)

. by SUBJID: egen minrisk=min(time)

. by SUBJID: replace dthdyxtdc=dthdyxtdc-minrisk

(24923 real changes made)

. by SUBJID: replace xotime=xotime-minrisk

(24117 real changes made)

. by SUBJID: replace time=time-minrisk

(24923 real changes made)

. by SUBJID: replace admin=admin-minrisk

(24923 real changes made)

.

. stset dthdyxtdc, failure(deathtdc) id(SUBJID)

Survival-time data settings

ID variable: SUBJID

Failure event: deathtdc!=0 & deathtdc<.

Observed time interval: (dthdyxtdc[_n-1], dthdyxtdc]

Exit on or before: failure

--------------------------------------------------------------------------

24,923 total observations

0 exclusions

--------------------------------------------------------------------------

24,923 observations remaining, representing

106 subjects

100 failures in single-failure-per-subject data

24,923 total analysis time at risk and under observation

At risk from t = 0

Earliest observed entry t = 0

Last observed exit t = 784

. streg trtnew becogstrat diagtype eq5dbase eq5dmissb regionstrat PDDYLR eq5datprog eq5dind

> eq5dmissingatprog ecogatprog bestrespatprog respmissingatprog LSSLDatprog LSSLDmissingatpr

> og, dist(ggamma) time iterate(200)

Failure _d: deathtdc

Analysis time _t: dthdyxtdc

ID variable: SUBJID

Fitting constant-only model:

Iteration 0: log likelihood = -580.23762 (not concave)

Iteration 1: log likelihood = -226.73916 (not concave)

Iteration 2: log likelihood = -160.10683

Iteration 3: log likelihood = -156.87958

Iteration 4: log likelihood = -153.47437

Iteration 5: log likelihood = -153.32015

Iteration 6: log likelihood = -153.31691

Iteration 7: log likelihood = -153.31691

Fitting full model:

Iteration 0: log likelihood = -153.31691 (not concave)

Iteration 1: log likelihood = -139.92273 (not concave)

Iteration 2: log likelihood = -134.69516 (not concave)

Iteration 3: log likelihood = -132.09858 (not concave)

Iteration 4: log likelihood = -129.45604

Iteration 5: log likelihood = -126.09495

Iteration 6: log likelihood = -124.22244

Iteration 7: log likelihood = -123.97936

Iteration 8: log likelihood = -123.96834

Iteration 9: log likelihood = -123.96833

Generalized gamma AFT regression

No. of subjects = 106 Number of obs = 24,923

No. of failures = 100

Time at risk = 24,923

LR chi2(15) = 58.70

Log likelihood = -123.96833 Prob > chi2 = 0.0000

------------------------------------------------------------------------------------

_t | Coefficient Std. err. z P>|z| [95% conf. interval]

-------------------+----------------------------------------------------------------

trtnew | .9202868 .2501288 3.68 0.000 .4300433 1.41053

becogstrat | .3145031 .3656033 0.86 0.390 -.4020662 1.031073

diagtype | .0811653 .1935202 0.42 0.675 -.2981274 .460458

eq5dbase | -.8475395 .3802808 -2.23 0.026 -1.592876 -.1022028

eq5dmissb | -.9329829 .4640518 -2.01 0.044 -1.842508 -.0234582

regionstrat | -.0296866 .1402058 -0.21 0.832 -.3044849 .2451116

PDDYLR | .0108213 .0039915 2.71 0.007 .0029982 .0186445

eq5datprog | .1081325 .3683005 0.29 0.769 -.6137232 .8299882

eq5dind | -.2872059 .6715222 -0.43 0.669 -1.603365 1.028953

eq5dmissingatprog | .154406 .3281451 0.47 0.638 -.4887465 .7975585

ecogatprog | -.498531 .1446274 -3.45 0.001 -.7819954 -.2150666

bestrespatprog | .2550848 .1673917 1.52 0.128 -.072997 .5831665

respmissingatprog | -.3347191 .2352875 -1.42 0.155 -.7958742 .126436

LSSLDatprog | -.0012841 .0008177 -1.57 0.116 -.0028867 .0003185

LSSLDmissingatprog | -.2729895 .1612406 -1.69 0.090 -.5890153 .0430364

_cons | 5.050585 .8878638 5.69 0.000 3.310404 6.790766

-------------------+----------------------------------------------------------------

/lnsigma | -.5277874 .2617886 -2.02 0.044 -1.040884 -.0146911

/kappa | 1.138928 .7880484 1.45 0.148 -.405618 2.683475

-------------------+----------------------------------------------------------------

sigma | .5899088 .1544314 .3531425 .9854163

------------------------------------------------------------------------------------

.

. * Same model as for IPCW denominator. Note, had to take ecogmisslastvisit out as !=0 perfe

> ctly predicted failure

.

. scalar tsec_af = exp(_b[trtnew])

. di tsec_af

2.5100102

.

. restore

. sort SUBJID

. preserve

.

. ***Analysis on overall survival***

. collapse (max) trtgrp krasi xo regionstrat becogstrat dthdyxtdc xotime deathtdc admin, by(

> SUBJID)

. by SUBJID: replace xotime=0 if xotime==.

(259 real changes made)

. drop if krasi==1

(184 observations deleted)

.

. ***below no recensoring***

. gen cfact = dthdyxtdc if trtgrp==2

(119 missing values generated)

. gen dcfact = deathtdc if trtgrp==2

(119 missing values generated)

.

. replace cfact = (xotime + ((dthdyxtdc-xotime)/(tsec_af))) if (trtgrp==1 & xotime>0)

(91 real changes made)

. replace cfact = dthdyxtdc if (trtgrp==1 & xotime==0)

(28 real changes made)

. replace dcfact = deathtdc if trtgrp==1

(119 real changes made)

.

. ***do survival analysis on re-estimated survival times***

. stset cfact, failure(dcfact) id(SUBJID)

Survival-time data settings

ID variable: SUBJID

Failure event: dcfact!=0 & dcfact<.

Observed time interval: (cfact[_n-1], cfact]

Exit on or before: failure

--------------------------------------------------------------------------

243 total observations

0 exclusions

--------------------------------------------------------------------------

243 observations remaining, representing

243 subjects

217 failures in single-failure-per-subject data

51,439.17 total analysis time at risk and under observation

At risk from t = 0
[truncated: 753,861 more chars]
